# Copper-catalyzed asymmetric carbonylative hydroallylation of vinylarenes†

**DOI:** 10.1039/d5sc02421h

**Published:** 2025-05-14

**Authors:** Sufang Shao, Yang Yuan, Alban Schmoll, Xiao-Feng Wu

**Affiliations:** a Dalian National Laboratory for Clean Energy, Dalian Institute of Chemical Physics, Chinese Academy of Sciences Dalian 116023 P. R. China xwu2020@dicp.ac.cn; b Leibniz-Institut für Katalyse Rostock 18059 Germany; c State Key Laboratory of Elemento-Organic Chemistry, Nankai University Tianjin 300071 China

## Abstract

We describe a novel and efficient copper-catalyzed carbonylative hydroallylation of vinylarenes, providing a direct route to chiral α,β-unsaturated ketones, which are important compounds in organic synthesis and bioactive molecules. The method employs readily accessible vinylarenes and allylic phosphates, utilizing carbon monoxide as the carbonyl source under mild reaction conditions. The reaction demonstrates a broad substrate scope, including diverse vinylarenes with various functional groups, as well as vinylarenes derived from natural products. Additionally, all four stereoisomers of a chiral allylic alcohol were prepared by employing this strategy, showcasing its versatility in stereodivergent synthesis.

## Abstract 2

An efficient copper-catalyzed carbonylative hydroallylation of vinylarenes has been developed using carbon monoxide as the C1 source.

## Graphical abstract

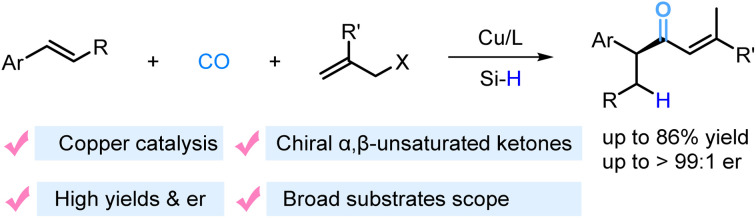

## Introduction

α,β-Unsaturated carbonyl compounds are important intermediates in organic chemistry, extensively used in synthetic transformations and the production of fine and commodity chemicals.^1^ Traditionally, these compounds have been prepared through methods such as the Horner–Wadsworth–Emmons reaction,^2^ aldol condensation,^3^ Knoevenagel condensation,^4^ and Saegusa oxidation.^5^ More recently, alternative methods, including dehydrogenation reactions,^6^ coupling reactions,^7^ and other strategies,^8^ have also been developed for the synthesis of α,β-unsaturated carbonyl compounds. However, most of these methods require a pre-existing carbonyl group in the reactant, which somewhat limits the substrate scope. Consequently, developing practical and efficient methods for their synthesis is still important and desired.

**Fig. 1:**
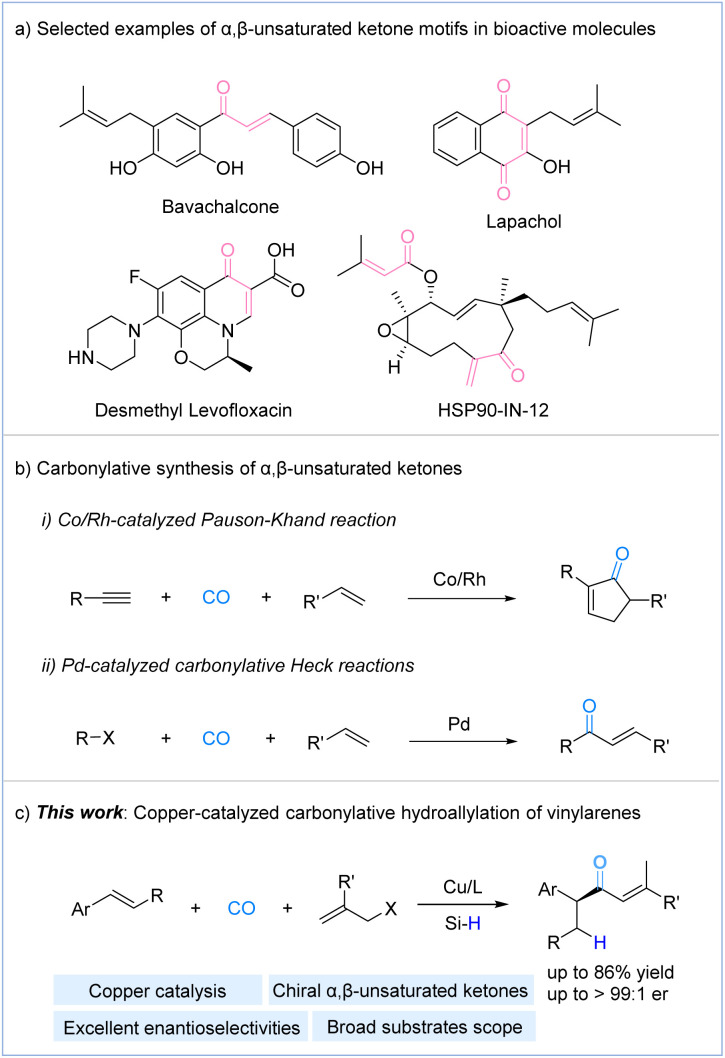
(a) Selected examples of α,β-unsaturated ketone motifs in bioactive molecules; (b) carbonylative synthesis of α,β-unsaturated ketones; (c) this work: copper-catalyzed carbonylative hydroallylation of vinylarenes.

Carbonylation, a process that introduces carbon monoxide (CO) into molecules, has become one of the most direct and efficient methods for synthesizing carbonyl compounds.^9^ Among these, α,β-unsaturated ketones are important intermediates in organic synthesis, widely utilized in various transformations such as Michael addition,^10^ Baylis–Hillman reaction,^11^ epoxidation,^12^ Simmons–Smith reaction,^13^ Diels–Alder reaction,^14^ hydrogenation,^15^ hydroalkoxylation,^16^ and aldol addition.^17^ Additionally, they serve as key structural elements in numerous drug molecules and bioactive compounds (Fig. 1a).^18^ In contrast to other α,β-unsaturated derivatives, such as α,β-unsaturated amides and α,β-unsaturated esters, the carbonylative synthesis of α,β-unsaturated ketones remains relatively challenging and underdeveloped, primarily due to the generally lower reactivity of carbon-based nucleophiles.^19^ The two most important carbonylative methods for synthesizing α,β-unsaturated ketones are the Pauson–Khand reaction^20^ and carbonylative Heck reactions^21^ (Fig. 1b). In addition, Mankad and co-workers presented a copper-catalyzed four-component coupling reaction of alkynes, alkyl halides, B_2_pin_2_, and CO enables modular synthesis of β-borylated tetrasubstituted enones.^22^ Alexanian group reported a cobalt-catalyzed stereospecific carbonylative coupling of alkyl tosylates and dienes producing enantioenriched enones.^23^

Recently, our group developed copper-catalyzed hydroaminocarbonylation of alkenes with hydroxylamine electrophiles to provide α-chiral amides, β-chiral amides, and γ-chiral amides.^24^ Given the rapid advancements in copper-catalyzed enantioselective hydrofunctionalization and allylic substitution reactions in recent years,^25^ as well as our ongoing interest in carbonylation reactions, we hypothesized that a similar approach could be employed to construct enantioenriched α,β-unsaturated ketones using a suitable allylic agent. Herein, we report a copper-catalyzed carbonylative hydroallylation of vinylarenes with allylic electrophiles, using carbon monoxide (CO) as the carbonyl source under mild conditions. This approach provides a highly efficient strategy for the synthesis of valuable chiral α,β-unsaturated ketones with good functional group tolerance (Fig. 1c).

## Results and discussion

In the initial phase of our research, we studied the reaction of vinylarene (1k) with 2-methylallyl diphenyl phosphate (2k) in THF at 50 °C under 10 bar CO, using (MeO)_2_MeSiH as the hydrogen source. Xantphos was used as the ligand, and various bases were screened. The results showed that LiOMe, NaO^*t*^Bu, and CsF produced trace amounts of the desired product 3k (Table 1, entries 1, 3 and 4). However, using LiO^*t*^Bu as the base improved the yield slightly (Table 1, entry 2). Subsequently, with LiO^*t*^Bu showing the most promise, we evaluated different ligands. BINAP, (*R*)-Segphos, and (*R*)-DTBM-Segphos exhibited similar low reactivity to Xantphos, yielding very low yields of the product (entries 5–7). The use of (*S*,*S*)-Ph-BPE as the ligand significantly improved the yield to 44% with 84 : 16 enantiomeric ratio (entry 8). The examination of the solvent effects revealed that 1,2-dichloroethane (DCE) was the optimal choice, affording the product in 89% yield and high enantioselectivity (entry 9). Despite this, DCE led to the formation of side product 3k′ (9% yield), which posed a considerable challenge to the purification and isolation. This problem was resolved by employing a mixed solvent system of DCE and 1,4-dioxane, which yielded a single product 3k in 84% isolated yield with 92 : 8 enantiomeric ratio (Table 1, entry 12).

**Table 1:** Optimization of the reaction conditions^a^

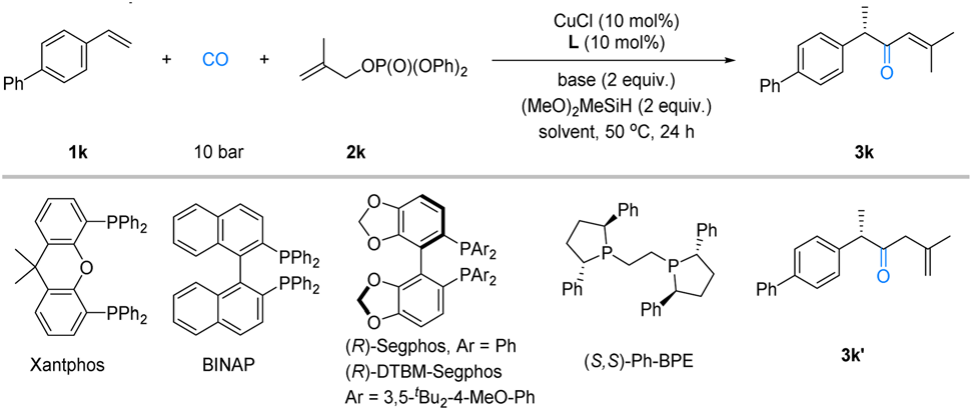					
Entry	Ligand	Base	Solvent	Yield (%)	er
1	Xantphos	LiOMe	THF	Trace	—
2	Xantphos	LiO^*t*^Bu	THF	<10	—
3	Xantphos	NaO^*t*^Bu	THF	Trace	—
4	Xantphos	CsF	THF	0	—
5	BINAP	LiO^*t*^Bu	THF	<10	—
6	(*R*)-Segphos	LiO^*t*^Bu	THF	<10	—
7	(*R*)-DTBM-Segphos	LiO^*t*^Bu	THF	13	—
8	(*S*,*S*)-Ph-BPE	LiO^*t*^Bu	THF	44	84 : 16
9	(*S*,*S*)-Ph-BPE	LiO^*t*^Bu	PhMe	54	89 : 11
10	(*S*,*S*)-Ph-BPE	LiO^*t*^Bu	Dioxane	75	92 : 8
11	(*S*,*S*)-Ph-BPE	LiO^*t*^Bu	DCE	89^c^	93 : 7
12	(*S*,*S*)-Ph-BPE	LiO^*t*^Bu	Dioxane : DCE = 1 : 1	88 (84)^b^	92 : 8

a Reaction conditions: 1a (0.1 mmol), 1b (0.2 mmol), CuCl (10 mol%), L (10 mol%), base (2.0 equiv.), (MeO)_2_MeSiH (2.0 equiv.) and CO (10 bar) in solvent (1 mL) at 50 °C for 24 h. Yields are determined by GC-FID with *n*-hexadecane as the internal standard, enantiomeric ratios were determined by chiral HPLC.

b Isolated yield.

c Mixed with 9% yield of 3k′.

**Fig. 2:**
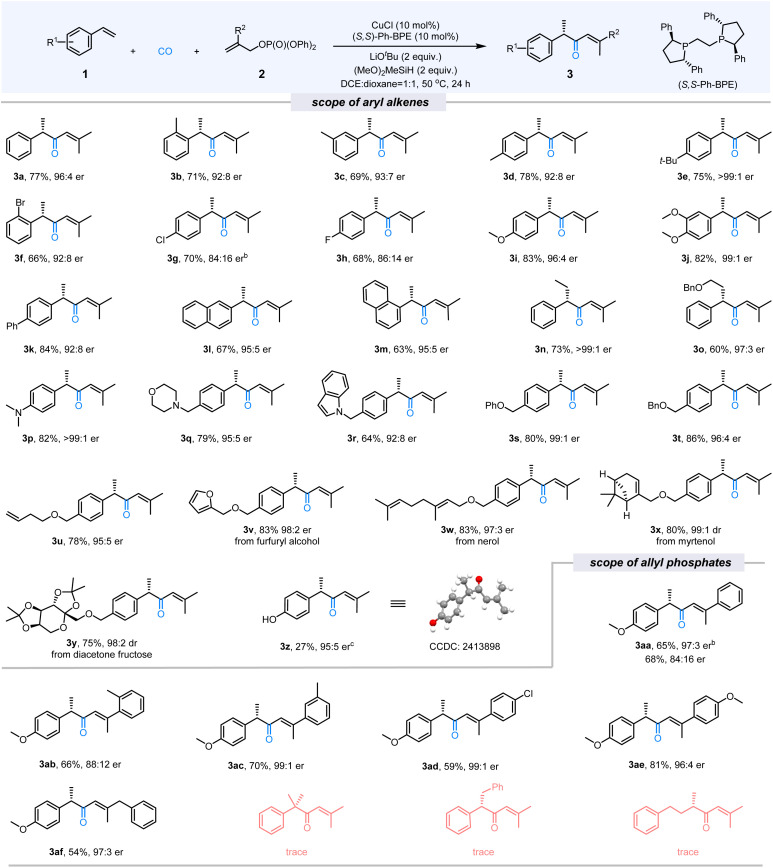
Substrate scope.^*a a*^Reaction conditions: 1 (0.1 mmol), 2 (0.2 mmol), CuCl (10 mol%), (*S*,*S*)-Ph-BPE (10 mol%), LiO^*t*^Bu (2.0 equiv.), (MeO)_2_MeSiH (2 equiv.) and CO (10 bar) in DCE (0.5 mL) and 1,4-dioxane (0.5 mL) at 50 °C for 24 h; ^*b*^30 °C for 48 h; ^*c*^The reactant of the reaction was 4-vinylphenyl acetate, and only product 3z can be detected and isolated. Enantiomeric ratios and diastereomeric ratios were determined by chiral HPLC.

After establishing the optimal conditions for the carbonylative hydroallylation reaction, an extensive investigation into the substrate scope of olefins was conducted. The results, summarized in the Fig. 2, demonstrate that a wide variety of aryl olefins can produce the corresponding α,β-unsaturated ketones with excellent yields (up to 84%) and enantiomeric ratios (up to >99 : 1 er). Specifically, variations in the position of alkyl substituents on the aromatic ring had a negligible effect on reaction outcomes, as evident in examples (3a–3d, 69–78% yield, 92 : 8–96 : 4 er). Substrates with electron-donating groups on the aromatic ring (3e, 3i, 3j) exhibited improvements in both yield and enantioselectivity, achieving yields of up to 83% and enantiomeric ratios of >99 : 1 er. Halogen-substituted olefins (3f–3h) afforded the desired products with slightly lower enantioselectivity, as shown by yields of 66–84% and er values of 84 : 16–96 : 4. Notably, internal aryl olefins, such as 3n and 3o, were well-tolerated, achieving enantiomeric ratios of up to >99 : 1 er. Olefins containing heteroatoms (N, O) exhibited excellent reactivity and stereoselectivity, with yields ranging from 64% to 86% and er values of up to >99 : 1 (3p–3u). Furthermore, natural product-derived olefins were explored. Substrates derived from furfuryl alcohol (3v), nerol (3w), myrtenol (3x), and diacetone fructose (3y) produced the desired products in high yields. It should be noted that when 4-vinylphenyl acetate was used as the substrate, only 3z can be detected and isolated in 27% yield with 95 : 5 er. The absolute configuration of 3z was determined to be *S* through X-ray crystallographic analysis.

Then, we investigated the scope of electrophilic reagents. Allyl phosphates with various substituents at the 2-position, including aryl, benzyl, and related groups, were compatible with the reaction. These substrates produced the desired α,β-unsaturated ketones (3aa–3af) with yields up to 81% and enantiomeric ratios of up to 99 : 1 er. α-Methylstyrene, (*E*)-stilbene, and 4-phenyl-1-butene were also subjected to the reaction under standard conditions, unfortunately, no desired ketone products can be detected.

**Fig. 3:**
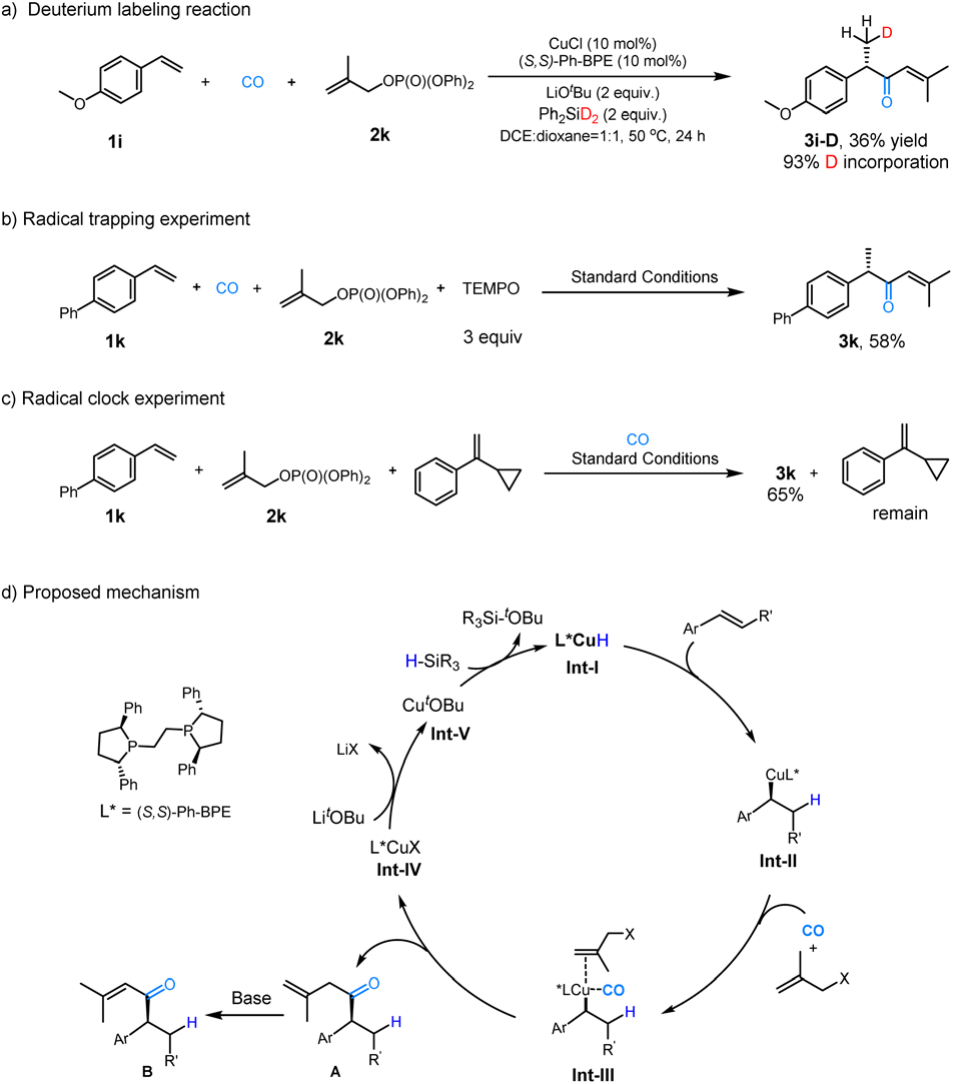
Mechanism investigation experiments and proposed mechanism. Standard conditions: CuCl (10 mol%), (*S*,*S*)-Ph-BPE (10 mol%), LiO^*t*^Bu (2.0 equiv.), (MeO)_2_MeSiH (2 equiv.) and CO (10 bar) in DCE (0.5 mL) and 1,4-dioxane (0.5 mL) at 50 °C for 24 h.

To gain deeper insight into the reaction pathway, a series of experiments was conducted. First, deuterated diphenylsilane was synthesized, and a deuterium-labelling experiment was performed under standard conditions. Using Ph_2_SiD_2_ as the hydrogen source, product 3i-D was obtained 36% yield, and 93% deuterium incorporation was observed at the β-position (Fig. 3a). To investigate whether the reaction proceeds through a radical-based mechanism, two experiments were conducted. First, TEMPO (3 equiv.) was employed as a radical trapping reagents, and the product 3k was obtained in 58% yield, indicating that the reaction was not significantly inhibited. Next, a radical clock experiment was performed using (1-cyclopropylvinyl)benzene as an additional substrate. In this case, the desired product 3k was formed in 68% yield, and (1-cyclopropylvinyl)benzene remained intact. These results indicate that no radical intermediates were involved, suggesting that the reaction likely proceeds through a non-radical pathway (Fig. 3b and c).

Based on the experimental results presented above and previous literature,^25,26^ we proposed the following plausible reaction mechanism (Fig. 3d). The cycle begins with the insertion of olefin into the key catalyst L*CuH Int-I, generating an enantiomerically enriched intermediate, Int-II. Subsequently, CO and the allyl electrophilic reagent coordinate with Int-II, leading to the formation of intermediate Int-III. Following this, CO insertion, S_N_2′-allylic substitution, and reductive elimination occur, affording product A, which undergoes isomerization to form the final α,β-unsaturated ketone (see ESI†). Finally, in the presence of a metal alkoxide and hydrosilane, Int-IV facilitates the regeneration of LCuH, completing the catalytic cycle.

**Fig. 4:**
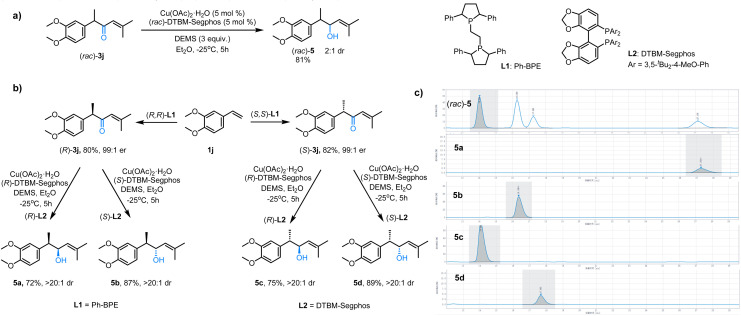
(a and b) Synthesis of all stereoisomeric chiral alcohols; (c) HPLC traces of all stereoisomeric chiral alcohols.

In biological systems, the chiral environment plays a crucial role, as drugs with different stereochemical configurations can exhibit varying therapeutic efficacies or cause adverse reactions. Despite many advances in the field of asymmetric catalytic reactions, the construction of multiple consecutive chiral centers continues to be a challenge.^27^ Therefore, we wondered whether we could identify reaction conditions suitable for the stereodivergent construction of all four stereoisomers of 5. Initially, following literature precedent,^28^ we subjected racemic 3j to reduction using the Cu(OAc)_2_/DTBM-Segphos catalytic system, obtaining racemic 5 in 81% isolated yield (Fig. 4a). Next, we prepared α,β-unsaturated ketones (*R*)-3j and (*S*)-3j by using (*R*,*R*)-L1 and (*S*,*S*)-L1 as the ligands, respectively. Notably, by subjecting (*R*)-3j and (*S*)-3j to the reaction under the specified conditions with (*R*)-L2 or (*S*)-L2-based catalysts, all four stereoisomeric chiral alcohols (5a–5d) were obtained in high yields (72–89%) with excellent enantioselectivities (>20 : 1 dr),as confirmed by the HPLC traces in Fig. 4c.

## Conclusions

In conclusion, we have developed an efficient copper-catalyzed carbonylative hydroallylation methodology for the enantioselective synthesis of α,β-unsaturated ketones. This approach utilizes mild reaction conditions, demonstrating high functional group tolerance and excellent enantioselectivity across a broad substrate scope. Moreover, the stereodivergent construction of all four stereoisomers of a chiral allylic alcohol was achieved, highlighting the method's versatility for synthesizing potential multiple contiguous stereocentres biologically relevant molecules in organic and medicinal chemistry.

## Data availability

The data supporting this article have been included as part of the ESI.†

## Author contributions

XW and YY directed the project and revised the manuscript. SS performed most of the experiments and prepared the manuscript. AS supported in substrates preparation.

## Conflicts of interest

There are no conflicts to declare.

## Supplementary Material

SC-OLF-D5SC02421H-s001

SC-OLF-D5SC02421H-s002
