# Supplementary material for: Copper-catalyzed asymmetric carbonylative hydroallylation of vinylarenes
Source: Chem Sci. 2025 May 14;16(24):10951–6. doi: 10.1039/d5sc02421h (PMC12086865; doi:10.1039/d5sc02421h)
Supplement: SC-016-D5SC02421H-s001 [file SC-016-D5SC02421H-s001.pdf]

Supplementary Information (SI) for Chemical Science.
This journal is © The Royal Society of Chemistry 2025

Supporting Information

Copper-Catalyzed Asymmetric Carbonylative Hydroallylation of Vinylarenes

Sufang Shao, Yang Yuan, Alban Schmoll, and Xiao-Feng Wu*

[*] S. Shao, C. Xu, Dr. Y. Yuan, A. Schmoll, and Prof. Dr. X.-F. Wu, Dalian National Laboratory for Clean Energy, Dalian Institute of Chemical Physics, Chinese Academy of Sciences, Dalian 116023, Liaoning, China. E-mail: yyuan@dicp.ac.cn; xwu2020@dicp.ac.cn;
S. Shao, and Prof. Dr. X.-F. Wu, Leibniz-Institut für Katalyse e.V., 18059 Rostock, Germany. E-mail: xiao-feng.wu@catalysis.de

Table of Contents

1. General information 2
2. General Procedures for Synthesis of Reactants 3
   2.1 General Procedure for the Synthesis of Allyl Alcohol 3
   2.2 General Procedure for the Synthesis of Allyl Phosphate 3
   2.3 General Procedure for the Synthesis of Olefin 4
   2.4 Characterization Data of Reactant 5
3. General Procedures for Carbonylation of Olefin with Allyl Phosphate 8
4. Mechanism investigations 9
5. Determination of the Absolute Configuration of Products 15
6. General Procedures for Enantioselective 1,2-Reductions of α,β-Unsaturated Ketones .. 17
7. Characterization Data 20
8. Copies of NMR Spectra 36
9. Copies of HPLC chromatogram 81
10. References 117

1. General information

All chemicals and reagents were obtained from Macklin, Bidepharm and Sigma-Aldrich, and were used without further purification. All solvents were dried by standard techniques and distilled prior to use. Column chromatography was performed on silica gel (200-300 meshes) using petroleum ether (bp. 60-90 °C), ethyl acetate and dichloromethane as eluent. All NMR spectra were recorded at ambient temperature using Bruker Avance III 400 MHz NMR (^{1}H, 400 MHz; ^{13}C, 100 MHz, ^{19}F 376 MHz). All ^{1}H NMR data are reported in δ units, parts per million (ppm), and were measured relative to the residual proton signal in the deuterated solvent at 7.26 ppm ($CDCl_3$) whereas ^{13}C NMR spectra are ^{1}H decoupled and reported in ppm relative to the solvent signal at 77.16 ppm ($CDCl_3$). Data for ^{1}H are reported as follows: chemical shift (δ ppm), multiplicity (s = singlet, d = doublet, t = triplet, q = quartet, quint = quintet, m = multiplet, br = broad), coupling constant (Hz), and integration. Enantioselectivities were recorded on an Agilent HPLC instrument, using a chiral stationary phase column (Daicel CHIRALPAK® AD-H, AS-H, IC, IA, OD-H, OJ-H). The chiral HPLC methods were calibrated with the corresponding racemic mixtures. All reactions were monitored by GC-FID or NMR analysis, GC-yields were calculated using hexadecane as internal standard. All measurements were carried out at room temperature unless otherwise stated.

Unless otherwise noted, all reactions were carried out under carbon monoxide (CO) or nitrogen atmosphere. Because of the high toxicity of carbon monoxide, all the reactions should be performed in an autoclave. The laboratory should be well-equipped with a CO detector and alarm system.

2. General Procedures for Synthesis of Reactants

2.1 General Procedure for the Synthesis of Allyl Alcohol

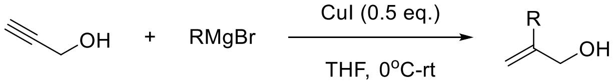

The material was prepared according to the reported literature.[1] To add CuI (10 mmol, 1.95 g) to a 250 mL two-mouth flask, and the flask was evacuated and backfilled with nitrogen for three times, then to add 30 mL THF to the flask and add Grignard reagent (RMgBr, 50 mmol, 50 mL) at 0 °C. After stirring for half an hour, propargyl alcohol (20 mmol, 2.9 mL) solution was added dropwise at 0 °C. Then the mixture was allowed to stir at rt for 20 h. The reaction was quenched carefully by aqueous saturated NH_4Cl solution in ice-water bath. The biphase system was extracted by ethyl acetate for three times (100 mL × 3) and then the combined organic phase was washed by brine for three times (100 mL × 3). The organic phase was dried over $MgSO_4$, concentrated in vacuo and the residue was purified by silica gel column chromatography to provide allyl alcohol.

2.2 General Procedure for the Synthesis of Allyl Phosphate

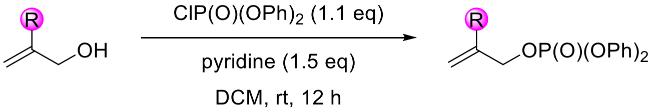

The material was prepared according to the reported literature.[2] A 50 mL round-bottom flask equipped with a stir bar was charged with allylic alcohol (1.0 equiv), CH_2Cl_2 (3 mL/mmol allylic alcohol), and pyridine (1.5 equiv). Diphenyl chlorophosphate (1.1 equiv) was added, the flask was stoppered with a vented septum, and the reaction mixture was stirred under air at room temperature for 12 h. The reaction mixture was then diluted with CH_2Cl_2 and water and transferred to a separatory funnel. The phases were partitioned, and the aqueous layer was extracted with CH_2Cl_2 (3×). The combined organic layers were dried (Na_2SO_4), filtered, and concentrated in vacuo to afford the crude material, which was purified by flash column chromatography to

provide the desired product.

2.3 General Procedure for the Synthesis of Olefin

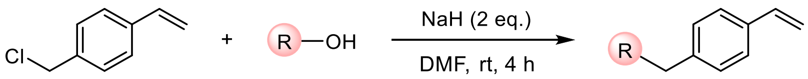

The material was prepared according to the reported literature.[3] Sodium hydride (60% in mineral oil, 800 mg, 20 mmol, 2.00 equiv) was suspended in DMF (300 mL), alcohol (25 mmol, 2.50 equiv) and 1-(chloromethyl)-4-vinylbenzene (1.4 mL, 10 mmol, 1.00 equiv) were added at 0 °C. After the mixture was further stirred for 4 h, the mixture was diluted with ethyl acetate (30 mL). Saturated aqueous ammonium chloride (15 mL) was added to quench the reaction and the aqueous layer was extracted with ethyl acetate (3 × 30 mL). The combined organic layers were dried over sodium sulfate and the solvent was removed in vacuo. Then, the residue was purified by silica gel column chromatography to product.

2.4 Characterization Data of Reactant

Diphenyl (2-phenylallyl) phosphate (A1)

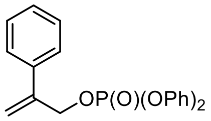

^{1}H NMR (400 MHz, $CDCl_3$) δ 7.41 – 7.36 (m, 2H), 7.31 – 7.25 (m, 7H), 7.16 (d, *J* = 8.0 Hz, 6H), 5.54 (s, 1H), 5.41 (s, 1H), 5.11 (d, *J* = 7.6 Hz, 2H).

Diphenyl (2-(o-tolyl)allyl) phosphate (A2)

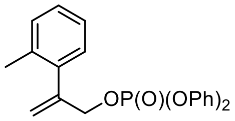

^{1}H NMR (400 MHz, $CDCl_3$) δ 7.29 (dd, *J* = 8.6, 7.2 Hz, 4H), 7.23 – 7.07 (m, 10H), 5.55 (d, *J* = 1.5 Hz, 1H), 5.13 (d, *J* = 1.3 Hz, 1H), 4.89 (dt, *J* = 7.1, 1.4 Hz, 2H), 2.26 (s, 3H).

Diphenyl (2-(m-tolyl)allyl) phosphate (A3)

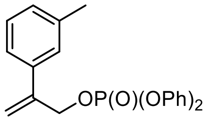

^{1}H NMR (400 MHz, $CDCl_3$) δ 7.28 (dd, *J* = 8.7, 7.1 Hz, 4H), 7.23 – 7.08 (m, 10H), 5.53 (s, 1H), 5.39 (d, *J* = 1.3 Hz, 1H), 5.10 (dd, *J* = 7.6, 1.2 Hz, 2H), 2.31 (s, 3H).

2-(4-Chlorophenyl)allyl diphenyl phosphate (A4)

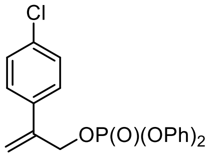

^{1}H NMR (400 MHz, $CDCl_3$) δ 7.33 – 7.24 (m, 8H), 7.23 – 7.10 (m, 6H), 5.54 (s, 1H), 5.43 (s, 1H), 5.07 (d, *J* = 7.8 Hz, 2H).

2-(4-Methoxyphenyl)allyl diphenyl phosphate (A5)

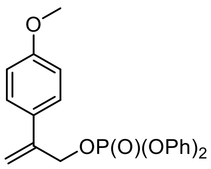

^{1}H NMR (400 MHz, $CDCl_3$) δ 7.37 – 7.24 (m, 7H), 7.18 – 7.14 (m, 5H), 6.84 (d, *J* = 8.9 Hz, 2H), 6.75 (s, 1H), 5.47 (s, 1H), 5.32 (d, *J* = 1.1 Hz, 1H), 5.09 (dd, *J* = 7.6, 1.1 Hz, 2H), 3.81 (s, 3H).

(*E*)-1-(((3,7-Dimethylocta-2,6-dien-1-yl)oxy)methyl)-4-vinylbenzene (A6)

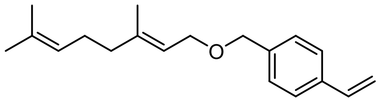

^{1}H NMR (400 MHz, CDCl$_3$) δ 7.37 (d, *J* = 8.2 Hz, 2H), 7.29 (d, *J* = 8.1 Hz, 2H), 6.69 (dd, *J* = 17.6, 10.9 Hz, 1H), 5.72 (d, *J* = 18.5 Hz, 1H), 5.40 (ddt, *J* = 6.7, 5.4, 1.3 Hz, 1H), 5.21 (dd, *J* = 10.9, 0.9 Hz, 1H), 5.10 (ddt, *J* = 8.4, 5.1, 1.5 Hz, 1H), 4.47 (s, 2H), 4.01 (d, *J* = 6.8 Hz, 2H), 2.16 – 1.99 (m, 4H), 1.70 – 1.57 (m, 9H).

2-(((4-Vinylbenzyl)oxy)methyl)furan (A7)

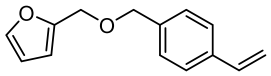

^{1}H NMR (400 MHz, CDCl$_3$) δ 7.41 – 7.39 (m, 1H), 7.39 – 7.25 (m, 4H), 6.69 (dd, *J* = 17.6, 10.9 Hz, 1H), 6.31 (dd, *J* = 7.9, 2.6 Hz, 2H), 5.73 (d, *J* = 17.6 Hz, 1H), 5.22 (d, *J* = 10.6 Hz, 1H), 4.51 (s, 2H), 4.46 (s, 2H).

1-((But-3-en-1-yloxy)methyl)-4-vinylbenzene (A8)

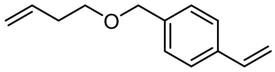

^{1}H NMR (400 MHz, CDCl$_3$) δ 7.45 – 7.25 (m, 4H), 6.70 (dd, *J* = 17.6, 10.9 Hz, 1H), 5.84 (ddt, *J* = 17.0, 10.2, 6.7 Hz, 1H), 5.73 (dd, *J* = 17.6, 1.0 Hz, 1H), 5.22 (d, *J* = 10.9 Hz, 1H), 5.15 – 5.00 (m, 2H), 4.50 (s, 2H), 3.51 (t, *J* = 6.7 Hz, 2H), 2.37 (q, *J* = 6.7 Hz, 2H).

1-((Benzyloxy)methyl)-4-vinylbenzene (A9)

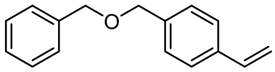

^{1}H NMR (400 MHz, CDCl$_3$) δ 7.43 – 7.26 (m, 9H), 6.72 (dd, *J* = 17.6, 10.9 Hz, 1H), 5.74 (dd, *J* = 17.6, 0.9 Hz, 1H), 5.24 (dd, *J* = 10.9, 0.9 Hz, 1H), 4.55 (d, *J* = 1.6 Hz, 4H).

(1*R*,5*S*)-6,6-Dimethyl-2-(((4-vinylbenzyl)oxy)methyl)bicyclo[3.1.1]hept-2-ene (A10)

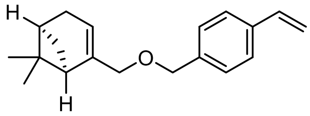

^{1}H NMR (400 MHz, CDCl$_3$) δ 7.42 – 7.24 (m, 4H), 6.70 (dd, *J* = 17.6, 10.9 Hz, 1H), 5.73 (dd, *J* = 17.6, 0.9 Hz, 1H), 5.51 (tt, *J* = 3.0, 1.5 Hz, 1H), 5.22 (dd, *J* = 10.9, 0.9 Hz,

1H), 4.45 (s, 2H), 3.88 (q, *J* = 1.6 Hz, 2H), 2.41 (dt, *J* = 8.6, 5.6 Hz, 1H), 2.37 – 2.25 (m, 2H), 2.21 (td, *J* = 5.7, 1.6 Hz, 1H), 2.11 (dddt, *J* = 5.9, 4.3, 3.0, 1.3 Hz, 1H), 1.29 (s, 3H), 1.20 (d, *J* = 8.6 Hz, 1H), 0.86 (s, 3H).

(3a*S*,5a*R*,8a*R*,8b*S*)-2,2,7,7-Tetramethyl-3a-(((4-vinylbenzyl)oxy)methyl)tetrahydro-5H-bis([1,3]dioxolo)[4,5-b:4',5'-d]pyran (A11)

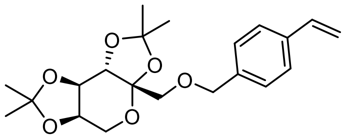

^{1}H NMR (400 MHz, CDCl$_3$) δ 7.42 – 7.27 (m, 4H), 6.69 (ddd, *J* = 17.6, 10.9, 1.4 Hz, 1H), 5.72 (dd, *J* = 17.6, 1.3 Hz, 1H), 5.21 (dd, *J* = 10.9, 1.5 Hz, 1H), 4.69 – 4.52 (m, 3H), 4.43 (d, *J* = 2.6 Hz, 1H), 4.24 – 4.18 (m, 1H), 3.91 (dt, *J* = 12.9, 1.6 Hz, 1H), 3.72 (dd, *J* = 13.1, 1.4 Hz, 1H), 3.65 – 3.55 (m, 2H), 1.54 (s, 3H), 1.41 (d, *J* = 4.2 Hz, 6H), 1.32 (s, 3H).

Diphenyl (2-phenylallyl) phosphate (A12)

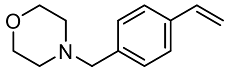

^{1}H NMR (400 MHz, CDCl$_3$) δ 7.40 – 7.25 (m, 4H), 6.70 (dd, *J* = 17.6, 10.9 Hz, 1H), 5.73 (dd, *J* = 17.6, 0.9 Hz, 1H), 5.22 (dd, *J* = 10.9, 0.9 Hz, 1H), 3.75 – 3.64 (m, 4H), 3.47 (s, 2H), 2.48 – 2.37 (m, 4H).

Diphenyl (2-phenylallyl) phosphate (A13)

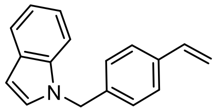

^{1}H NMR (400 MHz, CDCl$_3$) δ 7.42 – 7.24 (m, 4H), 6.70 (dd, *J* = 17.6, 10.9 Hz, 1H), 5.73 (dd, *J* = 17.6, 0.9 Hz, 1H), 5.51 (tt, *J* = 3.0, 1.5 Hz, 1H), 5.22 (dd, *J* = 10.9, 0.9 Hz, 1H), 4.45 (s, 2H), 3.88 (q, *J* = 1.6 Hz, 2H), 2.41 (dt, *J* = 8.6, 5.6 Hz, 1H), 2.37 – 2.25 (m, 2H), 2.21 (td, *J* = 5.7, 1.6 Hz, 1H), 2.11 (dddt, *J* = 5.9, 4.3, 3.0, 1.3 Hz, 1H), 1.29 (s, 3H), 1.20 (d, *J* = 8.6 Hz, 1H), 0.86 (s, 3H).

3. General Procedures for Carbonylation of Olefin with Allyl Phosphate

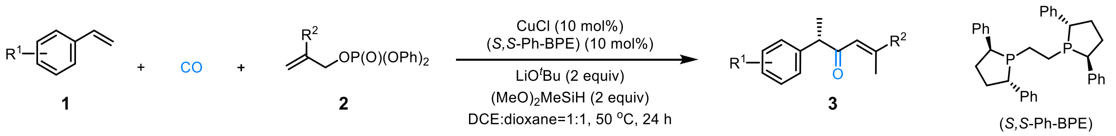

General Procedure A A screw-cap vial (4 mL) was loaded with CuCl (1.0 mg, 10 mol%), (*S,S*)-Ph-BPE (5.1 mg, 10 mol%), LiO^{t}Bu (16.0 mg, 0.2 mmol) and a stir bar. First, the vial was sealed with a Teflon septum and cap and connected to the atmosphere via a needle. Then, under a nitrogen atmosphere, DCE (0.5 mL), dioxane (0.5 mL), **1** (0.1 mmol), **2** (2.0 equiv., 0.2 mmol) and $(MeO)_2MeSiH$ (2.0 equiv., 0.2 mmol) were added using a syringe. Next, the vial was transferred to an alloy plate and placed into a Parr 4560 series autoclave (300 mL) under an argon atmosphere. After that, at room temperature, the autoclave was purged with CO three times and then charged with 10 atm of CO. Subsequently, the autoclave was placed on a heating plate equipped with a magnetic stirrer and an aluminum block. The reaction mixture was heated to 50 °C and maintained at this temperature for 24 h. Once the reaction was complete, the autoclave was cooled to room temperature, and the pressure was carefully released. Finally, the reaction mixture was directly purified by column chromatography on silica gel, with petroleum ether and ethyl acetate being used to obtain the corresponding product **3**.

4. Mechanism investigations

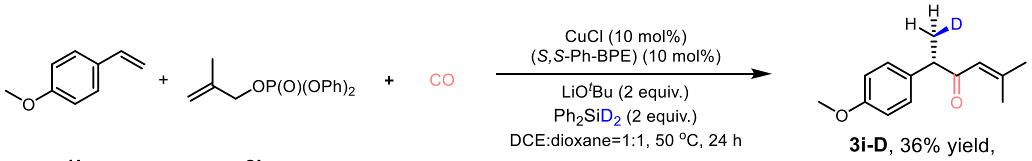

A screw-cap vial (4 mL) was loaded with CuCl (1.0 mg, 10 mol%), (*S,S*)-Ph-BPE (5.1 mg, 10 mol%), LiO^{t}Bu (16.0 mg, 0.2 mmol) and a stir bar. First, the vial was sealed with a Teflon septum and cap and connected to the atmosphere via a needle. Then, under a nitrogen atmosphere, DCE (0.5 mL), dioxane (0.5 mL), **1i** (0.1 mmol), **2k** (2.0 equiv., 0.2 mmol) and Ph_2SiD_2 (2.0 equiv., 0.2 mmol) were added using a syringe. Next, the vial was transferred to an alloy plate and placed into a Parr 4560 series autoclave (300 mL) under an argon atmosphere. After that, at room temperature, the autoclave was purged with CO three times and then charged with 10 atm of CO. Subsequently, the autoclave was placed on a heating plate equipped with a magnetic stirrer and an aluminum block. The reaction mixture was heated to 50 °C and maintained at this temperature for 24 h. Once the reaction was complete, the autoclave was cooled to room temperature, and the pressure was carefully released. Finally, the reaction mixture was directly purified by column chromatography on silica gel, with petroleum ether and ethyl acetate being used to obtain the corresponding product **3i-D** (7.9 mg, 36% yield, 93% D) as a colorless oil. **^{1}H NMR** (400 MHz, Chloroform-*d*) δ 7.13 (d, *J* = 8.7 Hz, 2H), 6.86 (d, *J* = 8.7 Hz, 2H), 5.99 (p, *J* = 1.3 Hz, 1H), 3.79 (s, 3H), 3.67 (t, *J* = 6.9 Hz, 1H), 2.13 (s, 3H), 1.79 (s, 3H), 1.35 (dt, *J* = 7.1, 1.8 Hz, 2H).

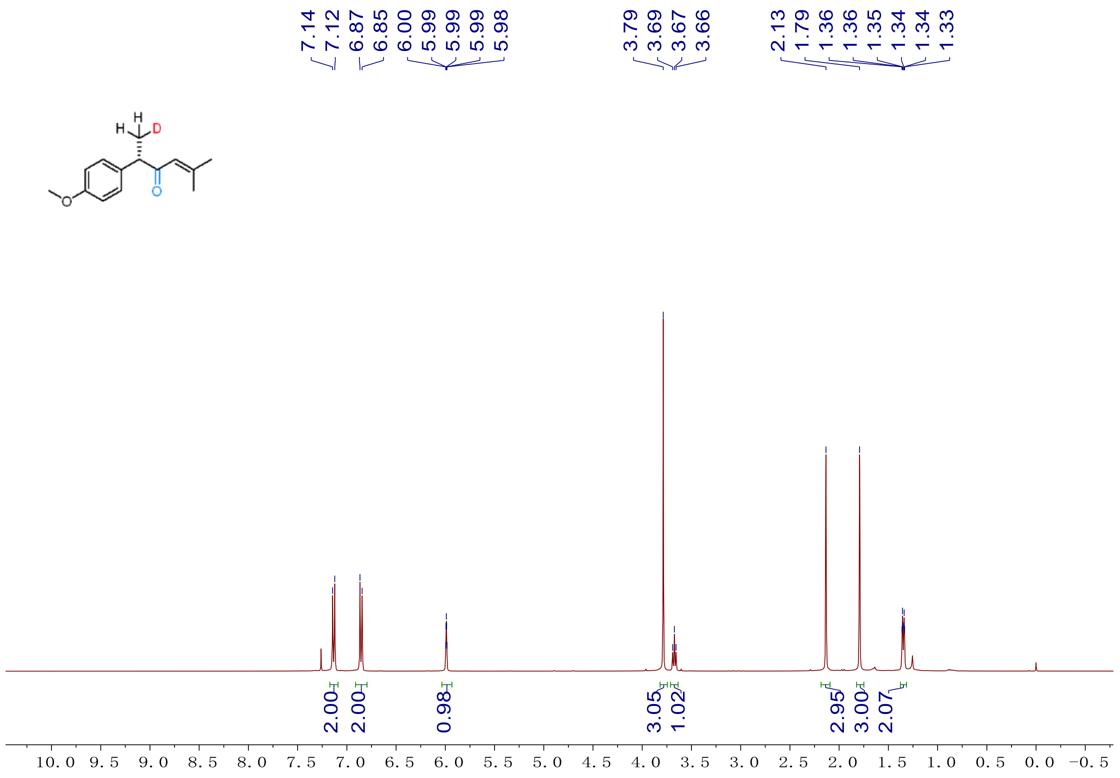

^{1}H NMR spectra of **3i-D** in $CDCl_3$ (400 MHz)

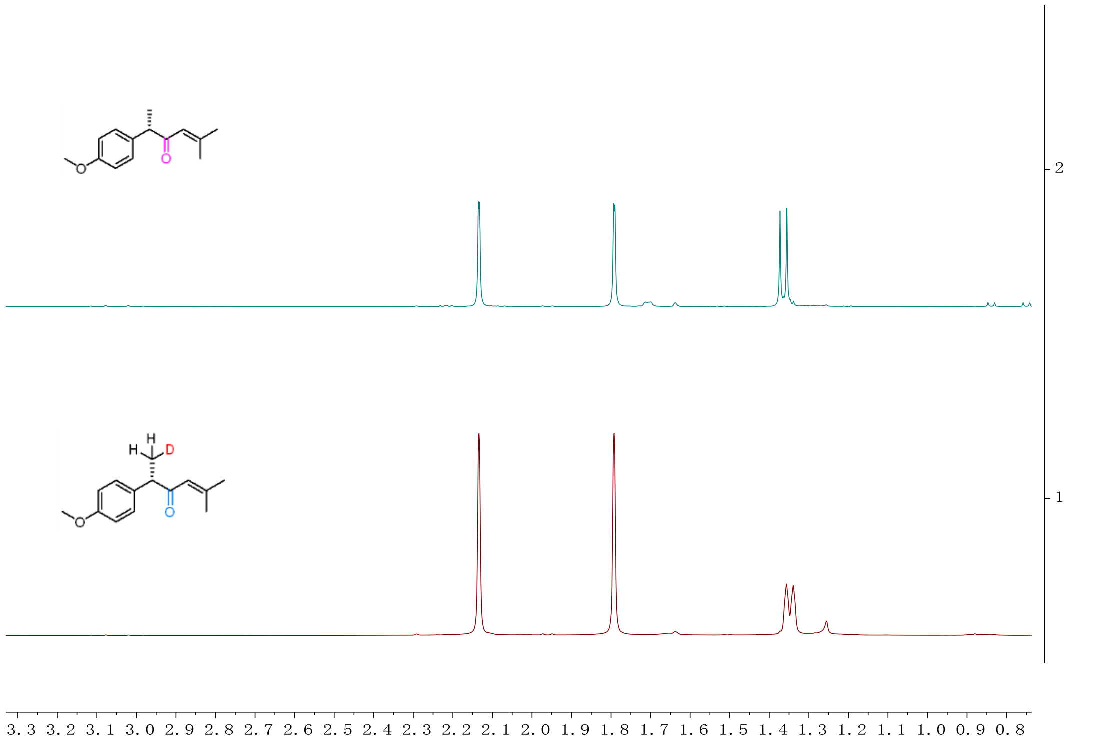

^{1}H NMR spectra of **3i** and **3i-D** in $CDCl_3$ (400 MHz)

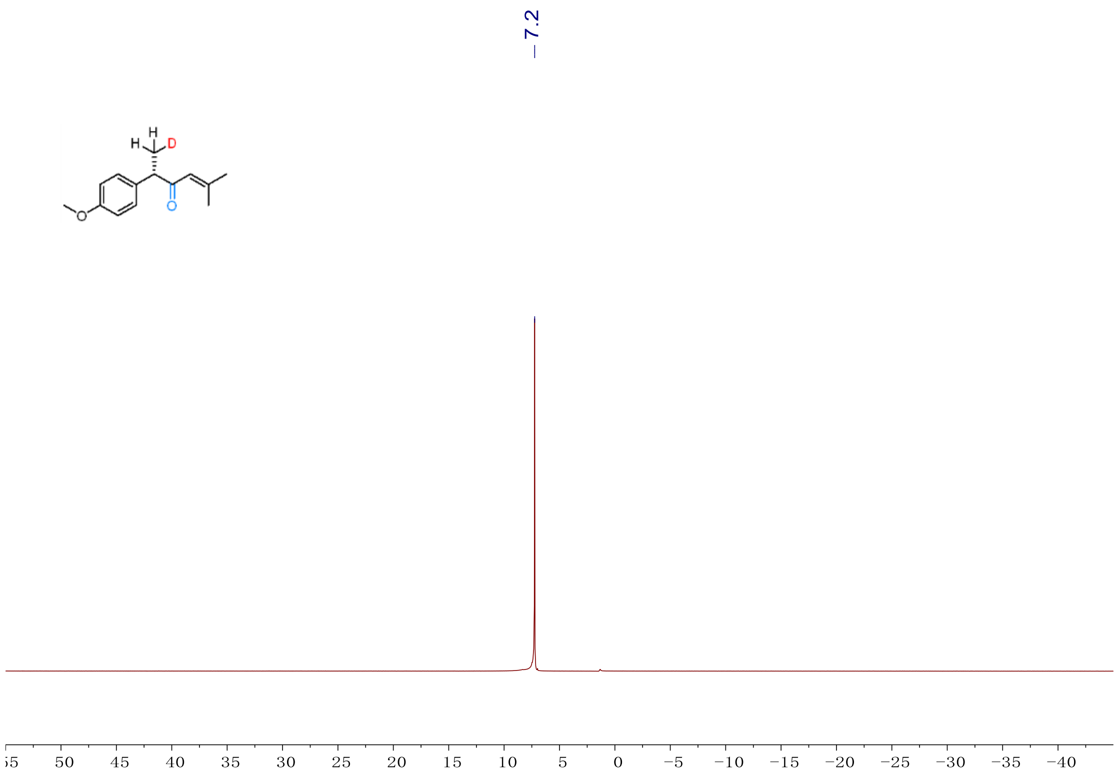

^{2}H NMR spectra of **3i-D** in $CDCl_3$ (400 MHz)

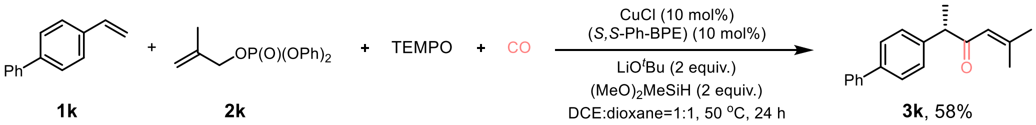

A screw-cap vial (4 mL) was loaded with CuCl (1.0 mg, 10 mol%), (*S,S*)-Ph-BPE (5.1 mg, 10 mol%), LiO^{t}Bu (16.0 mg, 0.2 mmol) and a stir bar. First, the vial was sealed with a Teflon septum and cap and connected to the atmosphere via a needle. Then, under a nitrogen atmosphere, DCE (0.5 mL), dioxane (0.5 mL), **1k** (0.1 mmol), **2k** (2.0 equiv., 0.2 mmol), TEMPO (3 equiv., 0.3 mmol) and $(MeO)_2MeSiH$ (2.0 equiv., 0.2 mmol) were added using a syringe. Next, the vial was transferred to an alloy plate and placed into a Parr 4560 series autoclave (300 mL) under an argon atmosphere. After that, at room temperature, the autoclave was purged with CO three times and then charged with 10 atm of CO. Subsequently, the autoclave was placed on a heating plate equipped with a magnetic stirrer and an aluminum block. The reaction mixture was heated to 50 °C and maintained at this temperature for 24 h. Once the reaction was complete, the autoclave was cooled to room temperature, and the pressure was carefully released. Finally, the reaction mixture was directly purified by column chromatography on silica gel, with petroleum ether and ethyl acetate being used to obtain the corresponding

product **3k** (15.3 mg, 58% yield) as a colorless oil.

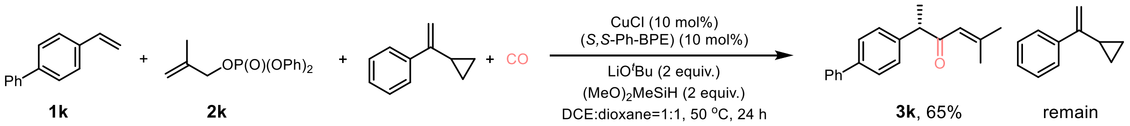

A screw-cap vial (4 mL) was loaded with CuCl (1.0 mg, 10 mol%), (*S,S*)-Ph-BPE (5.1 mg, 10 mol%), LiO^{t}Bu (16.0 mg, 0.2 mmol) and a stir bar. First, the vial was sealed with a Teflon septum and cap and connected to the atmosphere via a needle. Then, under a nitrogen atmosphere, DCE (0.5 mL), dioxane (0.5 mL), **1k** (0.1 mmol), **2k** (2.0 equiv., 0.2 mmol), (1-cyclopropylvinyl)benzene (3.0 equiv., 0.3 mmol) and (MeO)$_2$MeSiH (2.0 equiv., 0.2 mmol) were added using a syringe. Next, the vial was transferred to an alloy plate and placed into a Parr 4560 series autoclave (300 mL) under an argon atmosphere. After that, at room temperature, the autoclave was purged with CO three times and then charged with 10 atm of CO. Subsequently, the autoclave was placed on a heating plate equipped with a magnetic stirrer and an aluminum block. The reaction mixture was heated to 50 °C and maintained at this temperature for 24 h. Once the reaction was complete, the autoclave was cooled to room temperature, and the pressure was carefully released. Finally, the reaction mixture was directly purified by column chromatography on silica gel, with petroleum ether and ethyl acetate being used to obtain the corresponding product **3k** (17.2 mg, 65% yield) as a colorless oil.

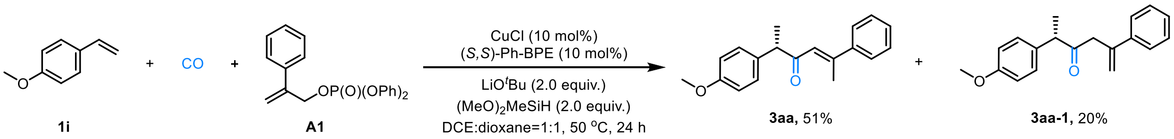

A screw-cap vial (4 mL) was loaded with CuCl (1.0 mg, 10 mol%), (*S,S*)-Ph-BPE (5.1 mg, 10 mol%), LiO^{t}Bu (16.0 mg, 0.2 mmol) and a stir bar. First, the vial was sealed with a Teflon septum and cap and connected to the atmosphere via a needle. Then, under a nitrogen atmosphere, DCE (0.5 mL), dioxane (0.5 mL), **1i** (0.1 mmol), **A1** (2.0 equiv., 0.2 mmol) and (MeO)$_2$MeSiH (2.0 equiv., 0.2 mmol) were added using a syringe. Next, the vial was transferred to an alloy plate and placed into a Parr 4560 series autoclave (300 mL) under an argon atmosphere. After that, at room temperature, the autoclave

was purged with CO three times and then charged with 10 atm of CO. Subsequently, the autoclave was placed on a heating plate equipped with a magnetic stirrer and an aluminum block. The reaction mixture was heated to 50 °C and maintained at this temperature for 24 h. Once the reaction was complete, the autoclave was cooled to room temperature, and the pressure was carefully released. Finally, the reaction mixture was directly purified by column chromatography on silica gel, with petroleum ether and ethyl acetate being used to obtain the corresponding product **3aa** (14.3 mg, 51% yield) and **3aa-1** (5.6 mg, 20% yield) as a colorless oil. **^{1}H NMR** of **3aa-1** (400 MHz, Chloroform-*d*) δ 7.28 – 7.20 (m, 5H), 7.09 (d, *J* = 8.7 Hz, 2H), 6.85 (d, *J* = 8.7 Hz, 2H), 5.52 (s, 1H), 5.05 (s, 1H), 3.80 (s, 4H), 3.60 – 3.46 (m, 2H), 1.30 (d, *J* = 6.9 Hz, 3H); **^{13}C NMR** of **3aa-1** (101 MHz, Chloroform-*d*) δ 208.4, 158.8, 141.5, 139.9, 132.4, 129.1, 128.3, 127.7, 125.8, 116.6, 114.3, 55.3, 51.1, 47.8, 17.7.

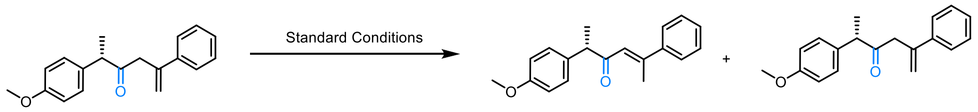

3aa-1 **3aa**, 63% **3aa-1**, 28%

Following **General Procedure A**, **3aa-1** was used as the reactant and was only partially converted to **3aa** (17.6 mg, 63% yield).

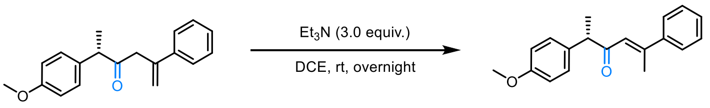

3aa-1 **3aa**, quant.

3aa-1 (28 mg, 0.1 mmol) was suspended in DCE (2 mL). Then, Et_3N (3.0 equiv, 0.3 mmol) was added, and the resulting mixture was allowed to react overnight. Subsequently, the reaction mixture was directly purified by column chromatography on silica gel to obtain the corresponding product **3aa** (28 mg, >99%). According to the reported literature,[4] the α,β-unsaturated ketone could be fully converted from the β,γ-unsaturated ketone in the presence of triethylamine. Therefore, we added triethylamine after the reaction was completed to perform the normalization of the mixed products.

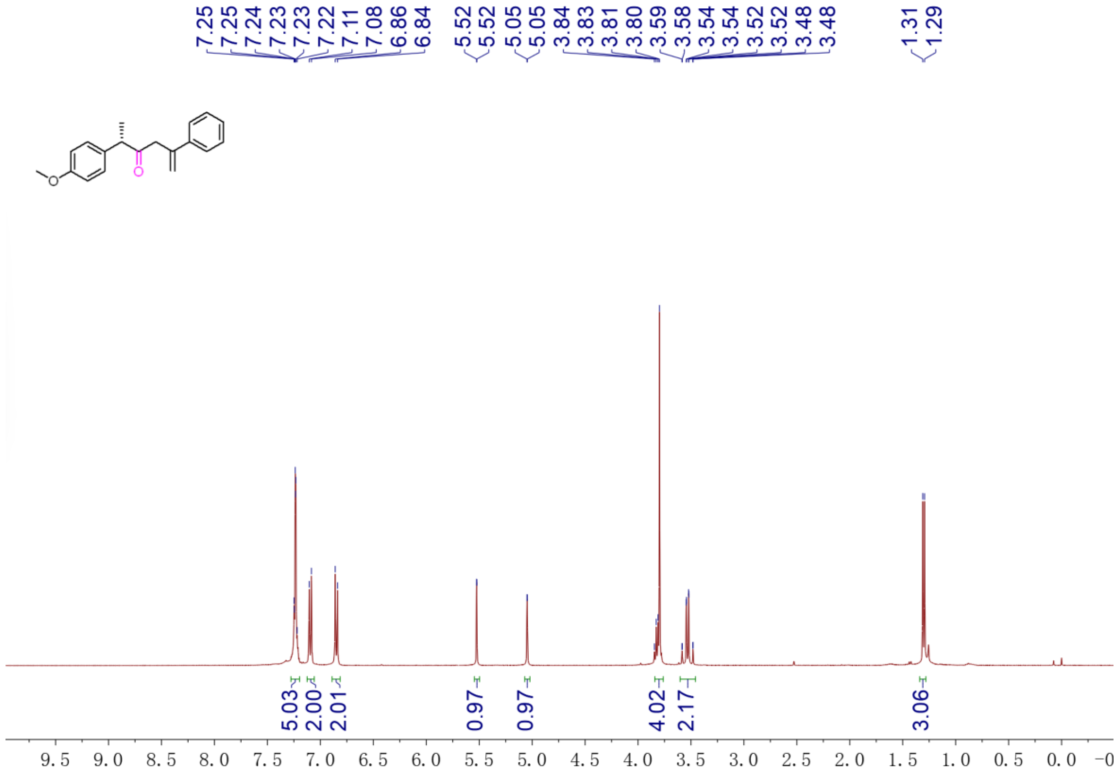

^{1}H NMR spectra of **3aa-1** in $CDCl_3$ (400 MHz)

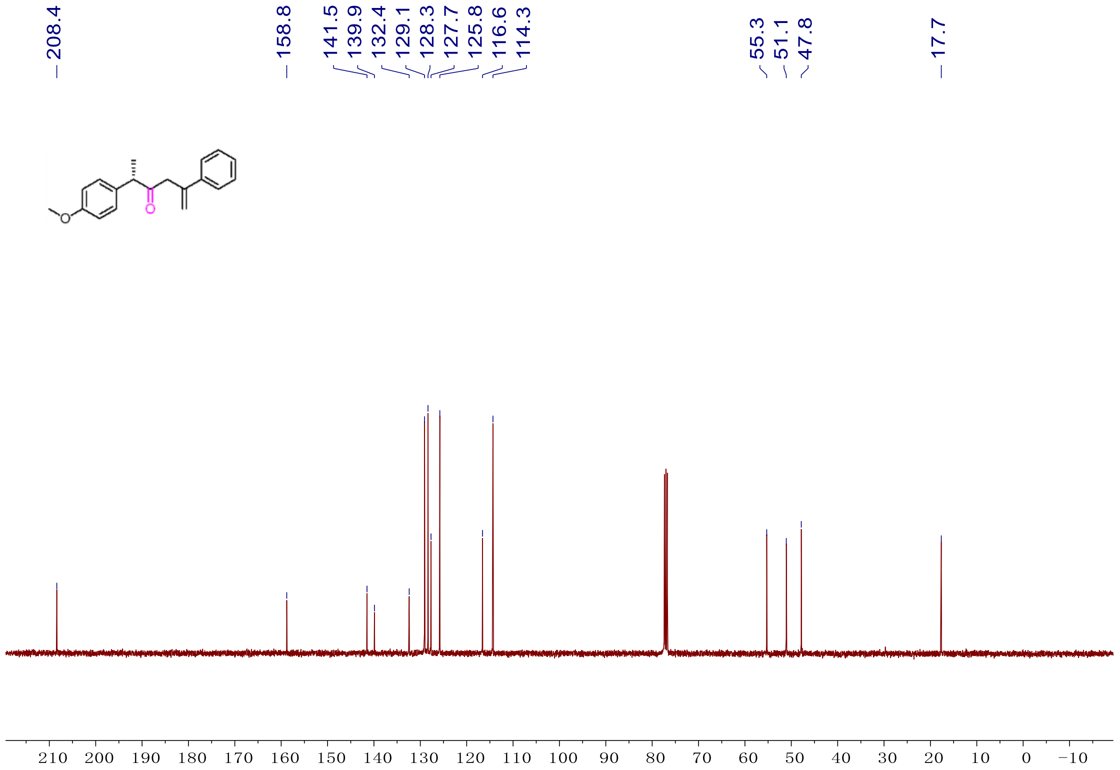

^{13}C NMR spectra of **3aa-1** in $CDCl_3$ (100 MHz)

5. Determination of the Absolute Configuration of Products

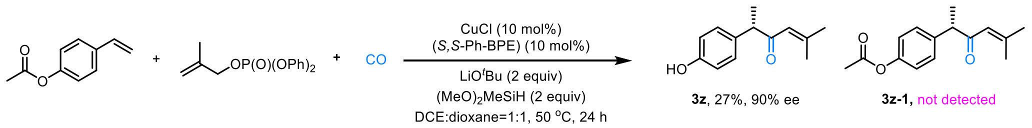

The single crystals of **3z** were obtained by slow evaporation of the mixed solvent of DCM and petroleum ether at rt. CCDC number of **3z** is 2413898. The data can be obtained free of charge from the Cambridge Crystallographic Data Centre-via www.fcdc.cam.ac.uk. **3z** was determined to be (*S*) by X-ray diffraction The stereochemistries of the remaining α,β-unsaturated ketones were assigned by analogy.

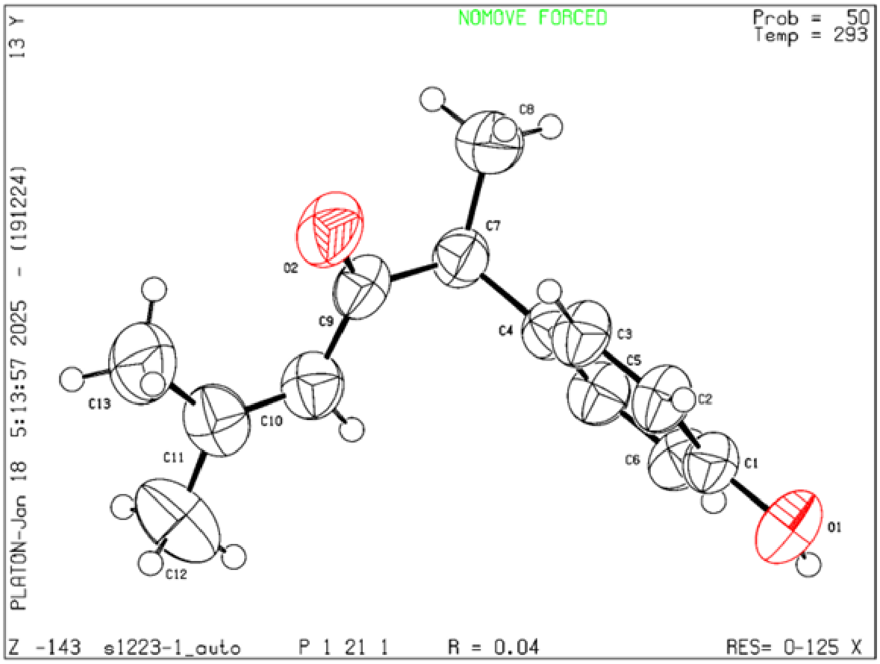

Figure S1 Crystallographic date of compound **3z** (CCDC number 2413898).

Datablock: s1223-1_auto

Bond precision:	C-C = 0.0052 A		Wavelength=1.54184
Cell:	a=8.9792(10)	b=6.2593(6)	c=11.4802(13)
	alpha=90	beta=111.827(13)	gamma=90
Temperature:	293 K		

	Calculated	Reported
Volume	598.97(12)	598.97(12)
Space group	P 21	P 1 21 1
Hall group	P 2yb	P 2yb
Moiety formula	C13 H16 O2	C13 H16 O2
Sum formula	C13 H16 O2	C13 H16 O2
Mr	204.26	204.26
Dx,g cm-3	1.133	1.133
Z	2	2
Mu (mm-1)	0.597	0.597
F000	220.0	220.0
F000'	220.64	
h,k,lmax	10,7,13	10,7,13
Nref	2134[1173]	1697
Tmin,Tmax	0.994,0.994	0.907,1.000
Tmin'	0.994	

Correction method= # Reported T Limits: Tmin=0.907 Tmax=1.000
AbsCorr = MULTI-SCAN

Data completeness= 1.45/0.80 Theta(max)= 66.760

R(reflections)= 0.0429(1336) wR2(reflections)= 0.1383(1697)

S = 0.928 Npar= 139

6. General Procedures for Enantioselective 1,2-Reductions of α,β-Unsaturated Ketones

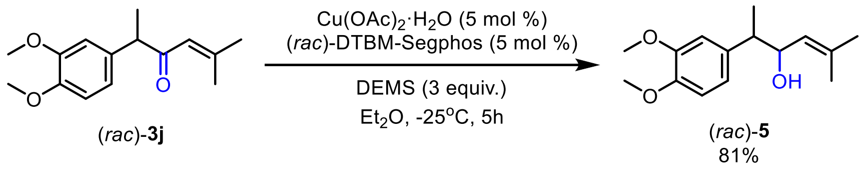

General Procedure B A screw-cap vial (4 mL) was loaded with fine powdered $Cu(OAc)_2 \cdot H_2O$ (0.9 mg, 5 mol %, 5 μmol) and (*rac*)-DTBM-Segphos (5.9 mg, 5 mol %, 5 μmol). The vial was capped with a rubber septum and placed under an Argon atmosphere, 0.4 mL Et_2O was added via syringe. At rt, DEMS (48 μL, 0.3 mmol) was introduced, resulting in a brown solution after 10 min. The vial was then placed into a pre-cooled acetone bath at -25°C and stirred for an additional 5 min. (*rac*)**-3j** (0.1 mmol) were subsequently introduced via syringe. The side of the reaction vial was rinsed with Et_2O (2 × 25 μL). After TLC confirmed full conversion, the reaction was quenched at -25°C by the addition of 0.5 mL sat. NH_4F/MeOH. The reaction vial was taken out of the cooling bath and warmed to rt. After filtration through SiO_2, the solvent was evaporated in vacuo and the crude reaction mixture purified by column chromatography on silica gel to obtain the corresponding product (*rac*)**-5** (20.3 mg, 81% yield).

(*2R,3R*)-2-(3,4-dimethoxyphenyl)-5-methylhex-4-en-3-ol-(5a)

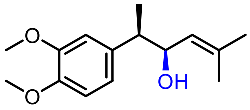

It was prepared following **General Procedure B**, using (*R*)-DTBM-Segphos as the ligand, **(*R*)-3j** as the reactant. Finally, the reaction mixture was directly purified by column chromatography on silica gel, with petroleum ether and ethyl acetate being used to obtain the corresponding product **5a**.

18.0 mg, 72% yield, >20:1 dr, colourless oil. Eluent: pentane/ethyl acetate = 10:1.

^{1}H NMR (400 MHz, $CDCl_3$) δ 6.87 – 6.76 (m, 3H), 5.19 (dt, *J* = 8.9, 1.4 Hz, 1H), 4.33 (t, *J* = 8.6 Hz, 1H), 3.88 (d, *J* = 15.8 Hz, 6H), 2.72 – 2.64 (m, 1H), 1.77 (d, *J* = 1.5 Hz, 3H), 1.70 (d, *J* = 1.5 Hz, 3H), 1.18 (d, *J* = 7.1 Hz, 3H).

^{13}C NMR (100 MHz, CDCl$_3$) δ 149.0, 147.8, 136.5, 136.0, 126.0, 119.9, 111.3, 111.3, 73.2, 55.9, 55.9, 46.6, 26.0, 18.6, 17.9.

HPLC analysis (OJ-H column, 80:20 hexanes/2-propanol, 0.6 mL/min, t = 27.3 min)

(*2R,3S*)-2-(3,4-dimethoxyphenyl)-5-methylhex-4-en-3-ol-(5b)

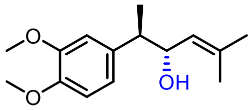

It was prepared following **General Procedure B**, using (*S*)-DTBM-Segphos as the ligand, **(*R*)-3j** as the reactant. Finally, the reaction mixture was directly purified by column chromatography on silica gel, with petroleum ether and ethyl acetate being used to obtain the corresponding product **5b**.

21.8 mg, 87% yield, >20:1 dr, colourless oil. Eluent: pentane/ethyl acetate = 10:1.

^{1}H NMR (400 MHz, CDCl$_3$) δ 6.86 – 6.70 (m, 3H), 5.10 (dq, *J* = 8.7, 1.3 Hz, 1H), 4.39 (dd, *J* = 8.8, 5.9 Hz, 1H), 3.87 (d, *J* = 4.1 Hz, 6H), 2.83 (p, *J* = 6.8 Hz, 1H), 1.67 (s, 2H), 1.58 (s, 2H), 1.31 (d, *J* = 7.1 Hz, 3H).

^{13}C NMR (100 MHz, CDCl$_3$) δ 148.5, 147.5, 136.1, 135.5, 125.9, 120.1, 111.7, 110.9, 72.9, 55.9, 55.9, 45.5, 25.8, 18.3, 16.1.

HPLC analysis (OJ-H column, 80:20 hexanes/2-propanol, 0.6 mL/min, t = 16.4 min)

(*2S,3R*)-2-(3,4-dimethoxyphenyl)-5-methylhex-4-en-3-ol-(5c)

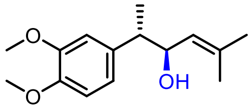

It was prepared following **General Procedure B**, using (*R*)-DTBM-Segphos as the ligand, **(*S*)-3j** as the reactant. Finally, the reaction mixture was directly purified by column chromatography on silica gel, with petroleum ether and ethyl acetate being used to obtain the corresponding product **5c**.

18.8 mg, 75% yield, >20:1 dr, colourless oil. Eluent: pentane/ethyl acetate = 10:1.

^{1}H NMR (400 MHz, CDCl$_3$) δ 6.85 – 6.68 (m, 3H), 5.10 (dq, *J* = 8.7, 1.3 Hz, 1H), 4.39 (dd, *J* = 8.8, 5.9 Hz, 1H), 3.87 (d, *J* = 4.1 Hz, 6H), 2.83 (p, *J* = 6.8 Hz, 1H), 1.66 (s, 3H), 1.58 (s, 3H), 1.31 (d, *J* = 7.1 Hz, 3H).

^{13}C NMR (100 MHz, CDCl$_3$) δ 148.6, 147.5, 136.1, 135.5, 125.9, 120.1, 111.7, 110.9, 72.9, 55.9, 55.9, 45.5, 25.8, 18.3, 16.1.

HPLC analysis (OJ-H column, 80:20 hexanes/2-propanol, 0.6 mL/min, t = 14.1 min)

(*2S,3S*)-2-(3,4-dimethoxyphenyl)-5-methylhex-4-en-3-ol-(5d)

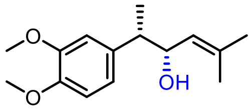

It was prepared following **General Procedure B**, using (*S*)-DTBM-Segphos as the ligand, **(*S*)-3j** as the reactant. Finally, the reaction mixture was directly purified by column chromatography on silica gel, with petroleum ether and ethyl acetate being used to obtain the corresponding product **5d**.

22.3 mg, 89% yield, >20:1 dr, colourless oil. Eluent: pentane/ethyl acetate = 10:1.

^{1}H NMR (400 MHz, CDCl$_3$) δ 6.87 – 6.76 (m, 3H), 5.19 (dq, *J* = 8.8, 1.5 Hz, 1H), 4.33 (t, *J* = 8.6 Hz, 1H), 3.88 (d, *J* = 8.9 Hz, 6H), 2.74 – 2.63 (m, 1H), 1.77 (s, 3H), 1.70 (s, 3H), 1.18 (d, *J* = 7.1 Hz, 3H).

^{13}C NMR (100 MHz, CDCl$_3$) δ 149.0, 147.8, 136.6, 136.0, 126.0, 119.9, 111.4, 111.3, 73.2, 55.9, 55.9, 46.7, 26.0, 18.6, 17.9.

HPLC analysis (OJ-H column, 80:20 hexanes/2-propanol, 0.6 mL/min, t = 17.7 min)

7. Characterization Data

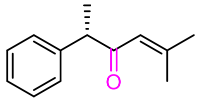

(*S*)-5-Methyl-2-phenylhex-4-en-3-one (3a)

14.5 mg, 77% yield, 96:4 er, colourless oil, $[\alpha]_D^{20}$ = +207.6 (*c* = 0.8, CH_2Cl_2). Eluent: pentane/ethyl acetate = 50:1.

^{1}H NMR (400 MHz, $CDCl_3$) δ 7.32 (t, *J* = 7.3 Hz, 2H), 7.28 – 7.19 (m, 3H), 5.99 (s, 1H), 3.73 (q, *J* = 7.0 Hz, 1H), 2.14 (s, 3H), 1.79 (s, 3H), 1.39 (d, *J* = 7.0 Hz, 3H).

^{13}C NMR (100 MHz, $CDCl_3$) δ 200.4, 156.2, 141.3, 128.8, 128.0, 126.8, 123.1, 53.5, 27.8, 20.9, 17.6.

HRMS (ESI-TOF) m/z: $[M+H]^+$ Calcd for $C_{13}H_{17}O^+$ 189.1274 Found: 189.1269.

HPLC analysis (OD-H column, 99:1 hexanes/2-propanol, 1.0 mL/min, t_{minor} = 14.9 min, t_{major} = 9.8 min)

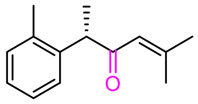

(*S*)-5-Methyl-2-(o-tolyl)hex-4-en-3-one (3b)

14.3 mg, 71% yield, 92:8 er, colourless oil, $[\alpha]_D^{20}$ = +215.8 (*c* = 0.8, CH_2Cl_2). Eluent: pentane/ethyl acetate = 50:1.

^{1}H NMR (400 MHz, $CDCl_3$) δ 7.18 – 7.13 (m, 3H), 7.08 – 7.05 (m, 3H), 5.86 (p, *J* = 1.3 Hz, 1H), 3.92 (q, *J* = 6.9 Hz, 1H), 2.37 (s, 3H), 2.16 (d, *J* = 1.4 Hz, 3H), 1.77 (d, *J* = 1.3 Hz, 3H), 1.35 (d, *J* = 7.0 Hz, 3H).

^{13}C NMR (100 MHz, $CDCl_3$) δ 200.9, 155.9, 139.8, 135.8, 130.6, 127.3, 126.7, 126.5, 123.2, 49.7, 27.8, 20.9, 19.8, 16.8.

HRMS (ESI-TOF) m/z: $[M+H]^+$ Calcd for $C_{14}H_{19}O^+$ 189.1274 Found: 189.1276.

HPLC analysis (OD-H column, 100:0 hexanes/2-propanol, 0.7 mL/min, t_{minor} = 22.4 min, t_{major} = 23.9 min)

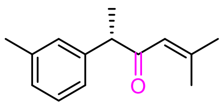

(*S*)-5-Methyl-2-(m-tolyl)hex-4-en-3-one (3c)

14.0 mg, 69% yield, 93:7 er, colourless oil, $[\alpha]_D^{20}$ = 217.1 (*c* = 0.8, CH_2Cl_2). Eluent: pentane/ethyl acetate = 50:1-20:1.

^{1}H NMR (400 MHz, $CDCl_3$) δ 7.20 (t, *J* = 7.9 Hz, 1H), 7.06 – 7.01 (m, 3H), 6.00 (p, *J* = 1.3 Hz, 1H), 3.69 (q, *J* = 6.9 Hz, 1H), 2.33 (s, 3H), 2.14 (d, *J* = 1.2 Hz, 3H), 1.79 (d, *J* = 1.3 Hz, 3H), 1.38 (d, *J* = 7.0 Hz, 3H).

^{13}C NMR (100 MHz, $CDCl_3$) ^{13}C NMR (100 MHz, $CDCl_3$) δ 200.5, 156.0, 141.2, 138.4, 128.7, 128.7, 127.6, 125.1, 123.1, 53.5, 27.8, 21.4, 20.9, 17.6.

HRMS (ESI-TOF) m/z: $[M+H]^+$ Calcd for $C_{14}H_{19}O^+$ 189.1274 Found: 189.1273.

HPLC analysis (OJ-H column, 98:2 hexanes/2-propanol, 0.7 mL/min, t_{minor} = 11.4 min, t_{major} = 8.6 min)

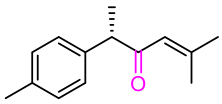

(*S*)-5-Methyl-2-(p-tolyl)hex-4-en-3-one (3d)

15.8 mg, 78% yield, 92:8 er, colourless oil, $[\alpha]_D^{20}$ = +87.7 (*c* = 1.5, CH_2Cl_2). Eluent: pentane/ethyl acetate = 50:1.

H NMR (400 MHz, $CDCl_3$ δ 7.14 – 7.09 (m, 4H), 5.99 (p, *J* = 1.3 Hz, 1H), 3.70 (q, *J* = 7.0 Hz, 1H), 2.32 (s, 3H), 2.14 (d, *J* = 1.2 Hz, 3H), 1.79 (d, *J* = 1.2 Hz, 3H), 1.37 (d, *J* = 6.9 Hz, 3H).

^{13}C NMR (100 MHz, $CDCl_3$) δ 200.6, 156.0, 138.3, 136.5, 129.5, 127.9, 123.1, 53.1, 27.8, 21.1, 20.8, 17.6.

HRMS (ESI-TOF) m/z: $[M+H]^+$ Calcd for $C_{14}H_{19}O^+$ 189.1274 Found: 189.1274.

HPLC analysis (OD-H column, 100:0 hexanes/2-propanol, 0.7 mL/min, t_{minor} = 17.4 min, t_{major} = 17.9 min)

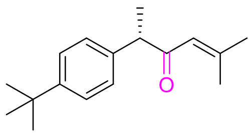

(*S*)-2-(4-(*tert*-Butyl)phenyl)-5-methylhex-4-en-3-one (3e)

18.3 mg, 75% yield, >99:1 er, colourless oil, $[\alpha]_D^{20}$ = +199.5 (c = 1.6, CH_2Cl_2). Eluent: pentane/ethyl acetate = 50:1.

^{1}H NMR (400 MHz, $CDCl_3$) δ 7.33 (d, J = 8.3 Hz, 2H), 7.14 (d, J = 8.3 Hz, 2H), 6.02 (s, 1H), 3.72 (q, J = 7.0 Hz, 1H), 2.14 (d, J = 1.3 Hz, 3H), 1.80 (d, J = 1.2 Hz, 3H), 1.38 (d, J = 7.0 Hz, 3H), 1.30 (s, 9H).

^{13}C NMR (100 MHz, $CDCl_3$) ^{13}C NMR (100 MHz, $CDCl_3$) δ 200.7, 156.0, 149.6, 138.1, 127.6, 125.7, 123.2, 53.0, 31.4, 27.8, 20.9, 17.6.

HRMS (ESI-TOF) m/z: $[M+H]^+$ Calcd for $C_{17}H2_5O^+$ 245.1900 Found: 245.1903.

HPLC analysis (OD-H column, 100:0 hexanes/2-propanol, 0.7 mL/min, t_{minor} = 14.0 min, t_{major} = 15.4 min)

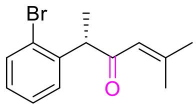

(*S*)-2-(2-Bromophenyl)-5-methylhex-4-en-3-one (3f)

17.6 mg, 66% yield, 92:8 er, colourless oil, $[\alpha]_D^{20}$ = +211.7 (c = 0.2, CH_2Cl_2). Eluent: pentane/ethyl acetate = 50:1.

^{1}H NMR (400 MHz, $CDCl_3$) δ 7.32 (t, J = 7.5 Hz, 2H), 7.24 – 7.20 (m, 2H), 5.99 (s, 1H), 3.73 (q, J = 6.9 Hz, 1H), 2.14 (s, 3H), 1.79 (s, 3H), 1.39 (d, J = 7.0 Hz, 3H).

^{13}C NMR (100 MHz, $CDCl_3$) δ 200.4, 156.2, 141.3, 128.8, 128.0, 126.8, 123.1, 53.5, 27.8, 20.9, 17.6.

HRMS (ESI-TOF) m/z: $[M+H]^+$ Calcd for $C_{13}H_{16}BrO^+$ 267.0379 Found: 267.0381.

HPLC analysis (OD-H column, 100:0 hexanes/2-propanol, 0.7 mL/min, t_{minor} = 21.8 min, t_{major} = 22.7 min)

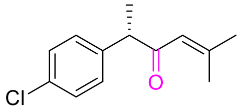

(*S*)-2-(4-Chlorophenyl)-5-methylhex-4-en-3-one (3g)

15.5 mg, 70% yield, 84:16 er, colourless oil, $[\alpha]_D^{20}$: +219.0 (c = 0.3, CH_2Cl_2). Eluent: pentane/ethyl acetate = 50:1.

^{1}H NMR (400 MHz, $CDCl_3$) δ 7.29 (d, J = 8.5 Hz, 2H), 7.15 (d, J = 8.4 Hz, 2H), 5.97 (p, J = 1.3 Hz, 1H), 3.71 (q, J = 7.0 Hz, 1H), 2.14 (s, 3H), 1.81 (s, 3H), 1.37 (d, J = 7.0 Hz, 3H).

^{13}C NMR (100 MHz, $CDCl_3$) ^{13}C NMR (100 MHz, DMSO) δ 199.8, 157.0, 139.8, 132.7, 129.3, 128.9, 122.9, 52.8, 27.9, 20.9, 17.6.

HRMS (ESI-TOF) m/z: $[M+H]^+$ Calcd for $C_{13}H_{16}ClO^+$ 223.0884 Found: 223.0882.

HPLC analysis (OD-H column, 100:0 hexanes/2-propanol, 0.8 mL/min, t_{minor} = 16.6 min, t_{major} = 15.1 min)

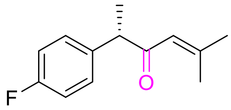

(*S*)-2-(4-Fluorophenyl)-5-methylhex-4-en-3-one (3h)

14.0 mg, 68% yield, 86:14 er, colourless oil, $[\alpha]_D^{20}$ = +164.8 (c = 1.0, CH_2Cl_2). Eluent: dichloromethane/methanol = 50:1.

^{1}H NMR (400 MHz, $CDCl_3$) δ 7.23 – 7.13 (m, 2H), 7.00 (t, J = 8.7 Hz, 2H), 5.98 (p, J = 1.3 Hz, 1H), 3.72 (q, J = 7.0 Hz, 1H), 2.14 (d, J = 1.3 Hz, 3H), 1.81 (d, J = 1.3 Hz, 3H), 1.38 (d, J = 7.0 Hz, 3H).

^{13}C NMR (100 MHz, $CDCl_3$) δ 200.2, 161.8 (d, J = 240 Hz), 156.7, 137.0 (d, J = 10 Hz), 129.5 (d, J = 10 Hz), 122.9, 115.6 (d, J = 20 Hz), 52.6, 27.8, 20.9, 17.7.

^{19}F NMR (376 MHz, $CDCl_3$) δ -116.1.

HRMS (ESI-TOF) m/z: $[M+H]^+$ Calcd for $C_{13}H_{16}FO^+$ 207.1180 Found: 207.1187.

HPLC analysis (OD-H column, 100:0 hexanes/2-propanol, 0.8 mL/min, t_{minor} = 15.4 min, t_{major} = 14.1 min)

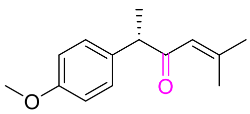

(*S*)-2-(4-Methoxyphenyl)-5-methylhex-4-en-3-one (3i)

18.1 mg, 83% yield, 96:4 er, $[\alpha]_D^{24}$ = +245.5 (c = 0.8, CH_2Cl_2). colourless oil. Eluent: pentane/ethyl acetate = 50:1.

^{1}H NMR (400 MHz, $CDCl_3$) δ 7.13 (d, J = 8.6 Hz, 2H), 6.86 (d, J = 8.7 Hz, 2H), 5.99 (p, J = 1.3 Hz, 1H), 3.78 (s, 3H), 3.68 (q, J = 6.9 Hz, 1H), 2.13 (d, J = 1.2 Hz, 3H), 1.79 (d, J = 1.3 Hz, 3H), 1.36 (d, J = 7.0 Hz, 3H).

^{13}C NMR (100 MHz, $CDCl_3$) δ 200.7, 158.5, 156.0, 133.3, 129.0, 123.1, 114.2, 55.2, 52.6, 27.8, 20.8, 17.6.

HRMS (ESI-TOF) m/z: $[M+H]^+$ Calcd for $C_{14}H_{19}O_2$ 219.1380 Found: 219.1382.

HPLC analysis (OJ-H column, 98:2 hexanes/2-propanol, 0.8 mL/min, t_{minor} = 15.8 min, t_{major} = 14.8 min)

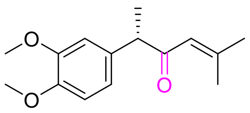

(*S*)-2-(3,4-Dimethoxyphenyl)-5-methylhex-4-en-3-one (3j)

20.3 mg, 82% yield, 99:1 er, yellow oil, $[\alpha]_D^{20}$ = +121.9 (c = 0.1, CH_2Cl_2). Eluent: pentane/ethyl acetate = 50:1-20:1.

^{1}H NMR (400 MHz, $CDCl_3$) δ 6.85 – 6.75 (m, 2H), 6.71 (d, J = 2.0 Hz, 1H), 6.01 (s, 1H), 3.86 (d, J = 2.5 Hz, 7H), 3.67 (q, J = 6.9 Hz, 1H), 2.14 (d, J = 1.2 Hz, 3H), 1.80 (d, J = 1.2 Hz, 3H), 1.38 (d, J = 7.0 Hz, 3H).

^{13}C NMR (100 MHz, $CDCl_3$) δ 200.5, 155.9, 149.1, 148.0, 133.8, 123.0, 120.2, 111.4, 110.8, 55.9, 55.8, 53.0, 27.8, 20.8, 17.6.

HRMS (ESI-TOF) m/z: $[M+H]^+$ Calcd for $C_{15}H_{21}O_3^+$ 249.1485 Found: 249.1488.

HPLC analysis (OJ-H column, 80:20 hexanes/2-propanol, 0.6 mL/min, t_{minor} = 12.6 min, t_{major} = 14.5 min)

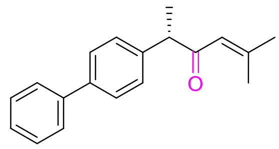

(*S*)-2-([1,1'-Biphenyl]-4-yl)-5-methylhex-4-en-3-one (3k)

22.2 mg, 84% yield, 92:8 er, colourless oil, $[\alpha]_D^{20}$ = +302.1 (*c* = 0.7, CH_2Cl_2). Eluent: pentane/ethyl acetate = 50:1.

^{1}H NMR (400 MHz, $CDCl_3$) δ 7.61 – 7.52 (m, 4H), 7.43 (t, *J* = 7.6 Hz, 2H), 7.32 (dd, *J* = 19.2, 7.8 Hz, 3H), 6.04 (p, *J* = 1.3 Hz, 1H), 3.78 (q, *J* = 7.0 Hz, 1H), 2.16 (d, *J* = 1.2 Hz, 3H), 1.82 (d, *J* = 1.2 Hz, 3H), 1.43 (d, *J* = 7.0 Hz, 3H).

^{13}C NMR (100 MHz, $CDCl_3$) δ 200.3, 156.4, 140.8, 140.3, 139.8, 128.7, 128.4, 127.5, 127.2, 127.0, 123.1, 53.2, 27.8, 20.9, 17.6.

HRMS (ESI-TOF) m/z: $[M+H]^+$ Calcd for $C_{19}H_{21}O^+$ 265.1587 Found: 265.1586.

HPLC analysis (OJ-H column, 98:2 hexanes/2-propanol, 0.8 mL/min, t_{minor} = 16.7 min, t_{major} = 20.9 min)

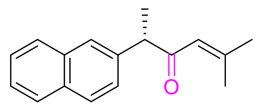

(*S*)-5-Methyl-2-(naphthalen-2-yl)hex-4-en-3-one (3l)

15.9 mg, 67% yield, 95:5 er, colourless oil, $[\alpha]_D^{20}$ = +242.6 (*c* = 1.0, CH_2Cl_2). Eluent: pentane/ethyl acetate = 50:1.

^{1}H NMR (400 MHz, $CDCl_3$) δ 7.80 (dd, *J* = 8.9, 3.2 Hz, 3H), 7.68 (s, 1H), 7.50 – 7.39 (m, 2H), 7.33 (dd, *J* = 8.5, 1.7 Hz, 1H), 6.02 (s, 1H), 3.89 (q, *J* = 6.9 Hz, 1H), 2.15 (d, *J* = 1.3 Hz, 3H), 1.75 (d, *J* = 1.3 Hz, 3H), 1.48 (d, *J* = 6.9 Hz, 3H).

^{13}C NMR (100 MHz, $CDCl_3$) δ 200.3, 156.4, 138.8, 133.7, 132.5, 128.5, 127.7, 127.7, 126.7, 126.2, 126.2, 125.7, 123.2, 53.7, 27.8, 20.9, 17.6.

HRMS (ESI-TOF) m/z: $[M+H]^+$ Calcd for $C_{17}H_{19}O^+$ 239.1430 Found: 239.1430.

HPLC analysis (OJ-H column, 98:2 hexanes/2-propanol, 0.8 mL/min, t_{minor} = 13.8 min, t_{major} = 19.2 min)

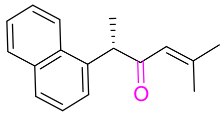

(*S*)-5-Methyl-2-(naphthalen-1-yl)hex-4-en-3-one (3m)

15.0 mg, 63% yield, 95:5 er, colourless oil, $[\alpha]_D^{20}$ = +220.3 (*c* = 0.2, CH_2Cl_2). Eluent: pentane/ethyl acetate = 50:1.

^{1}H NMR (400 MHz, $CDCl_3$) δ 8.09 (d, *J* = 8.3 Hz, 1H), 7.88 (d, *J* = 8.5 Hz, 1H), 7.77 (d, *J* = 8.1 Hz, 1H), 7.57 – 7.47 (m, 2H), 7.47 – 7.41 (m, 1H), 7.31 (d, *J* = 7.1 Hz, 1H), 5.91 (p, *J* = 1.4 Hz, 1H), 4.46 (q, *J* = 7.0 Hz, 1H), 2.16 (d, *J* = 1.3 Hz, 3H), 1.70 (d, *J* = 1.3 Hz, 3H), 1.54 (d, *J* = 7.0 Hz, 3H).

^{13}C NMR (100 MHz, $CDCl_3$) δ 201.0, 156.3, 137.7, 134.1, 131.7, 129.0, 127.5, 126.4, 125.8, 125.7, 125.3, 123.3, 122.9, 49.5, 27.7, 20.9, 17.2.

HRMS (ESI-TOF) m/z: $[M+H]^+$ Calcd for $C_{17}H_{19}O^+$ 239.1430 Found: 239.1432.

HPLC analysis (OJ-H column, 98:2 hexanes/2-propanol, 0.8 mL/min, t_{minor} = 11.6 min, t_{major} = 16.4 min)

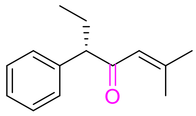

(*S*)-2-Methyl-5-phenylhept-2-en-4-one (3n)

14.7 mg, 73% yield, >99:1 er, colourless oil, $[\alpha]_D^{20}$ = +296.8 (*c* = 0.3, CH_2Cl_2). Eluent: pentane/ethyl acetate = 50:1.

^{1}H NMR (400 MHz, $CDCl_3$) δ 7.34 – 7.28 (m, 2H), 7.26 – 7.18 (m, 3H), 6.01 (t, *J* = 1.3 Hz, 1H), 3.49 (t, *J* = 7.4 Hz, 1H), 2.12 (s, 3H), 2.08 (dt, *J* = 14.2, 7.2 Hz, 1H), 1.79 (s, 3H), 1.71 (dt, *J* = 13.8, 7.4 Hz, 1H), 0.84 (t, *J* = 7.4 Hz, 3H).

^{13}C NMR (100 MHz, $CDCl_3$) δ 200.1, 156.0, 139.7, 128.7, 128.4, 126.9, 123.7, 61.5, 27.7, 25.3, 20.8, 12.2.

HRMS (ESI-TOF) m/z: $[M+H]^+$ Calcd for $C_{14}H_{19}O^+$ 203.1430 Found: 203.1436.

HPLC analysis (OJ-H column, 100:0 hexanes/2-propanol, 0.8 mL/min, t_{minor} = 9.5 min, t_{major} = 11.8 min)

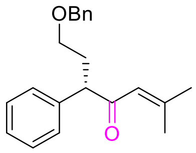

(*S*)-7-(Benzyloxy)-2-methyl-5-phenylhept-2-en-4-one (3o)

18.5 mg, 60% yield, 97:3 er, colourless oil, $[\alpha]_D^{20}$ = +201.6 (*c* = 1.8, CH_2Cl_2). Eluent: pentane/ethyl acetate = 40:1.

^{1}H NMR (400 MHz, $CDCl_3$) δ 7.35 – 7.18 (m, 10H), 6.00 (dt, *J* = 2.5, 1.2 Hz, 1H), 4.42 (s, 2H), 3.89 (t, *J* = 7.4 Hz, 1H), 3.48 – 3.41 (m, 1H), 3.36 – 3.24 (m, 1H), 2.46 – 2.35 (m, 1H), 2.11 (d, *J* = 1.4 Hz, 3H), 2.00 – 1.87 (m, 1H), 1.77 (s, 3H).

^{13}C NMR (100 MHz, $CDCl_3$) δ 204.16, 135.06, 129.06, 129.03, 128.04, 49.10, 39.32, 36.47, 28.27.

HRMS (ESI-TOF) m/z: $[M+H]^+$ Calcd for $C_{21}H_{25}O_2^+$ 309.1849 Found: 309.1845.

HPLC analysis (OJ-H column, 99:1 hexanes/2-propanol, 0.8 mL/min, t_{minor} = 12.4 min, t_{major} = 15.8 min)

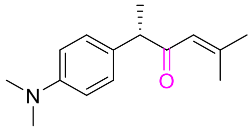

(*S*)-2-(4-(Dimethylamino)phenyl)-5-methylhex-4-en-3-one (3p)

18.9 mg, 82% yield, >99:1 er, colourless oil, $[\alpha]_D^{20}$ = +278.8 (*c* = 0.4, CH_2Cl_2). Eluent: pentane/ethyl acetate = 50:1-20:1.

^{1}H NMR (400 MHz, $CDCl_3$) δ 7.08 (d, *J* = 8.7 Hz, 2H), 6.69 (d, *J* = 8.7 Hz, 2H), 6.01 (s, 1H), 3.63 (q, *J* = 6.9 Hz, 1H), 2.92 (s, 6H), 2.13 (s, 3H), 1.78 (s, 3H), 1.35 (d, *J* = 6.9 Hz, 3H).

^{13}C NMR (100 MHz, $CDCl_3$) δ 201.0, 155.3, 149.5, 129.0, 128.6, 123.2, 112.9, 52.6, 40.6, 27.8, 20.8, 17.5.

HRMS (ESI-TOF) m/z: $[M+H]^+$ Calcd for $C_{15}H_{22}NO^+$ 232.1696 Found: 232.1699.

HPLC analysis (OJ-H column, 97:3 hexanes/2-propanol, 0.8 mL/min, t_{minor} = 7.1 min, t_{major} = 6.7 min)

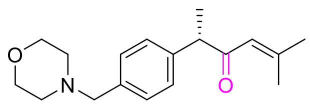

(*S*)-5-Methyl-2-(4-(morpholinomethyl)phenyl)hex-4-en-3-one (3q)

22.7 mg, 79% yield, 95:5 er, colourless oil, $[\alpha]_D^{20}$ = +148.8 (*c* = 1.1, CH_2Cl_2). Eluent: pentane/ethyl acetate = 30:1.

^{1}H NMR (400 MHz, $CDCl_3$) δ 7.27 (d, *J* = 8.0 Hz, 2H), 7.17 (d, *J* = 8.0 Hz, 2H), 6.00 (s, 1H), 3.73 – 3.68 (m, 5H), 3.47 (s, 2H), 2.44 (t, *J* = 4.6 Hz, 4H), 2.14 (s, 3H), 1.80 (s, 3H), 1.38 (d, *J* = 7.0 Hz, 3H).

^{13}C NMR (100 MHz, $CDCl_3$) δ 200.4, 156.2, 140.1, 136.3, 129.6, 127.9, 123.1, 67.0, 63.1, 53.6, 53.2, 27.8, 20.9, 17.6.

HRMS (ESI-TOF) m/z: $[M+H]^+$ Calcd for $C_{18}H_{26}NO_2^+$ 288.1958 Found: 288.1960.

HPLC analysis (OJ-H column, 97:3 hexanes/2-propanol, 0.8 mL/min, t_{minor} = 9.8 min, t_{major} = 9.5 min)

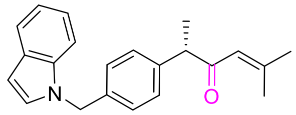

(*S*)-2-(4-((1*H*-indol-1-yl)methyl)phenyl)-5-methylhex-4-en-3-one (3r)

20.3 mg, 64% yield, 92:8 er, colourless oil, $[\alpha]_D^{20}$ = +176.0 (*c* = 0.2, CH_2Cl_2). Eluent: pentane/ethyl acetate = 30:1.

^{1}H NMR (400 MHz, $CDCl_3$) δ 7.65 (d, *J* = 7.8 Hz, 1H), 7.29 (d, *J* = 9.1 Hz, 1H), 7.20 – 7.04 (m, 7H), 6.55 (d, *J* = 3.2 Hz, 1H), 5.95 (p, *J* = 1.4 Hz, 1H), 5.29 (s, 2H), 3.69 (q, *J* = 7.0 Hz, 1H), 2.12 (d, *J* = 1.2 Hz, 3H), 1.78 (d, *J* = 1.3 Hz, 3H), 1.35 (d, *J* = 7.0 Hz, 3H).

^{13}C NMR (100 MHz, $CDCl_3$) δ 200.1, 156.5, 140.7, 136.3, 136.2, 128.7, 128.4, 128.2, 127.2, 123.0, 121.7, 121.0, 119.5, 109.7, 101.7, 53.1, 49.8, 27.8, 20.9, 17.6.

HRMS (ESI-TOF) m/z: $[M+H]^+$ Calcd for $C_{22}H_{24}NO^+$ 318.1852 Found: 318.1852.

HPLC analysis (OJ-H column, 97:3 hexanes/2-propanol, 0.8 mL/min, t_{minor} = 9.2 min, t_{major} = 8.9 min)

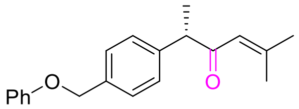

(*S*)-5-Methyl-2-(4-(phenoxymethyl)phenyl)hex-4-en-3-one (3s)

23.5 mg, 80% yield, 99:1 er, colourless oil, $[\alpha]_D^{20}$ = +0.86 (*c* = 0.3, CH_2Cl_2). Eluent: pentane/ethyl acetate = 40:1.

^{1}H NMR (400 MHz, $CDCl_3$) δ 7.28 (d, *J* = 15.8 Hz, 2H), 7.14 (s, 2H), 7.07 (s, 1H), 6.96 (d, *J* = 8.1 Hz, 2H), 6.91 (d, *J* = 8.5 Hz, 2H), 5.97 (s, 1H), 3.69 (s, 1H), 2.10 (s, 3H), 1.78 (s, 3H), 1.35 (d, *J* = 7.0 Hz, 3H).

^{13}C NMR (100 MHz, $CDCl_3$) δ 200.4, 157.1, 156.3, 156.2, 136.0, 129.7, 129.2, 123.3, 123.0, 119.0, 119.0, 52.8, 27.8, 20.9, 17.7.

HRMS (ESI-TOF) m/z: $[M+H]^+$ Calcd for $C_{20}H_{23}O_2^+$ 295.1693 Found: 295.1699.

HPLC analysis (OJ-H column, 99:1 hexanes/2-propanol, 0.8 mL/min, t_{minor} = 9.2 min, t_{major} = 8.9 min)

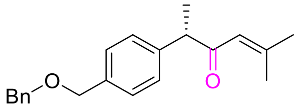

(*S*)-5-Methyl-2-(4-(phenoxymethyl)phenyl)hex-4-en-3-one (3t)

26.5 mg, 86% yield, 96:4 er, colourless oil, $[\alpha]_D^{20}$ = +173.4 (*c* = 0.9, CH_2Cl_2). Eluent: pentane/ethyl acetate = 40:1.

^{1}H NMR (400 MHz, $CDCl_3$) δ 7.38 – 7.27 (m, 7H), 7.22 – 7.18 (m, 2H), 5.98 (p, *J* = 1.4 Hz, 1H), 4.57 (s, 2H), 4.52 (s, 2H), 3.73 (q, *J* = 6.9 Hz, 1H), 2.13 (s, 3H), 1.78 (s, 3H), 1.38 (d, *J* = 7.0 Hz, 3H).

^{13}C NMR (100 MHz, $CDCl_3$) δ 200.3, 156.2, 140.7, 138.3, 136.9, 128.4, 128.3, 128.1, 127.8, 127.7, 123.1, 72.3, 71.9, 53.3, 27.8, 20.9, 17.6.

HRMS (ESI-TOF) m/z: $[M+H]^+$ Calcd for $C_{21}H_{25}O_2^+$ 309.1849 Found: 309.1851.

HPLC analysis (OJ-H column, 98:2 hexanes/2-propanol, 0.7 mL/min, t_{minor} = 14.8 min, t_{major} = 25.7 min)

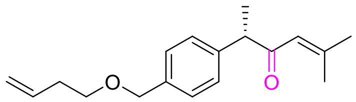

(*S*)-5-Methyl-2-(4-(phenoxymethyl)phenyl)hex-4-en-3-one (3u)

21.2 mg, 78% yield, 95:5 er, colourless oil, $[\alpha]_D^{20}$ = +191.4 (*c* = 1.3, CH_2Cl_2). Eluent: pentane/ethyl acetate = 40:1.

^{1}H NMR (400 MHz, $CDCl_3$) δ 7.29 (d, *J* = 8.1 Hz, 2H), 7.19 (d, *J* = 8.1 Hz, 2H), 5.98 (p, *J* = 1.4 Hz, 1H), 5.92 – 5.76 (m, 1H), 5.16 – 4.98 (m, 2H), 4.49 (s, 2H), 3.73 (q, *J* = 6.9 Hz, 1H), 3.54 (t, *J* = 6.8 Hz, 2H), 2.38 (qt, *J* = 6.7, 1.4 Hz, 2H), 2.13 (s, 3H), 1.79 (s, 3H), 1.38 (d, *J* = 7.0 Hz, 3H).

^{13}C NMR (100 MHz, $CDCl_3$) δ 200.3, 156.2, 140.6, 137.0, 135.2, 128.2, 128.0, 123.1, 116.4, 72.7, 69.8, 53.3, 34.2, 27.8, 20.8, 17.6.

HRMS (ESI-TOF) m/z: $[M+H]^+$ Calcd for $C_{18}H_{25}O_2^+$ 273.1849 Found: 273.1851.

HPLC analysis (OJ-H column, 98:2 hexanes/2-propanol, 0.7 mL/min, t_{minor} = 6.9 min, t_{major} = 8.8 min)

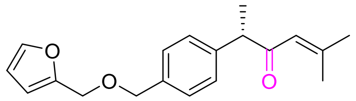

(*S*)-5-Methyl-2-(4-(phenoxymethyl)phenyl)hex-4-en-3-one (3v)

24.7 mg, 83% yield, 98:2 er, colourless oil, $[\alpha]_D^{20}$ = +166.1 (*c* = 1.0, CH_2Cl_2). Eluent: pentane/ethyl acetate = 40:1.

^{1}H NMR (400 MHz, $CDCl_3$) δ 7.38 (s, 1H), 7.27 (d, *J* = 8.0 Hz, 2H), 7.16 (d, *J* = 8.1 Hz, 2H), 6.33 – 6.28 (m, 2H), 5.94 (p, *J* = 1.3 Hz, 1H), 4.47 (d, *J* = 6.7 Hz, 4H), 3.69 (q, *J* = 6.9 Hz, 1H), 2.10 (s, 3H), 1.75 (s, 3H), 1.35 (d, *J* = 6.9 Hz, 3H).

^{13}C NMR (100 MHz, $CDCl_3$) δ 200.2, 156.2, 151.7, 142.8, 140.8, 136.5, 128.4, 128.1, 123.1, 110.3, 109.4, 71.7, 64.0, 53.3, 27.8, 20.9, 17.6.

HRMS (ESI-TOF) m/z: $[M+H]^+$ Calcd for $C_{19}H_{23}O_3^+$ 299.1642 Found: 299.1644.

HPLC analysis (OJ-H column, 93:7 hexanes/2-propanol, 0.7 mL/min, t_{minor} = 9.8 min, t_{major} = 9.4 min)

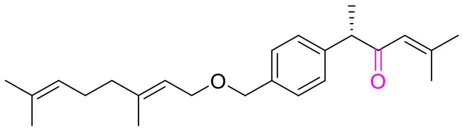

(*S*)-5-Methyl-2-(4-(phenoxymethyl)phenyl)hex-4-en-3-one (3w)

29.4 mg, 83% yield, 97:3 er, colourless oil. $[\alpha]_D^{20}$ = +140.5 (*c* = 1.4, CH_2Cl_2). Eluent: pentane/ethyl acetate = 40:1.

^{1}H NMR (400 MHz, $CDCl_3$) δ 7.30 (d, *J* = 8.0 Hz, 2H), 7.19 (d, *J* = 8.1 Hz, 2H), 5.97 (p, *J* = 1.4 Hz, 1H), 5.40 (t, *J* = 6.7 Hz, 1H), 5.10 (t, *J* = 6.7 Hz, 1H), 4.46 (s, 2H), 4.04 (d, *J* = 6.7 Hz, 2H), 3.72 (q, *J* = 6.9 Hz, 1H), 2.14 – 2.02 (m, 7H), 1.78 (s, 3H), 1.68 (s, 3H), 1.65 (s, 3H), 1.60 (s, 3H), 1.38 (d, *J* = 6.9 Hz, 3H).

^{13}C NMR (100 MHz, $CDCl_3$) δ 200.3, 156.1, 140.6, 140.4, 137.2, 131.6, 128.4, 128.0, 124.0, 123.1, 120.8, 71.7, 66.8, 53.3, 39.6, 27.7, 26.4, 25.7, 20.8, 17.7, 17.6, 16.5.

HRMS (ESI-TOF) m/z: $[M+H]^+$ Calcd for $C_{24}H_{35}O_2^+$ 355.2632 Found: 355.2630.

HPLC analysis (OJ-H column, 95:5 hexanes/2-propanol, 0.7 mL/min, t_{minor} = 9.2 min, t_{major} = 14.9 min)

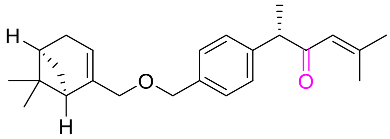

(*S*)-5-Methyl-2-(4-(phenoxymethyl)phenyl)hex-4-en-3-one (3x)

28.2 mg, 80% yield, 99:1 dr, colourless oil, $[\alpha]_D^{20}$ = +147.7 (*c* = 1.4, CH_2Cl_2). Eluent: pentane/ethyl acetate = 30:1.

^{1}H NMR (400 MHz, $CDCl_3$) δ 7.28 (d, *J* = 8.0 Hz, 2H), 7.18 (d, *J* = 8.0 Hz, 2H), 5.98 (p, *J* = 1.3 Hz, 1H), 5.52 (dt, *J* = 3.1, 1.5 Hz, 1H), 4.43 (s, 2H), 3.90 (s, 2H), 3.72 (q, *J* = 6.9 Hz, 1H), 2.43 – 2.20 (m, 4H), 2.13 (s, 4H), 1.78 (s, 3H), 1.38 (d, *J* = 7.0 Hz, 3H), 1.29 (s, 3H), 1.19 (d, *J* = 8.6 Hz, 1H), 0.86 (s, 3H).

^{13}C NMR (100 MHz, $CDCl_3$) δ 200.3, 156.1, 145.4, 140.5, 137.3, 128.2, 128.0, 123.1, 120.1, 73.3, 71.4, 53.3, 43.4, 40.9, 38.0, 31.6, 31.3, 27.8, 26.2, 21.1, 20.8, 17.6.

HRMS (ESI-TOF) m/z: $[M+H]^+$ Calcd for $C_{24}H_{33}O_2^+$ 353.2475 Found: 353.2477.

HPLC analysis (OJ-H column, 96:4 hexanes/2-propanol, 0.7 mL/min, t_{minor} = 14.5 min,

t_{major} = 11.3 min)

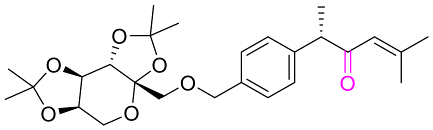

(*S*)-5-Methyl-2-(4-(phenoxymethyl)phenyl)hex-4-en-3-one (3y)

34.5 mg, 75% yield, 98:2 dr, colourless oil, $[\alpha]_D^{20}$ = +55.4 (c = 0.7, CH_2Cl_2). Eluent: pentane/ethyl acetate = 20:1.

^{1}H NMR (400 MHz, $CDCl_3$) δ 7.29 (d, J = 8.0 Hz, 2H), 7.18 (d, J = 8.0 Hz, 2H), 5.97 (s, 1H), 4.67 – 4.54 (m, 3H), 4.43 (d, J = 2.5 Hz, 1H), 4.23 (d, J = 7.9 Hz, 1H), 3.92 (dd, J = 13.0, 1.6 Hz, 1H), 3.77 – 3.65 (m, 2H), 3.60 (q, J = 10.6 Hz, 2H), 2.14 (s, 3H), 1.79 (s, 3H), 1.55 (s, 3H), 1.41 (d, J = 7.9 Hz, 6H), 1.37 (d, J = 6.9 Hz, 3H), 1.33 (s, 3H).

^{13}C NMR (100 MHz, $CDCl_3$) δ 200.3, 156.2, 140.5, 136.7, 128.0, 127.9, 123.1, 108.9, 108.5, 102.7, 73.4, 71.6, 71.0, 70.2, 70.2, 61.0, 53.3, 27.8, 26.6, 25.8, 25.4, 24.0, 20.8, 17.6.

HRMS (ESI-TOF) m/z: $[M+H]^+$ Calcd for $C_{26}H_{37}O_7^+$ 461.2534 Found: 461.2533.

HPLC analysis (OJ-H column, 88:12 hexanes/2-propanol, 0.6 mL/min, t_{minor} = 18.0 min, t_{major} = 14.2 min)

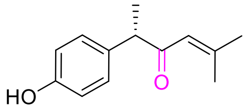

(S)-2-(4-hydroxyphenyl)-5-methylhex-4-en-3-one (3z)

5.5 mg, 27% yield, 95:5 er, colourless oil, $[\alpha]_D^{20}$ = +237.3 (c = 0.5, CH_2Cl_2). Eluent: pentane/ethyl acetate = 5:1.

^{1}H NMR (400 MHz, $CDCl_3$) δ 7.07 (d, J = 8.5 Hz, 2H), 6.81 (d, J = 8.5 Hz, 2H), 6.01 (s, 1H), 3.68 (q, J = 7.0 Hz, 1H), 2.13 (s, 3H), 1.80 (s, 3H), 1.36 (d, J = 7.0 Hz, 3H).

^{13}C NMR (100 MHz, $CDCl_3$) δ 201.5, 156.7, 154.9, 132.9, 129.1, 123.0, 115.8, 52.7, 27.9, 20.9, 17.6.

HRMS (ESI-TOF) m/z: $[M+H]^+$ Calcd for $C_{13}H_{17}O_2^+$ 205.1223 Found: 205.1227.

HPLC analysis (OJ-H column, 85:15 hexanes/2-propanol, 0.6 mL/min, t_{minor} = 16.5

min, t_{major} = 15.4 min)

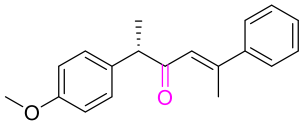

(*S*)-5-Methyl-2-(4-(phenoxymethyl)phenyl)hex-4-en-3-one (3aa)

18.2 mg, 65% yield, 97:3 er, colourless oil, $[\alpha]_D^{20}$ = +32.7 (*c* = 0.5, CH_2Cl_2). Eluent: pentane/ethyl acetate = 30:1.

^{1}H NMR (400 MHz, $CDCl_3$) δ 7.39 – 7.28 (m, 5H), 7.17 (d, *J* = 8.6 Hz, 2H), 6.86 (d, *J* = 8.7 Hz, 2H), 6.42 (q, *J* = 1.3 Hz, 1H), 3.85 – 3.75 (m, 4H), 2.53 (s, 3H), 1.43 (d, *J* = 7.0 Hz, 3H).

^{13}C NMR (100 MHz, $CDCl_3$) δ 201.1, 158.6, 154.3, 142.7, 133.1, 129.0, 129.0, 128.5, 126.5, 123.7, 114.3, 55.3, 53.2, 18.5, 17.7.

HRMS (ESI-TOF) m/z: $[M+H]^+$ Calcd for $C_{19}H_{21}O_2^+$ 281.1536 Found: 281.1545.

HPLC analysis (OJ-H column, 88:12 hexanes/2-propanol, 0.6 mL/min, t_{minor} = 9.9 min, t_{major} = 9.4 min)

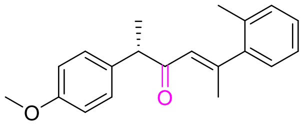

(*S*)-2-(4-Methoxyphenyl)-5-(o-tolyl)hex-4-en-3-one (3ab)

19.4 mg, 66% yield, 88:12 er, colourless oil, $[\alpha]_D^{20}$ = +76.6 (*c* = 1.6, CH_2Cl_2). Eluent: pentane/ethyl acetate = 30:1.

^{1}H NMR (400 MHz, $CDCl_3$) δ 7.18 – 7.09 (m, 5H), 6.97 – 6.93 (m, 1H), 6.88 – 6.82 (m, 2H), 6.05 (d, *J* = 1.5 Hz, 1H), 3.78 – 33.70 (m, 4H), 2.39 (d, *J* = 1.5 Hz, 3H), 2.08 (s, 3H), 1.42 (d, *J* = 6.9 Hz, 3H).

^{13}C NMR (100 MHz, $CDCl_3$) δ 201.1, 158.6, 156.4, 144.2, 134.0, 133.0, 130.4, 129.0, 127.6, 127.1, 126.0, 125.7, 114.3, 55.3, 53.1, 21.2, 19.7, 17.4.

HRMS (ESI-TOF) m/z: $[M+H]^+$ Calcd for $C_{20}H_{23}O_2^+$ 295.1693 Found: 295.1693.

HPLC analysis (OJ-H column, 98:2 hexanes/2-propanol, 0.9 mL/min, t_{minor} = 25.2 min, t_{major} = 14.4 min)

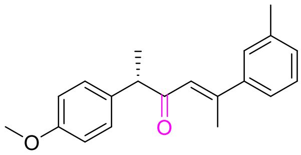

(*S*)-2-(4-Methoxyphenyl)-5-(m-tolyl)hex-4-en-3-one (3ac)

20.6 mg, 70% yield, 99:1 er, colourless oil, $[\alpha]_D^{20}$ = +20.8 (*c* = 0.2, CH_2Cl_2). Eluent: pentane/ethyl acetate = 30:1.

^{1}H NMR (400 MHz, CDCl$_3$) δ 7.24 – 7.10 (m, 6H), 6.89 – 6.83 (m, 2H), 6.40 (q, *J* = 1.3 Hz, 1H), 3.85 – 3.77 (m, 4H), 2.51 (d, *J* = 1.3 Hz, 3H), 2.34 (s, 3H), 1.43 (d, *J* = 6.9 Hz, 3H).

^{13}C NMR (100 MHz, CDCl$_3$) δ 201.1, 158.6, 154.6, 142.8, 138.1, 133.1, 129.7, 129.0, 128.3, 127.2, 123.6, 123.6, 114.3, 55.3, 53.2, 21.5, 18.6, 17.7.

HRMS (ESI-TOF) m/z: $[M+H]^+$ Calcd for $C_{20}H_{23}O_2^+$ 295.1693 Found: 295.1699.

HPLC analysis (OJ-H column, 98:2 hexanes/2-propanol, 0.9 mL/min, t_{minor} = 14.5 min, t_{major} = 20.7 min)

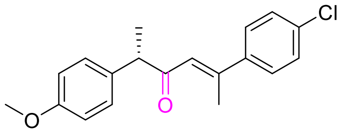

(*S*)-5-(4-Chlorophenyl)-2-(4-methoxyphenyl)hex-4-en-3-one (3ad)

18.5 mg, 59% yield, 99:1 er, colourless oil, $[\alpha]_D^{20}$ = -14.8 (*c* = 0.2, CH_2Cl_2). Eluent: pentane/ethyl acetate = 30:1.

^{1}H NMR (400 MHz, CDCl$_3$) δ 7.28 (d, *J* = 1.8 Hz, 4H), 7.16 (d, *J* = 8.7 Hz, 2H), 6.87 (d, *J* = 8.6 Hz, 2H), 6.38 (q, *J* = 1.3 Hz, 1H), 3.85 – 3.76 (m, 4H), 2.49 (d, *J* = 1.3 Hz, 3H), 1.42 (d, *J* = 6.9 Hz, 3H).

^{13}C NMR (100 MHz, CDCl$_3$) δ 200.9, 158.6, 152.8, 141.0, 134.9, 132.9, 129.0, 128.6, 127.8, 123.9, 114.3, 55.3, 53.3, 18.3, 17.6.

HRMS (ESI-TOF) m/z: $[M+H]^+$ Calcd for $C_{19}H_{20}ClO_2^+$ 315.1146 Found: 315.1150.

HPLC analysis (OJ-H column, 98:2 hexanes/2-propanol, 0.9 mL/min, t_{minor} = 10.2 min, t_{major} = 9.4 min)

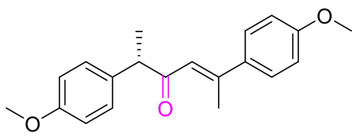

(*S*)-2-(4-Methoxyphenyl)-5-(m-tolyl)hex-4-en-3-one (3ae)

25.1 mg, 81% yield, 96:4 er, colourless oil, $[\alpha]_D^{20}$ = -46.5 (*c* = 0.5, CH_2Cl_2). Eluent: pentane/ethyl acetate = 20:1.

^{1}H NMR (400 MHz, $CDCl_3$) δ 7.33 (d, *J* = 8.9 Hz, 2H), 7.18 (d, *J* = 8.7 Hz, 2H), 6.85 (dd, *J* = 8.8, 6.8 Hz, 4H), 6.41 (q, *J* = 1.2 Hz, 1H), 3.84 – 3.77 (m, 7H), 2.52 (d, *J* = 1.2 Hz, 3H), 1.43 (d, *J* = 6.9 Hz, 3H).

^{13}C NMR (100 MHz, $CDCl_3$) δ 201.0, 160.5, 158.6, 153.9, 134.7, 133.3, 129.0, 127.9, 122.1, 114.3, 113.8, 55.3, 55.2, 53.2, 18.2, 17.7.

HRMS (ESI-TOF) m/z: $[M+H]^+$ Calcd for $C_{20}H_{23}O_3^+$ 311.1642 Found: 311.1648.

HPLC analysis (OJ-H column, 95:5 hexanes/2-propanol, 0.7 mL/min, t_{minor} = 16.9 min, t_{major} = 16.4 min)

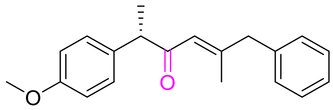

(*S*)-2-(4-methoxyphenyl)-5-methyl-6-phenylhex-4-en-3-one (3af)

15.9 mg, 54% yield, 97:3 er, colourless oil, $[\alpha]_D^{20}$ = +165.0 (*c* = 0.2, CH_2Cl_2). Eluent: pentane/ethyl acetate = 30:1.

^{1}H NMR (400 MHz, $CDCl_3$) δ 7.26 – 7.20 (m, 3H), 7.10 (d, *J* = 8.5 Hz, 2H), 7.03 – 6.96 (m, 2H), 6.86 (d, *J* = 8.6 Hz, 2H), 6.00 (s, 1H), 3.81 (s, 3H), 3.69 (q, *J* = 7.0 Hz, 1H), 3.30 (s, 2H), 2.04 (d, *J* = 1.2 Hz, 3H), 1.37 (d, *J* = 7.0 Hz, 3H).

^{13}C NMR (100 MHz, $CDCl_3$) δ 200.9, 158.6, 156.8, 137.8, 133.2, 129.0, 129.0, 128.4, 126.6, 124.2, 114.2, 55.3, 52.8, 47.2, 19.1, 17.4.

HRMS (ESI-TOF) m/z: $[M+H]^+$ Calcd for $C_{20}H_{23}O_2^+$ 295.1693 Found: 295.1692.

HPLC analysis (OJ-H column, 95:5 hexanes/2-propanol, 0.7 mL/min, t_{minor} = 36.2 min, t_{major} = 19.8 min)

8. Copies of NMR Spectra

^{1}H NMR (400 MHz, $CDCl_3$) - (A1)

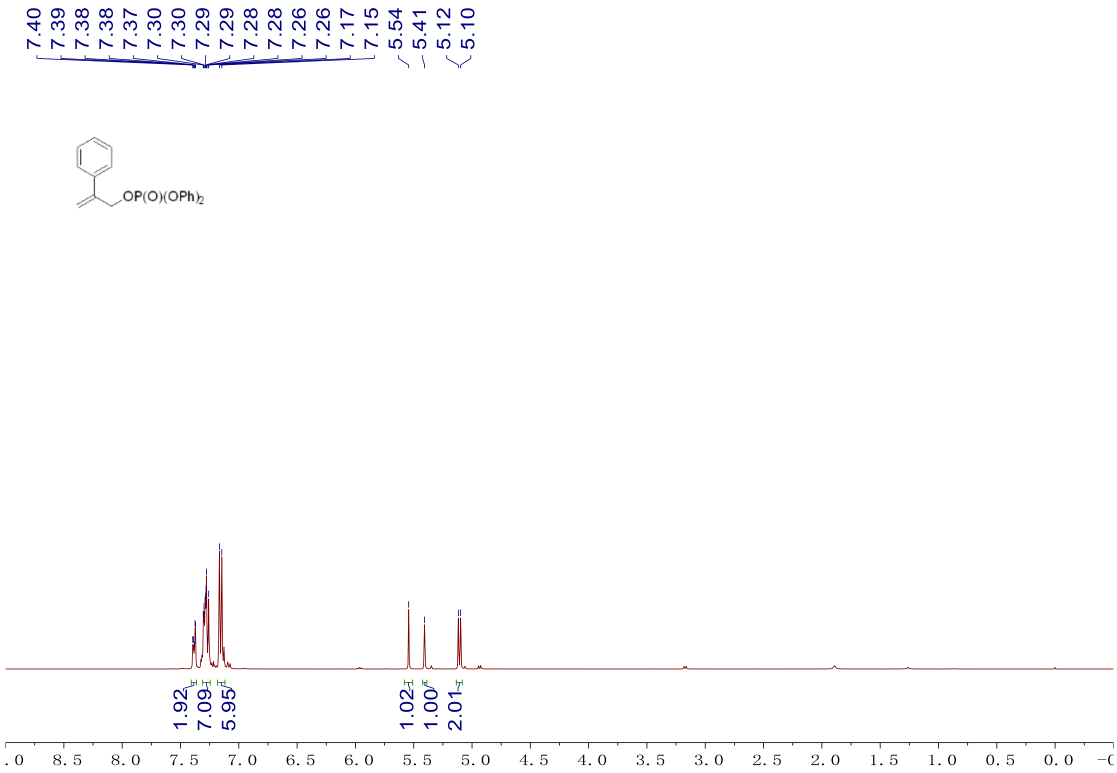

^{1}H NMR (400 MHz, $CDCl_3$) - (A2)

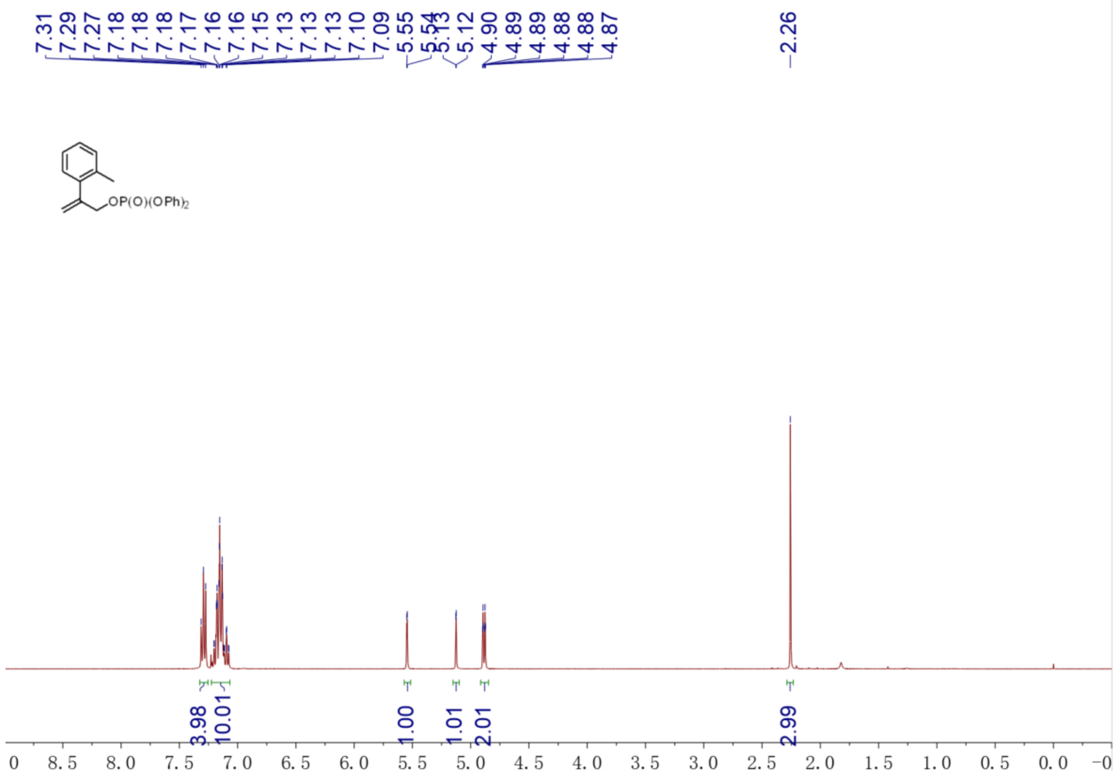

^{1}H NMR (400 MHz, $CDCl_3$) - (A3)

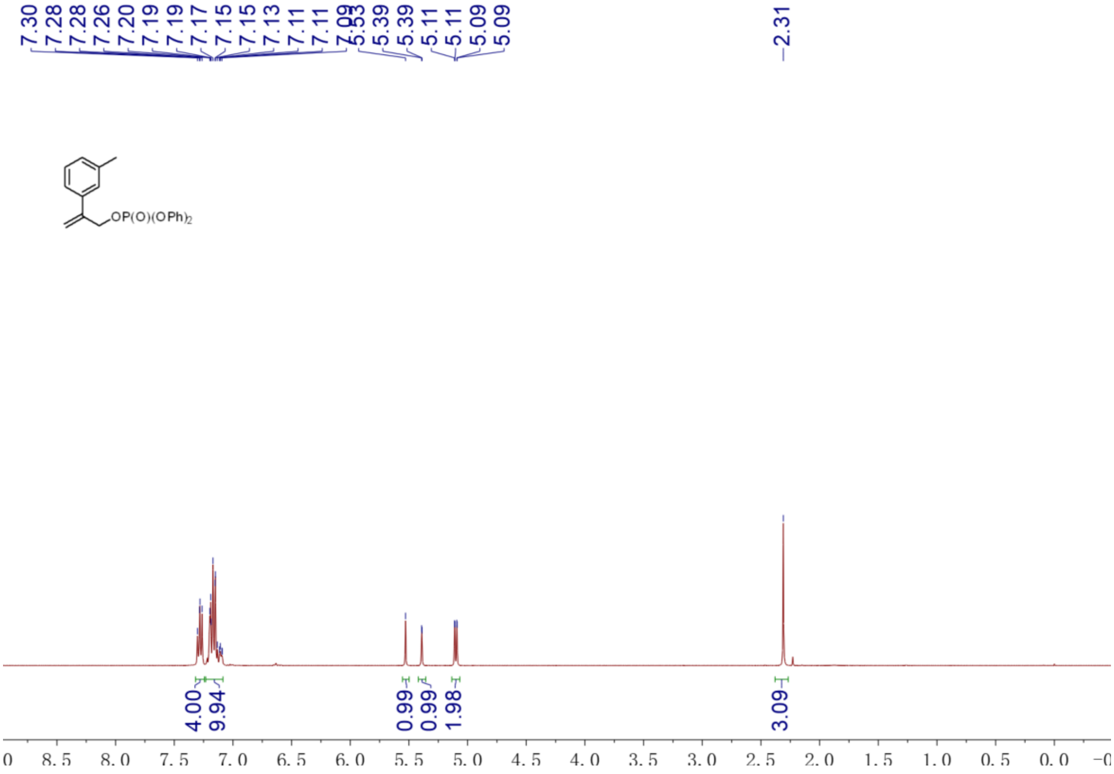

^{1}H NMR (400 MHz, $CDCl_3$) - (A4)

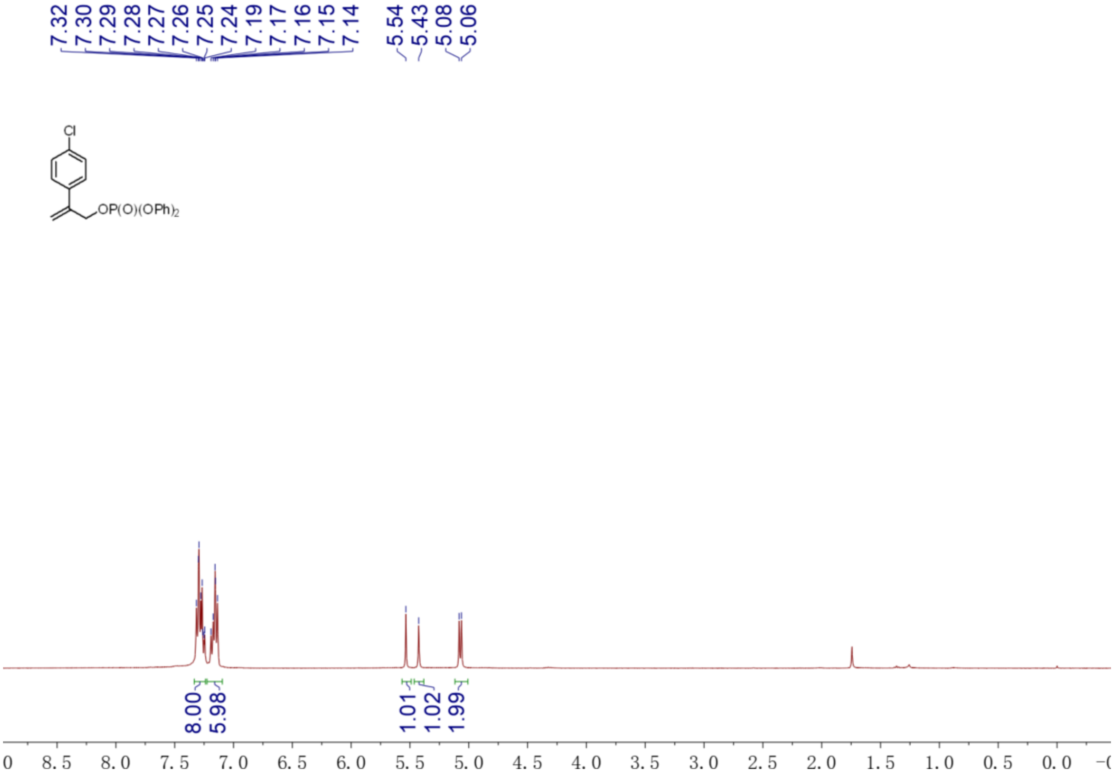

^{1}H NMR (400 MHz, $CDCl_3$) - (A5)

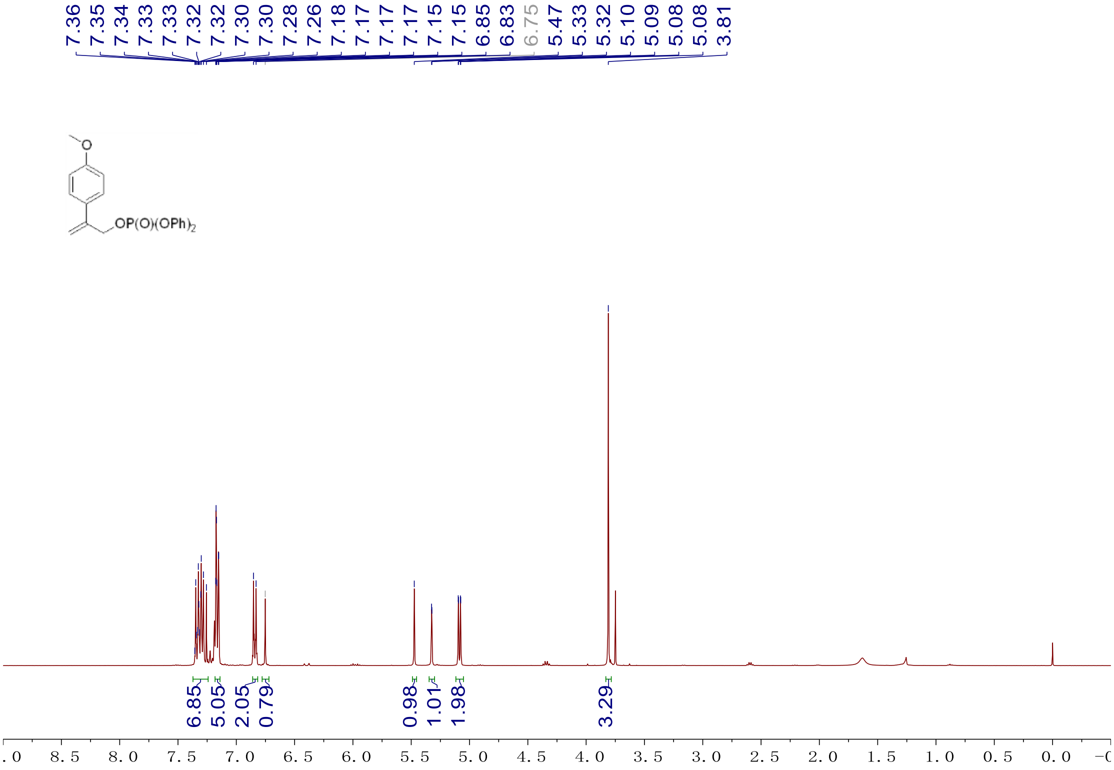

^{1}H NMR (400 MHz, $CDCl_3$) - (A6)

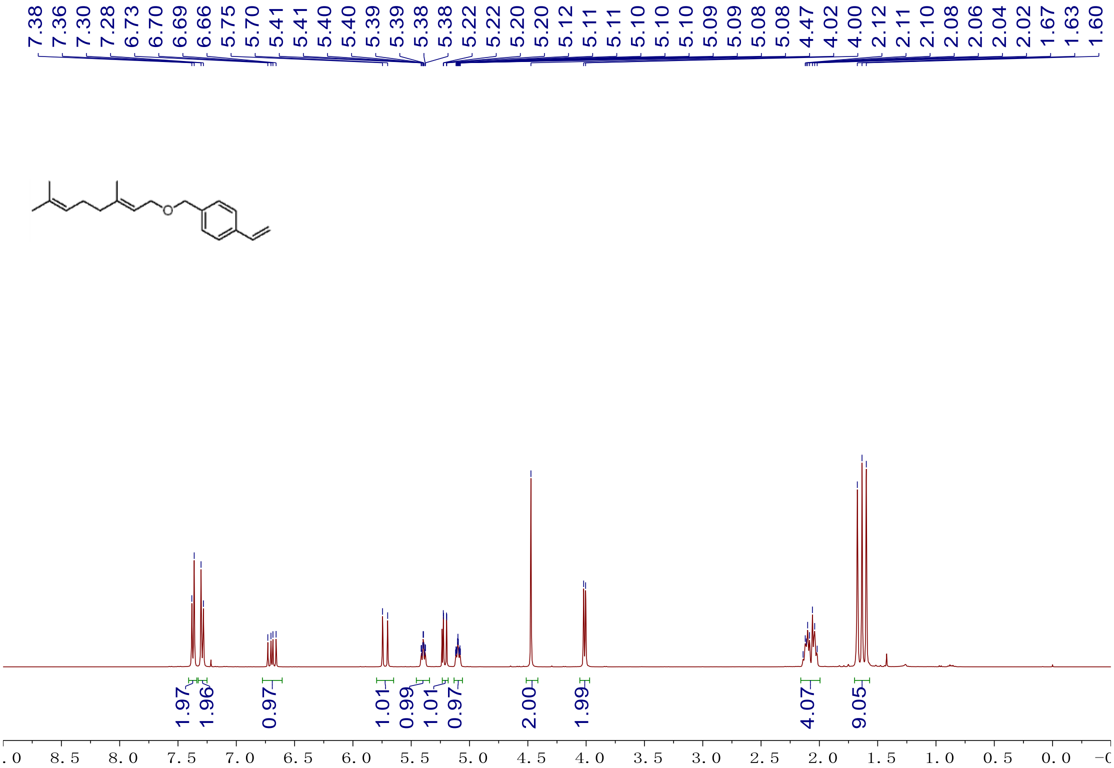

^{1}H NMR (400 MHz, $CDCl_3$) - (A7)

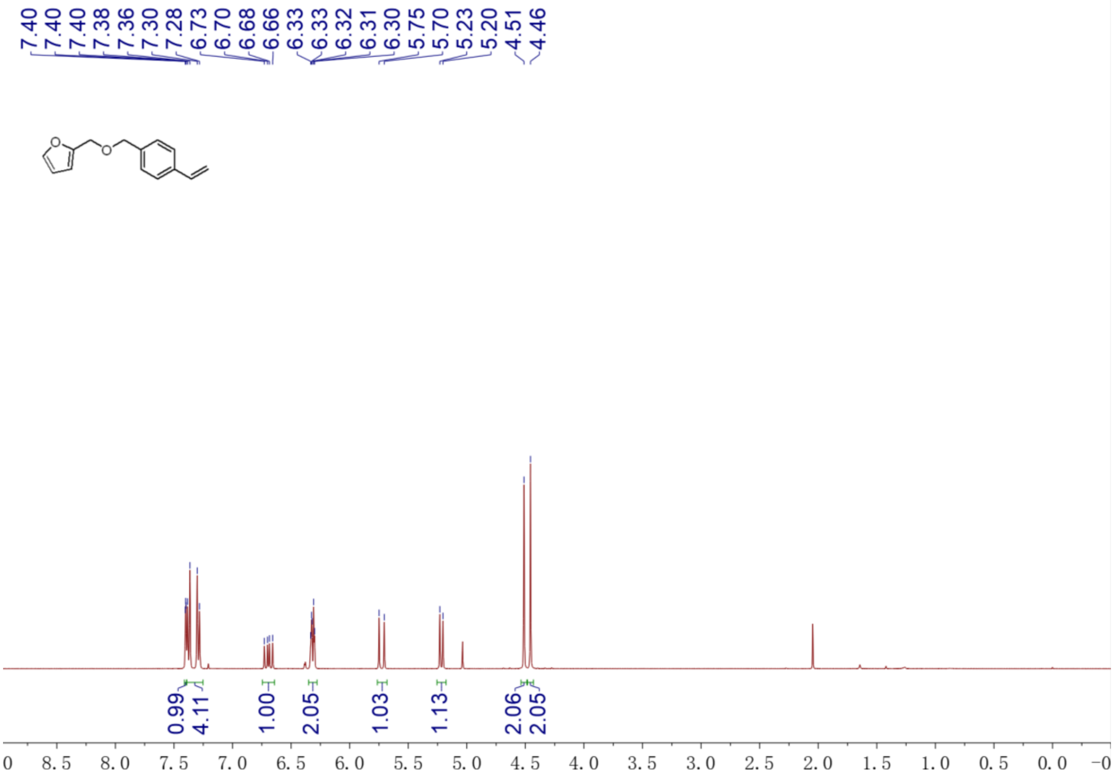

^{1}H NMR (400 MHz, $CDCl_3$) - (A8)

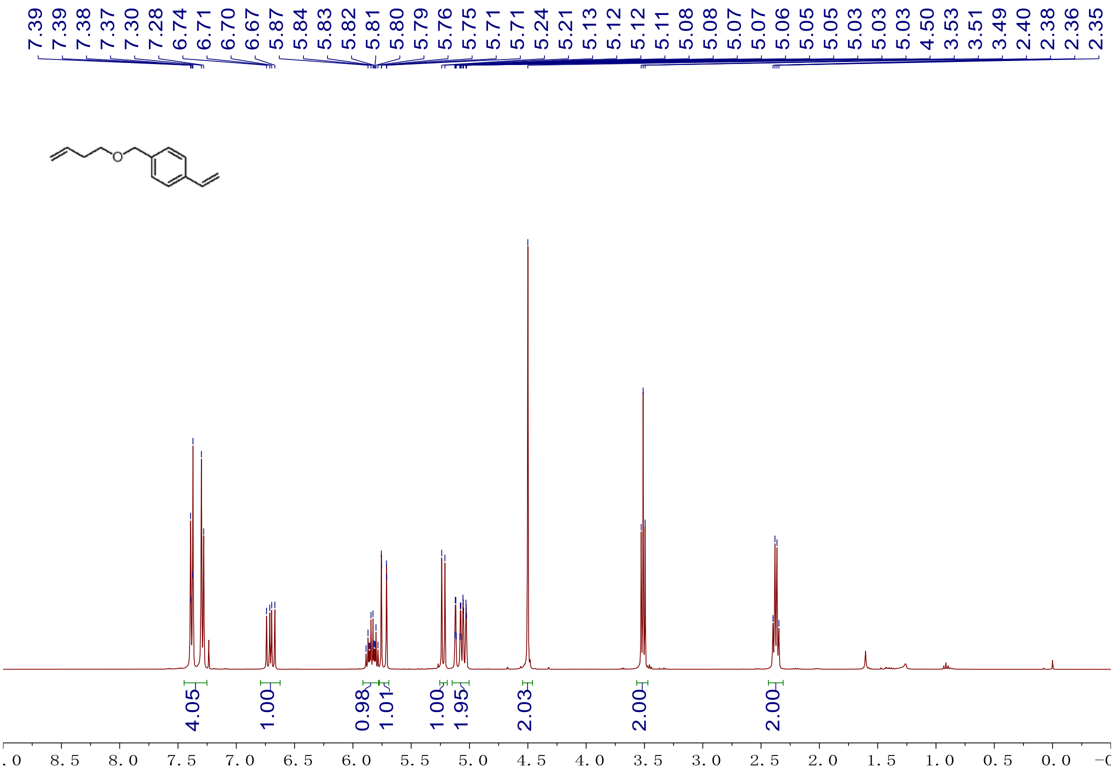

^{1}H NMR (400 MHz, $CDCl_3$) - (A9)

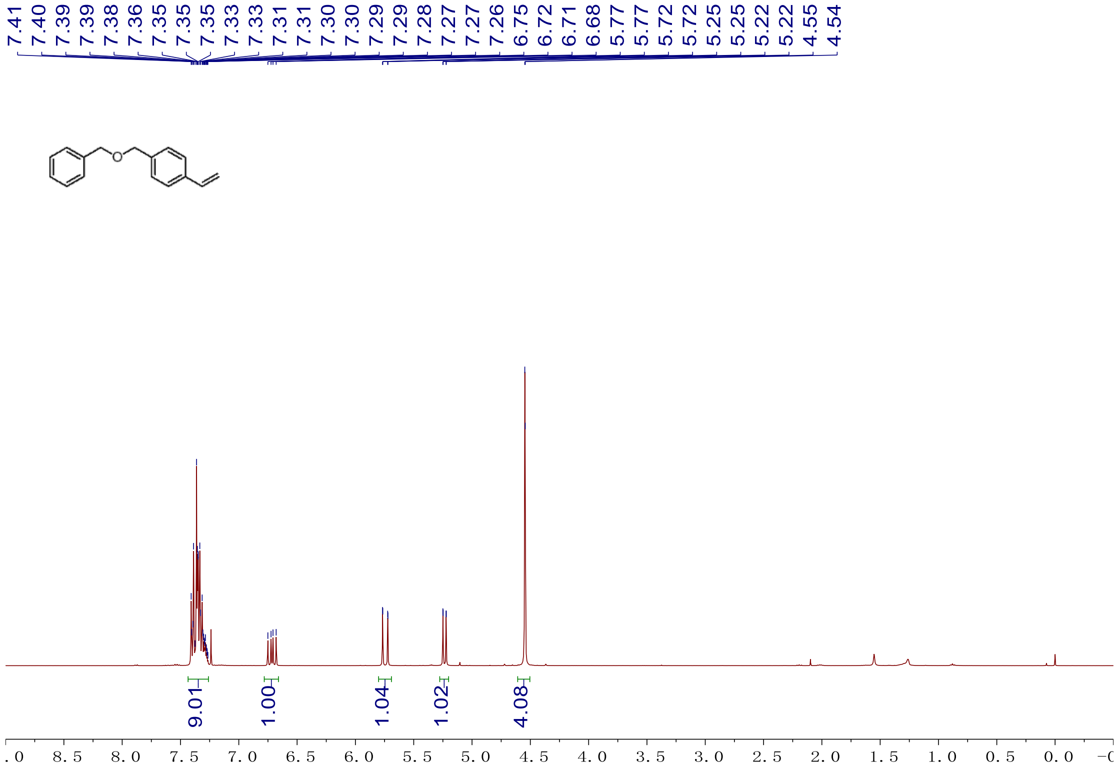

^{1}H NMR (400 MHz, $CDCl_3$) - (A10)

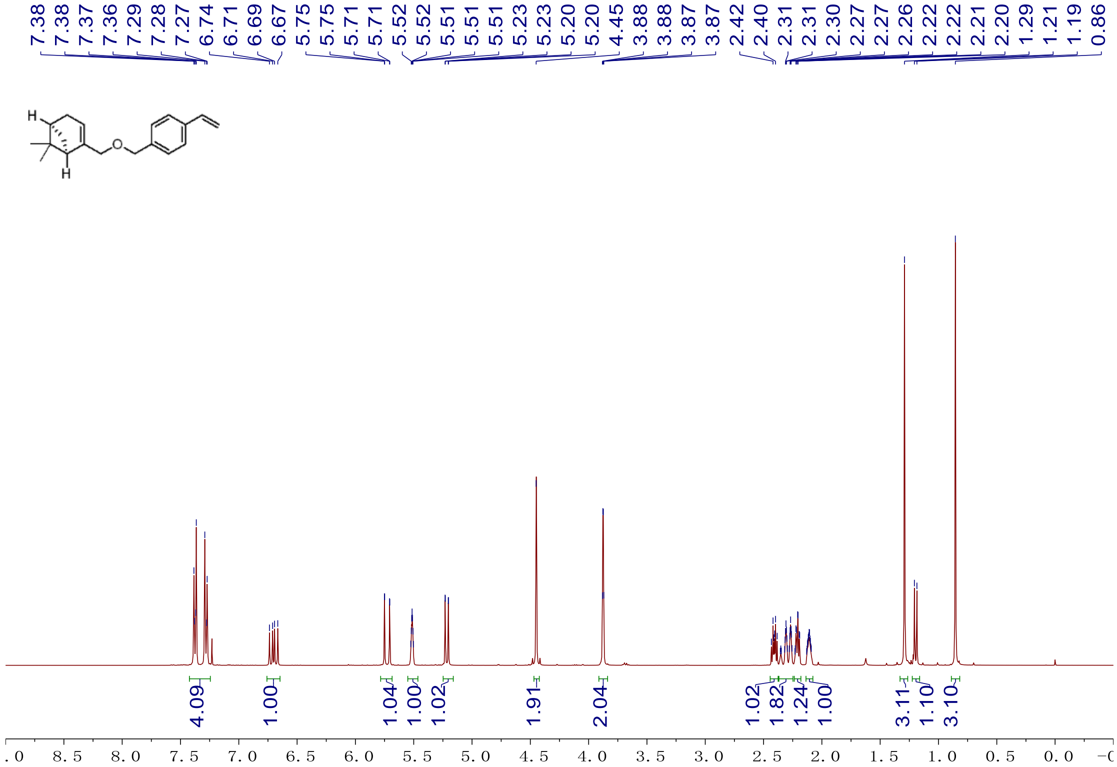

^1H NMR (400 MHz, $CDCl_3$) - (A11)

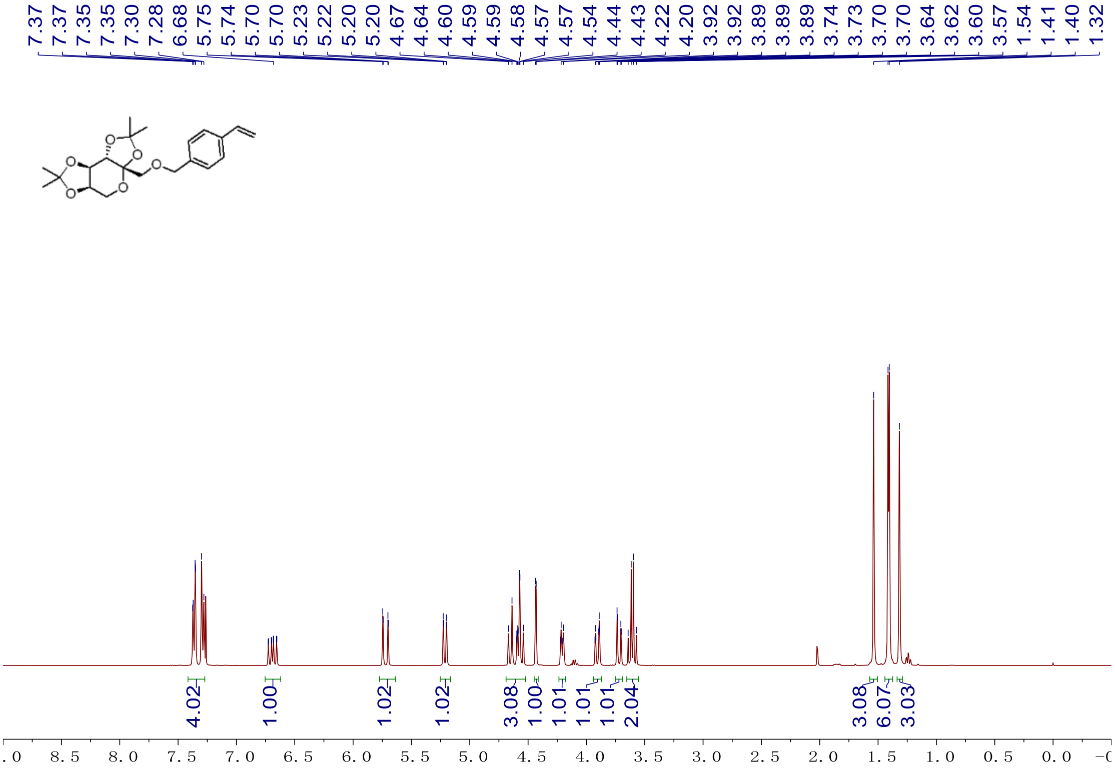

^1H NMR (400 MHz, $CDCl_3$) - (A12)

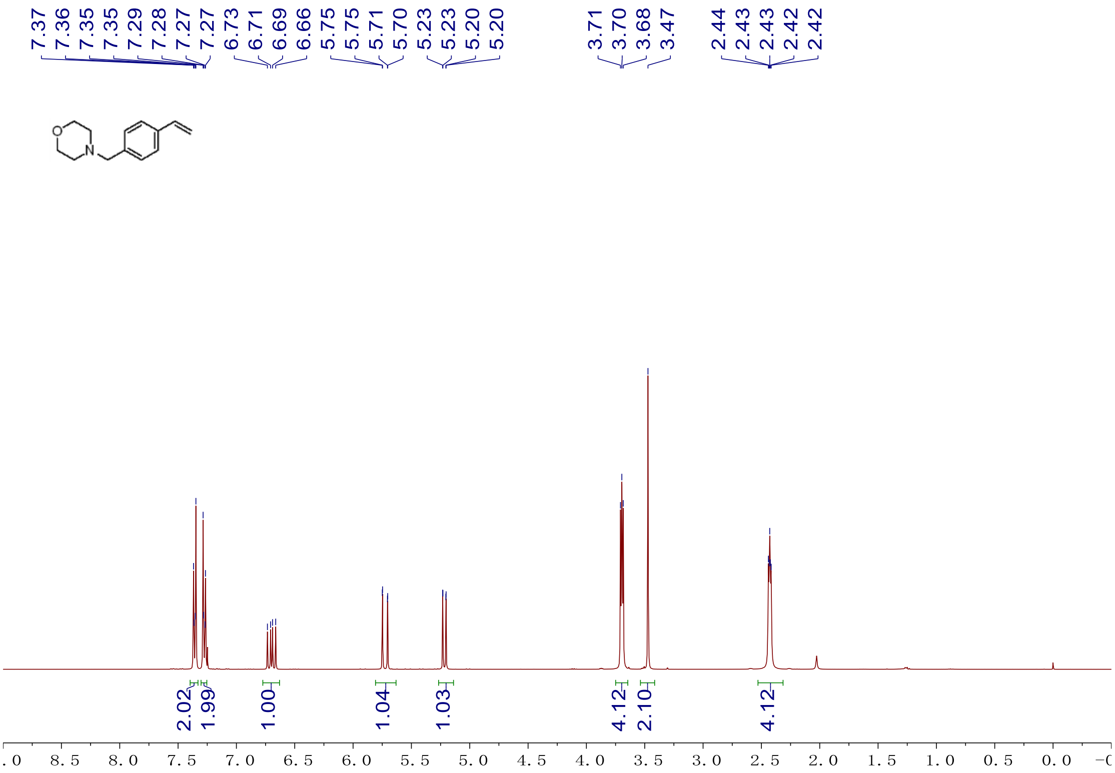

^{1}H NMR (400 MHz, $CDCl_3$) - (A13)

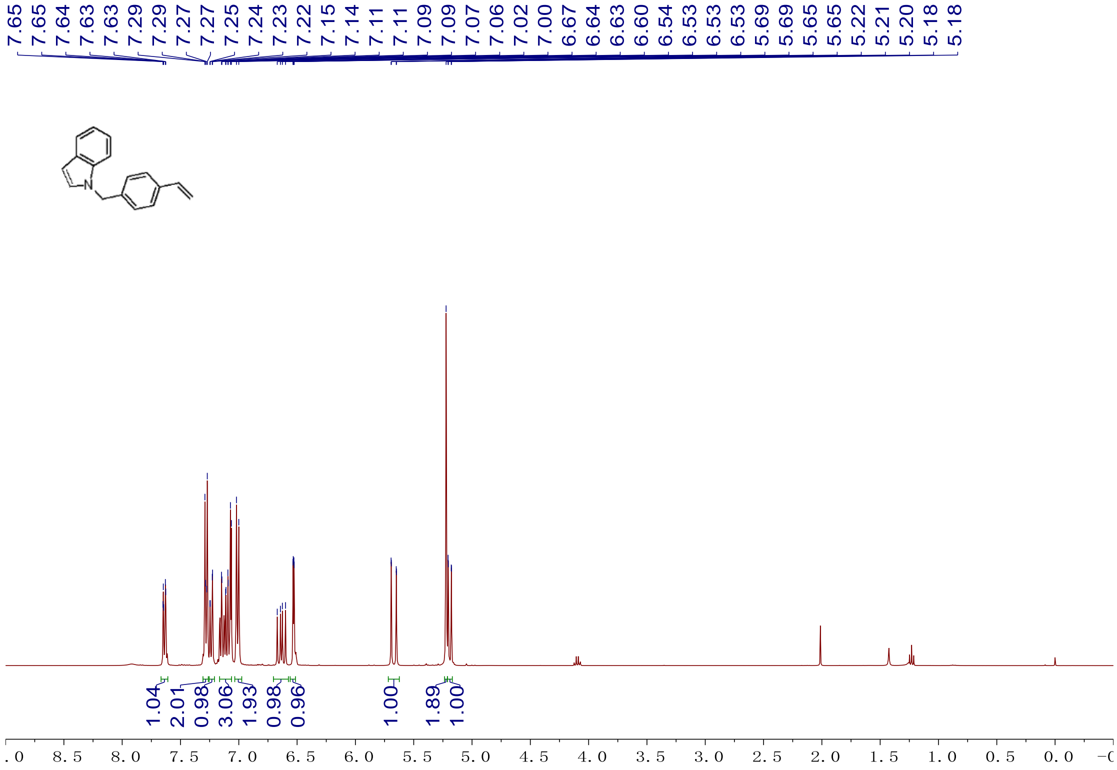

^{1}H NMR (400 MHz, CDCl$_3$) - (3a)

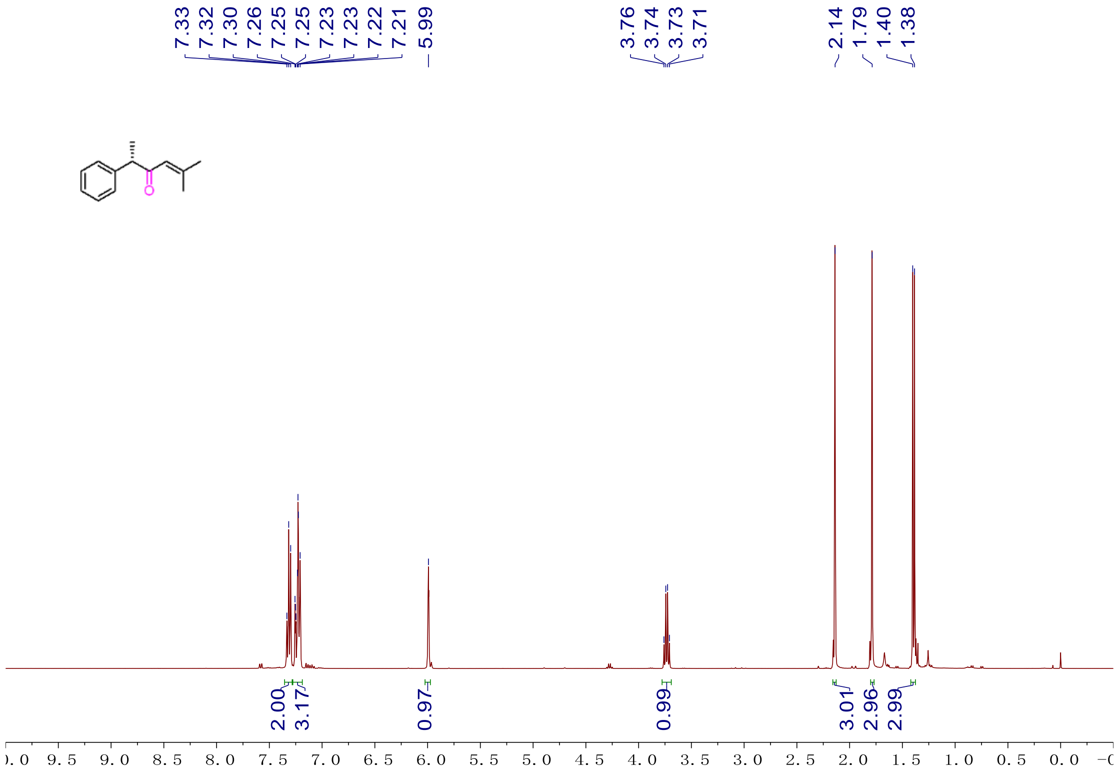

^{13}C NMR (100 MHz, CDCl$_3$) - (3a)

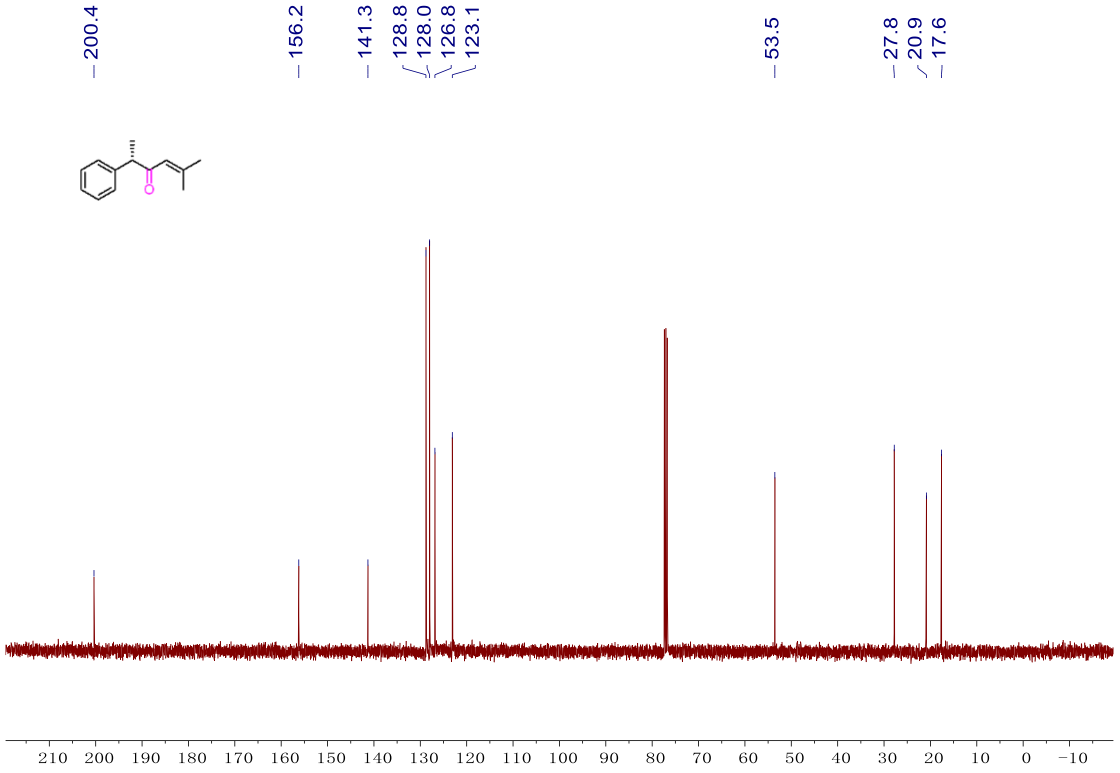

^{1}H NMR (400 MHz, CDCl3) - (3b)

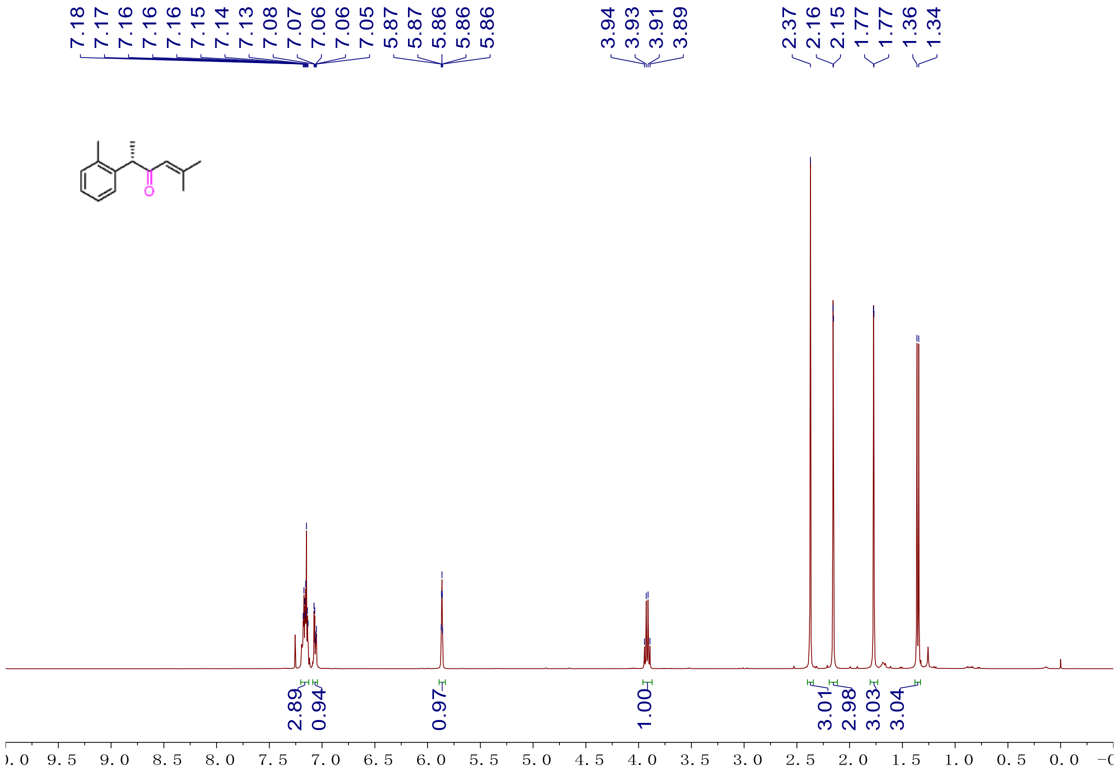

^{13}C NMR (100 MHz, CDCl3) - (3b)

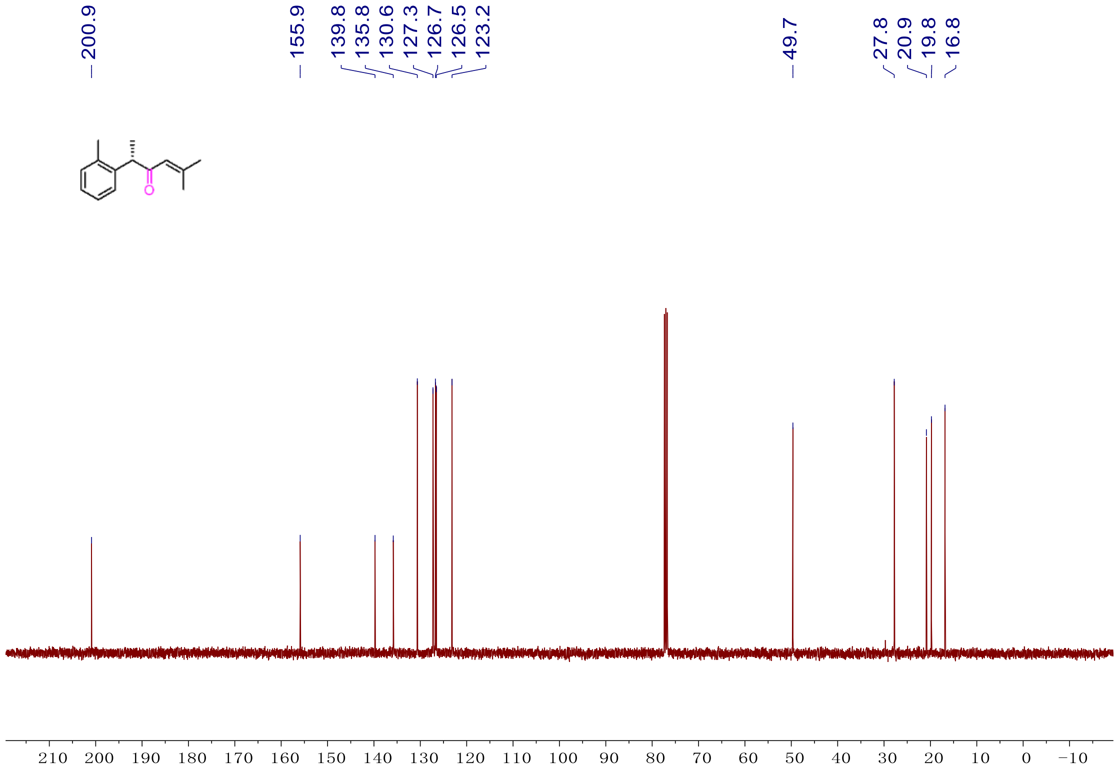

^{1}H NMR (400 MHz, $CDCl_3$) - (3c)

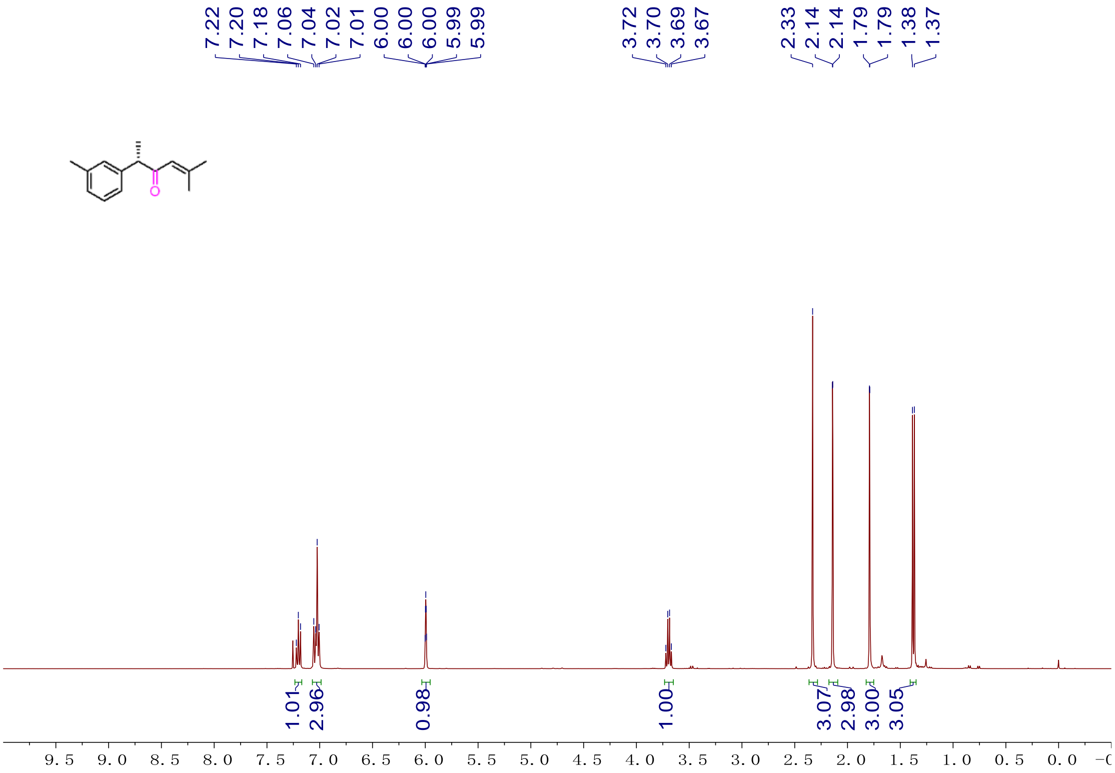

^{13}C NMR (100 MHz, $CDCl_3$) - (3c)

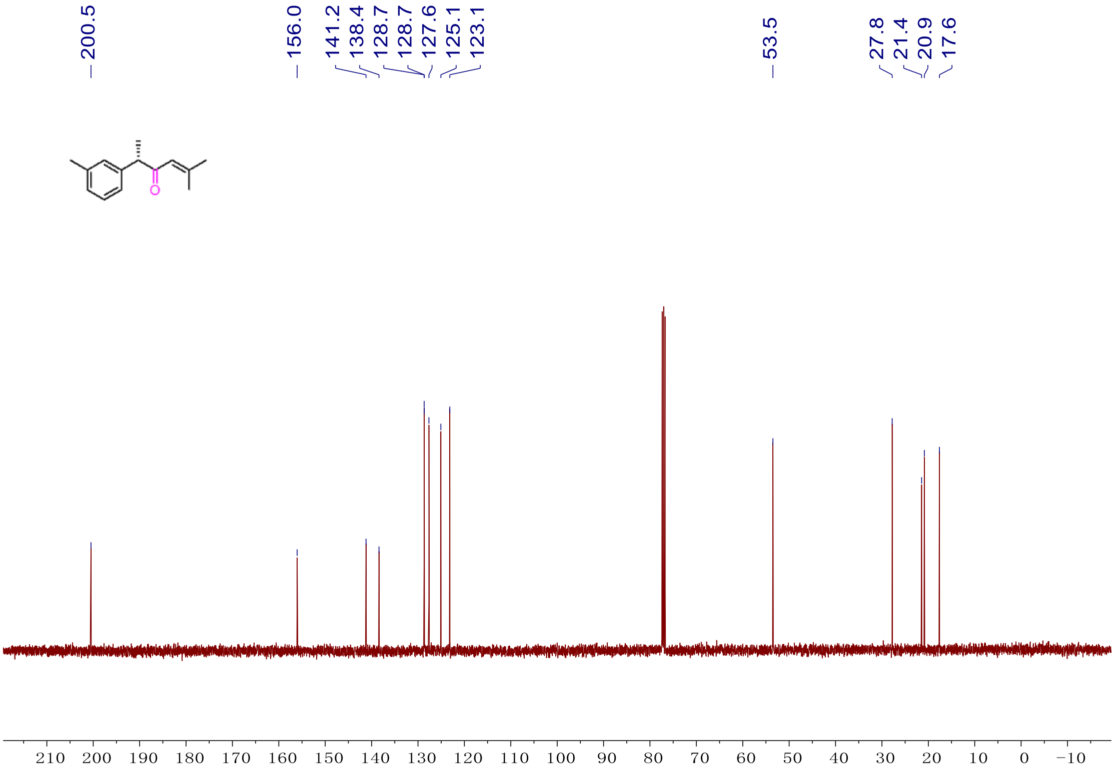

^{1}H NMR (400 MHz, $CDCl_3$) - (3d)

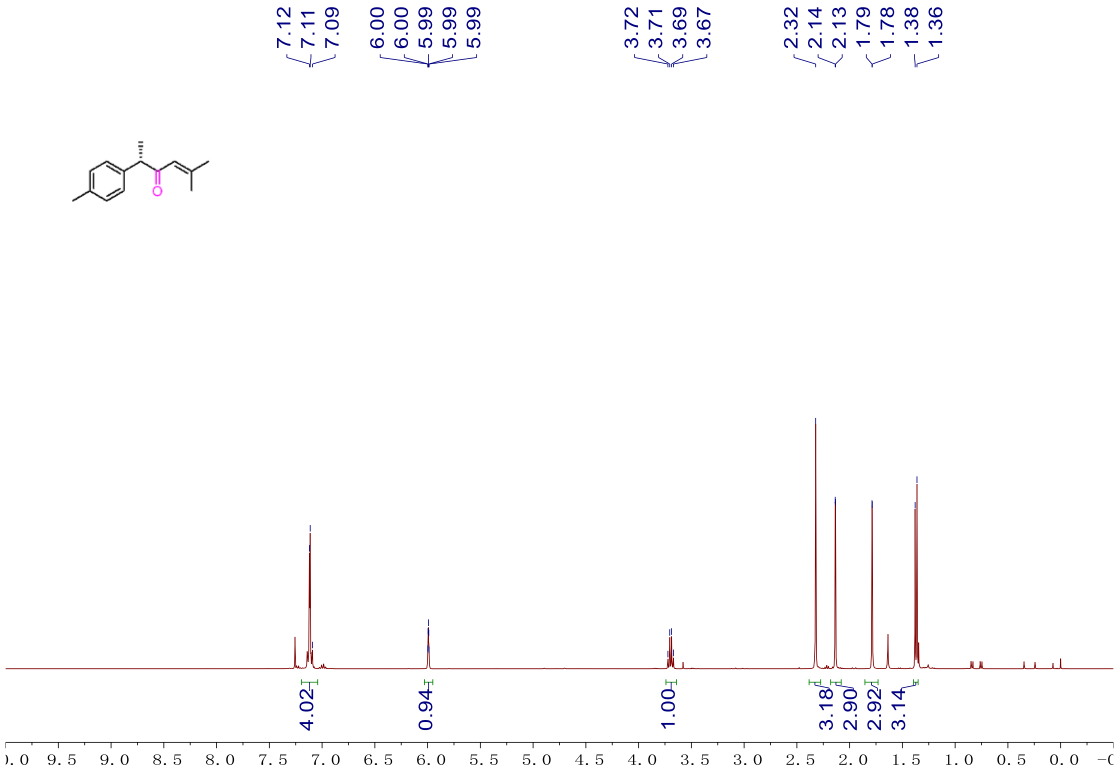

^{13}C NMR (100 MHz, $CDCl_3$) - (3d)

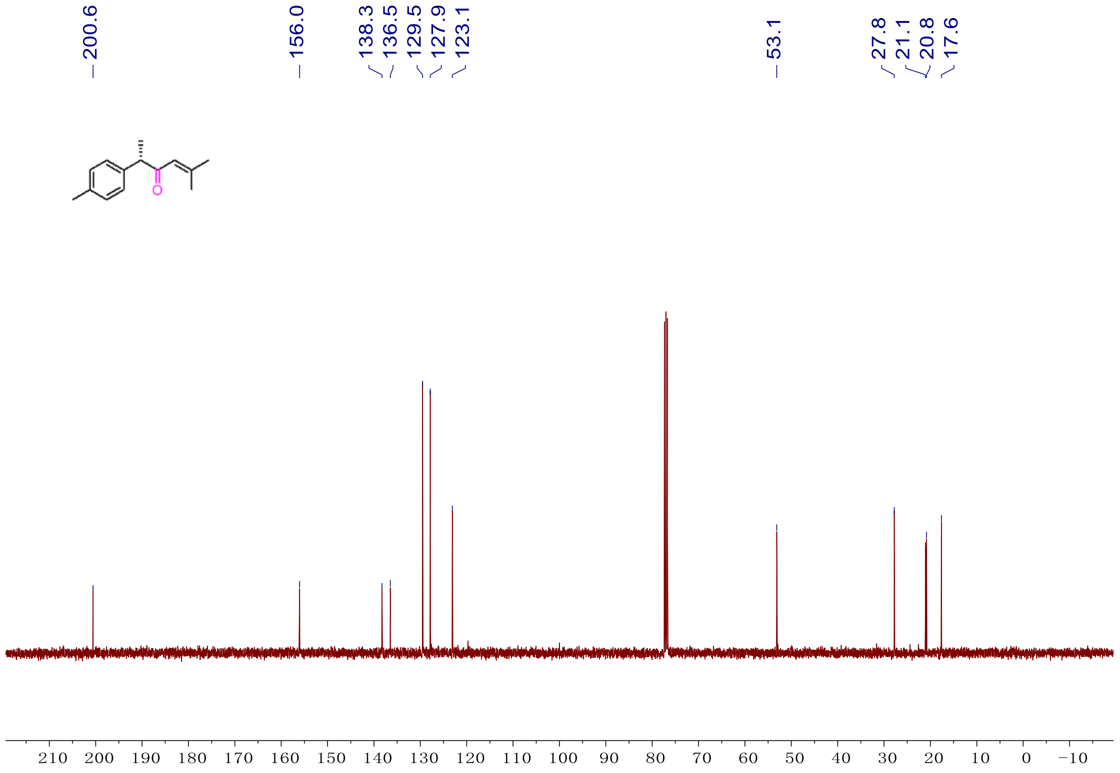

^{1}H NMR (400 MHz, CDCl$_3$) - (3e)

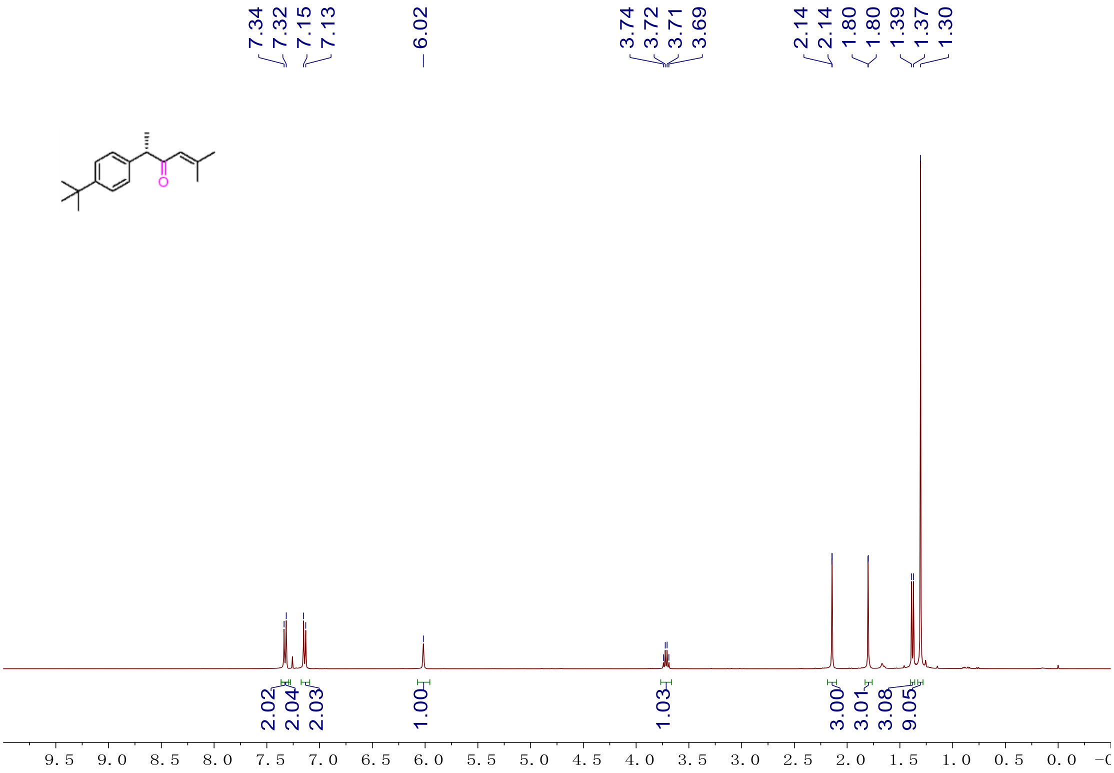

^{13}C NMR (100 MHz, CDCl$_3$) - (3e)

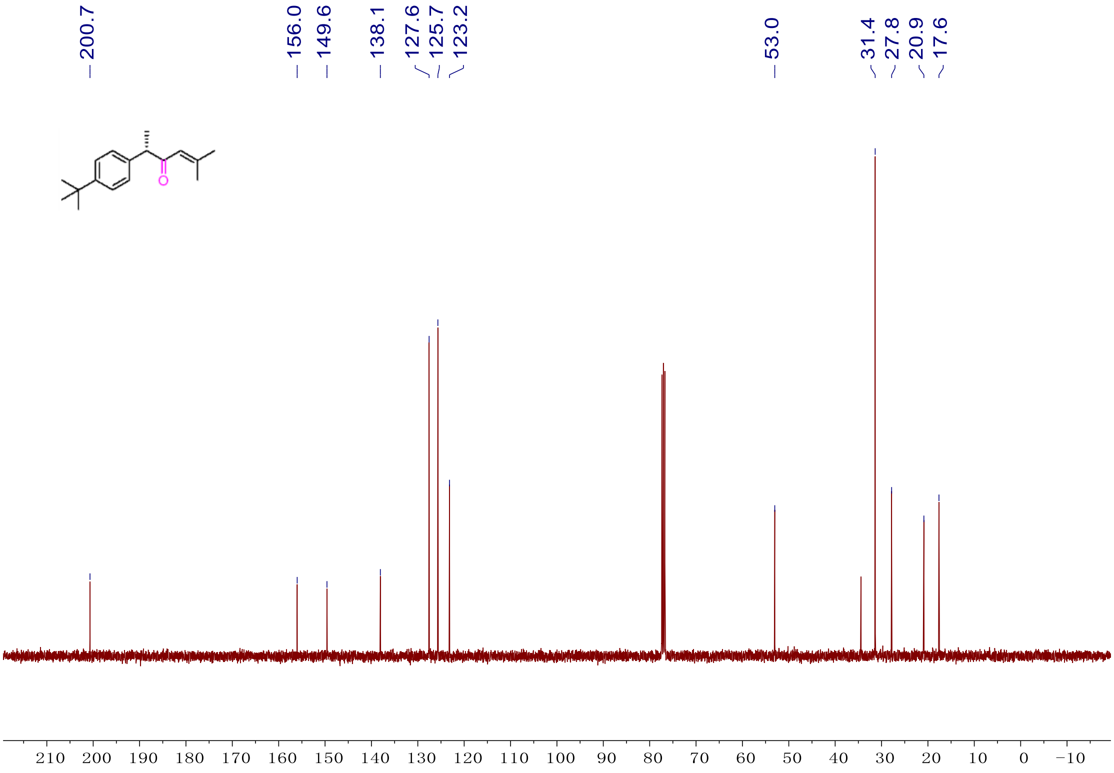

^{1}H NMR (400 MHz, CDCl$_3$) - (3f)

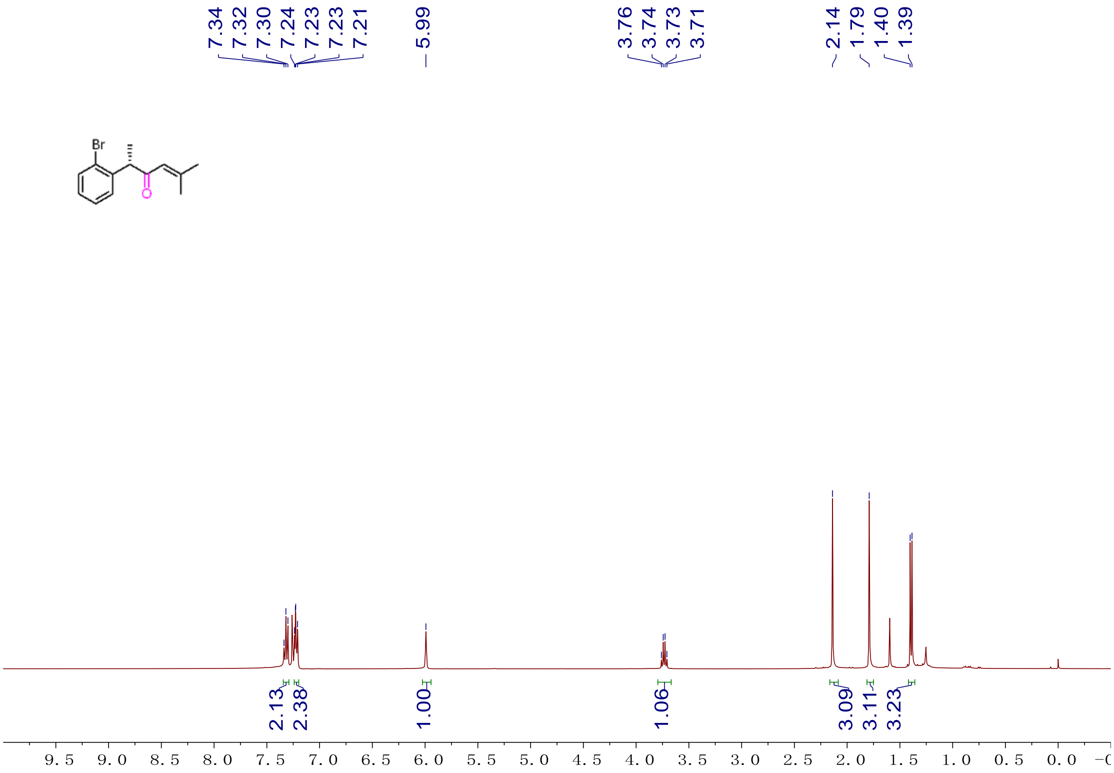

^{13}C NMR (100 MHz, CDCl$_3$) - (3f)

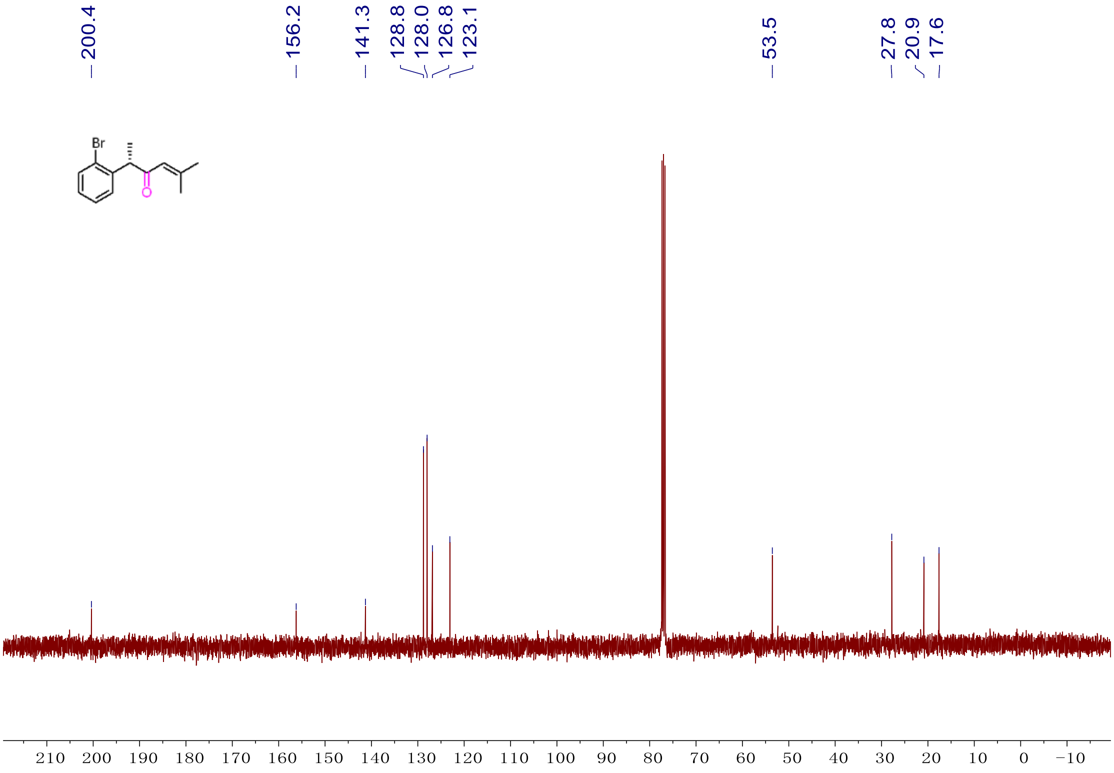

^{1}H NMR (400 MHz, $CDCl_3$) - (3g)

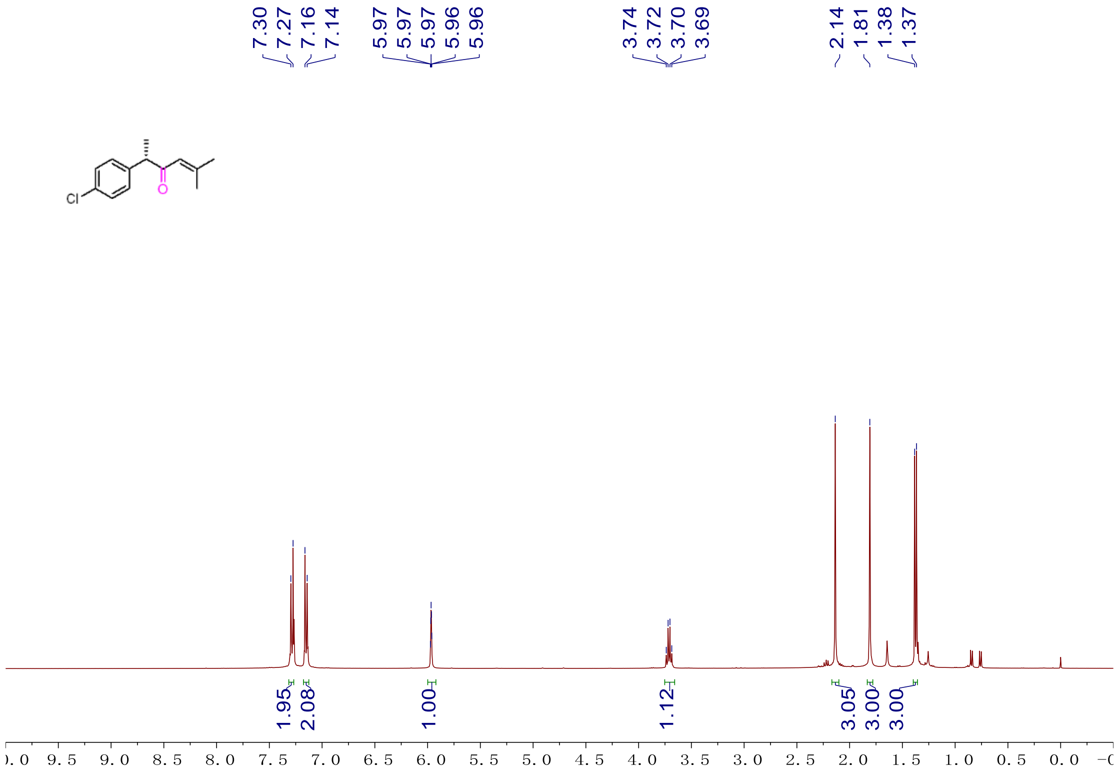

^{13}C NMR (100 MHz, $CDCl_3$) - (3g)

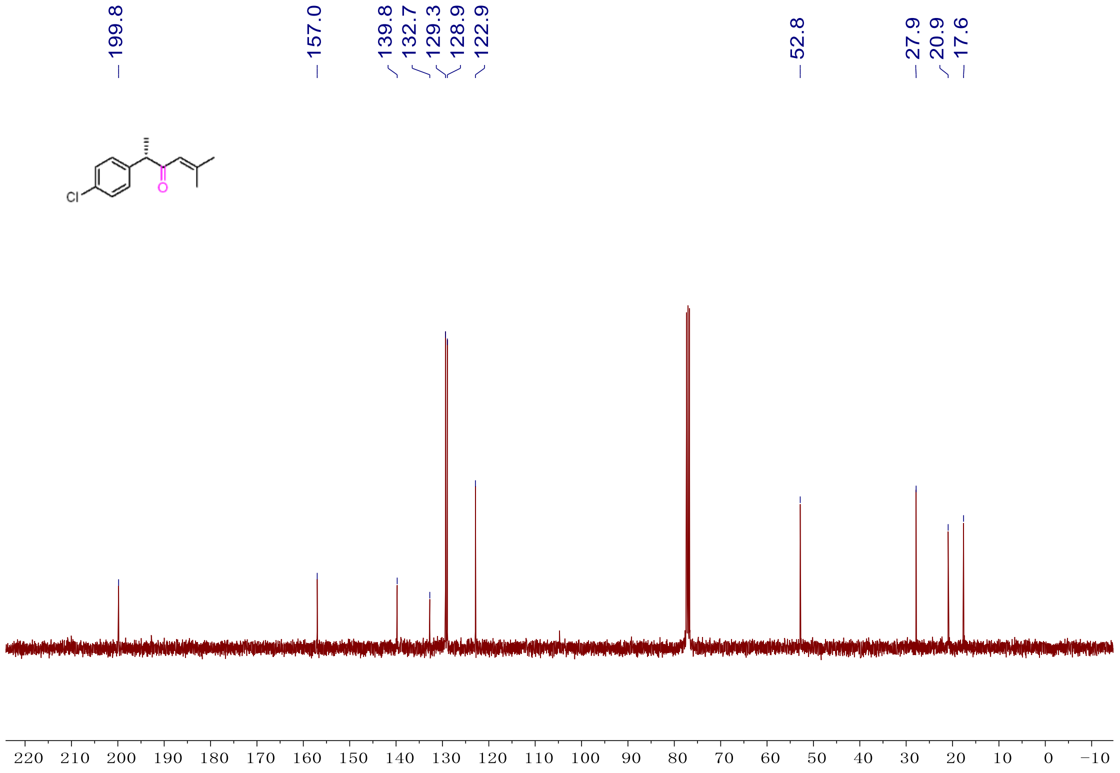

^{1}H NMR (400 MHz, CDCl3) - (3h)

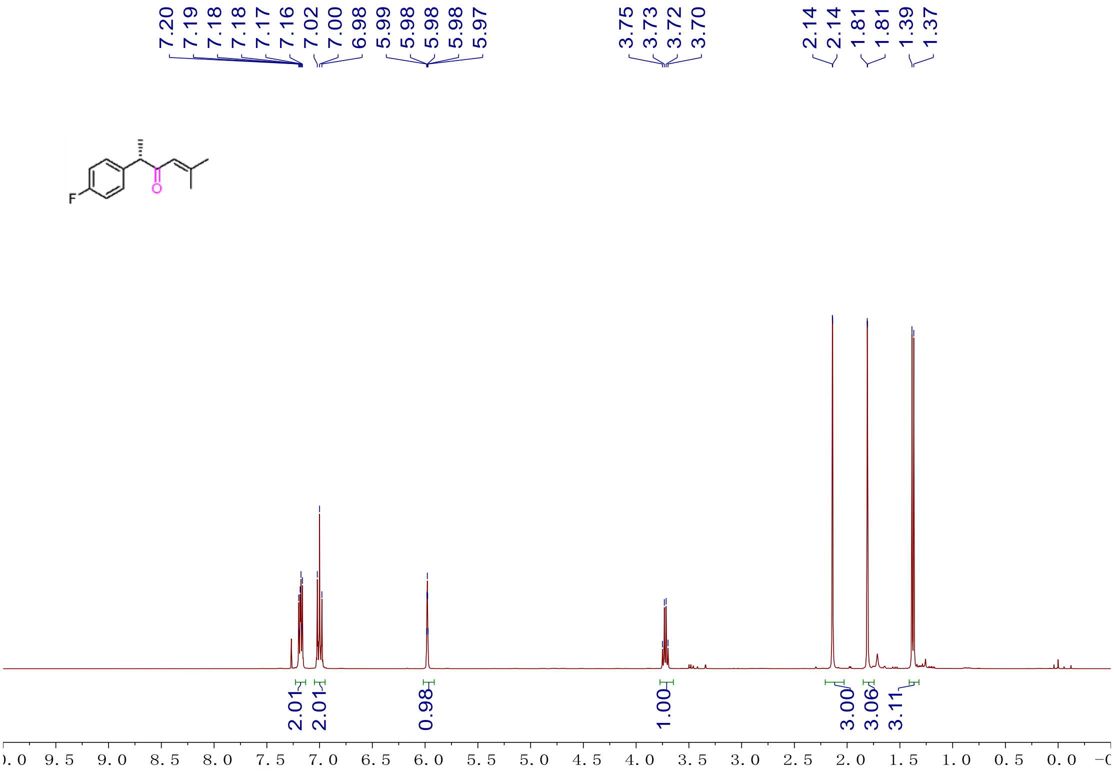

^{13}C NMR (100 MHz, CDCl3) - (3h)

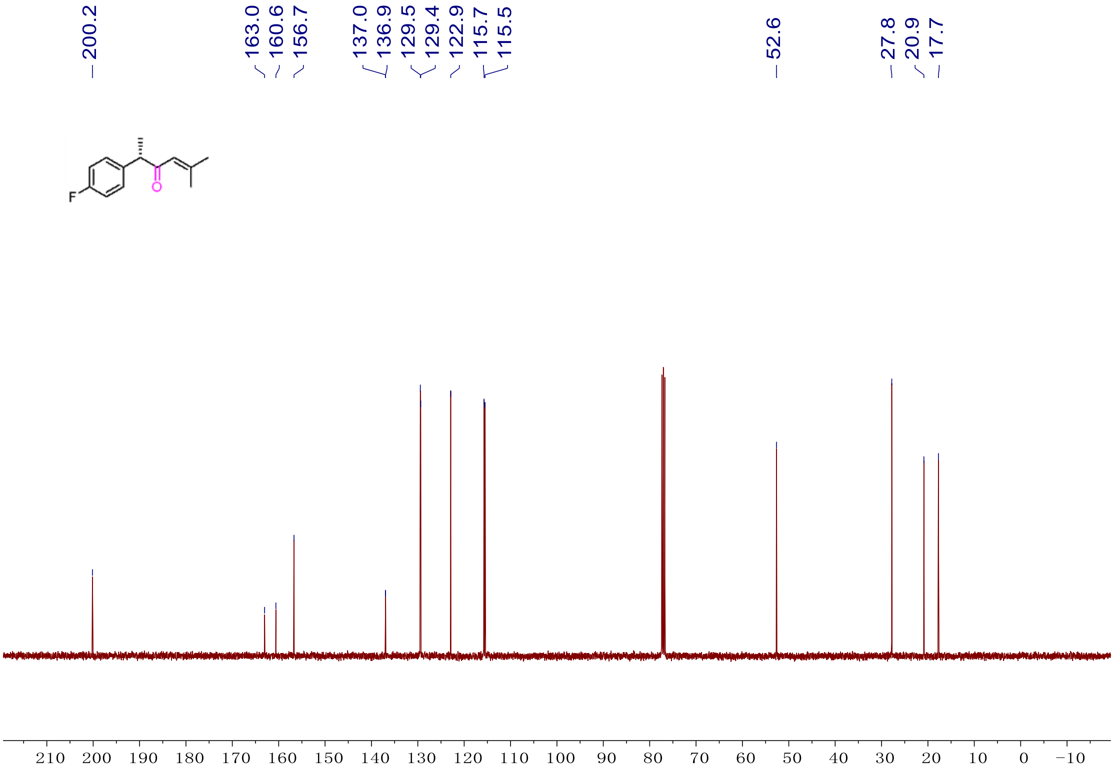

^{19}F NMR (376 MHz, CDCl$_3$) – (3h)

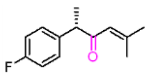

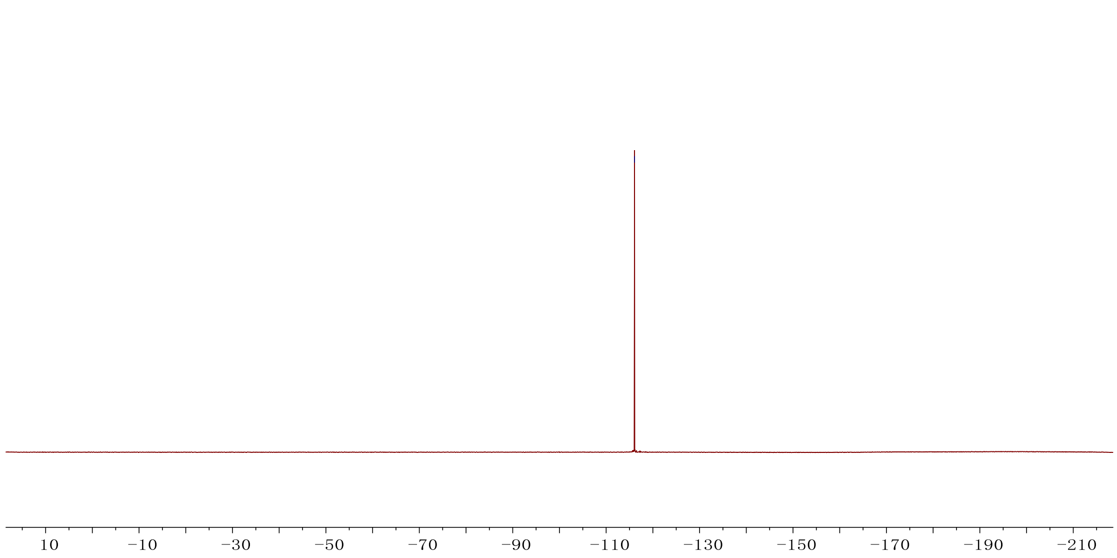

^{1}H NMR (400 MHz, CDCl3) - (3i)

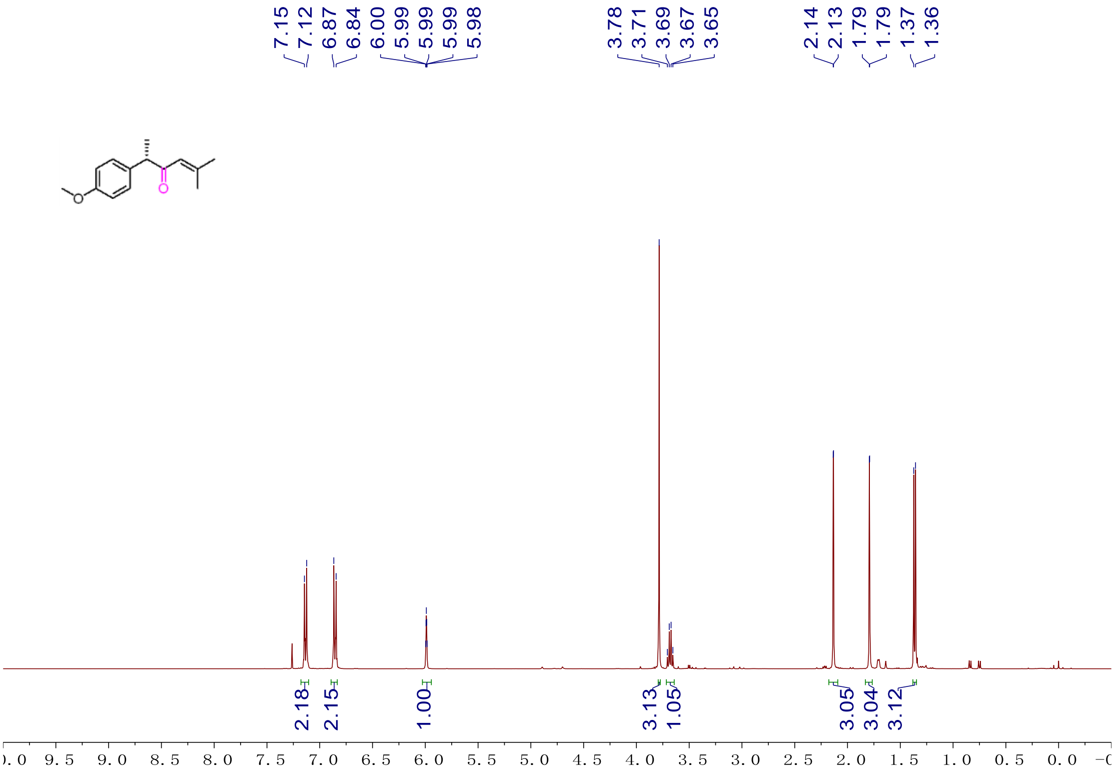

^{13}C NMR (100 MHz, CDCl3) - (3i)

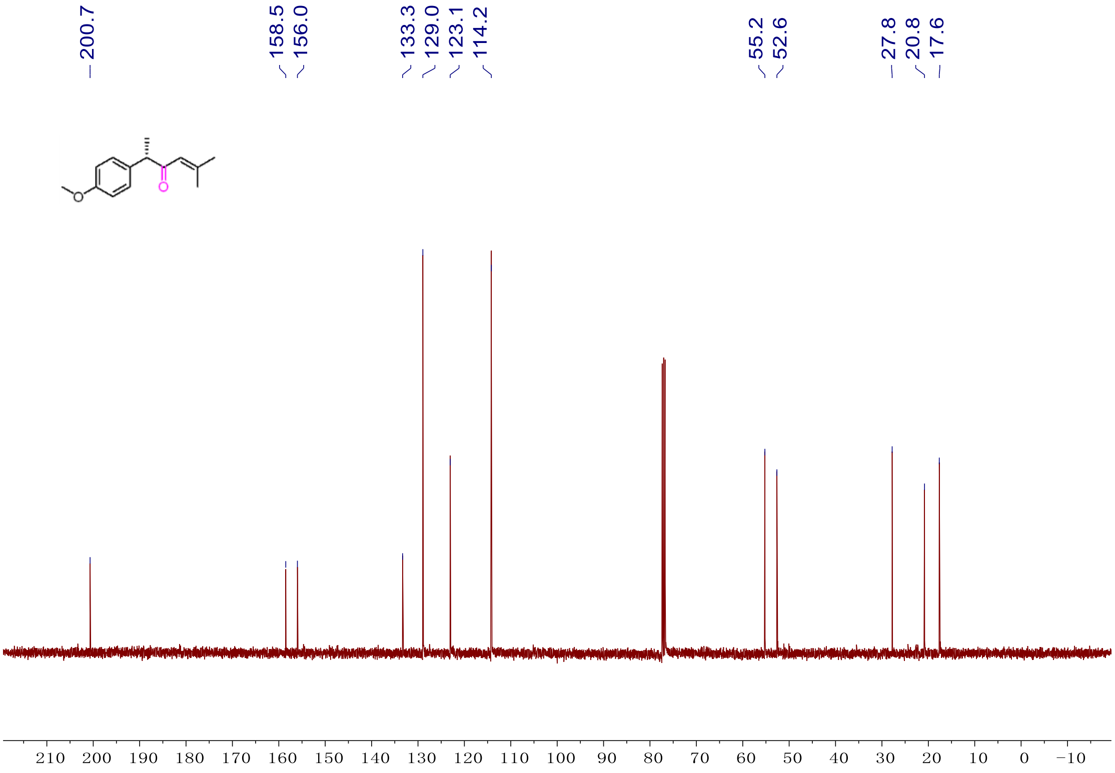

^{1}H NMR (400 MHz, $CDCl_3$) - (3j)

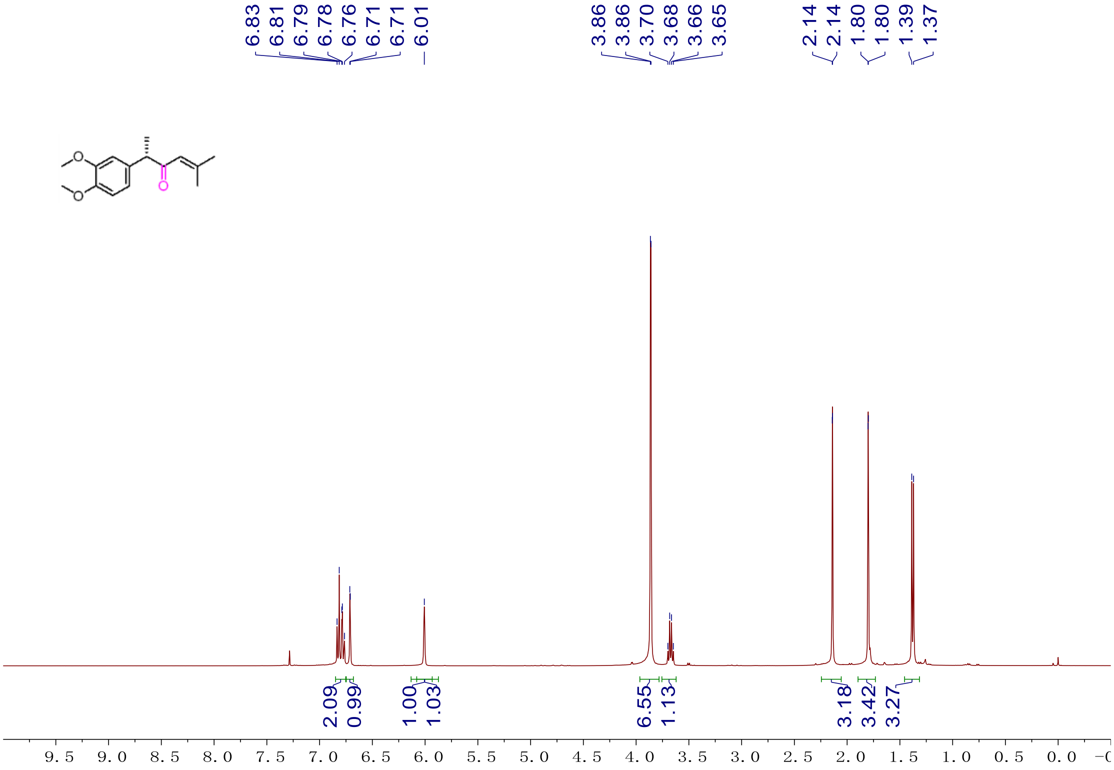

^{13}C NMR (100 MHz, $CDCl_3$) - (3j)

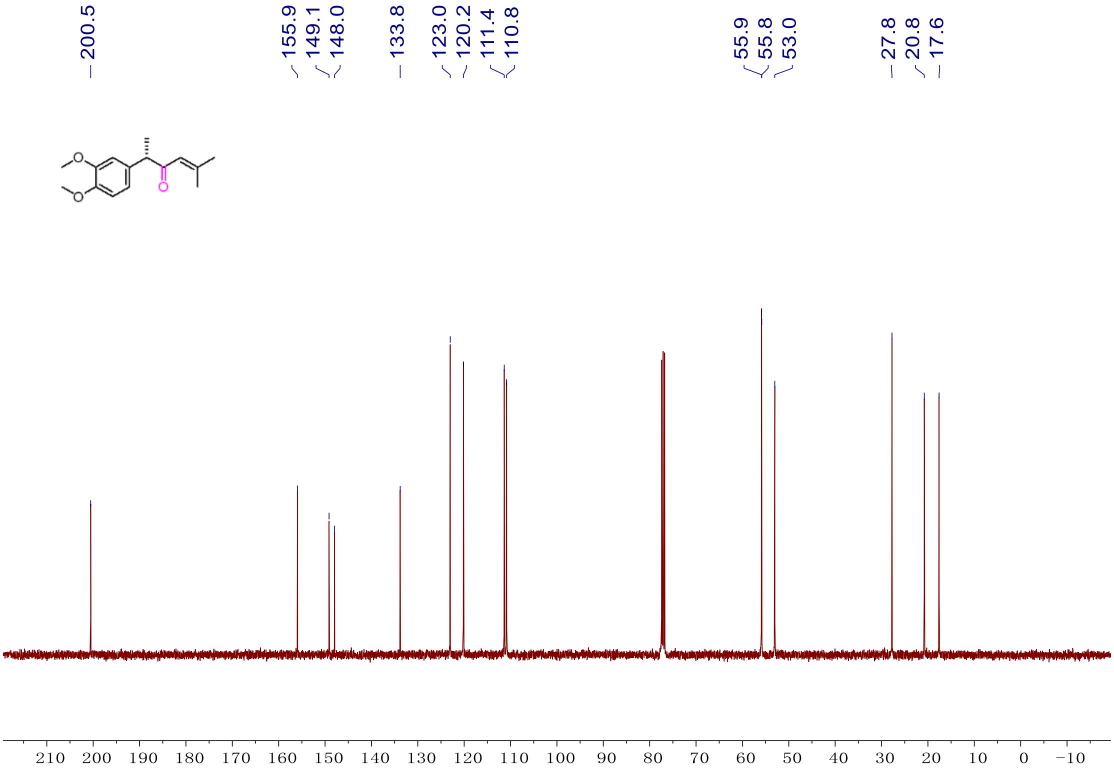

^{1}H NMR (400 MHz, $CDCl_3$) - (3k)

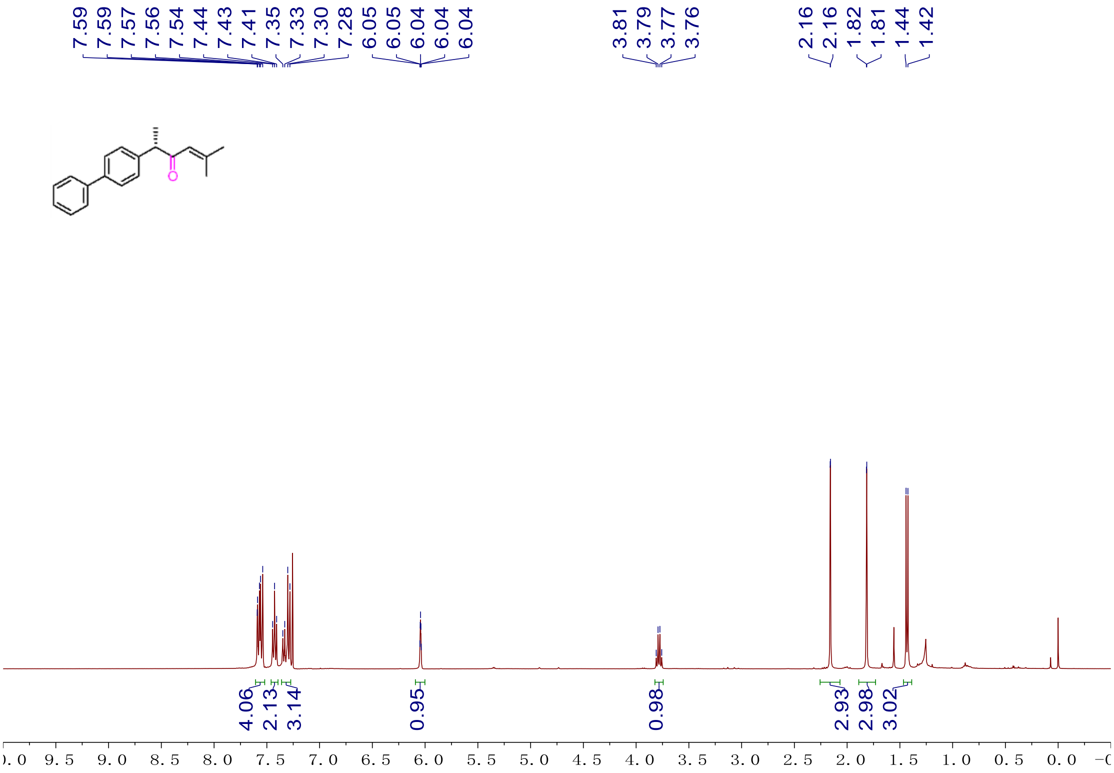

^{13}C NMR (100 MHz, $CDCl_3$) - (3k)

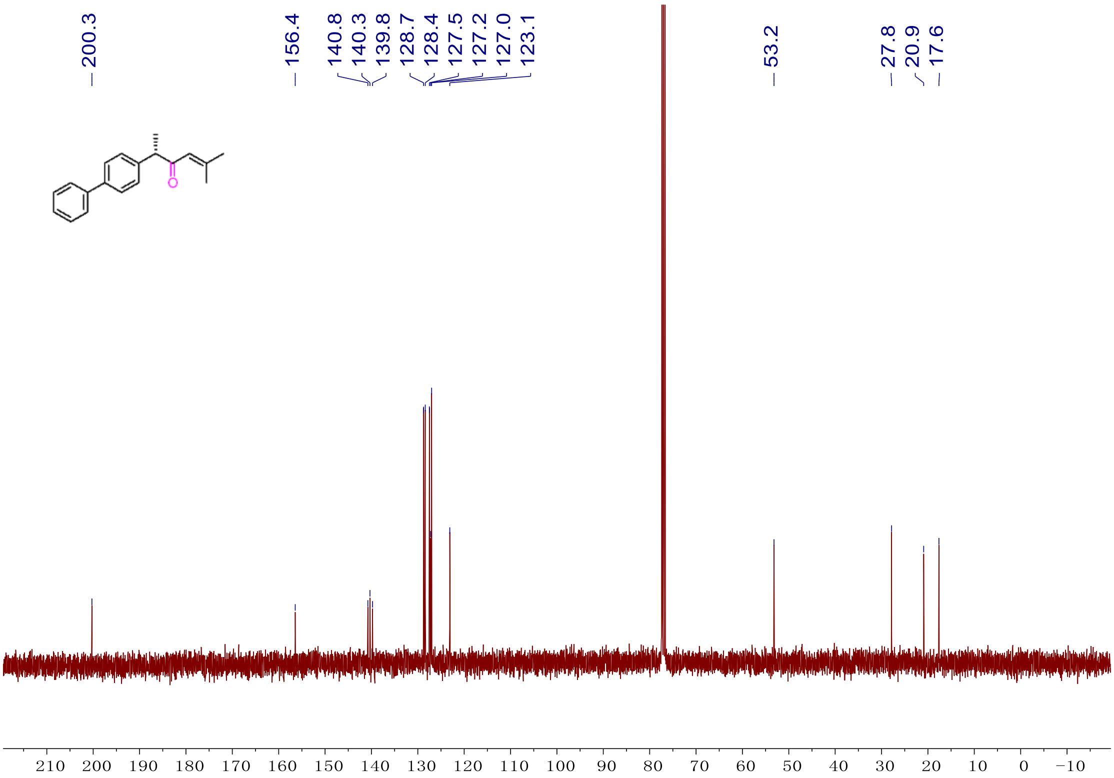

^{1}H NMR (400 MHz, $CDCl_3$) - (3l)

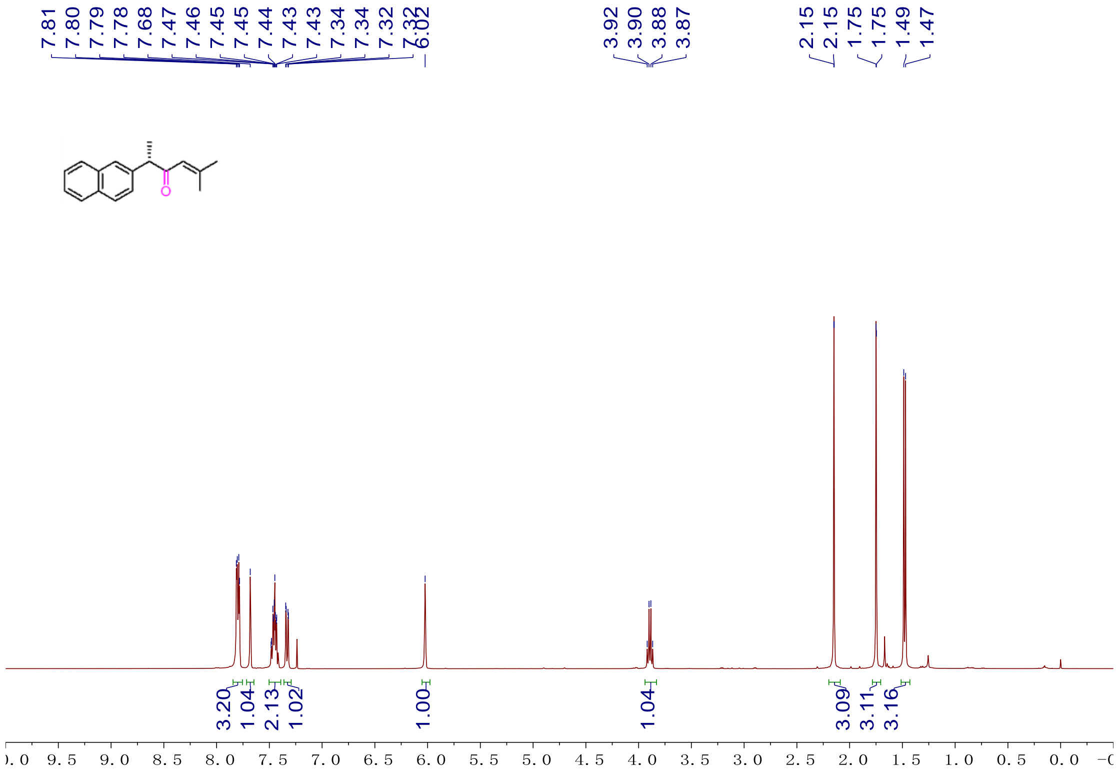

^{13}C NMR (100 MHz, $CDCl_3$) - (3l)

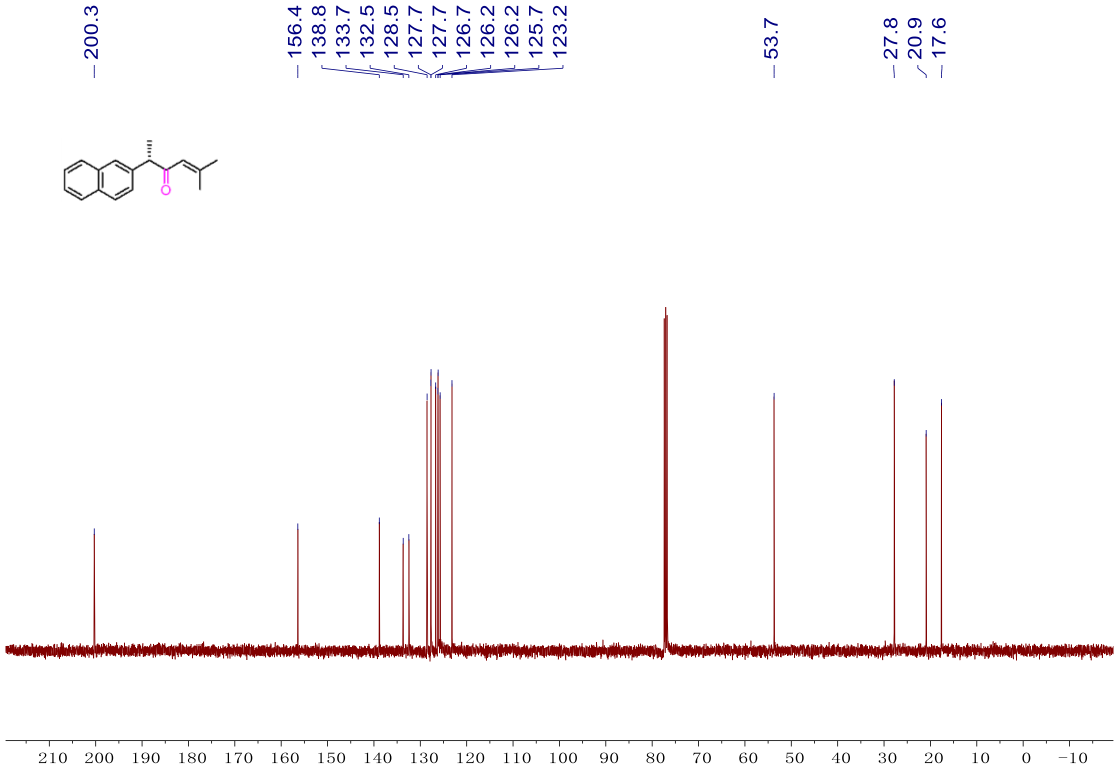

^{1}H NMR (400 MHz, $CDCl_3$) - (3m)

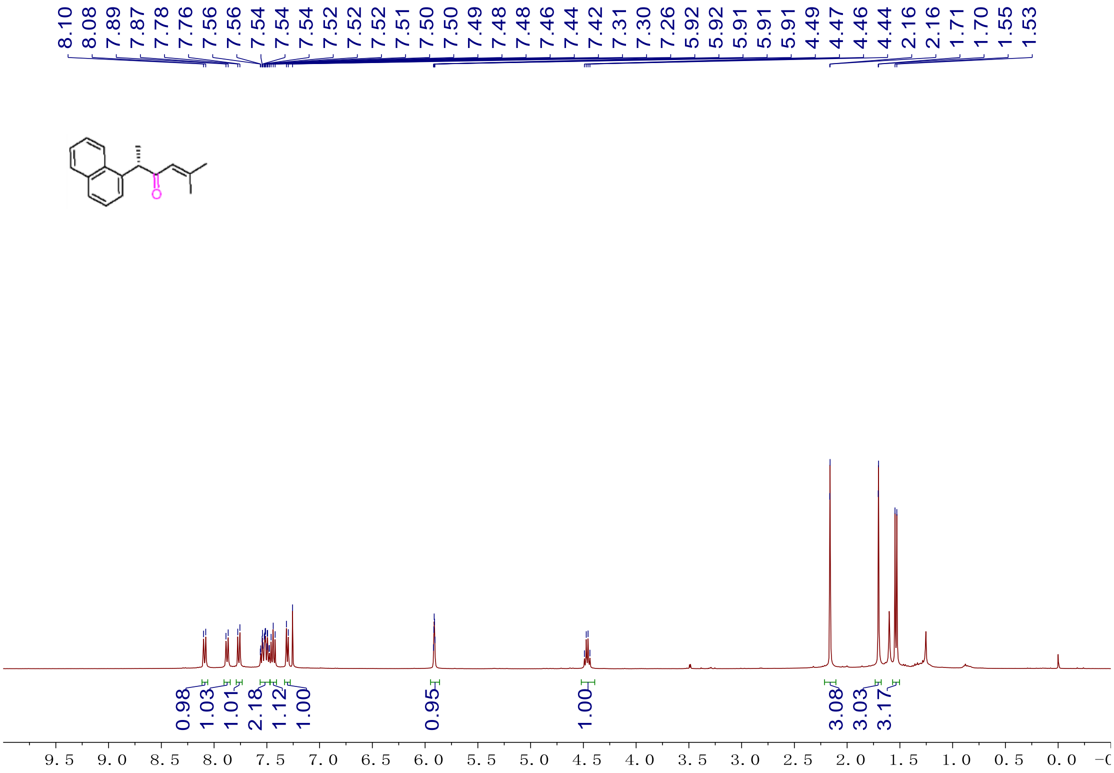

^{13}C NMR (100 MHz, $CDCl_3$) - (3m)

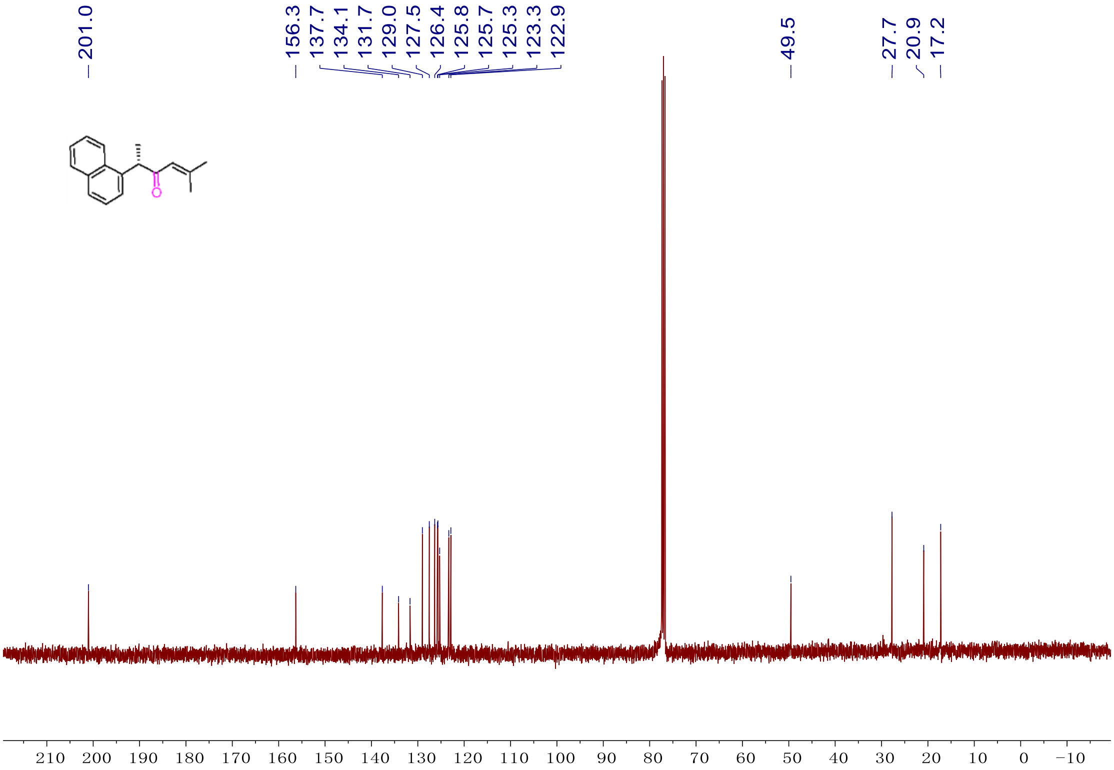

^{1}H NMR (400 MHz, $CDCl_3$) - (3n)

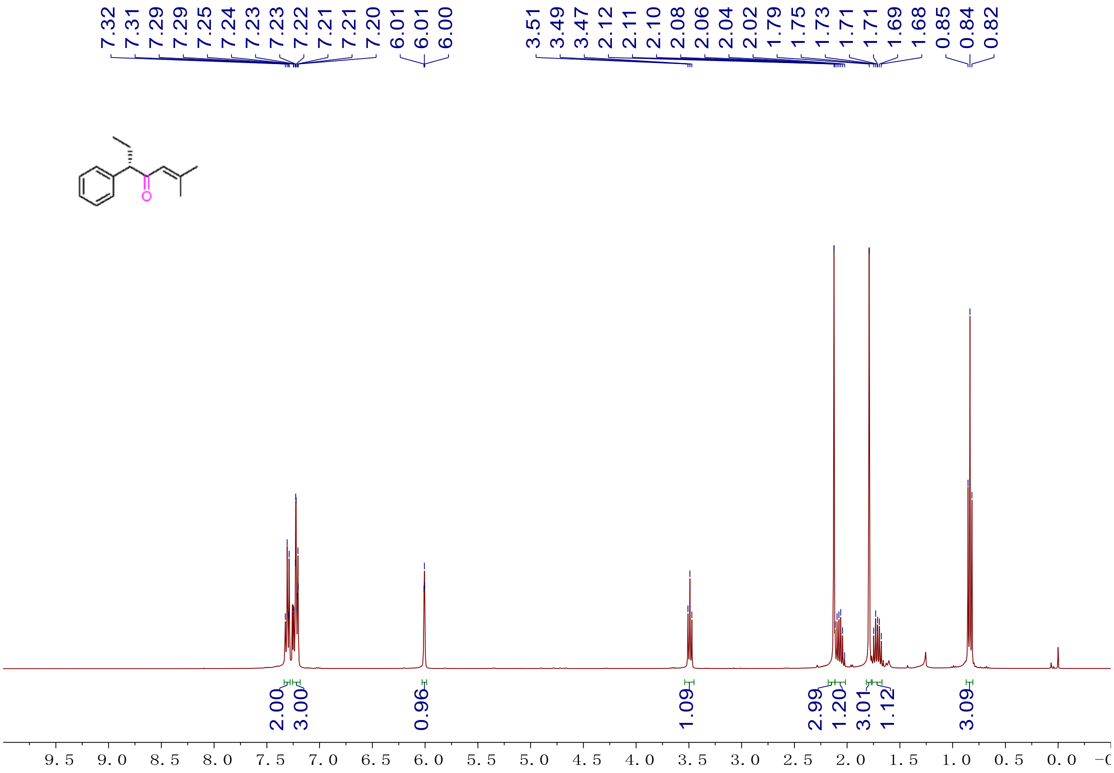

^{13}C NMR (100 MHz, $CDCl_3$) - (3n)

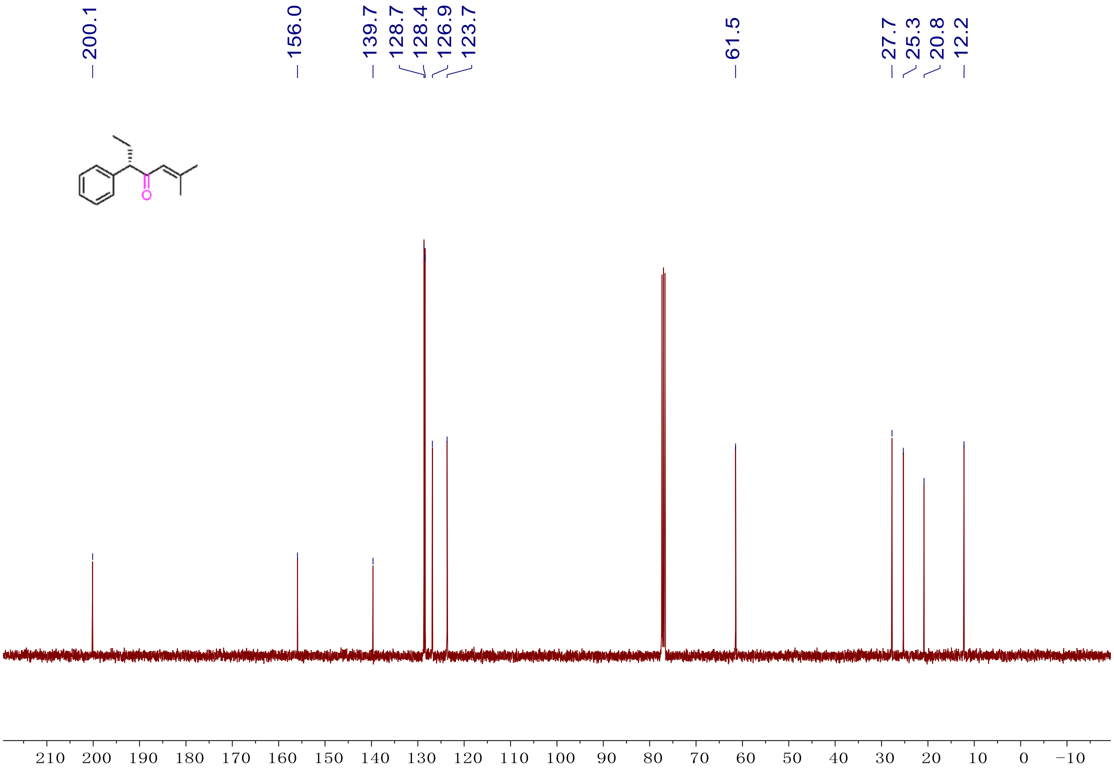

^{1}H NMR (400 MHz, CDCl$_3$) - (3o)

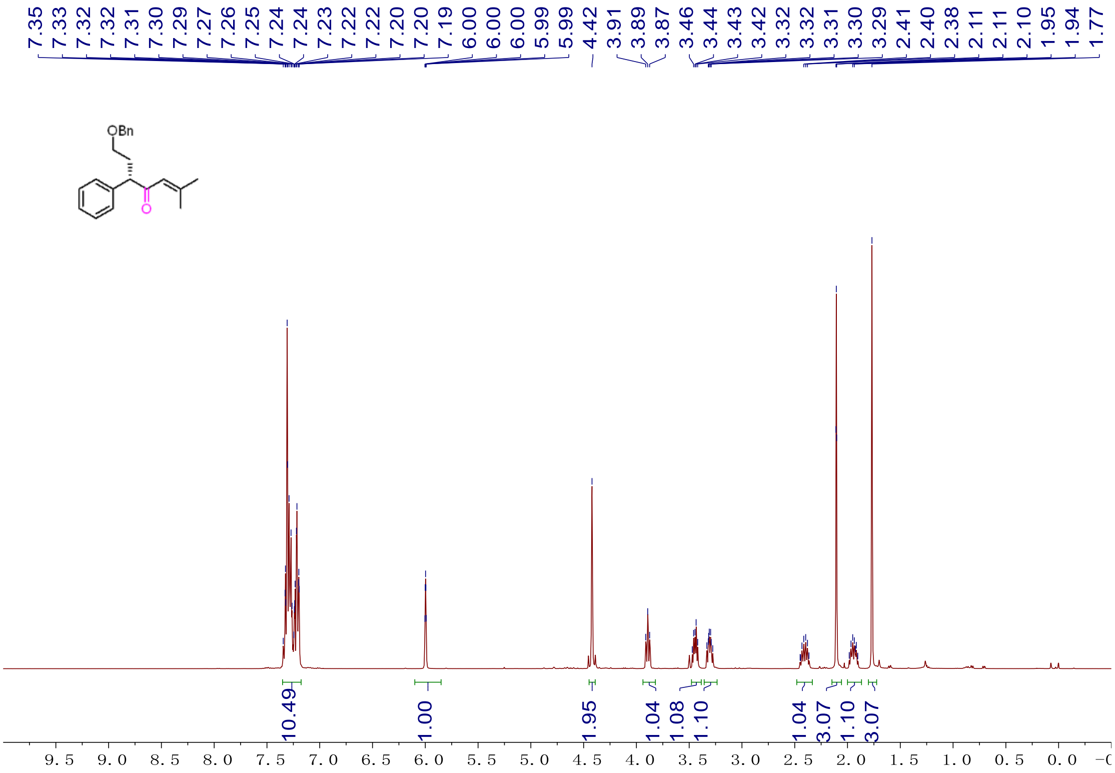

^{13}C NMR (100 MHz, CDCl$_3$) - (3o)

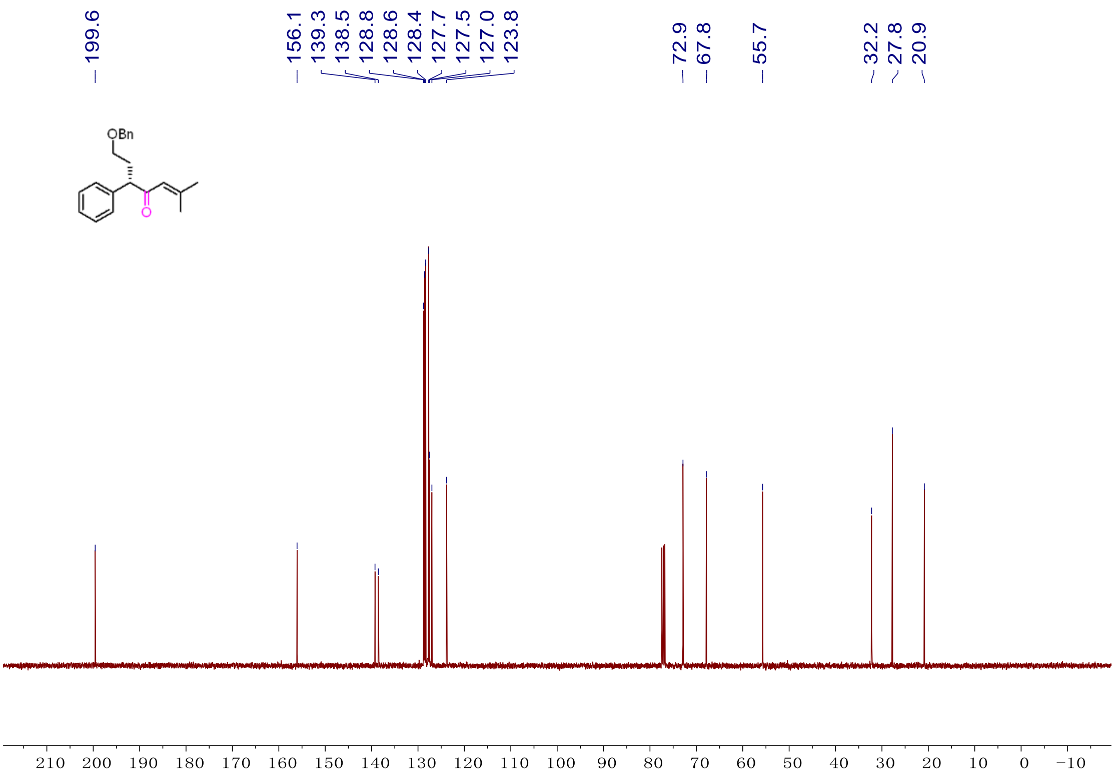

^{1}H NMR (400 MHz, CDCl$_3$) - (3p)

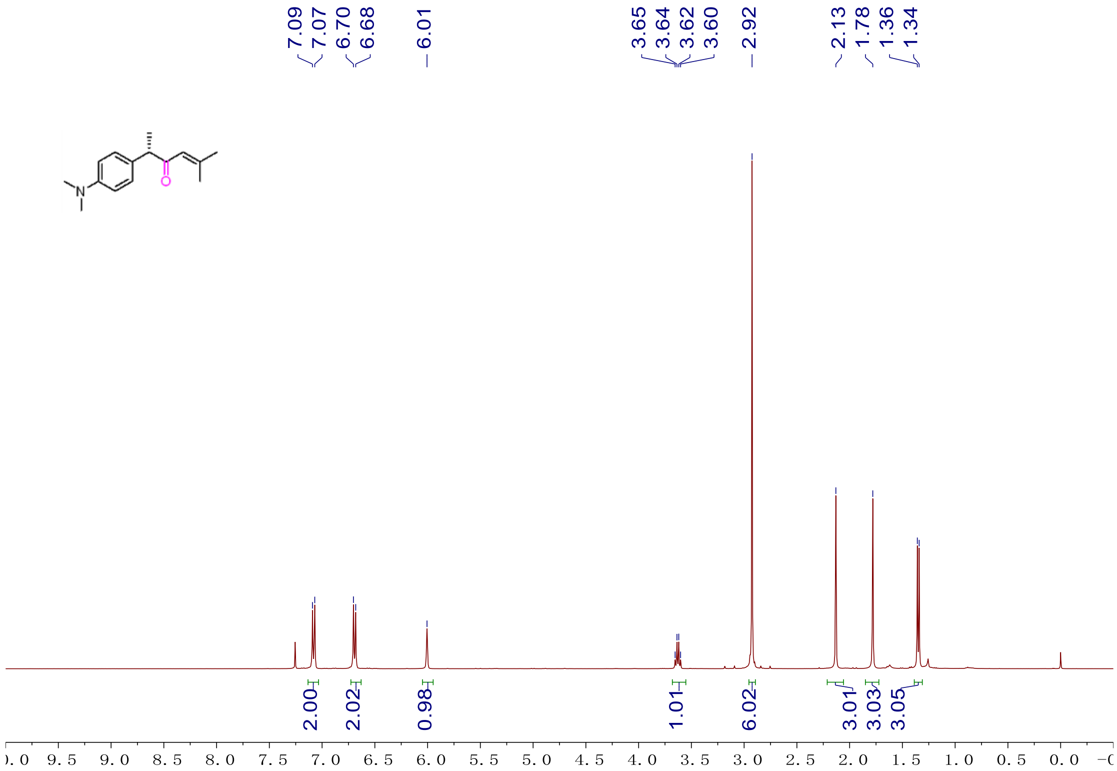

^{13}C NMR (100 MHz, CDCl$_3$) - (3p)

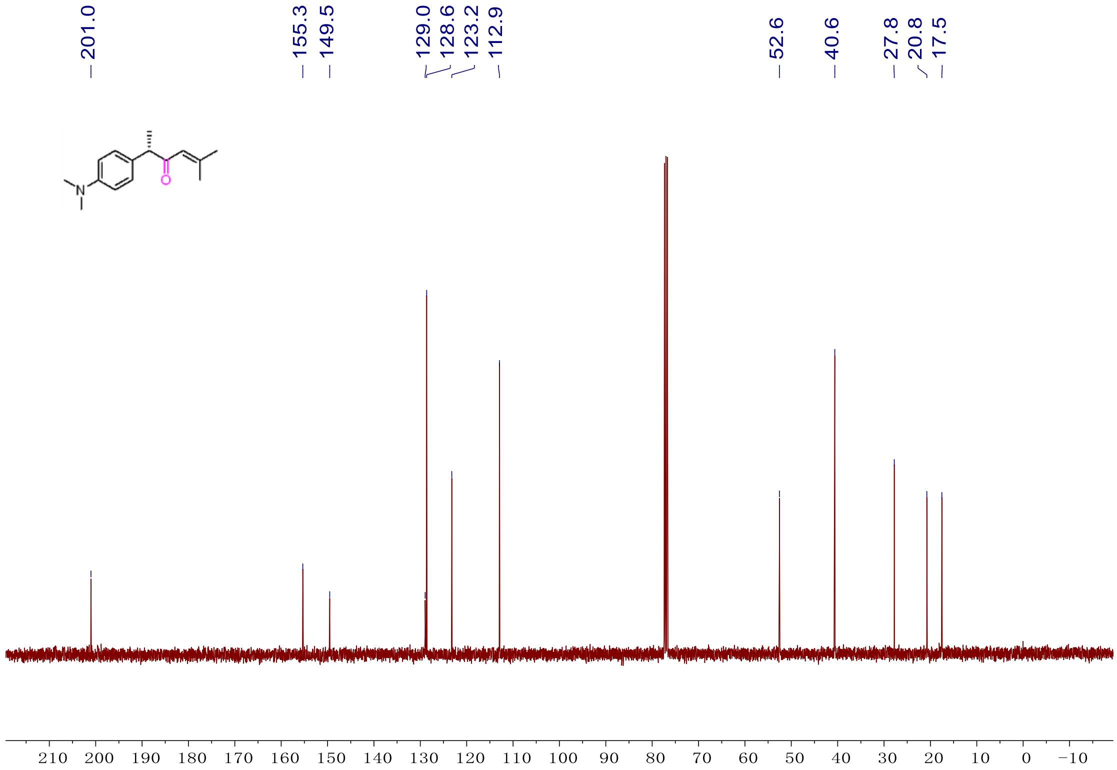

^{1}H NMR (400 MHz, $CDCl_3$) - (3q)

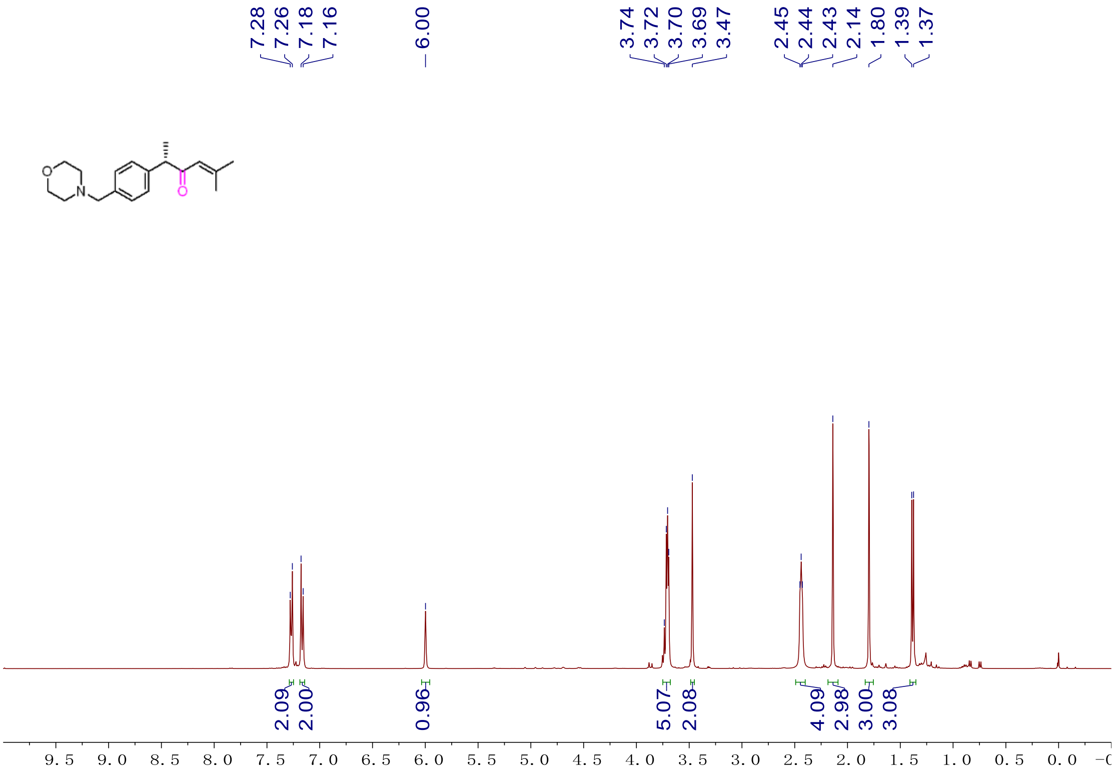

^{13}C NMR (100 MHz, $CDCl_3$) - (3q)

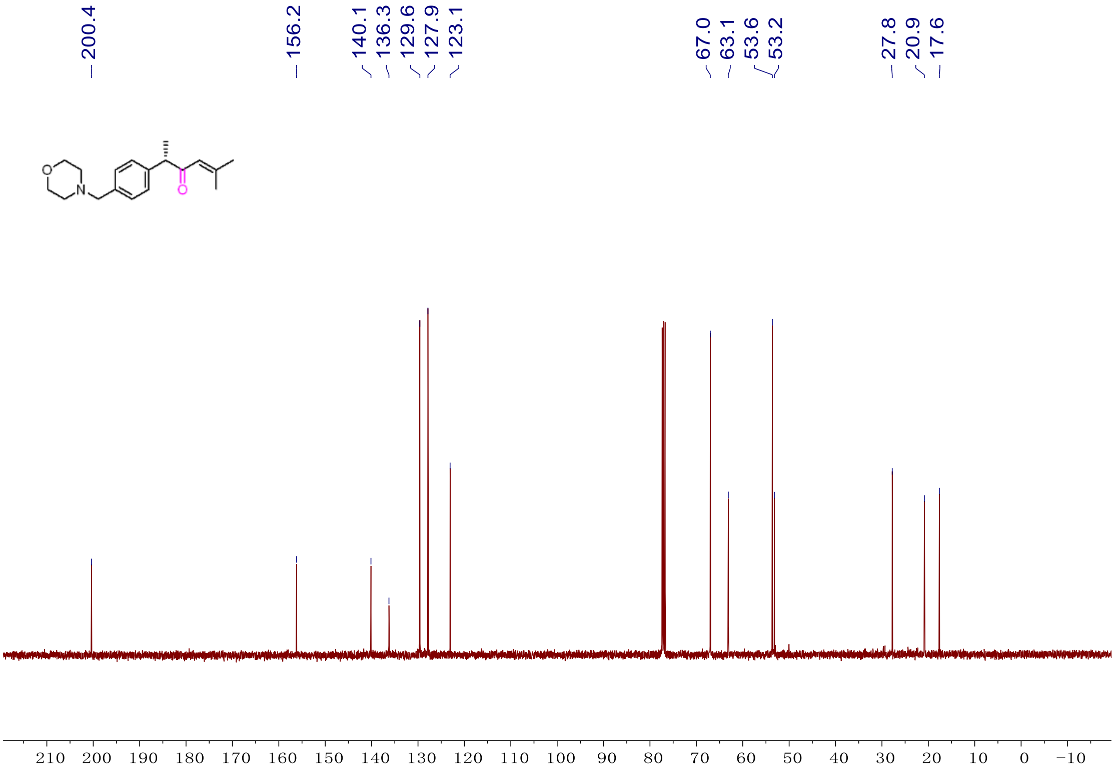

^{1}H NMR (400 MHz, $CDCl_3$) - (3r)

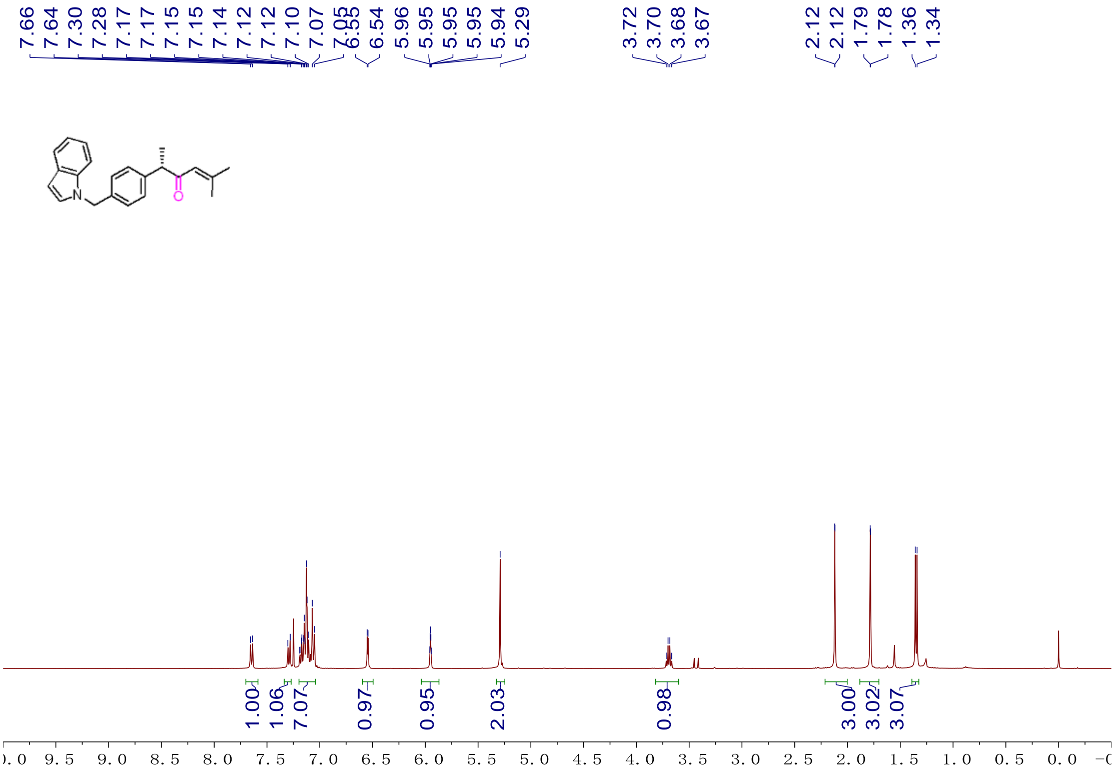

^{13}C NMR (100 MHz, $CDCl_3$) - (3r)

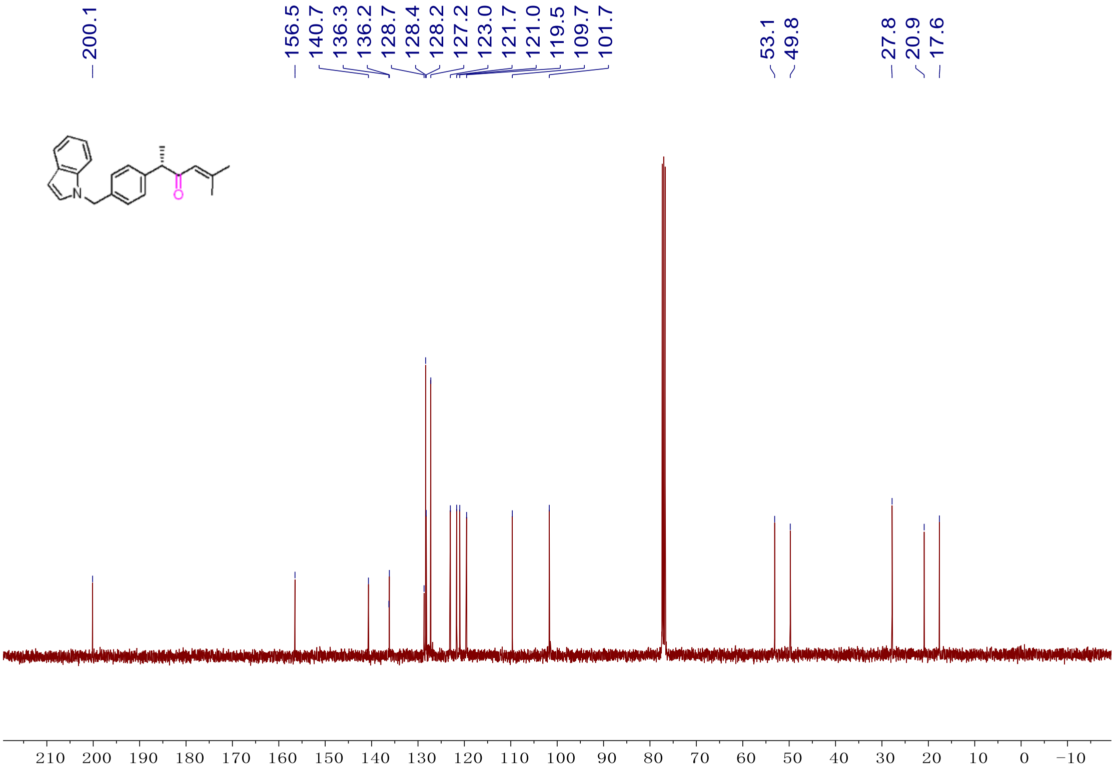

^{1}H NMR (400 MHz, CDCl3) - (3s)

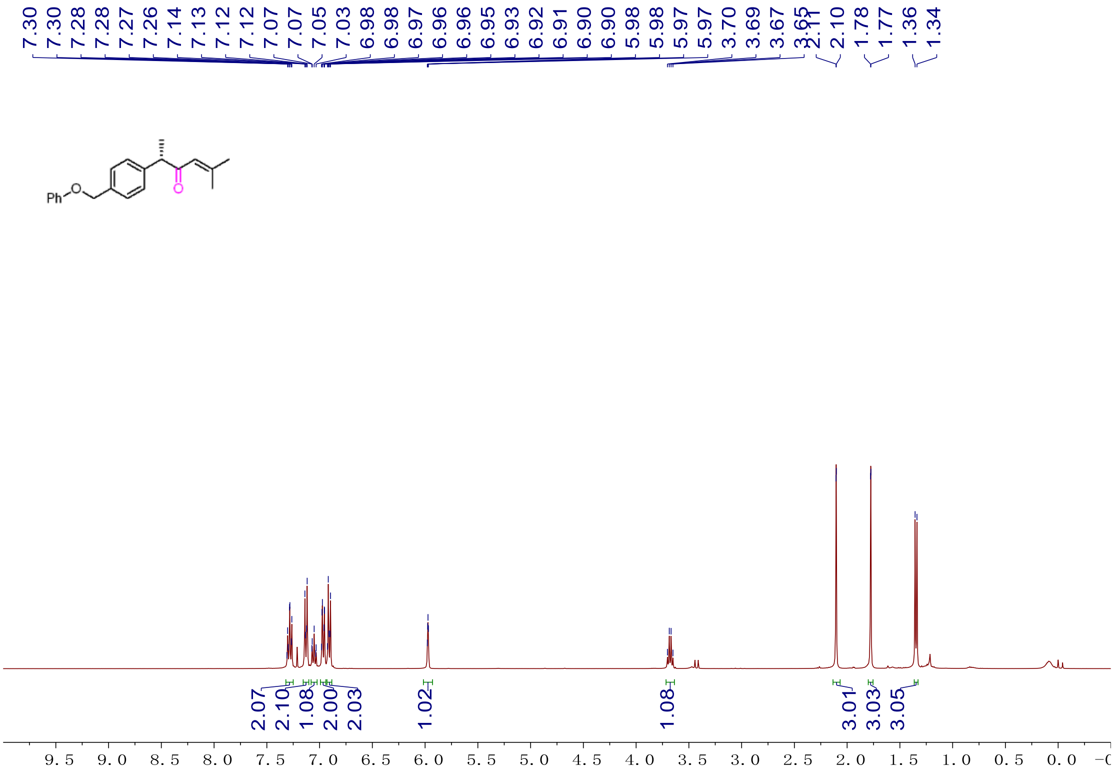

^{13}C NMR (100 MHz, CDCl3) - (3s)

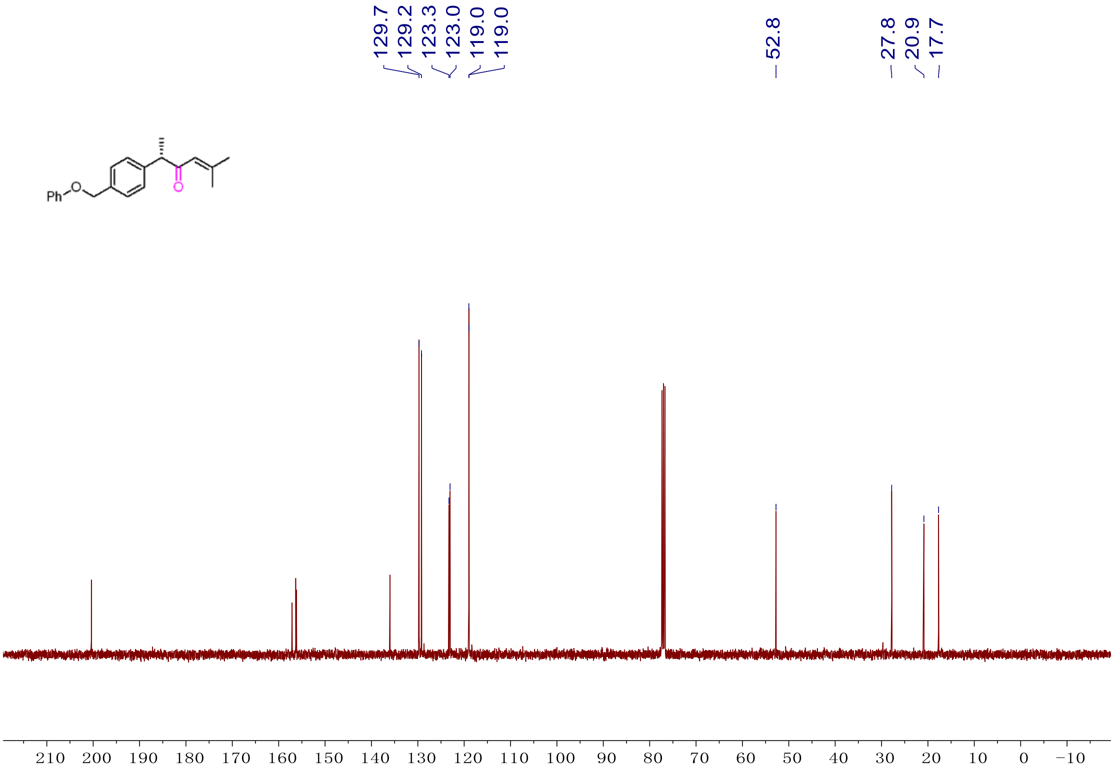

^{1}H NMR (400 MHz, CDCl$_3$) - (3t)

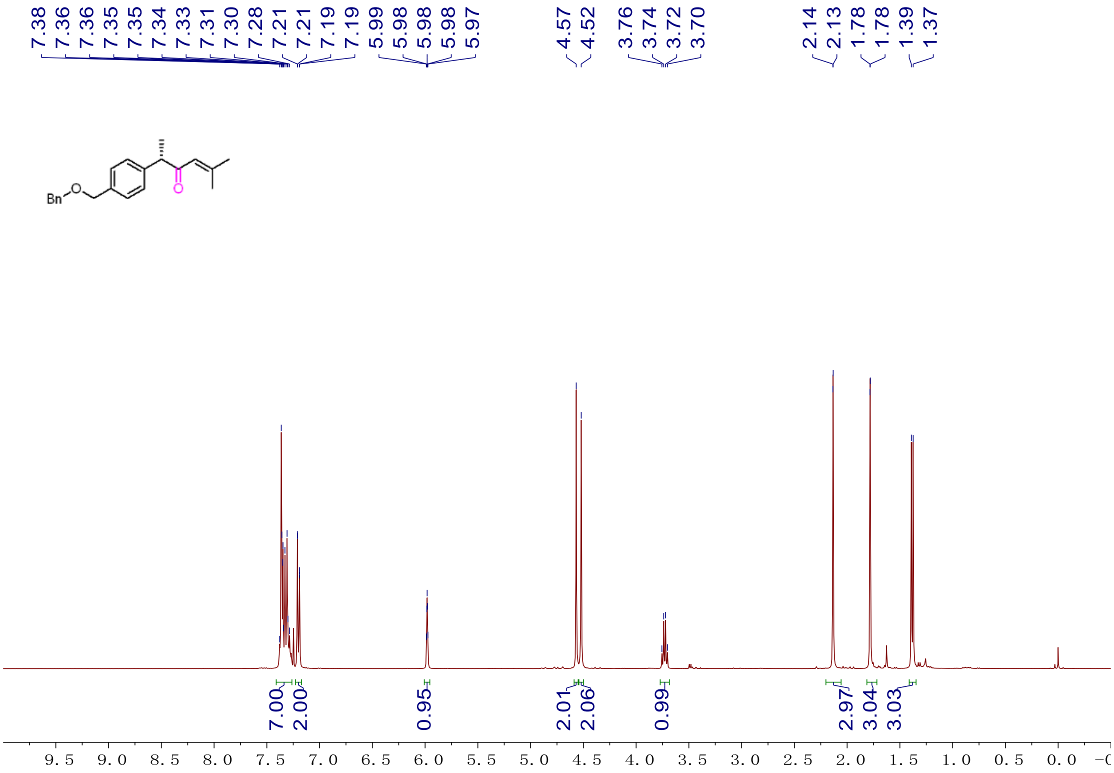

^{13}C NMR (100 MHz, CDCl$_3$) - (3t)

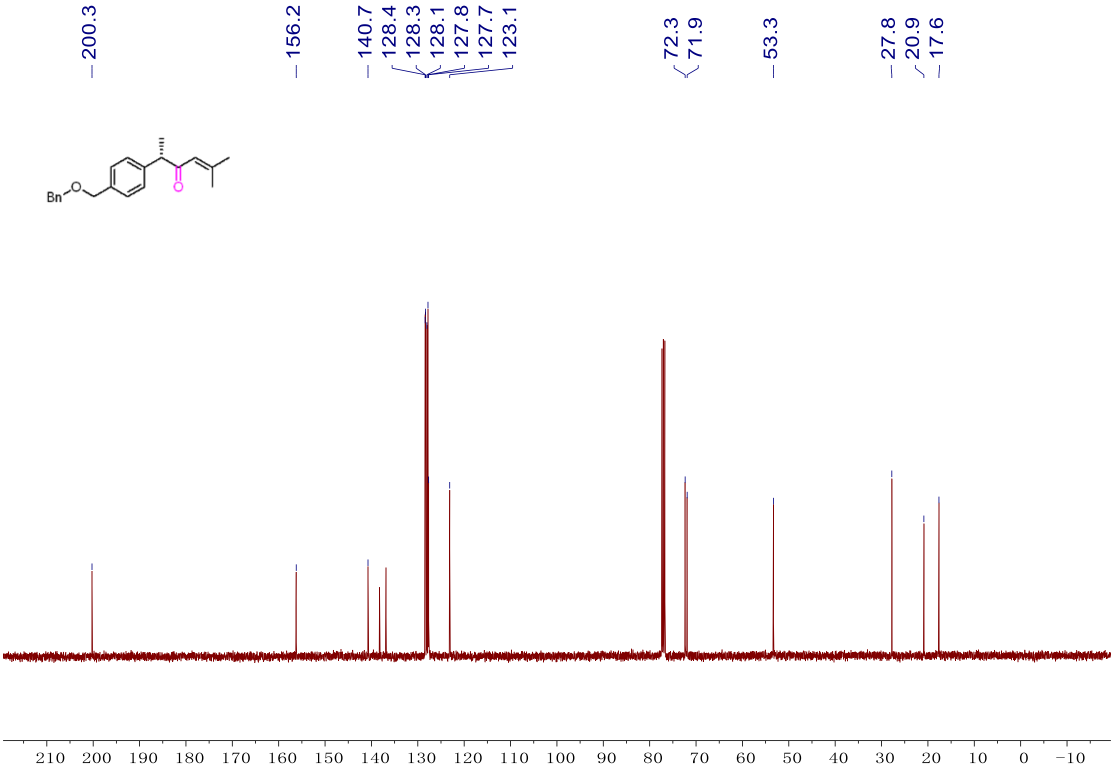

^{1}H NMR (400 MHz, $CDCl_3$) - (3u)

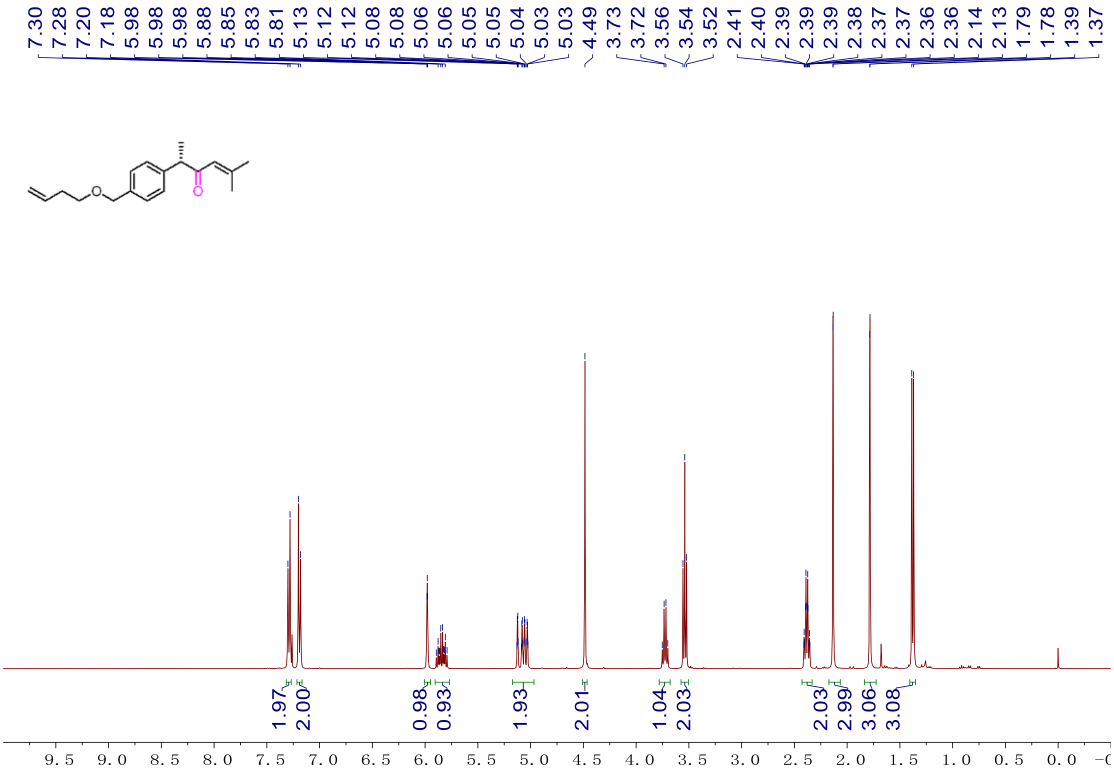

^{13}C NMR (100 MHz, $CDCl_3$) - (3u)

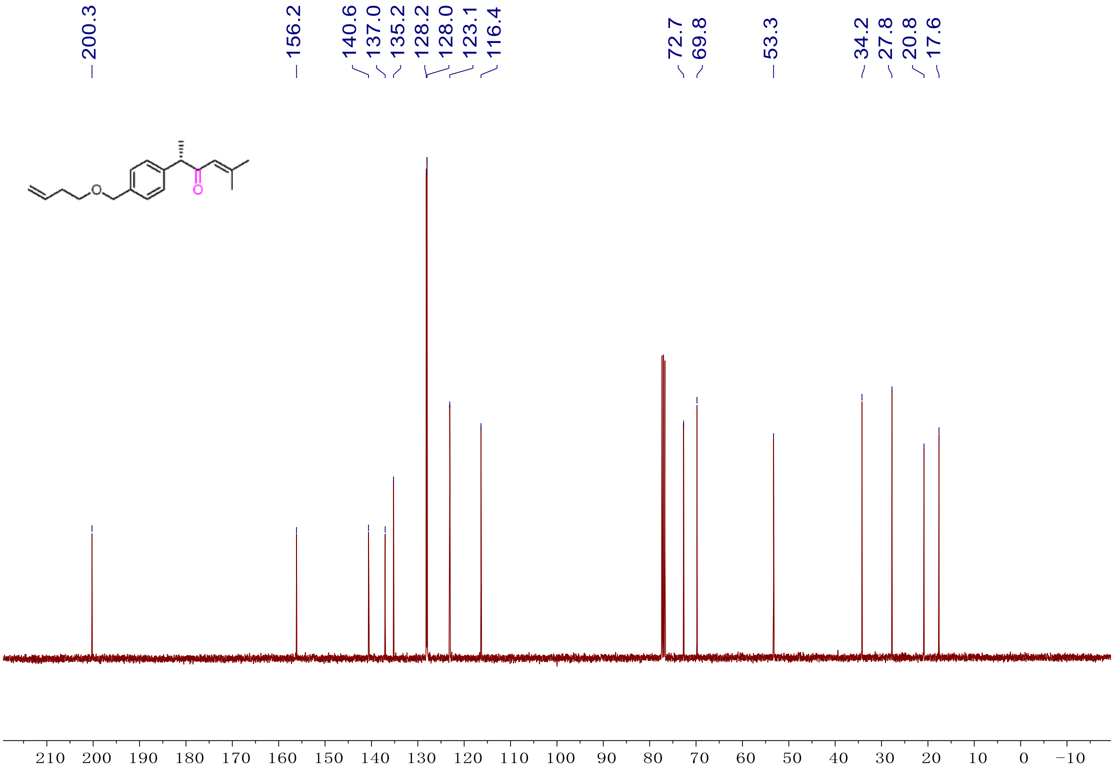

^{1}H NMR (400 MHz, $CDCl_3$) - (3v)

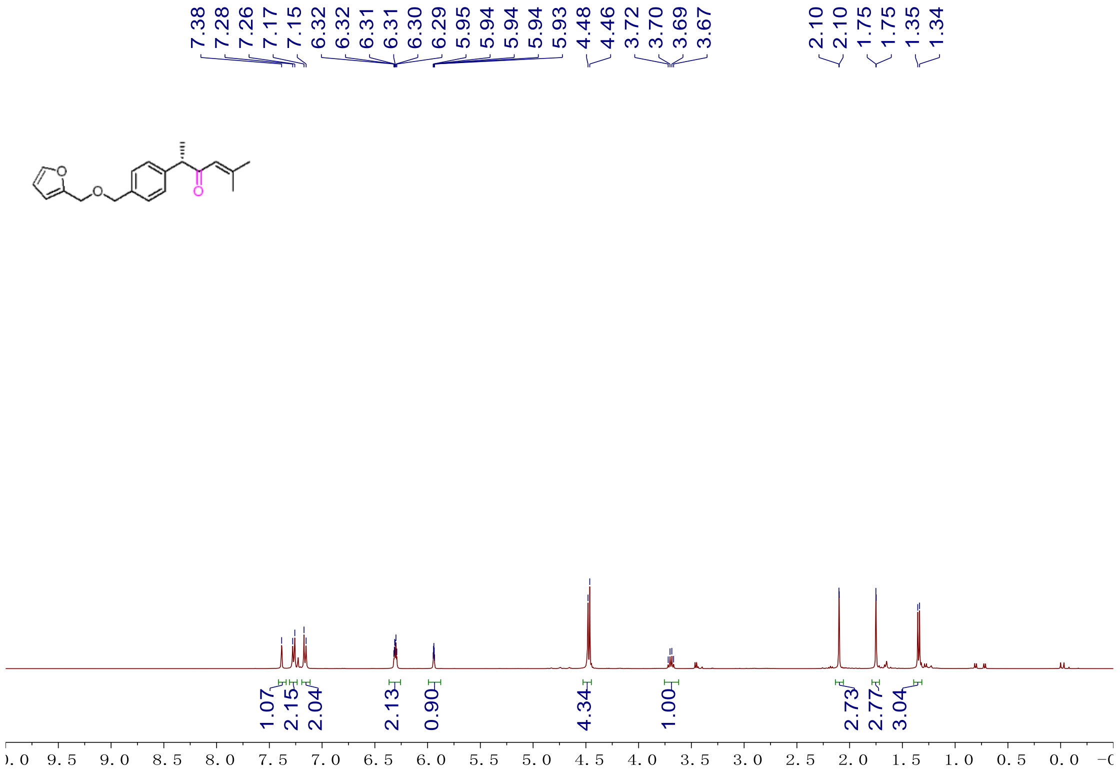

^{13}C NMR (100 MHz, $CDCl_3$) - (3v)

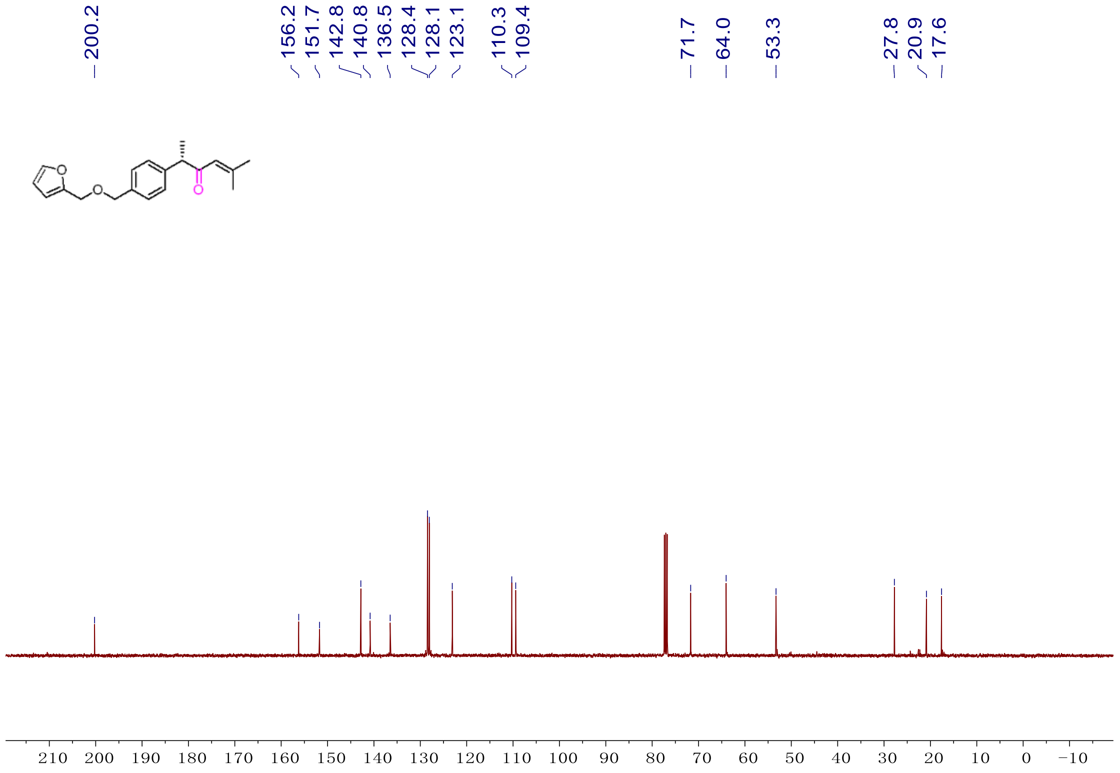

^{1}H NMR (400 MHz, $CDCl_3$) - (3w)

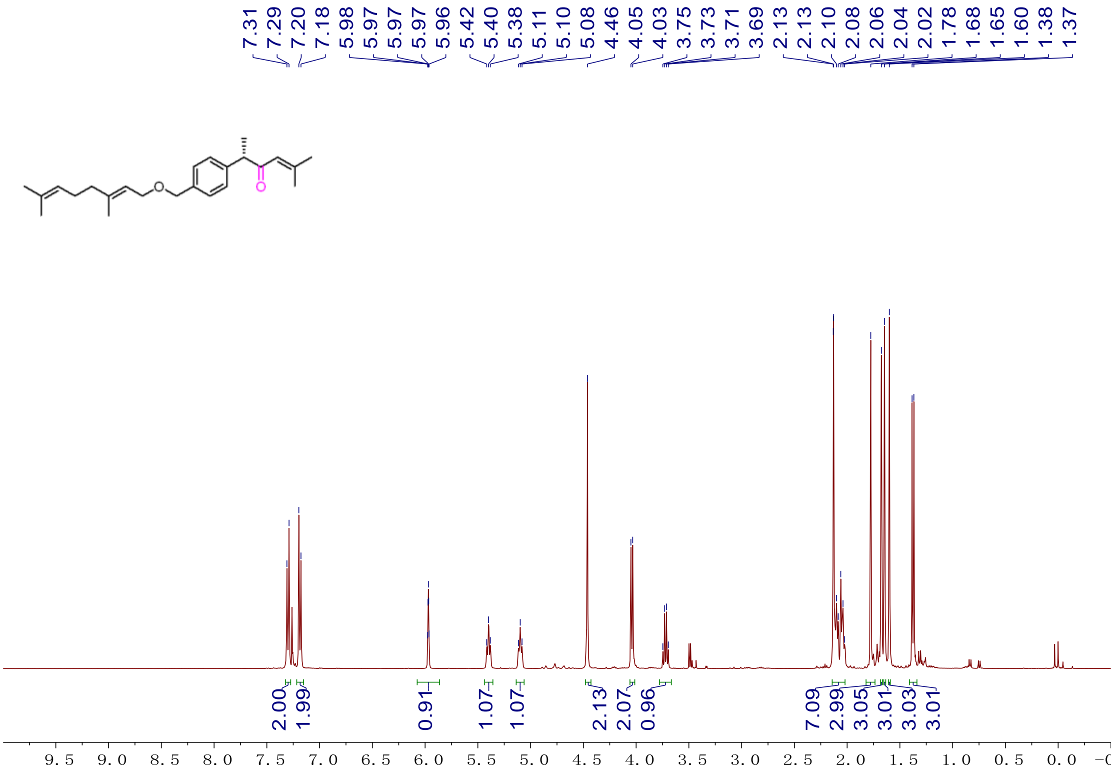

^{13}C NMR (100 MHz, $CDCl_3$) - (3w)

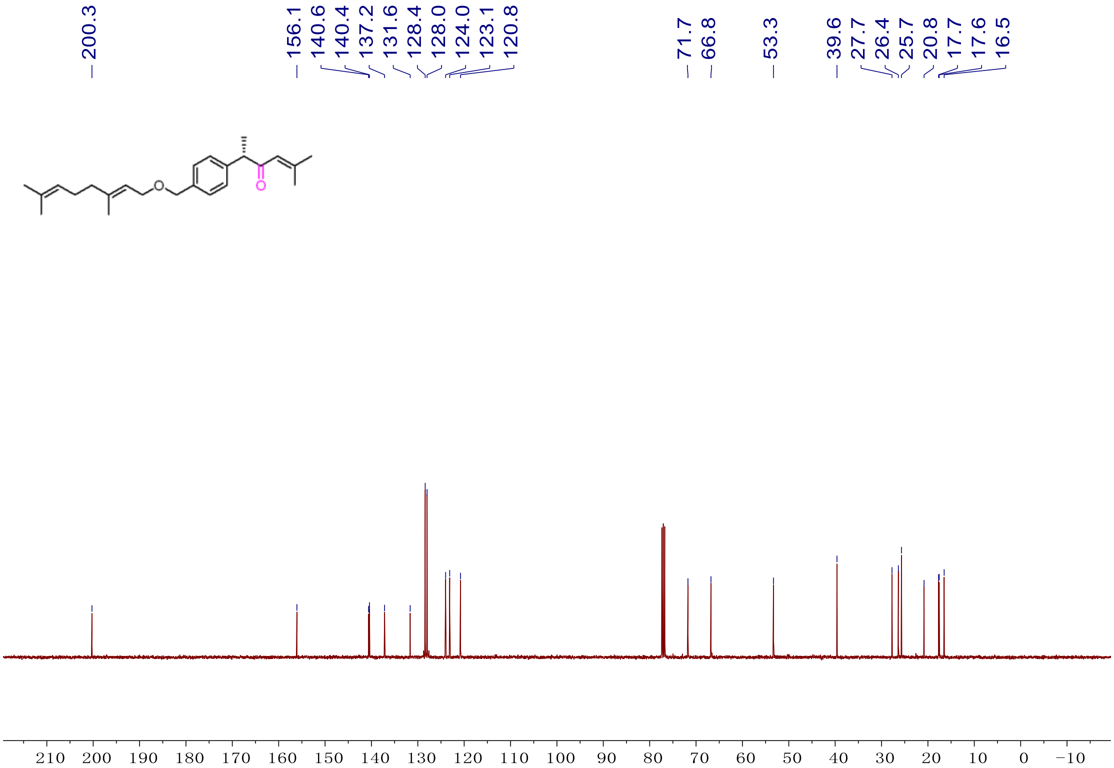

^{1}H NMR (400 MHz, $CDCl_3$) - (3x)

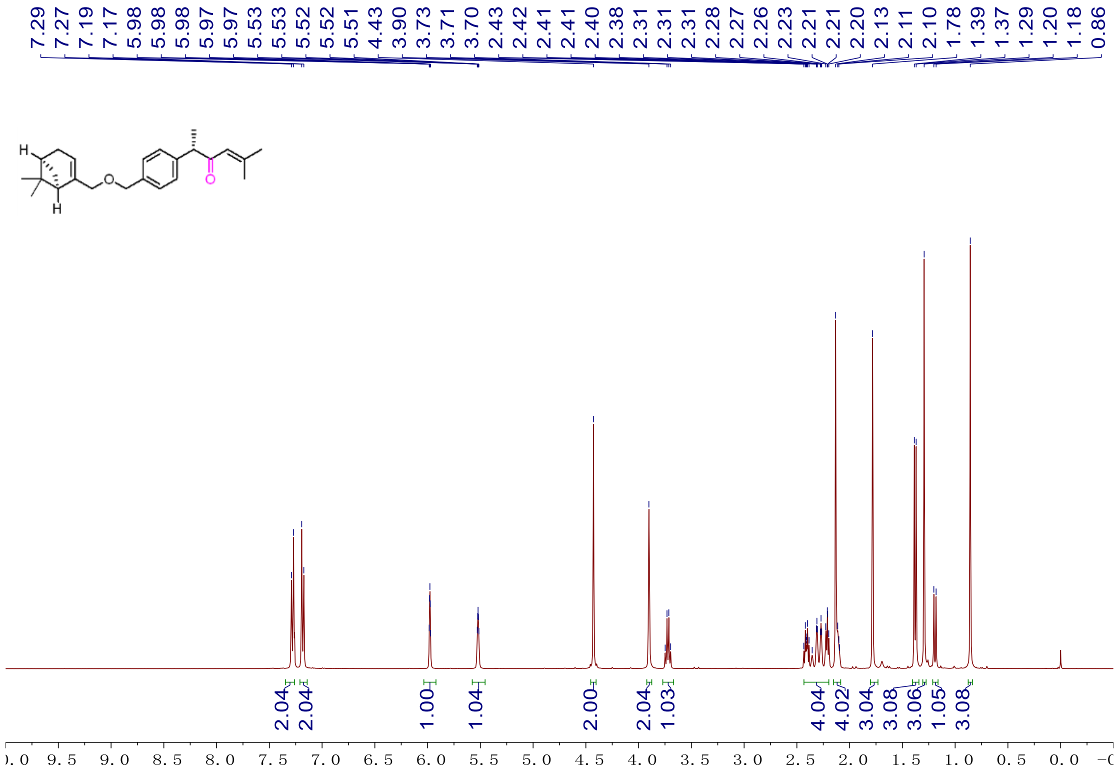

^{13}C NMR (100 MHz, $CDCl_3$) - (3x)

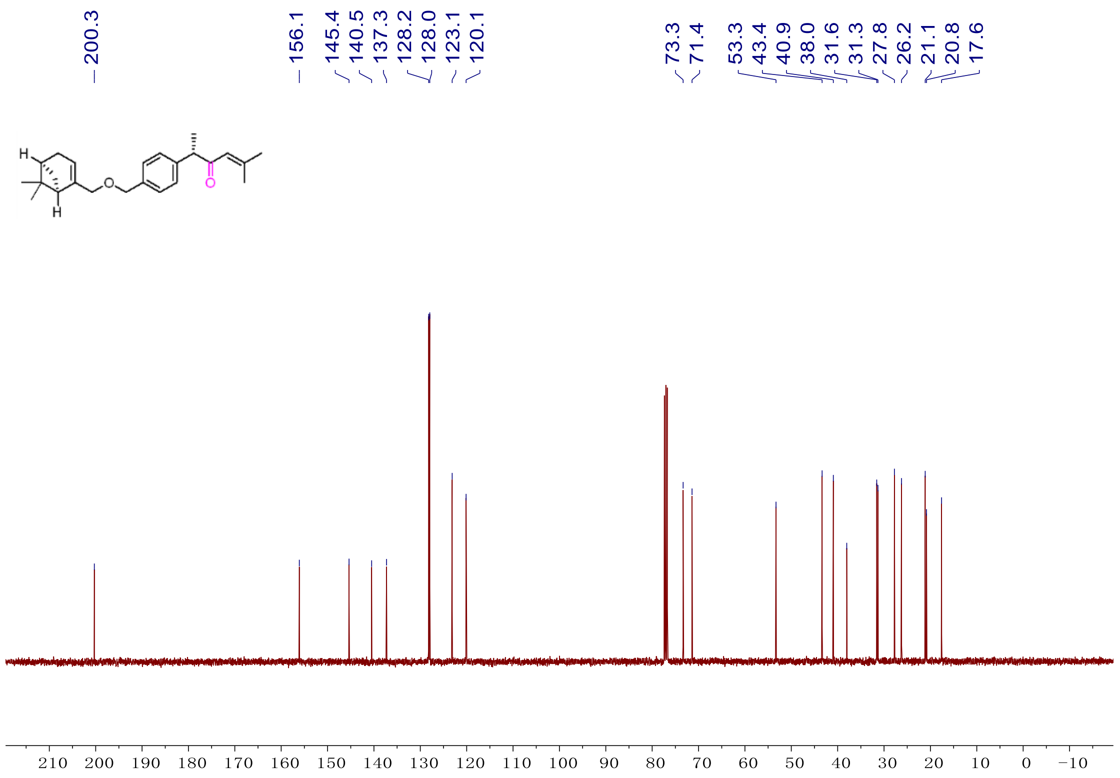

^{1}H NMR (400 MHz, $CDCl_3$) - (3y)

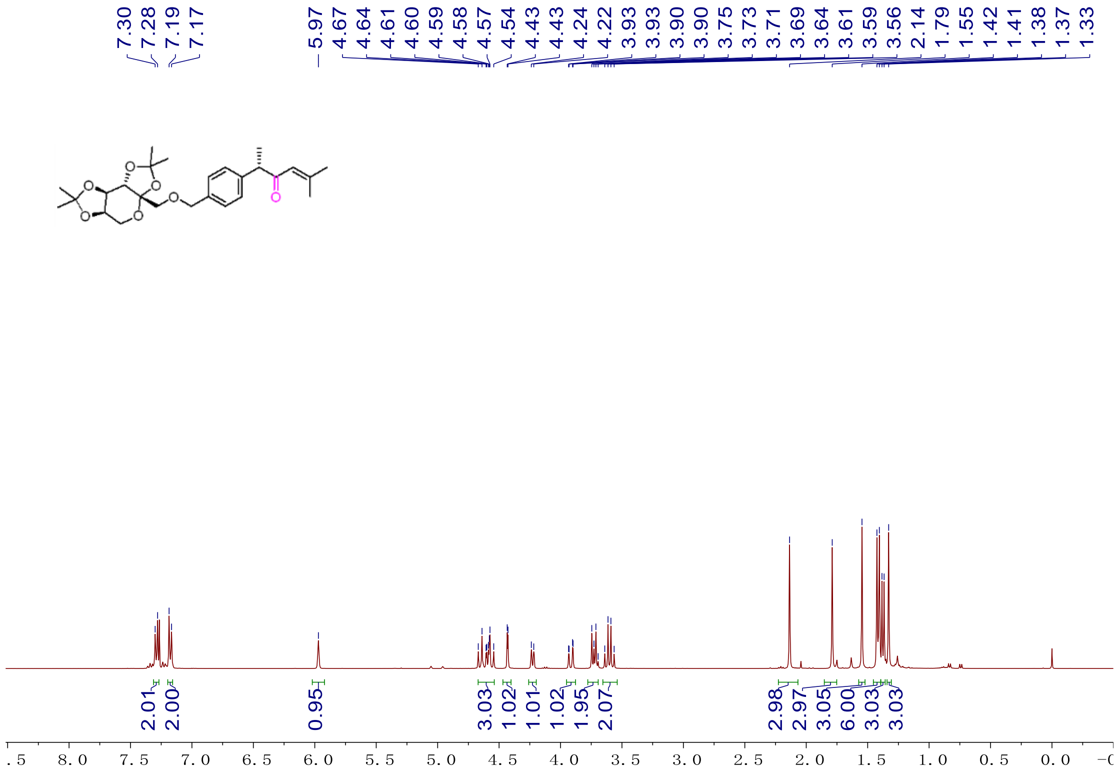

^{13}C NMR (100 MHz, $CDCl_3$) - (3y)

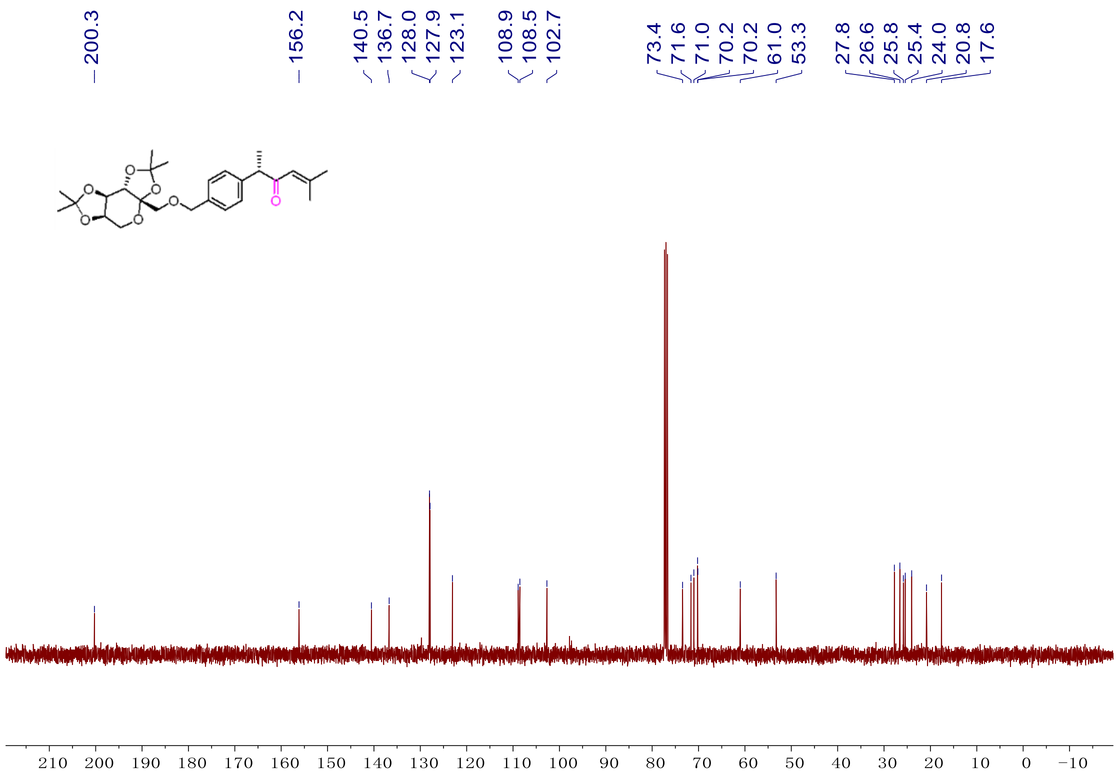

^{1}H NMR (400 MHz, $CDCl_3$) - (3z)

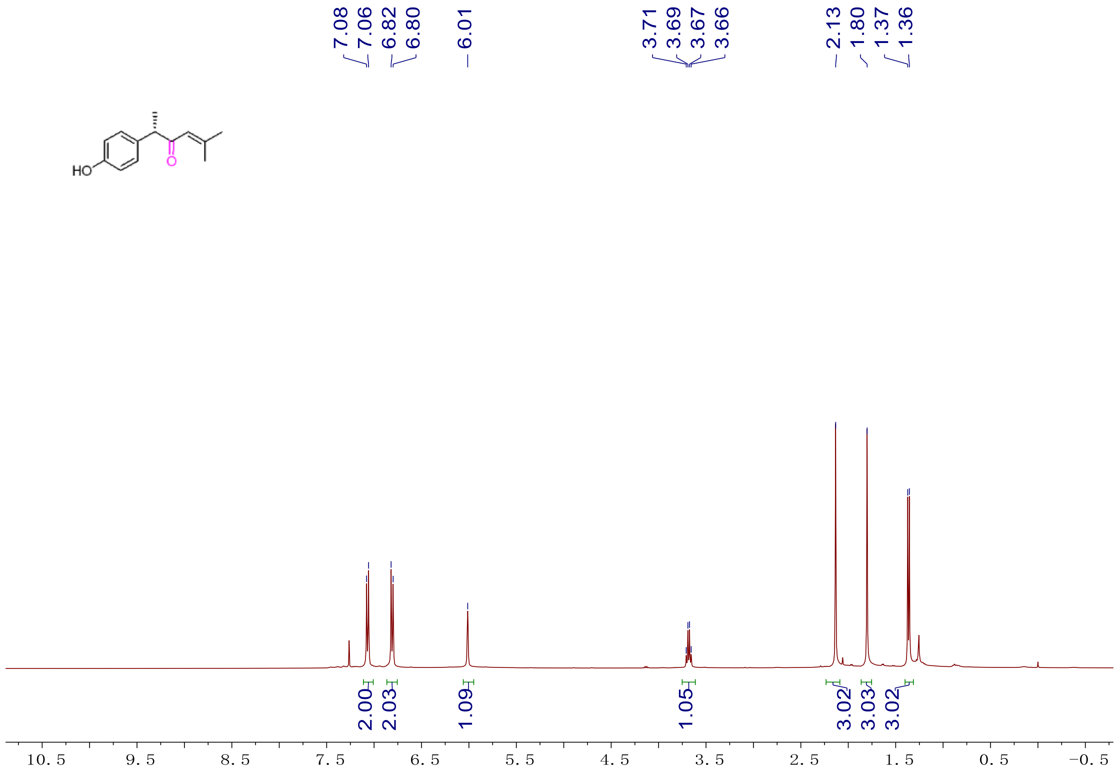

^{13}C NMR (100 MHz, $CDCl_3$) - (3z)

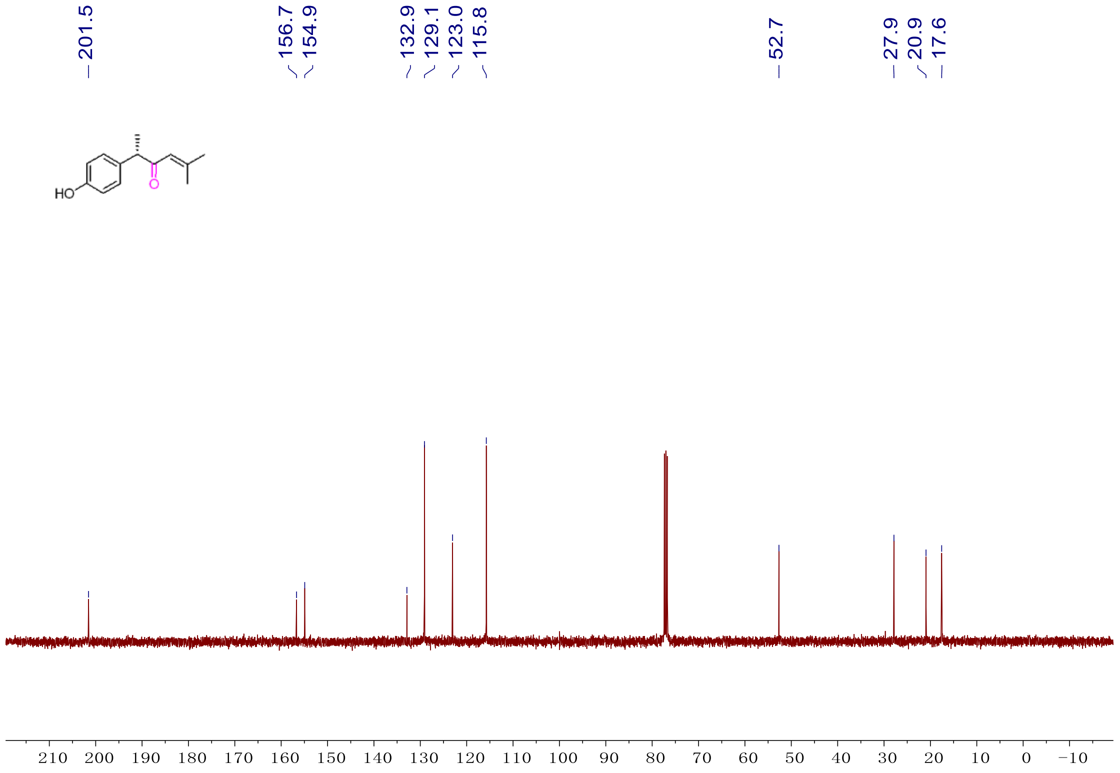

^{1}H NMR (400 MHz, $CDCl_3$) - (3aa)

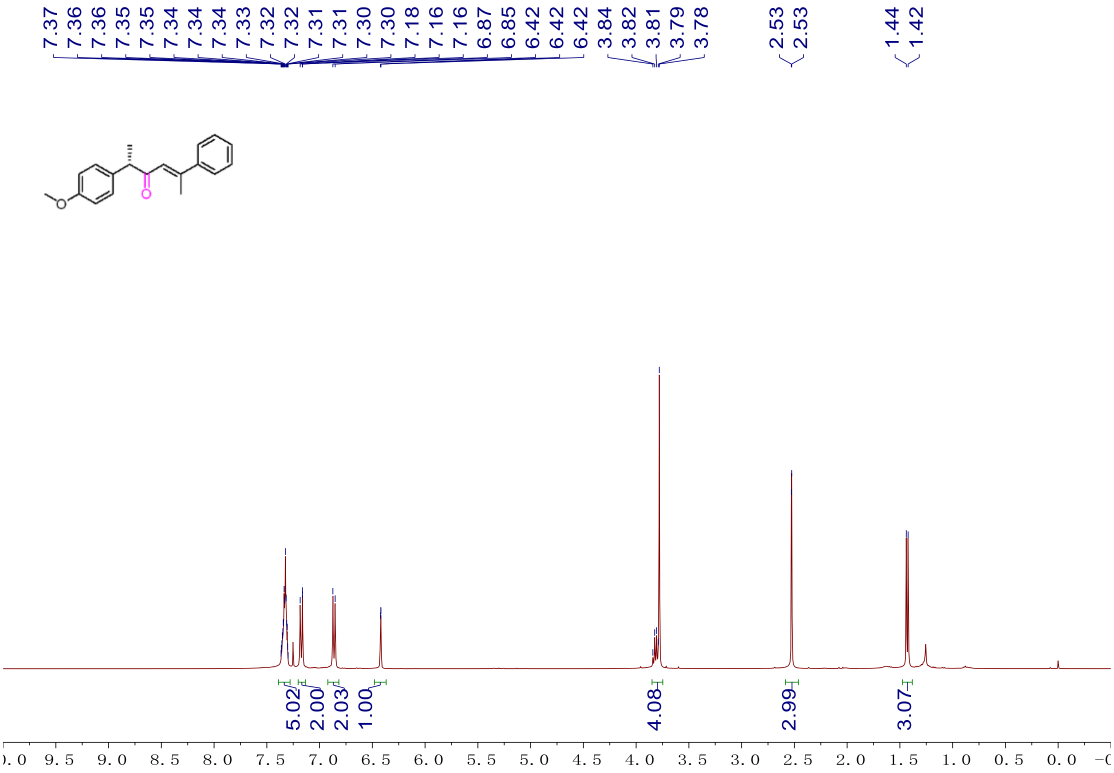

^{13}C NMR (100 MHz, $CDCl_3$) - (3aa)

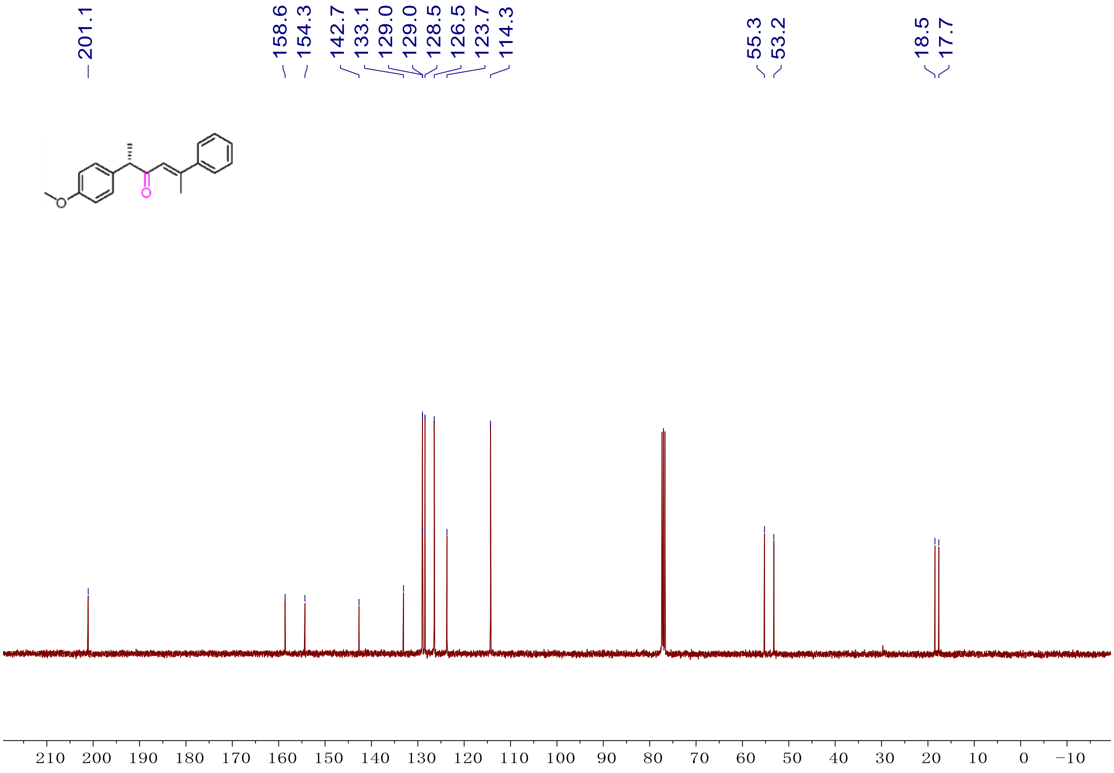

^{1}H NMR (400 MHz, $CDCl_3$) - (3ab)

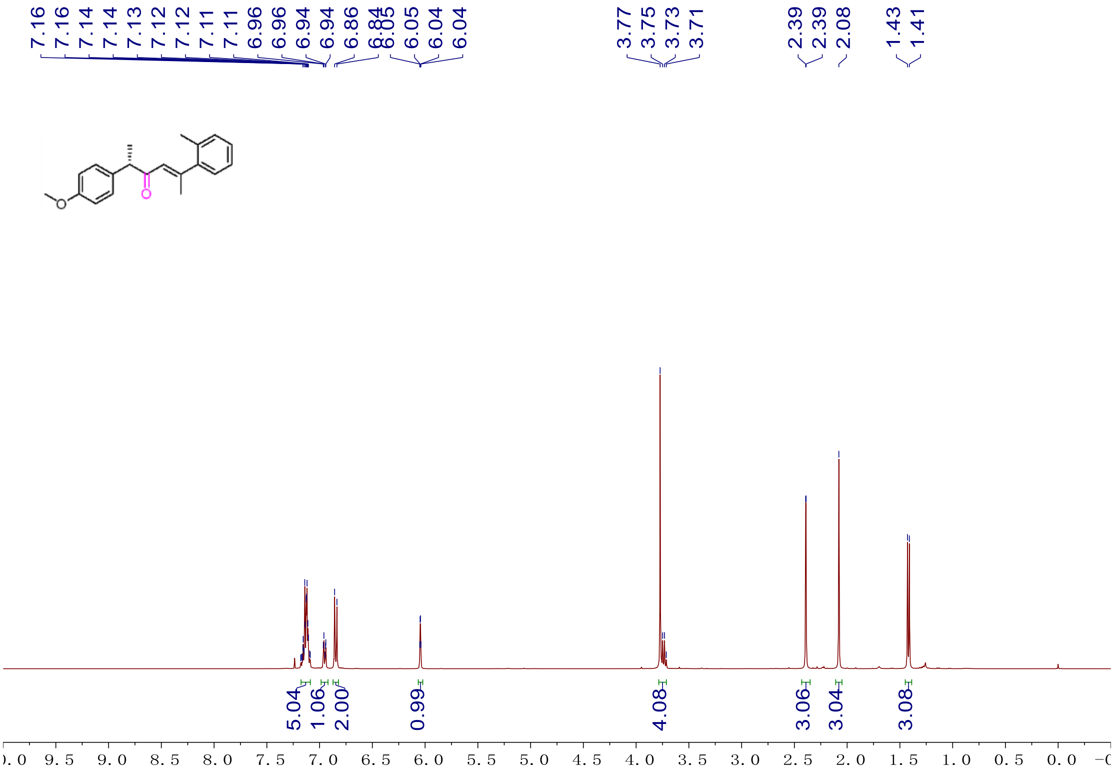

^{13}C NMR (100 MHz, $CDCl_3$) - (3ab)

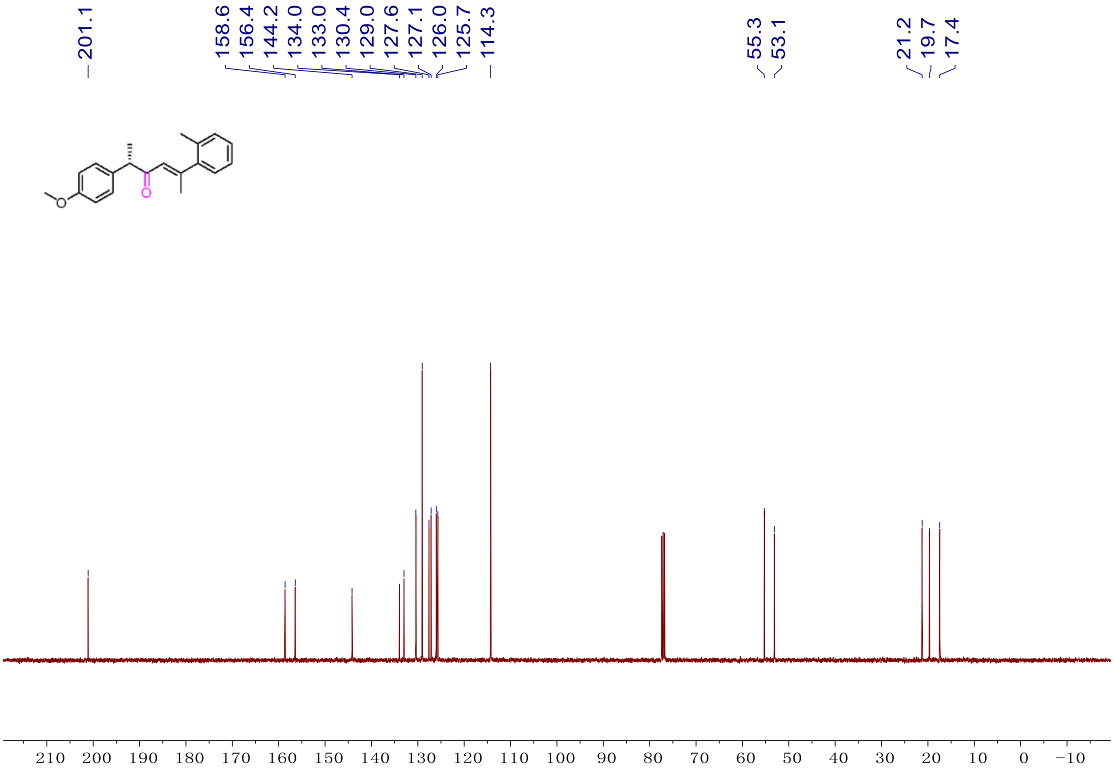

^{1}H NMR (400 MHz, $CDCl_3$) - (3ac)

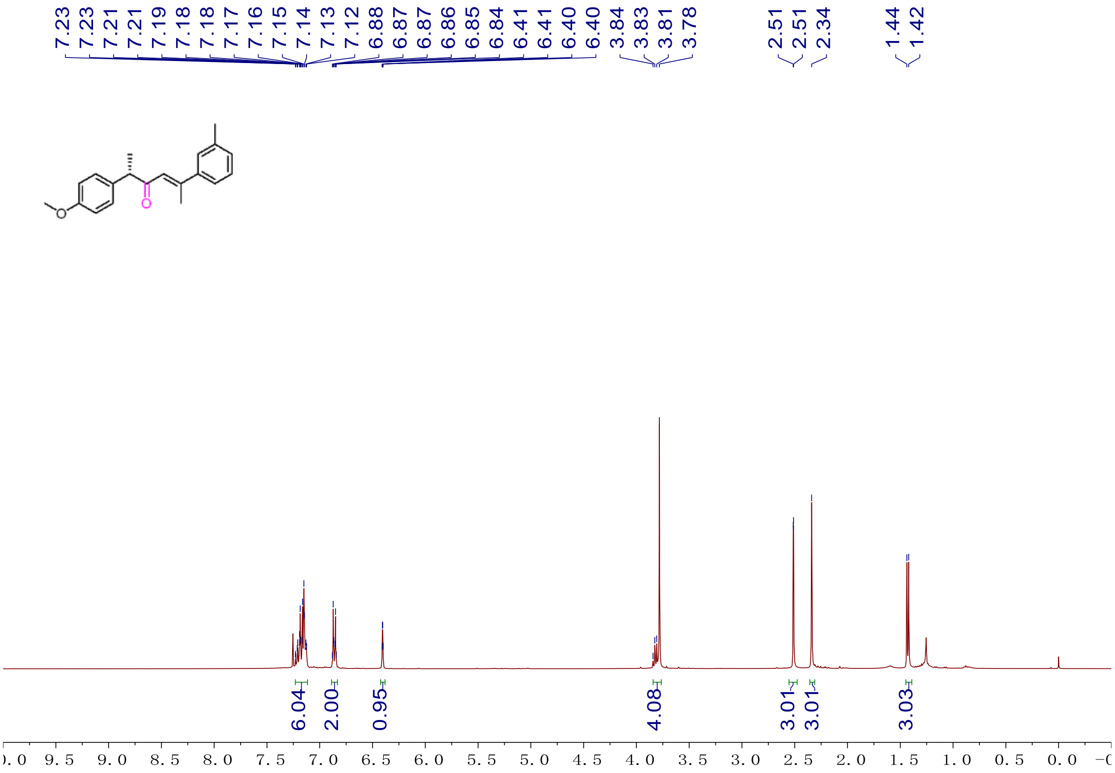

^{13}C NMR (100 MHz, $CDCl_3$) - (3ac)

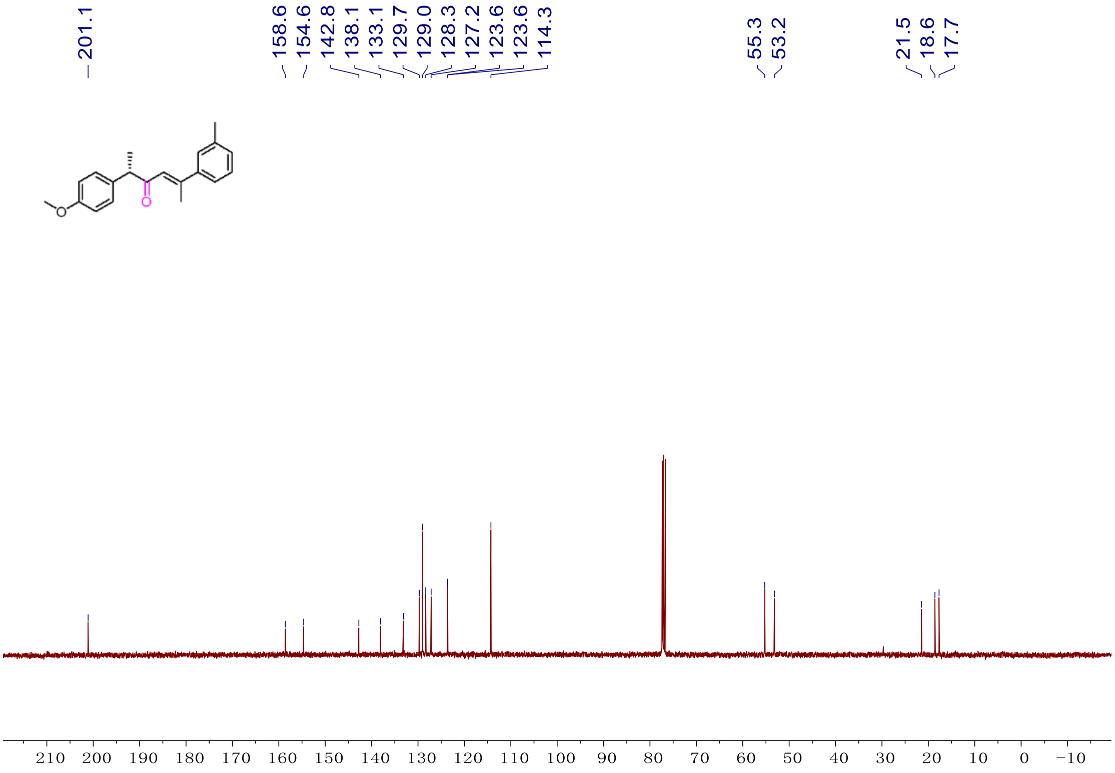

^{1}H NMR (400 MHz, $CDCl_3$) - (3ad)

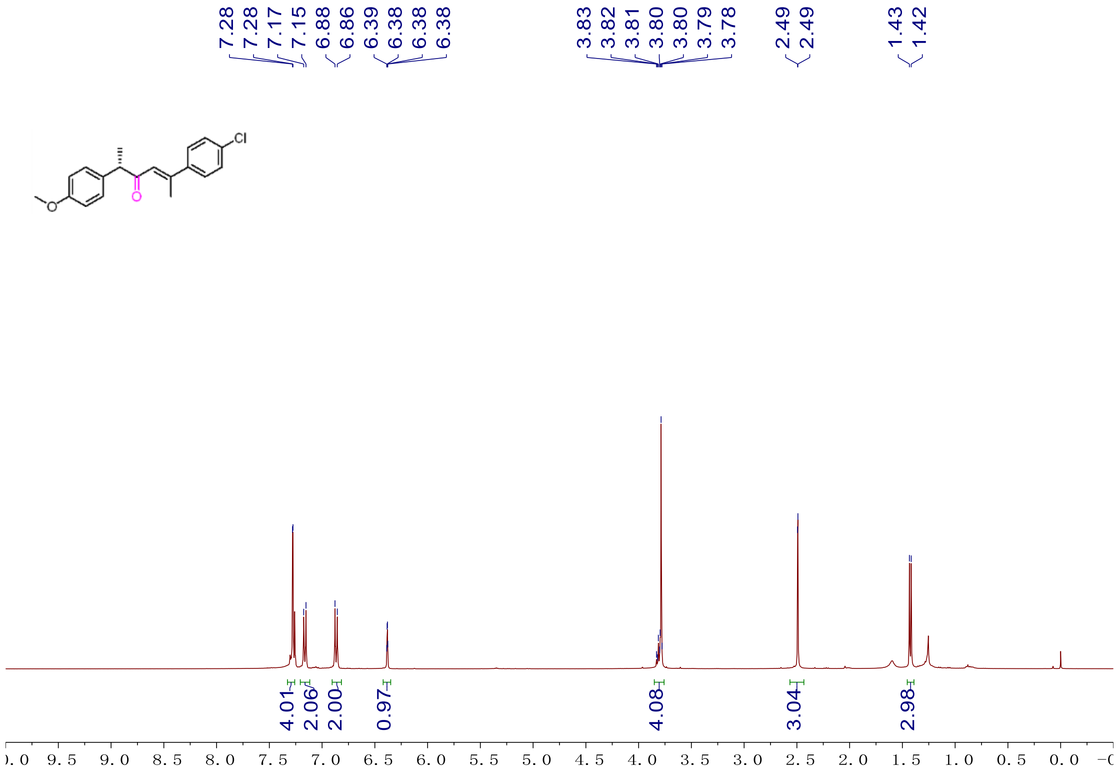

^{13}C NMR (100 MHz, $CDCl_3$) - (3ad)

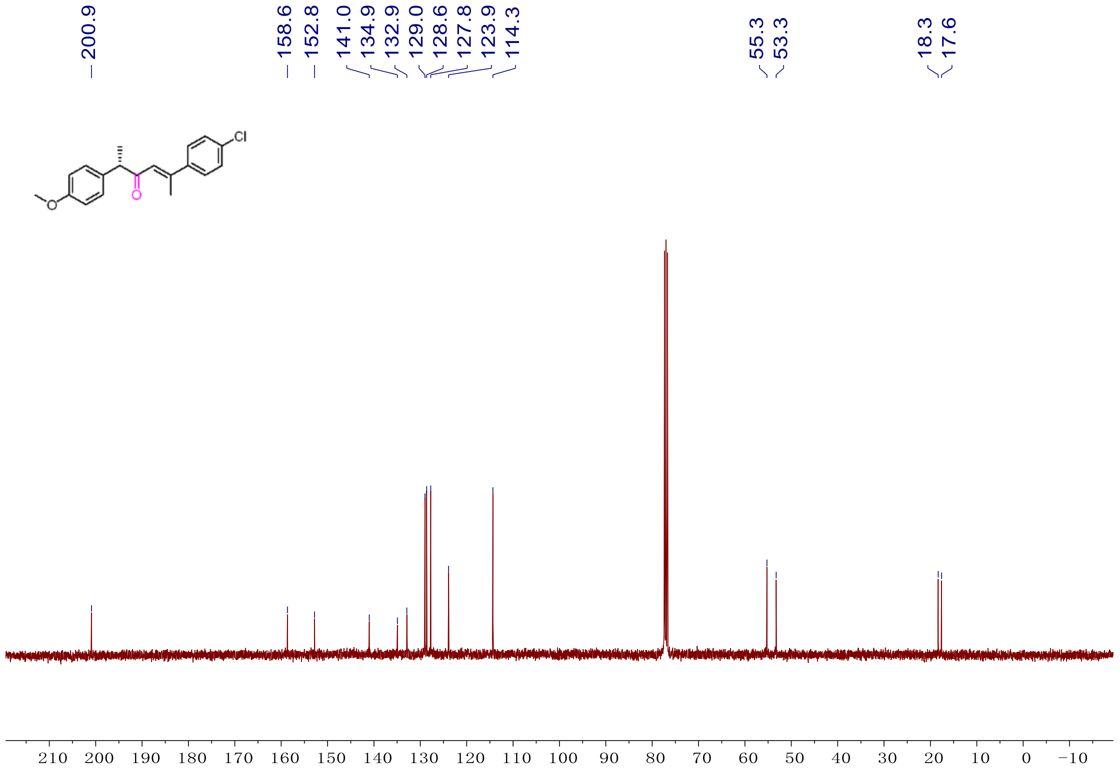

^{1}H NMR (400 MHz, $CDCl_3$) - (3ae)

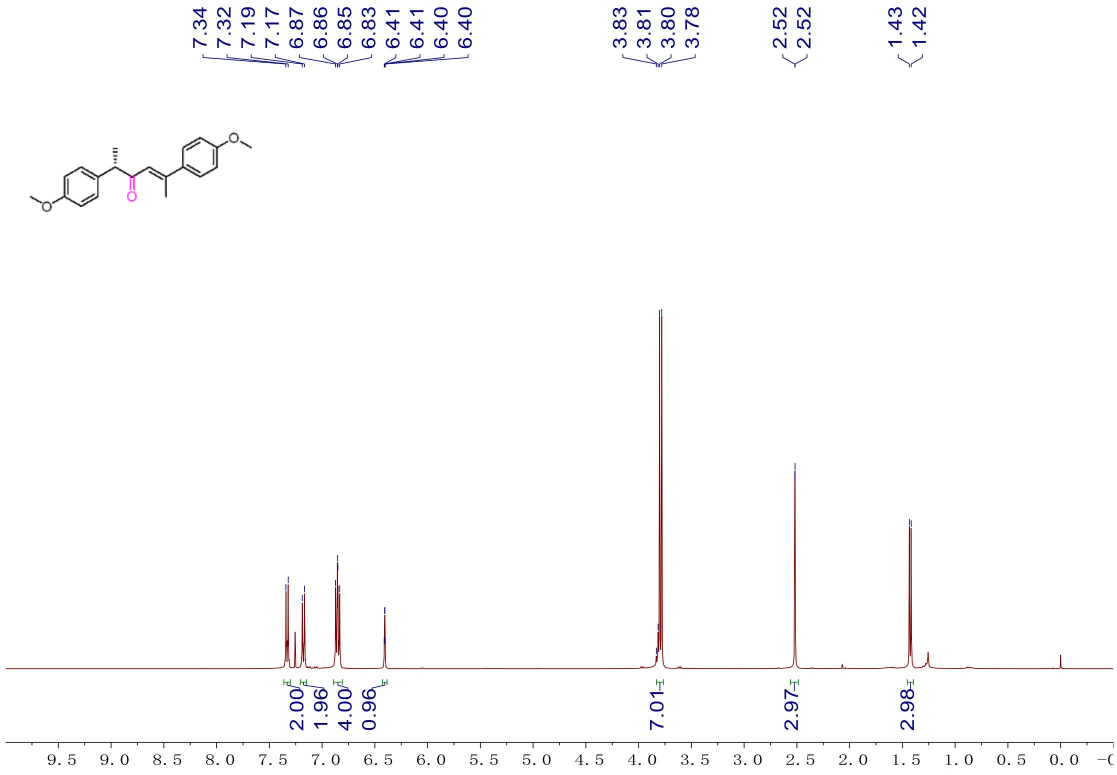

^{13}C NMR (100 MHz, $CDCl_3$) - (3ae)

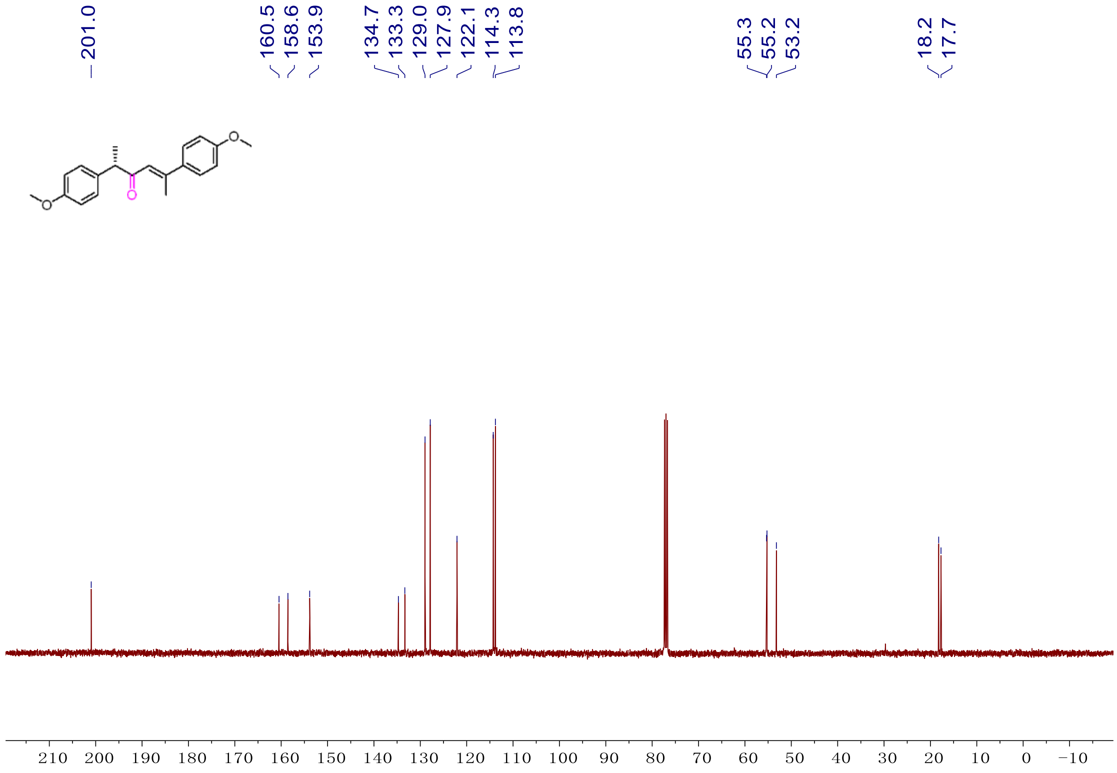

^{1}H NMR (400 MHz, $CDCl_3$) - (3af)

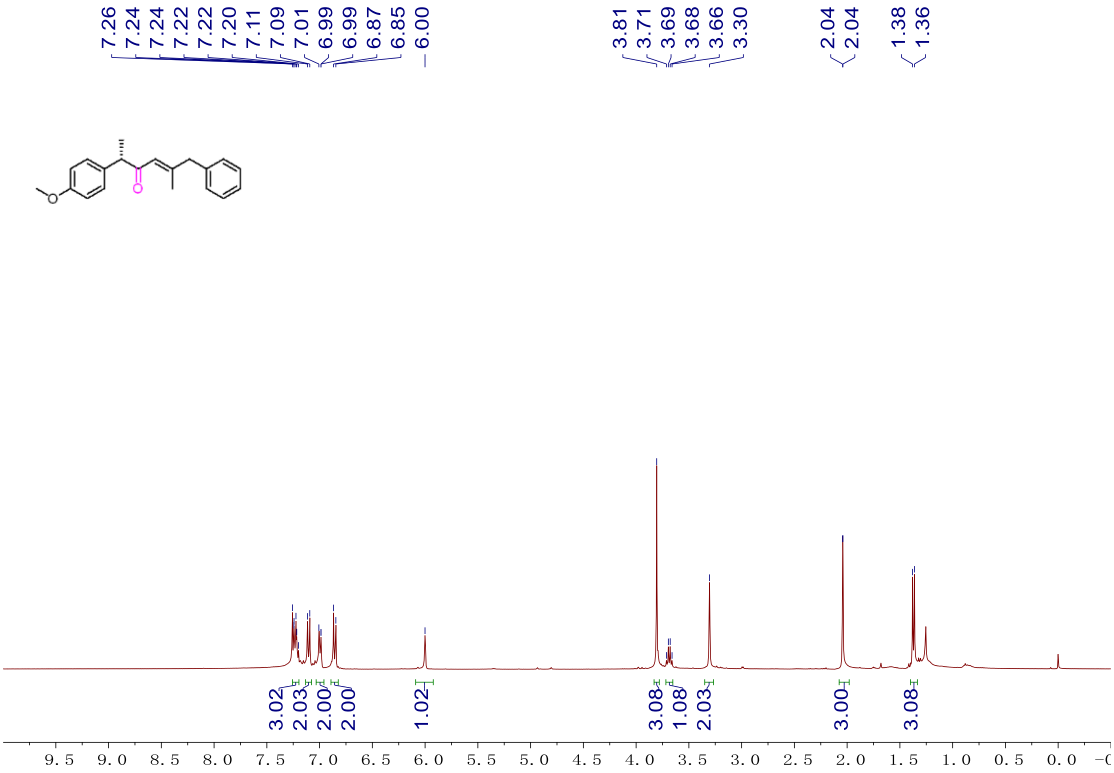

^{13}C NMR (100 MHz, $CDCl_3$) - (3af)

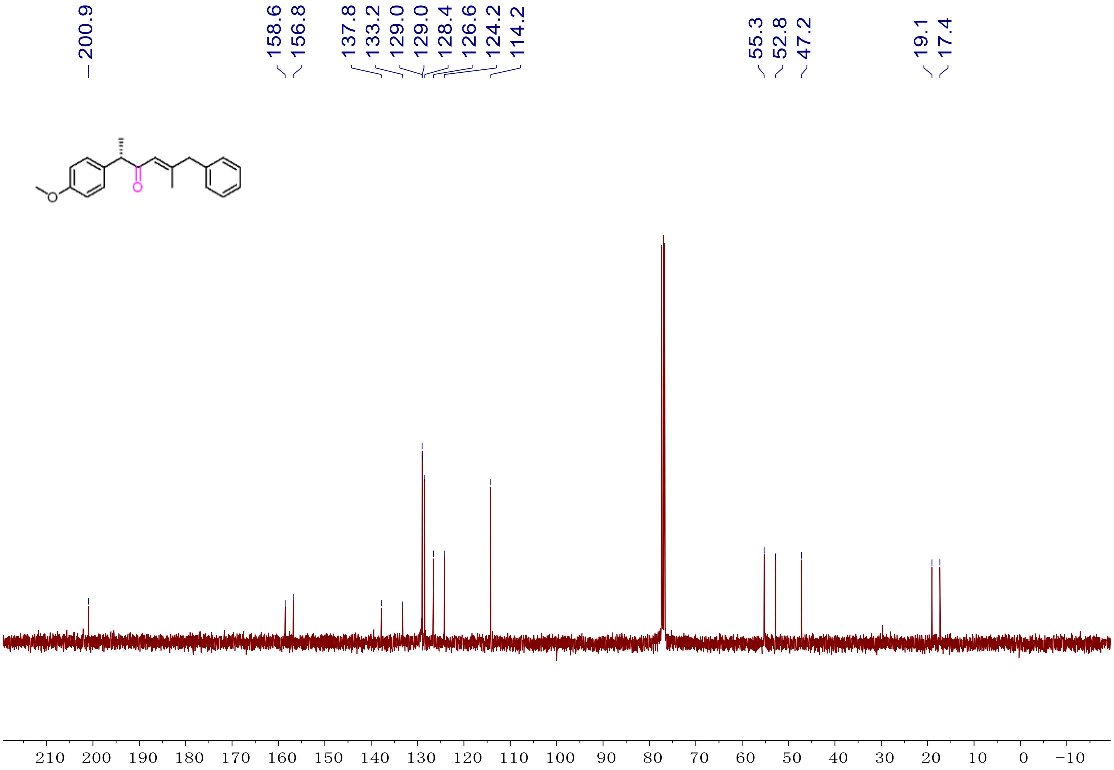

^{1}H NMR (400 MHz, $CDCl_3$) - (5)

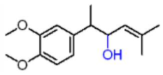

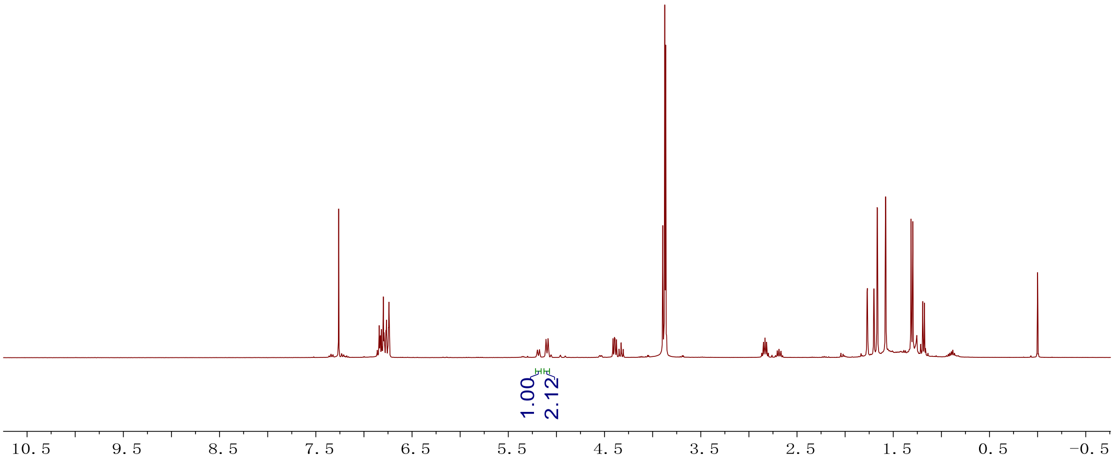

^{1}H NMR (400 MHz, CDCl$_3$) - (5a)

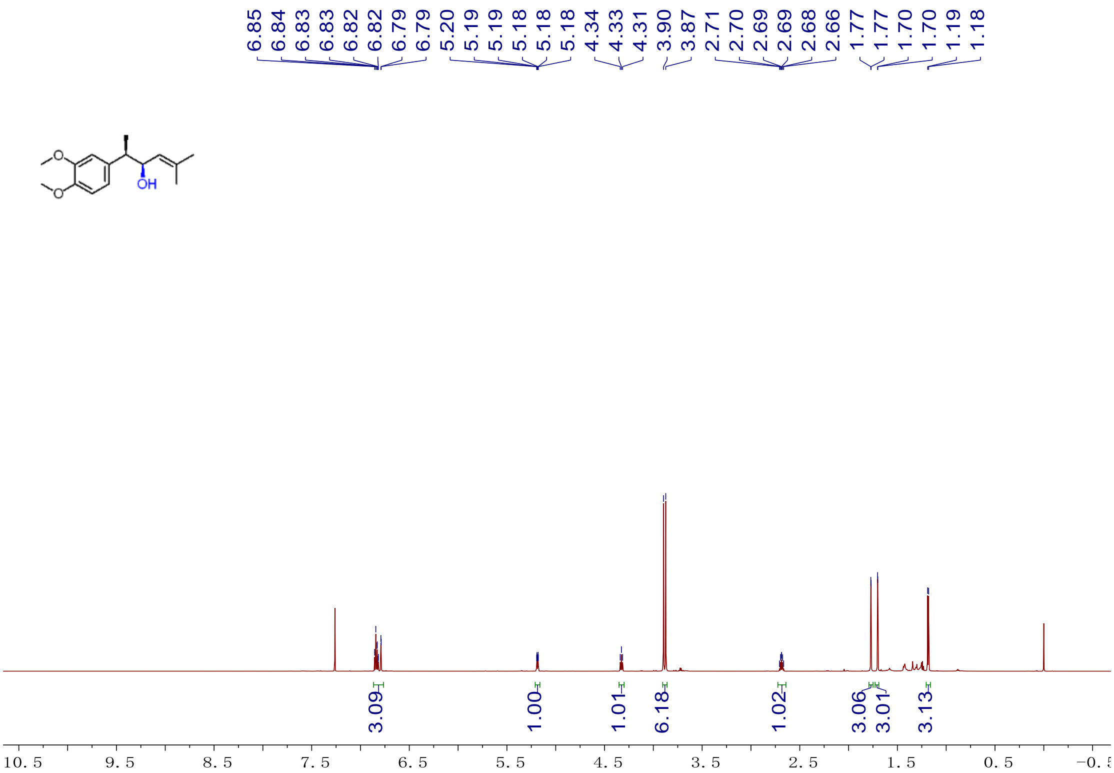

^{13}C NMR (100 MHz, CDCl$_3$) - (5a)

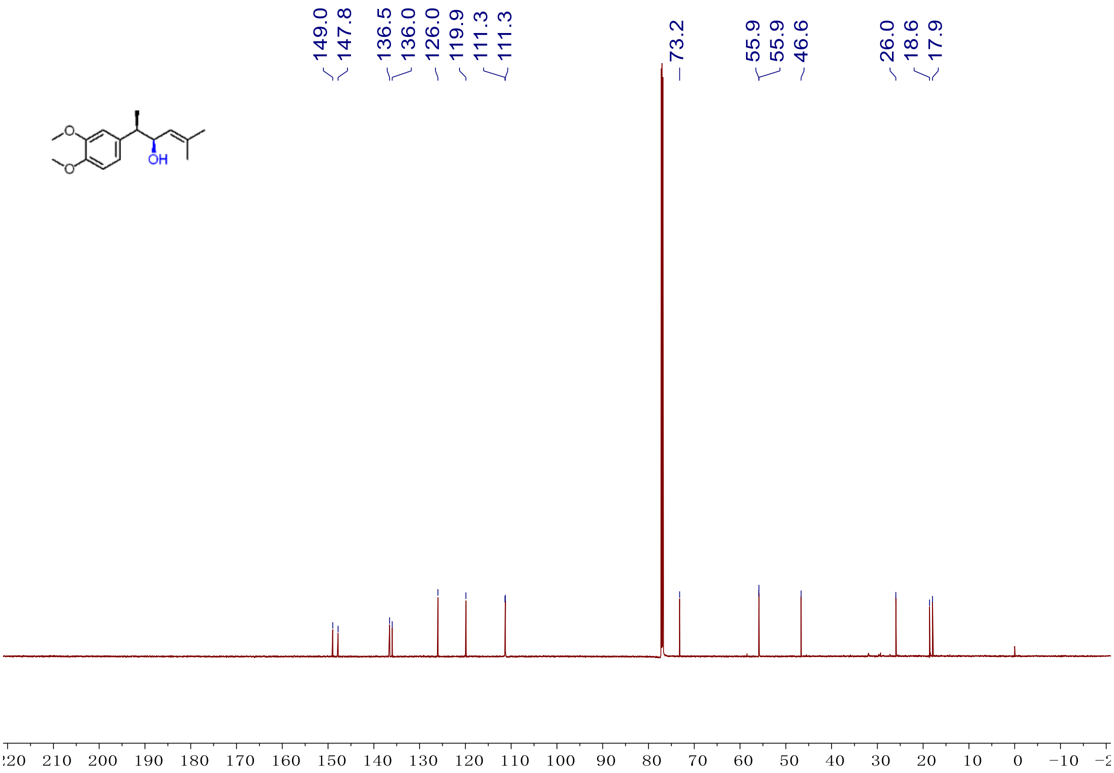

^{1}H NMR (400 MHz, CDCl3) - (5b)

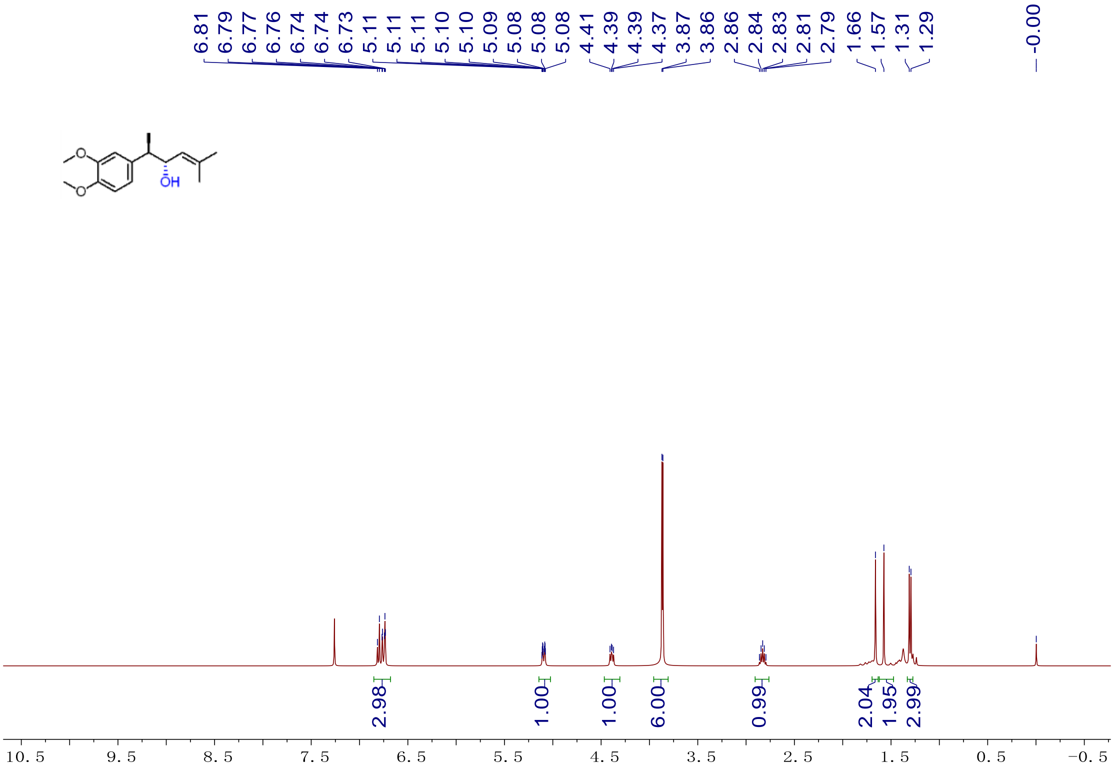

^{13}C NMR (100 MHz, CDCl3) - (5b)

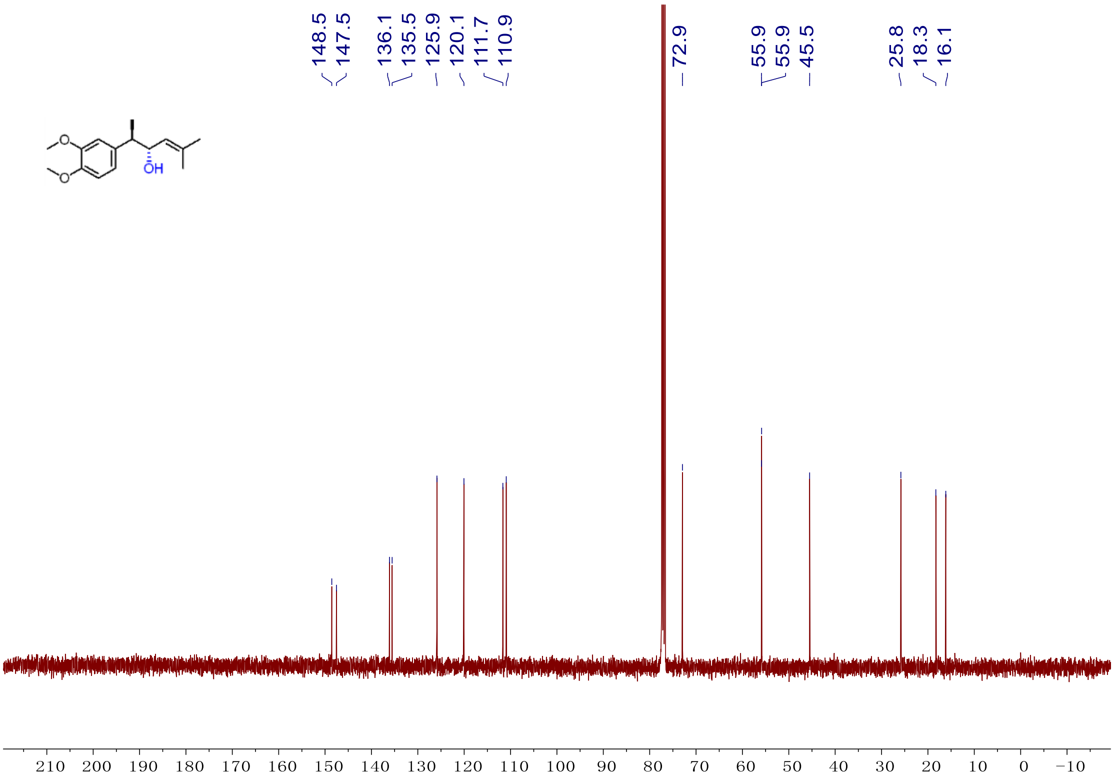

^{1}H NMR (400 MHz, $CDCl_3$) - (5c)

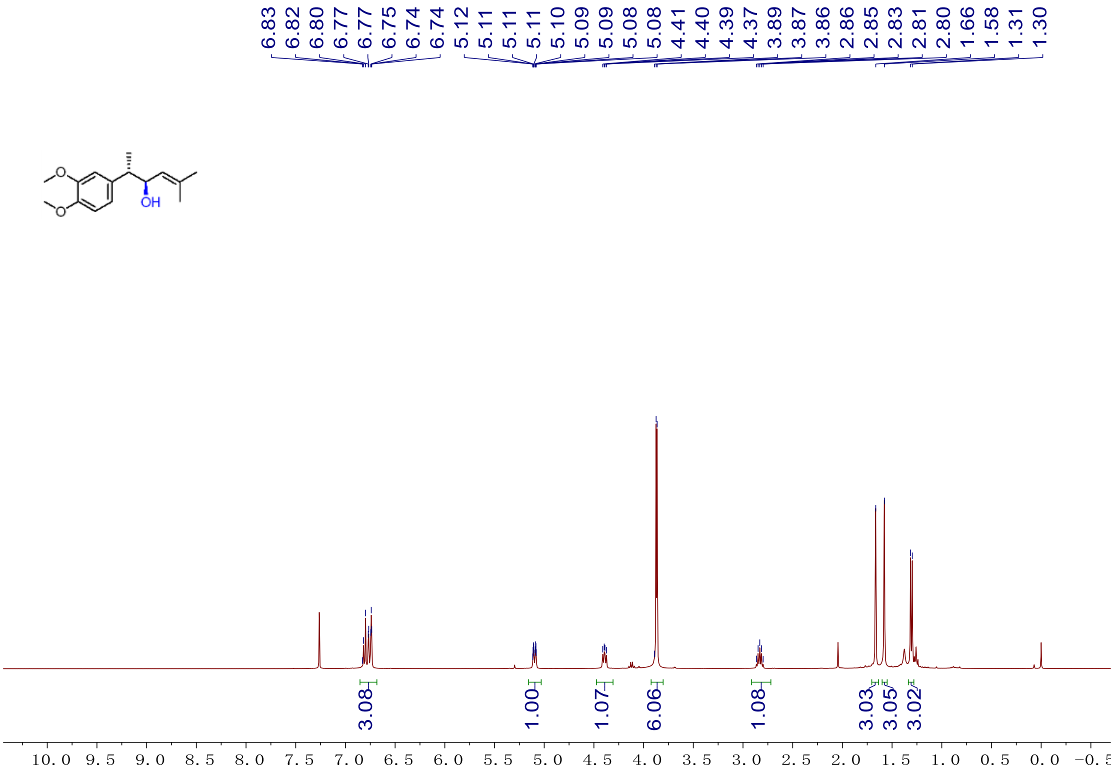

^{13}C NMR (100 MHz, $CDCl_3$) - (5c)

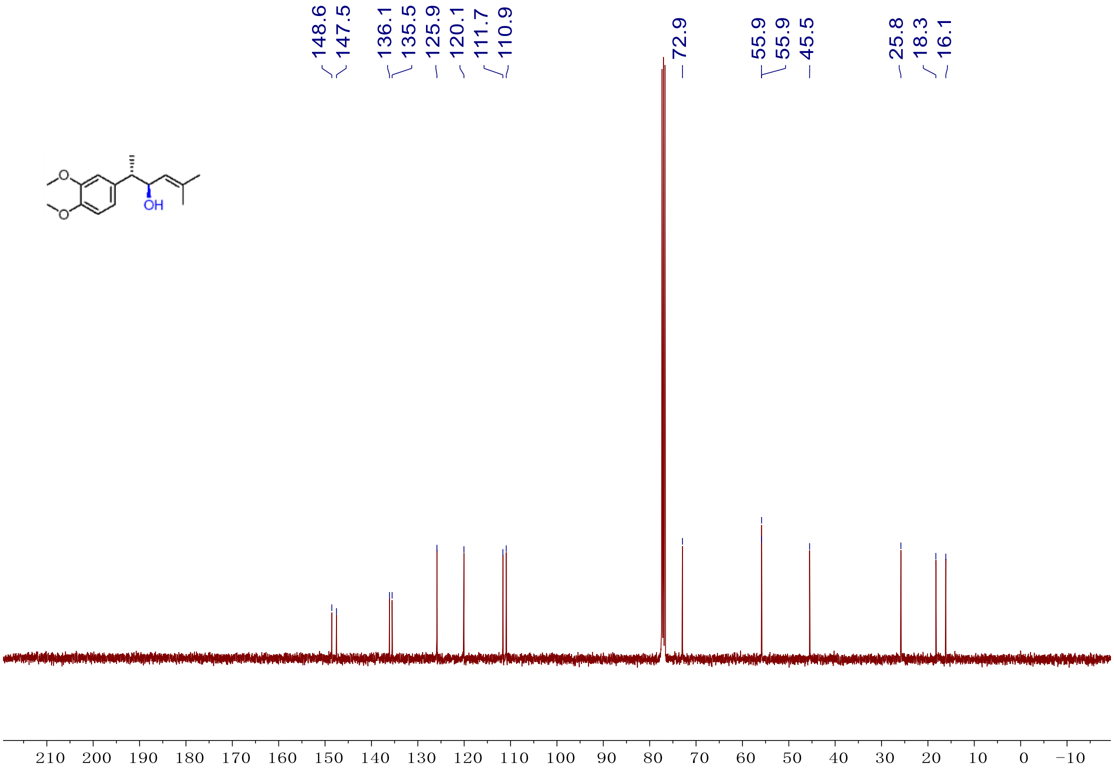

^{1}H NMR (400 MHz, $CDCl_3$) - (5d)

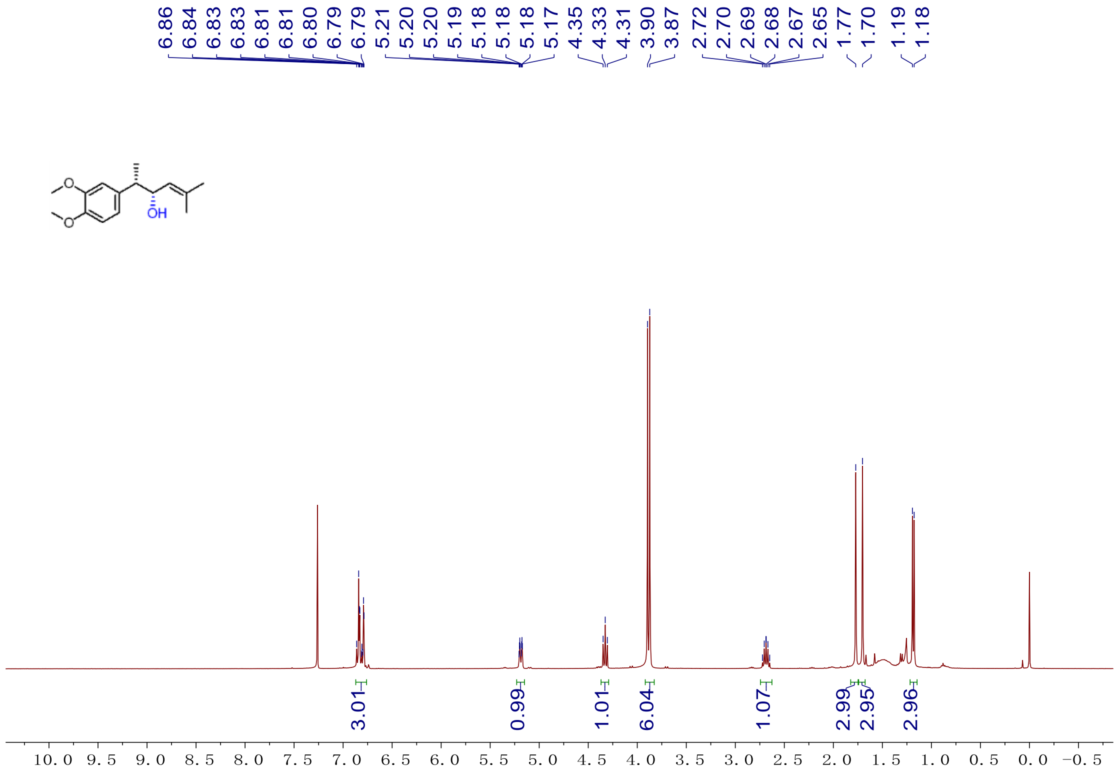

^{13}C NMR (100 MHz, $CDCl_3$) - (5d)

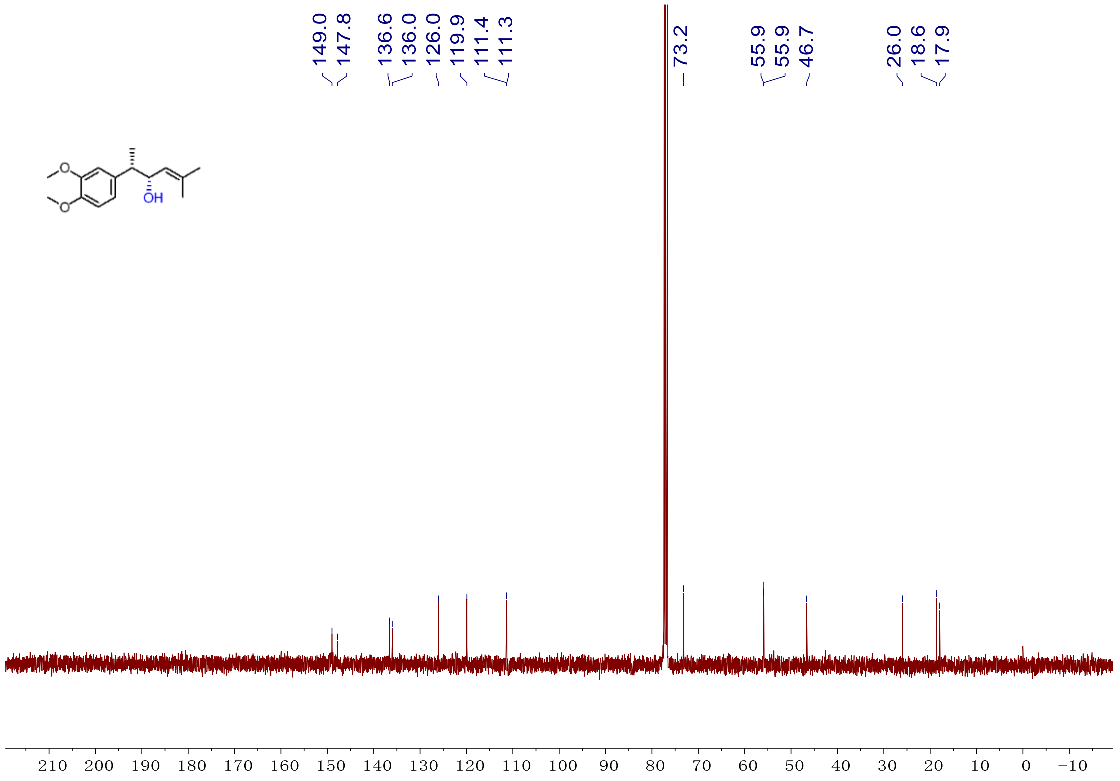

9. Copies of HPLC chromatogram

HPLC chromatogram (3a)

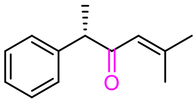

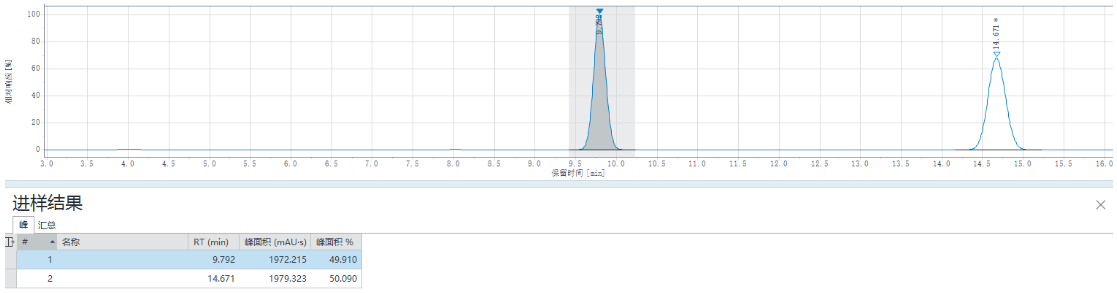

Peak (#)	Ret Time (min)	Area (%)
1	9.792	49.910
2	14.671	50.090

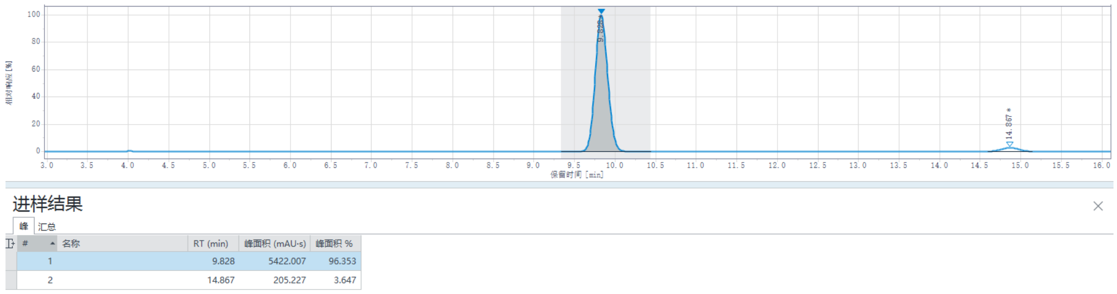

Peak (#)	Ret Time (min)	Area (%)
1	9.828	96.353
2	14.867	3.647

HPLC chromatogram (3b)

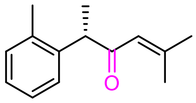

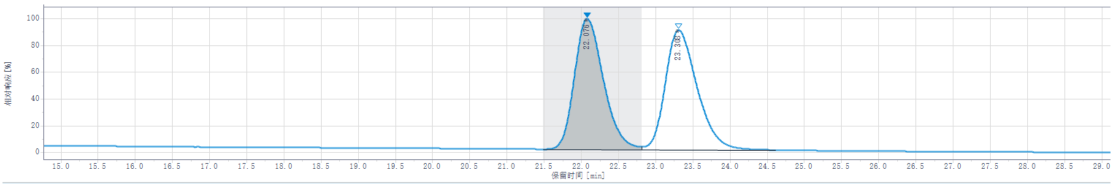

进样结果

峰 汇总

#	名称	RT (min)	峰面积 (mAU·s)	峰面积 %
1		22.076	407.836	49.913
2		23.308	409.261	50.087

Peak (#)	Ret Time (min)	Area (%)
1	22.076	49.913
2	23.308	50.087

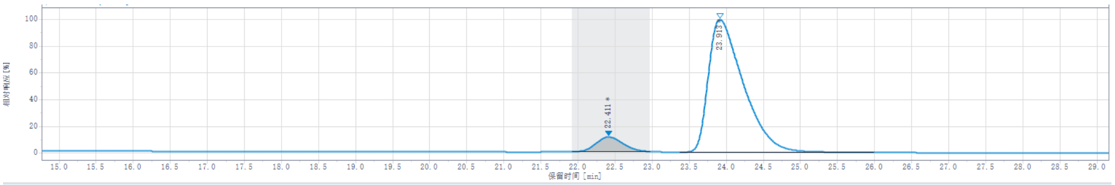

进样结果

峰 汇总

#	名称	RT (min)	峰面积 (mAU·s)	峰面积 %
1		22.411	102.443	7.715
2		23.913	1225.326	92.285

Peak (#)	Ret Time (min)	Area (%)
1	22.411	7.715
2	23.913	92.285

HPLC chromatogram (3c)

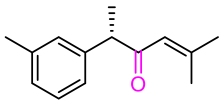

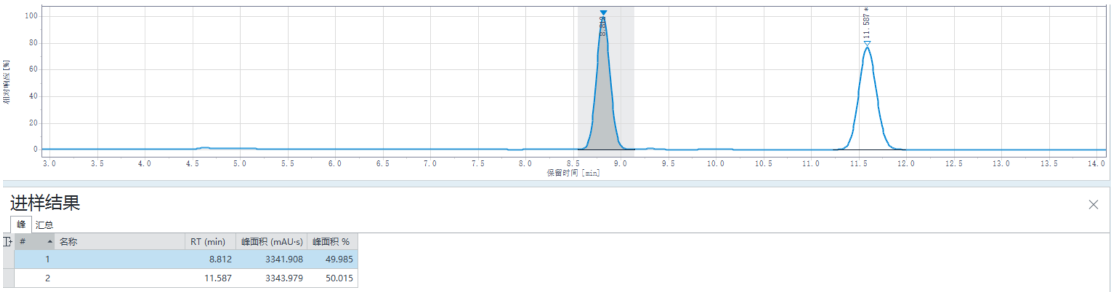

Peak (#)	Ret Time (min)	Area (%)
1	8.812	49.985
2	11.587	50.015

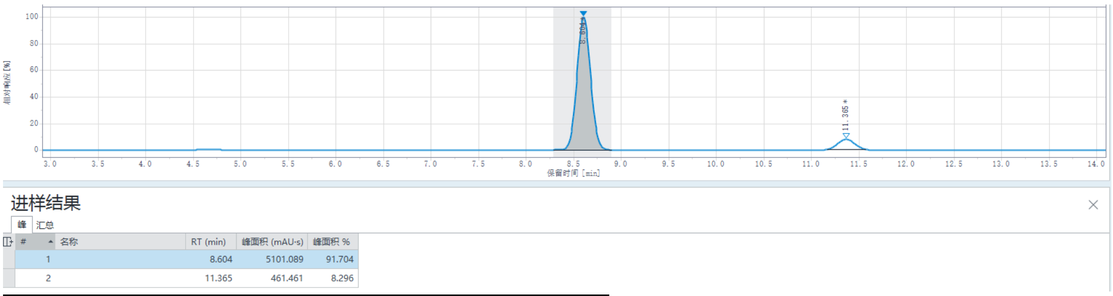

Peak (#)	Ret Time (min)	Area (%)
1	8.604	91.704
2	11.365	8.296

HPLC chromatogram (3d)

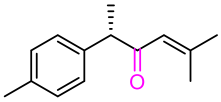

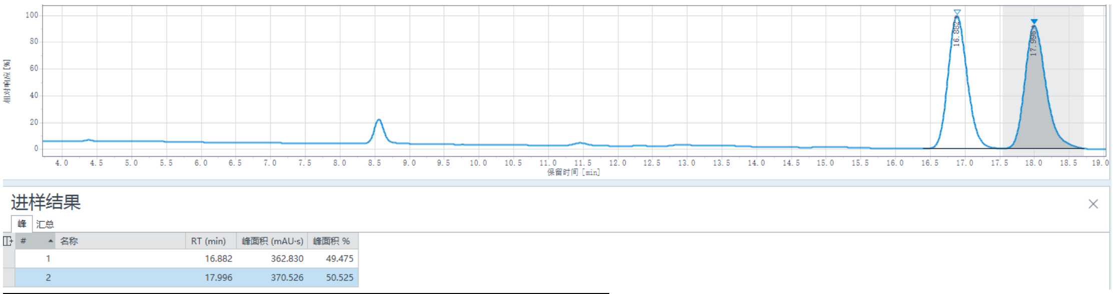

Peak (#)	Ret Time (min)	Area (%)
1	16.882	49.475
2	17.996	50.525

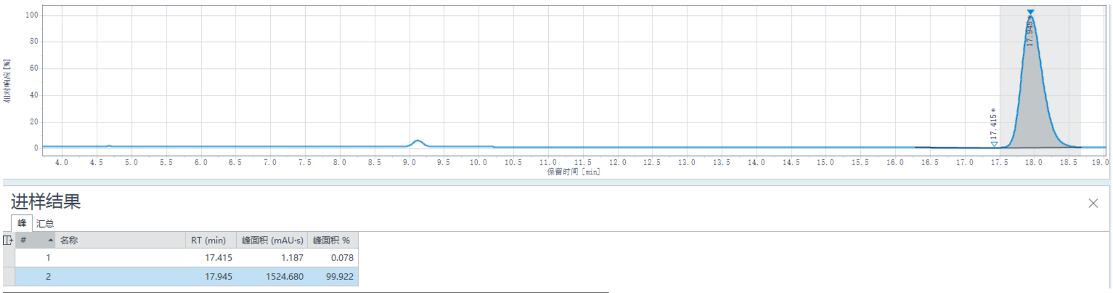

Peak (#)	Ret Time (min)	Area (%)
1	17.415	0.078
2	17.945	99.922

HPLC chromatogram (3e)

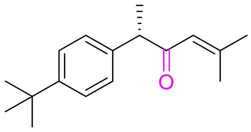

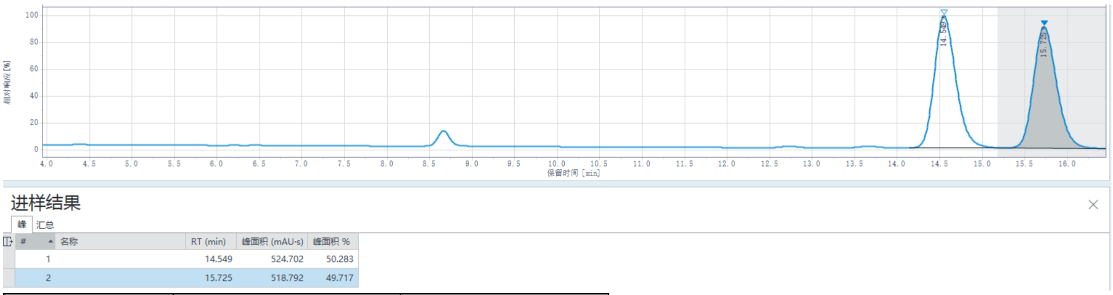

Peak (#)	Ret Time (min)	Area (%)
1	14.549	50.283
2	15.725	49.717

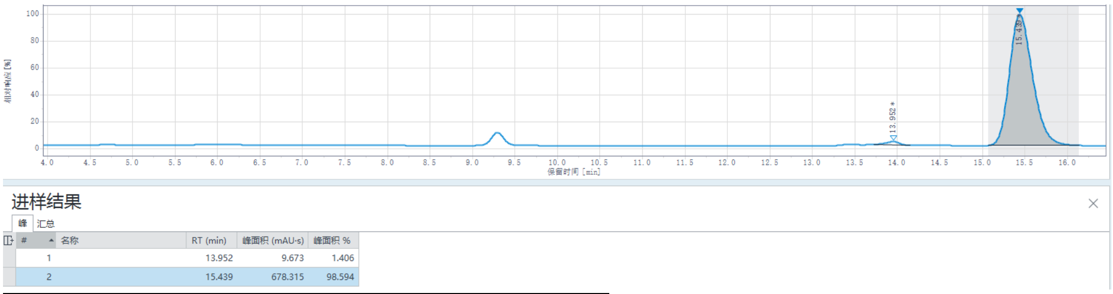

Peak (#)	Ret Time (min)	Area (%)
1	13.952	1.406
2	15.439	98.594

HPLC chromatogram (3f)

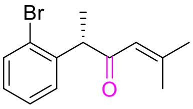

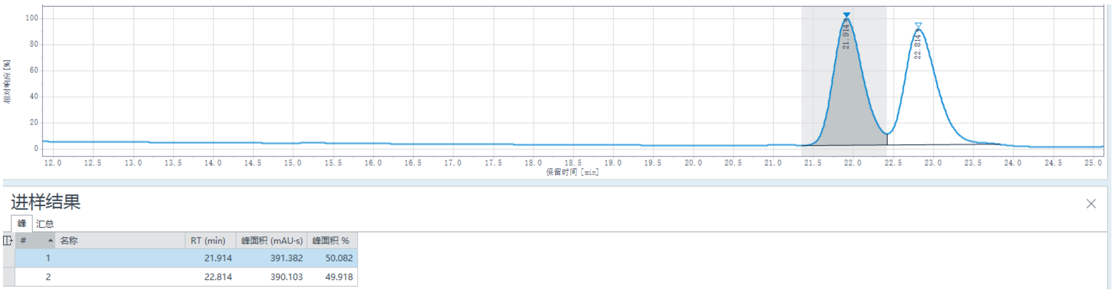

Peak (#)	Ret Time (min)	Area (%)
1	21.914	50.082
2	22.814	49.918

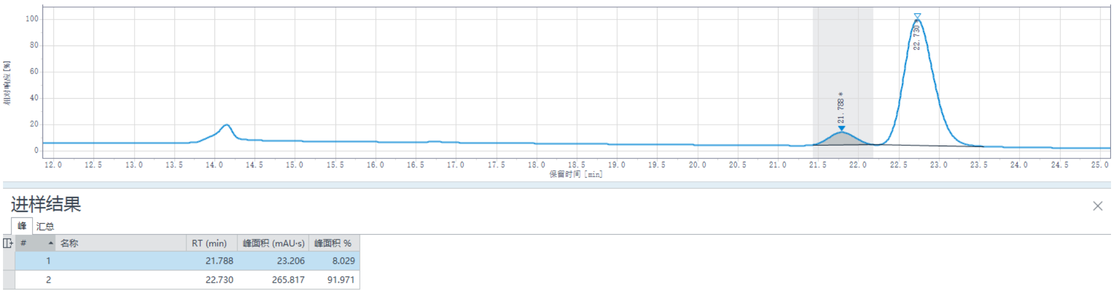

Peak (#)	Ret Time (min)	Area (%)
1	21.788	8.029
2	22.730	91.971

HPLC chromatogram (3g)

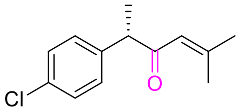

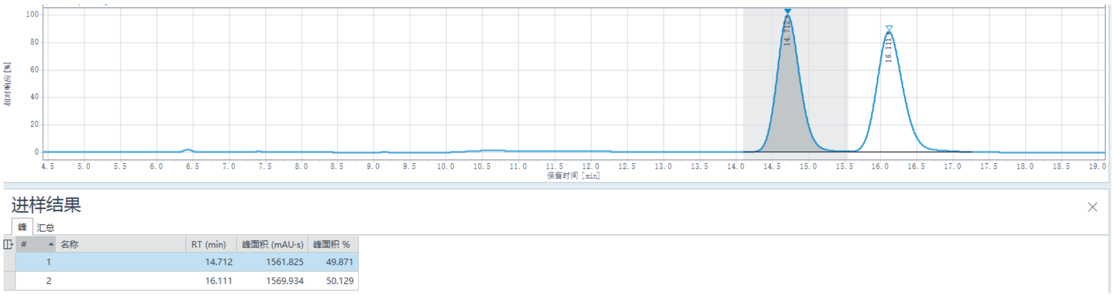

Peak (#)	Ret Time (min)	Area (%)
1	14.712	49.871
2	16.111	50.129

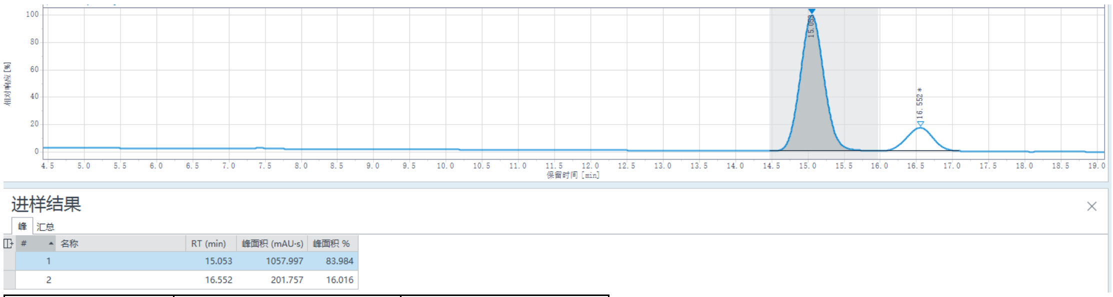

Peak (#)	Ret Time (min)	Area (%)
1	15.053	83.984
2	16.552	16.016

HPLC chromatogram (3h)

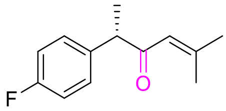

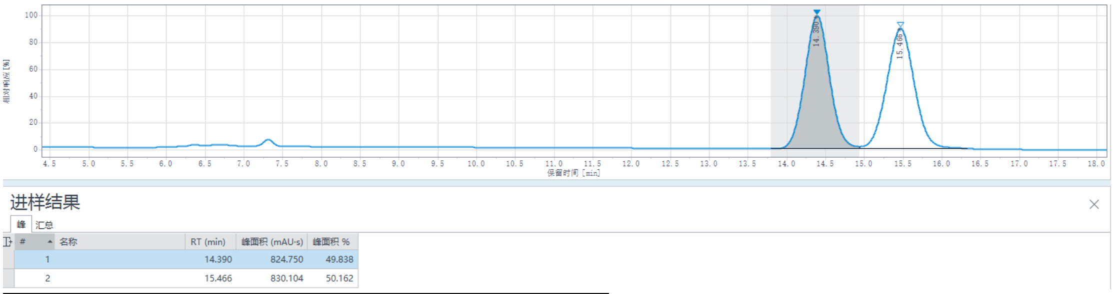

Peak (#)	Ret Time (min)	Area (%)
1	14.390	49.838
2	15.466	50.162

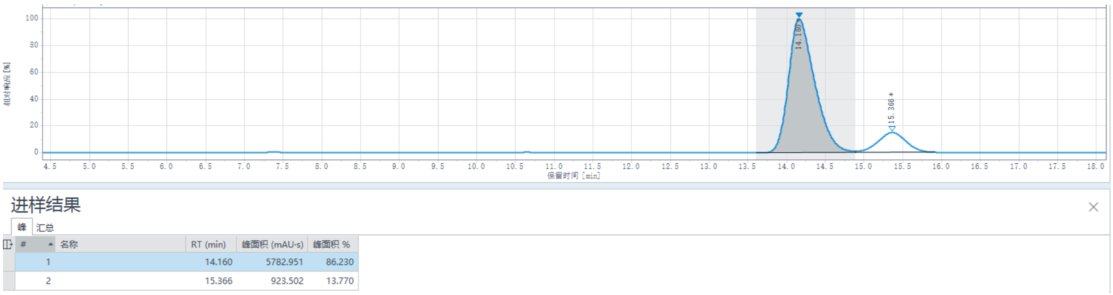

Peak (#)	Ret Time (min)	Area (%)
1	14.160	86.230
2	15.366	13.770

HPLC chromatogram (3i)

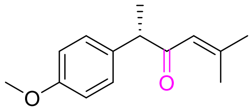

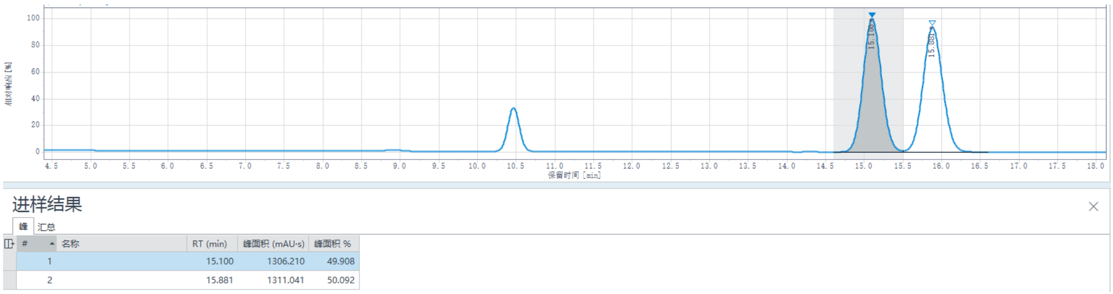

Peak (#)	Ret Time (min)	Area (%)
1	15.100	49.908
2	15.881	50.092

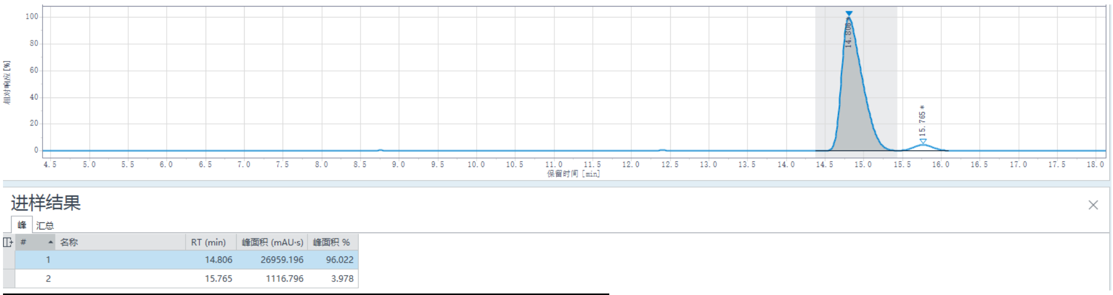

Peak (#)	Ret Time (min)	Area (%)
1	14.806	96.022
2	15.765	3.978

HPLC chromatogram (3j)

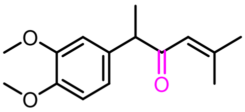

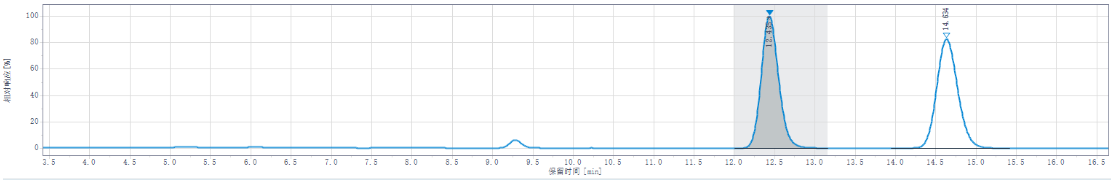

进样结果

峰 汇总

#	名称	RT (min)	峰面积 (mAU·s)	峰面积 %
1		12.435	1767.130	49.963
2		14.634	1769.755	50.037

Peak (#)	Ret Time (min)	Area (%)
1	12.435	49.963
2	14.634	50.037

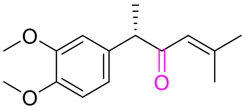

(*S*-3j)

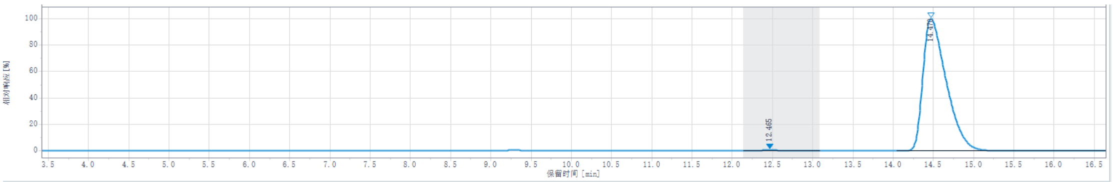

进样结果

峰 汇总

#	名称	RT (min)	峰面积 (mAU·s)	峰面积 %
1		12.465	139.137	0.549
2		14.470	25204.644	99.451

Peak (#)	Ret Time (min)	Area (%)
1	12.465	0.549
2	14.470	99.451

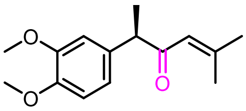

(*R*-3j)

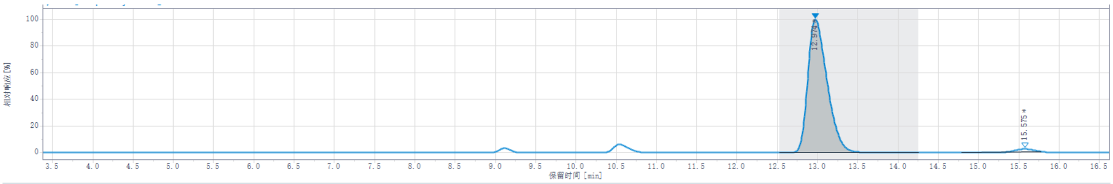

进样结果

峰 汇总

#	名称	RT (min)	峰面积 (mAU·s)	峰面积 %
1		12.974	26906.400	98.709
2		15.575	351.770	1.291

Peak (#)	Ret Time (min)	Area (%)
1	12.974	98.709
2	15.575	1.291

HPLC chromatogram (3k)

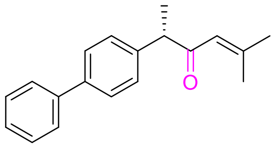

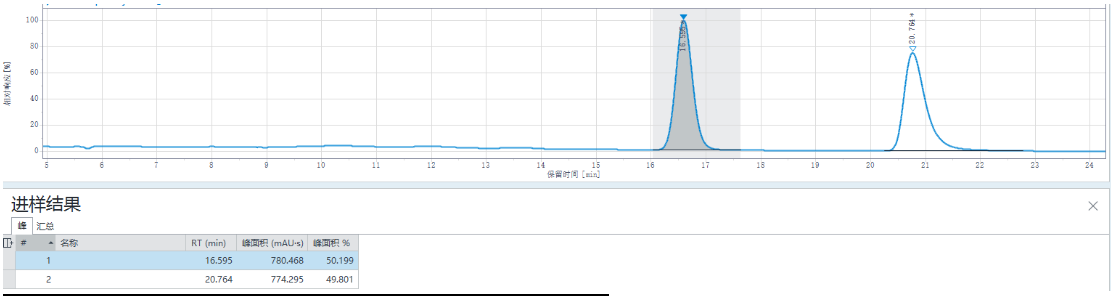

Peak (#)	Ret Time (min)	Area (%)
1	16.595	50.199
2	20.764	49.801

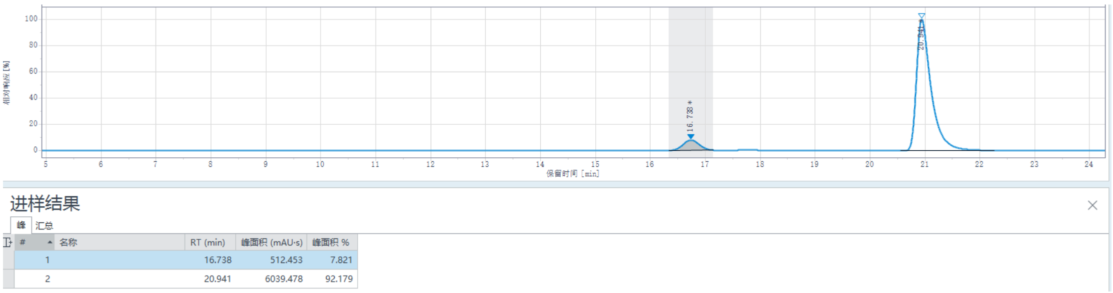

Peak (#)	Ret Time (min)	Area (%)
1	16.738	7.821
2	20.941	92.179

HPLC chromatogram (3l)

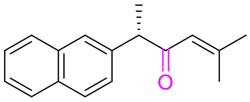

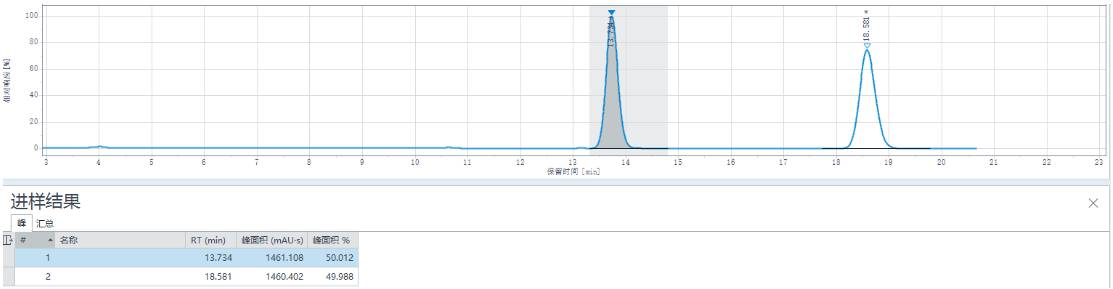

Peak (#)	Ret Time (min)	Area (%)
1	13.734	50.012
2	18.581	49.988

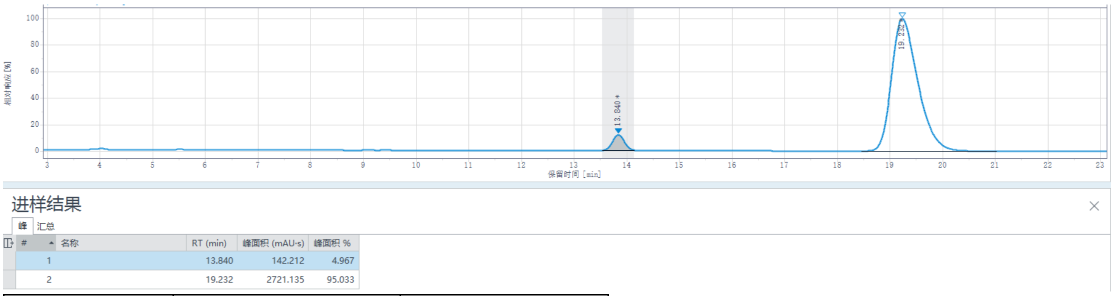

Peak (#)	Ret Time (min)	Area (%)
1	13.840	4.967
2	19.232	95.033

HPLC chromatogram (3m)

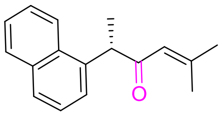

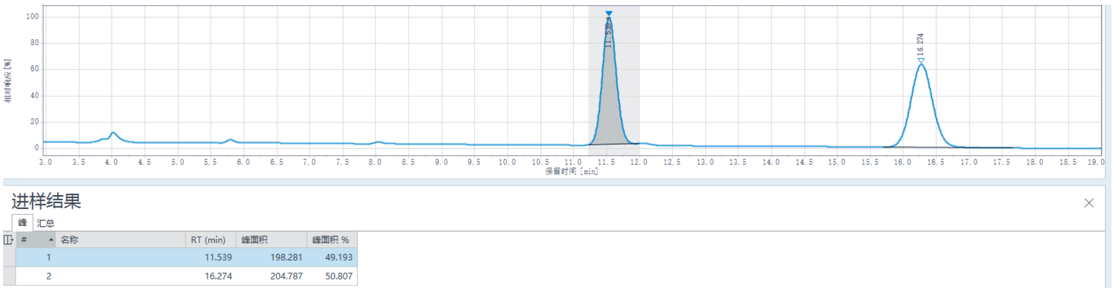

Peak (#)	Ret Time (min)	Area (%)
1	11.539	49.193
2	16.274	50.807

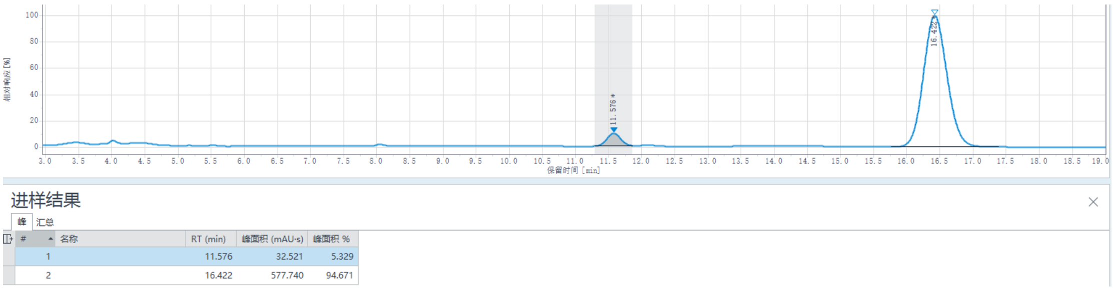

Peak (#)	Ret Time (min)	Area (%)
1	11.576	5.329
2	16.422	94.671

HPLC chromatogram (3n)

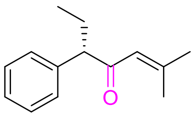

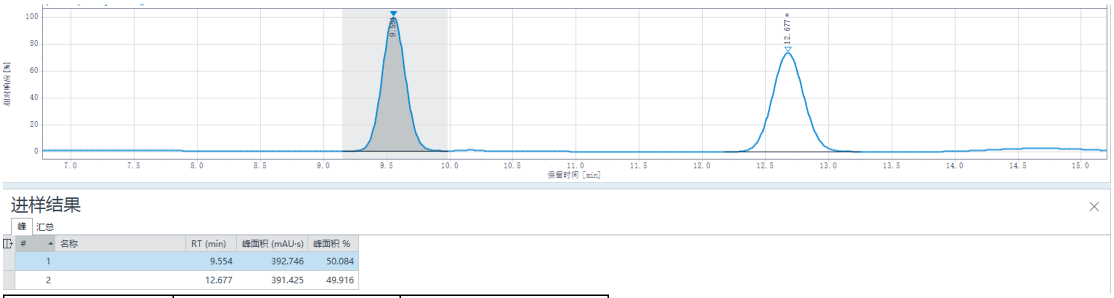

Peak (#)	Ret Time (min)	Area (%)
1	9.554	50.084
2	12.677	49.916

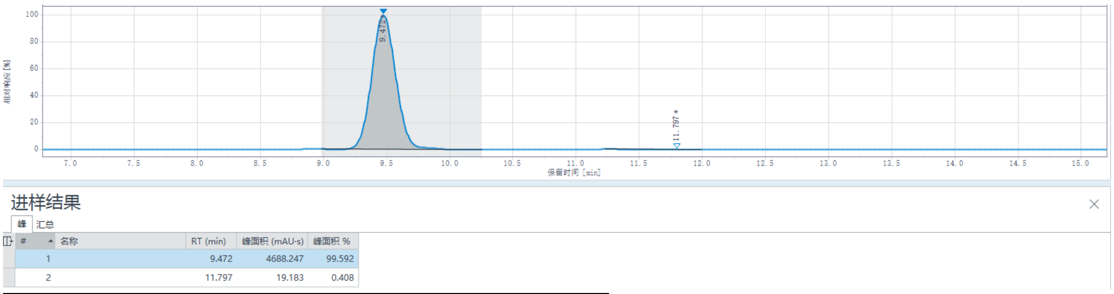

Peak (#)	Ret Time (min)	Area (%)
1	9.472	99.592
2	11.797	0.408

HPLC chromatogram (3o)

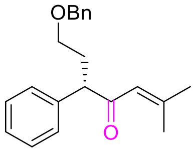

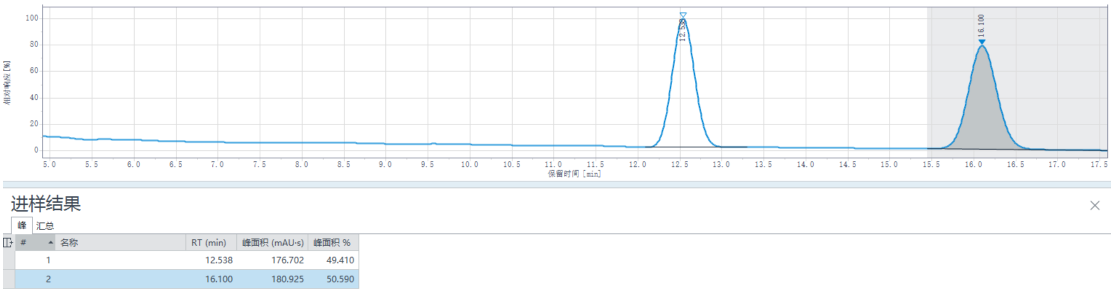

Peak (#)	Ret Time (min)	Area (%)
1	12.538	49.410
2	16.100	50.590

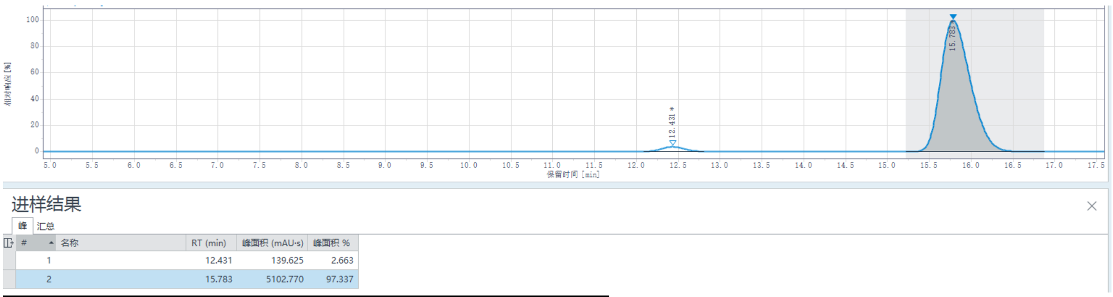

Peak (#)	Ret Time (min)	Area (%)
1	12.431	2.633
2	15.783	97.337

HPLC chromatogram (3p)

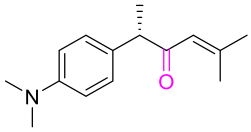

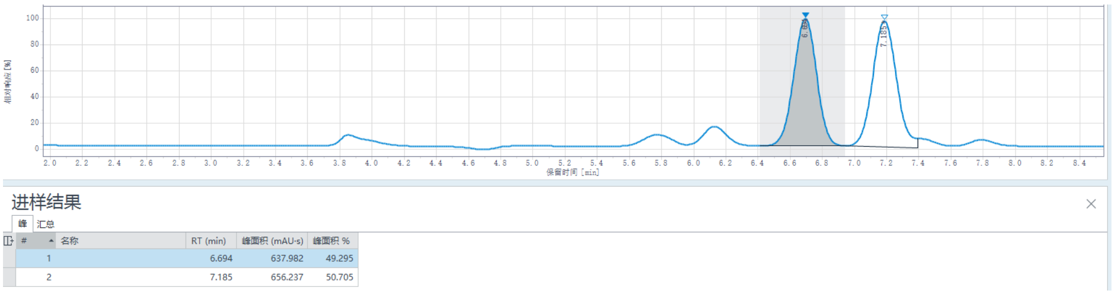

Peak (#)	Ret Time (min)	Area (%)
1	6.694	49.295
2	7.185	50.705

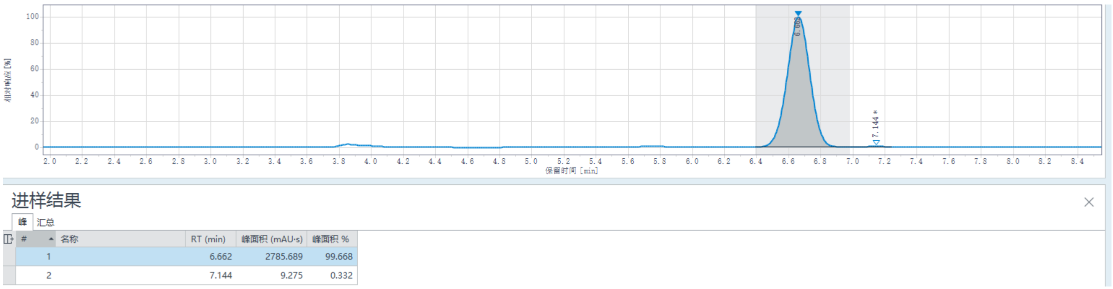

Peak (#)	Ret Time (min)	Area (%)
1	6.662	99.668
2	7.144	0.332

HPLC chromatogram (3q)

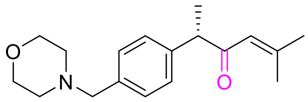

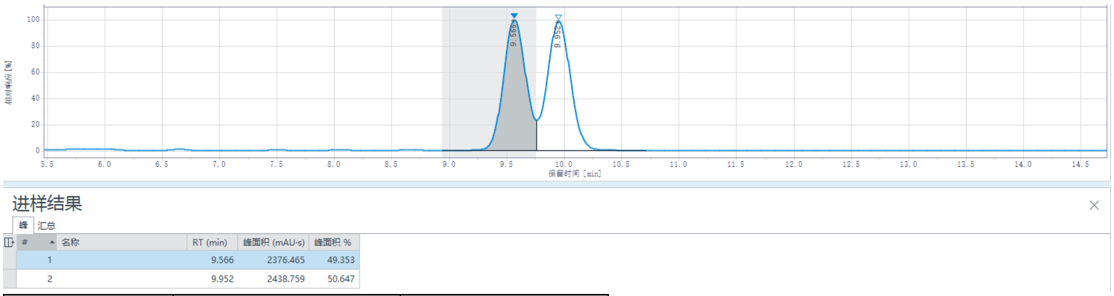

Peak (#)	Ret Time (min)	Area (%)
1	9.566	49.353
2	9.952	50.647

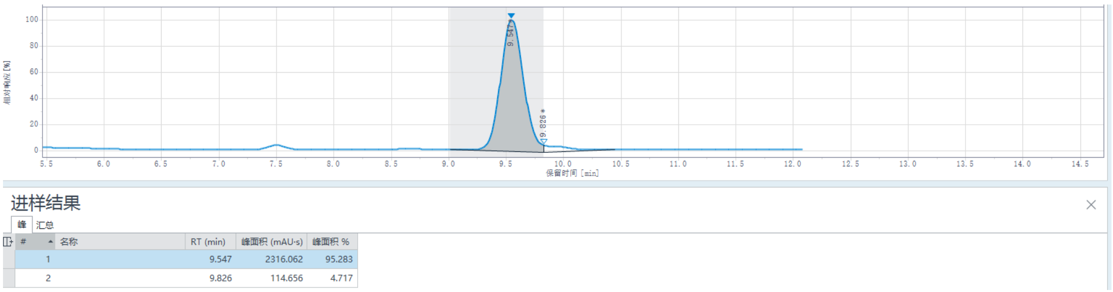

Peak (#)	Ret Time (min)	Area (%)
1	9.547	95.283
2	9.826	4.717

HPLC chromatogram (3r)

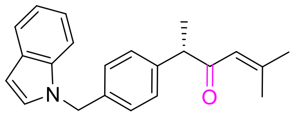

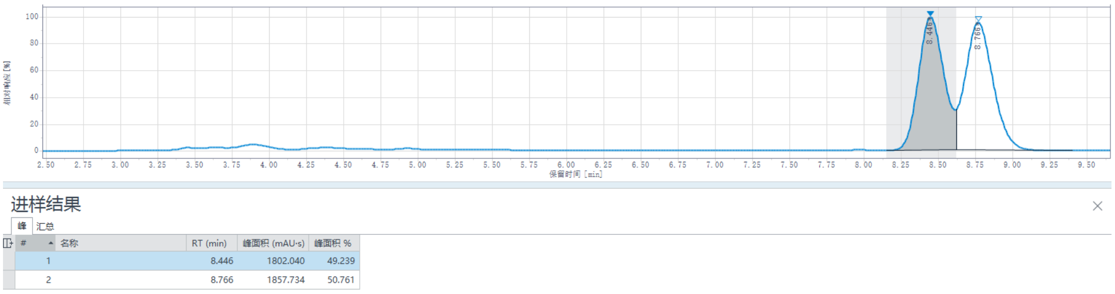

Peak (#)	Ret Time (min)	Area (%)
1	8.446	49.239
2	8.766	50.761

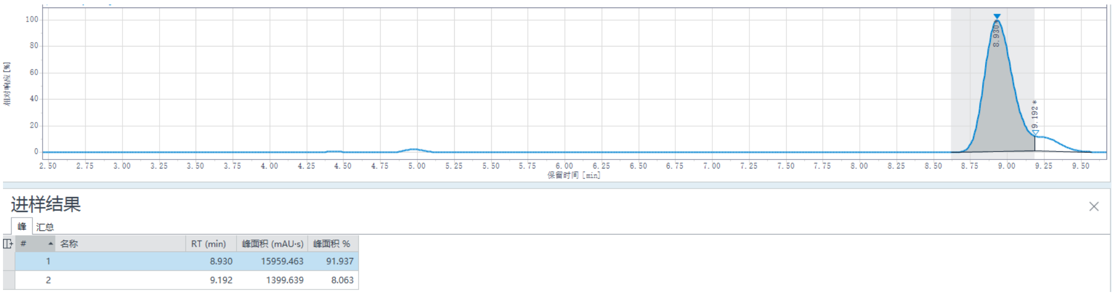

Peak (#)	Ret Time (min)	Area (%)
1	8.930	91.937
2	9.192	8.063

HPLC chromatogram (3s)

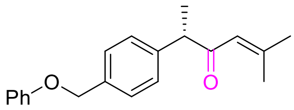

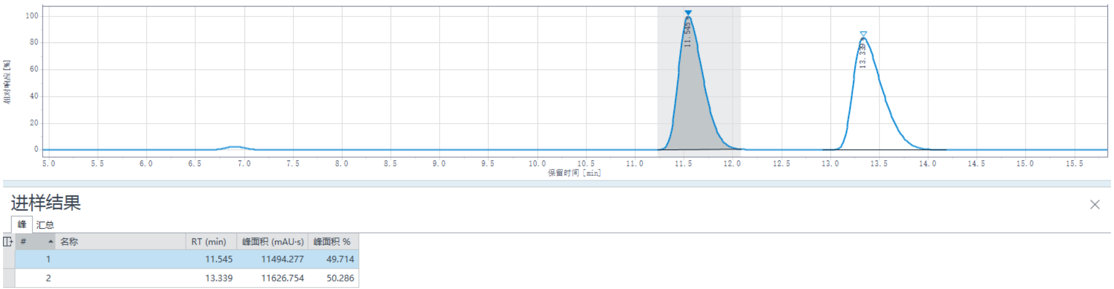

Peak (#)	Ret Time (min)	Area (%)
1	11.545	49.714
2	13.339	50.286

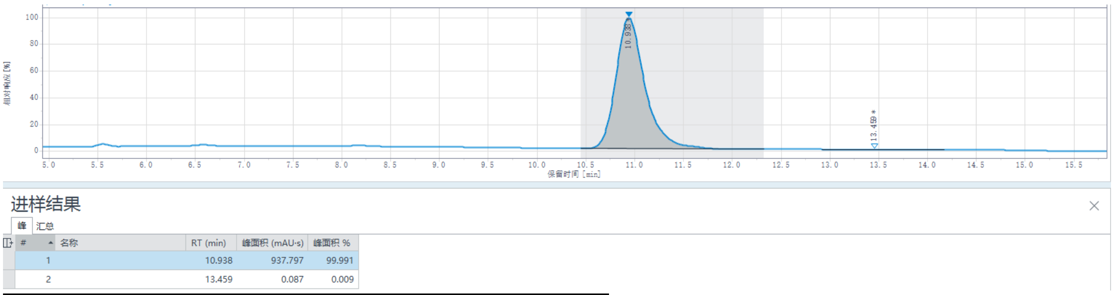

Peak (#)	Ret Time (min)	Area (%)
1	10.938	99.991
2	13.459	0.009

HPLC chromatogram (3t)

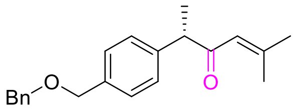

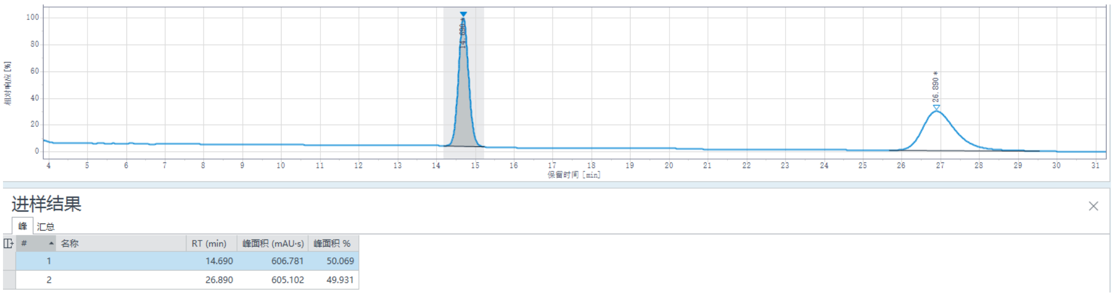

Peak (#)	Ret Time (min)	Area (%)
1	14.690	50.069
2	26.890	49.931

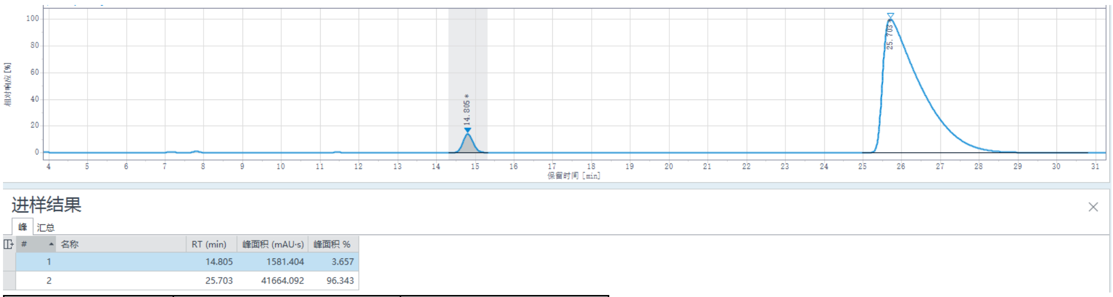

Peak (#)	Ret Time (min)	Area (%)
1	14.805	3.657
2	25.703	96.343

HPLC chromatogram (3u)

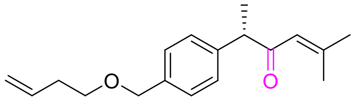

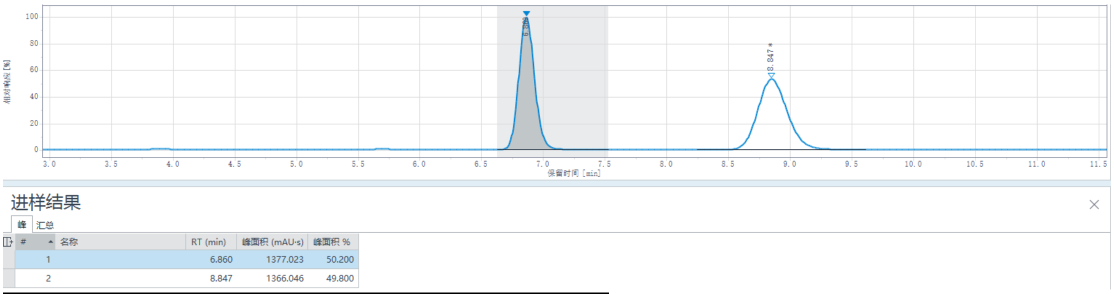

Peak (#)	Ret Time (min)	Area (%)
1	6.860	50.200
2	8.847	49.800

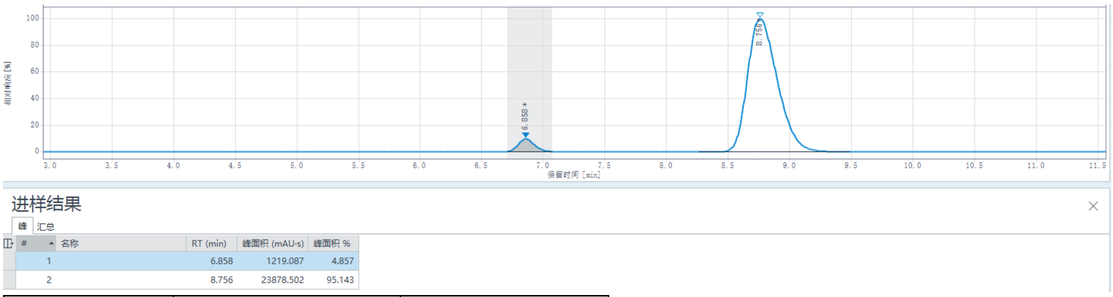

Peak (#)	Ret Time (min)	Area (%)
1	6.858	4.857
2	8.756	95.143

HPLC chromatogram (3v)

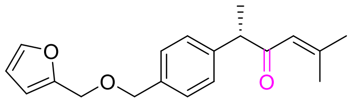

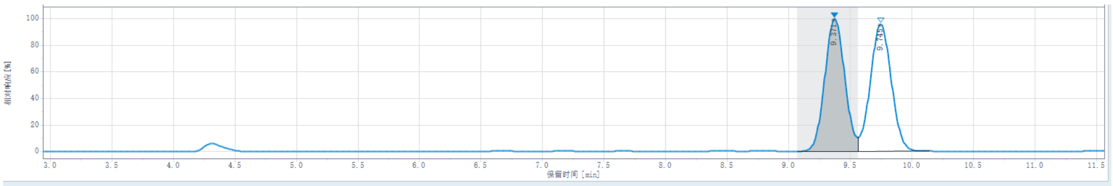

进样结果

峰 汇总

#	名称	RT (min)	峰面积 (mAU·s)	峰面积 %
1		9.371	1817.991	49.972
2		9.745	1820.011	50.028

Peak (#)	Ret Time (min)	Area (%)
1	9.371	49.972
2	9.745	50.028

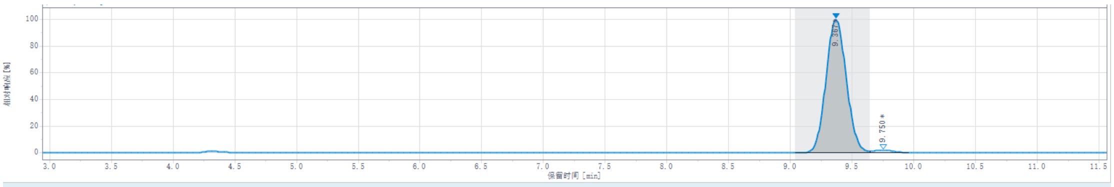

进样结果

峰 汇总

#	名称	RT (min)	峰面积 (mAU·s)	峰面积 %
1		9.367	9174.346	98.183
2		9.750	169.767	1.817

Peak (#)	Ret Time (min)	Area (%)
1	9.367	98.183
2	9.750	1.817

HPLC chromatogram (3w)

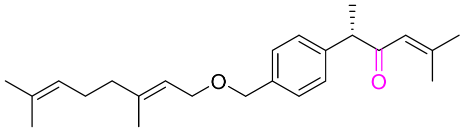

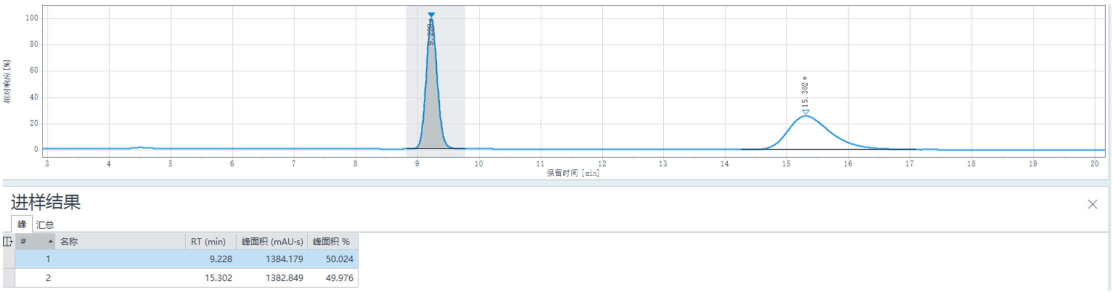

Peak (#)	Ret Time (min)	Area (%)
1	9.228	50.024
2	15.302	49.976

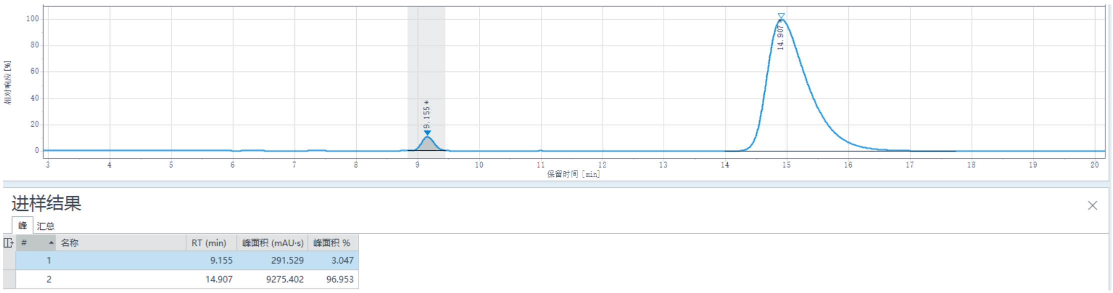

Peak (#)	Ret Time (min)	Area (%)
1	9.155	3.047
2	14.907	96.953

HPLC chromatogram (3x)

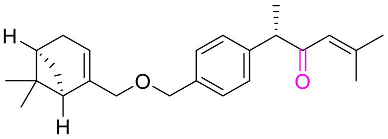

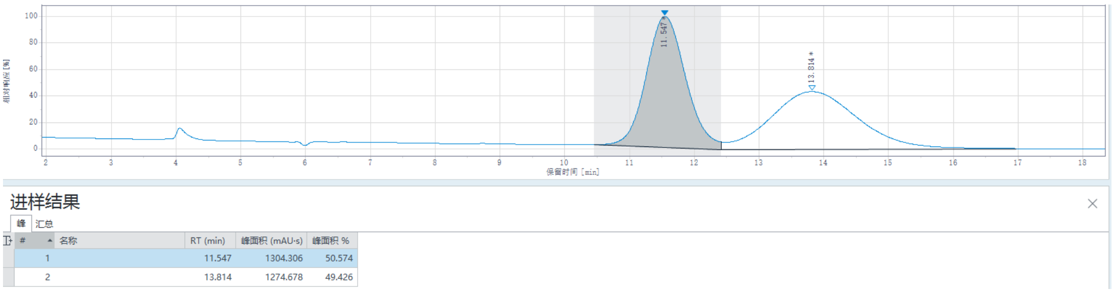

Peak (#)	Ret Time (min)	Area (%)
1	11.547	50.574
2	13.814	49.426

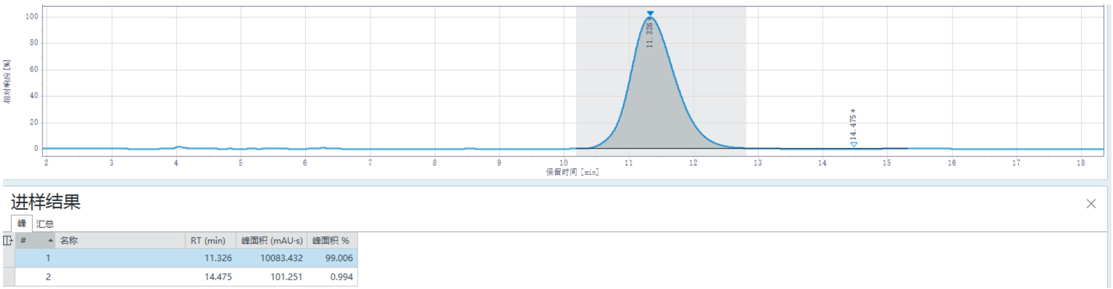

Peak (#)	Ret Time (min)	Area (%)
1	11.326	99.006
2	14.475	0.994

HPLC chromatogram (3y)

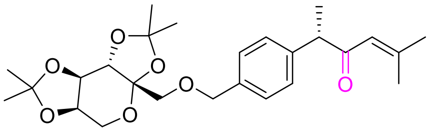

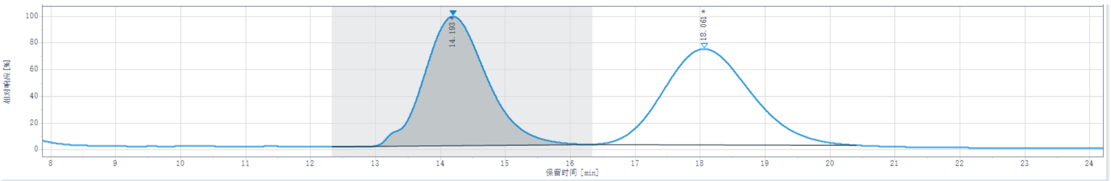

进样结果

峰 汇总

#	名称	RT (min)	峰面积 (mAU·s)	峰面积 %
1		14.193	1937.710	49.144
2		18.061	2005.253	50.856

Peak (#)	Ret Time (min)	Area (%)
1	14.193	49.144
2	18.061	50.856

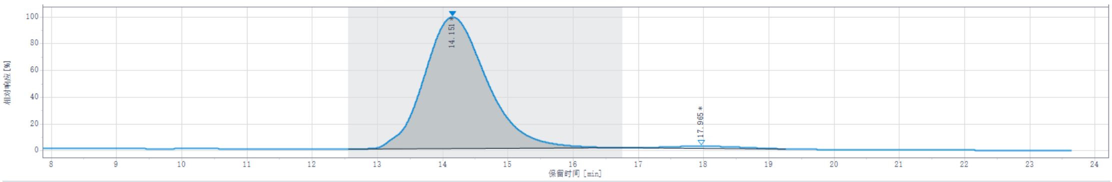

进样结果

峰 汇总

#	名称	RT (min)	峰面积 (mAU·s)	峰面积 %
1		14.151	1615.587	98.169
2		17.965	30.129	1.831

Peak (#)	Ret Time (min)	Area (%)
1	14.151	98.169
2	17.965	1.831

HPLC chromatogram (3z)

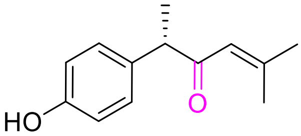

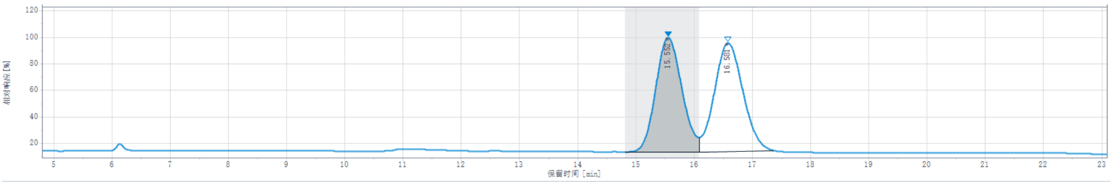

进样结果

峰 汇总

#	名称	RT (min)	峰面积 (mAU·s)	峰面积 %
1		15.552	3561.576	49.273
2		16.581	3666.626	50.727

Peak (#)	Ret Time (min)	Area (%)
1	15.552	49.273
2	16.581	50.727

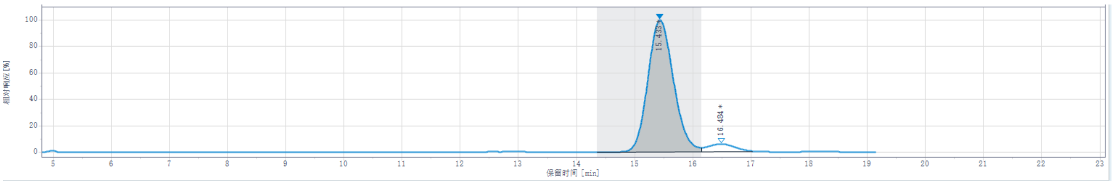

进样结果

峰 汇总

#	名称	RT (min)	峰面积 (mAU·s)	峰面积 %
1		15.433	4289.209	94.522
2		16.484	248.567	5.478

Peak (#)	Ret Time (min)	Area (%)
1	15.433	94.522
2	16.484	5.478

HPLC chromatogram (3aa)

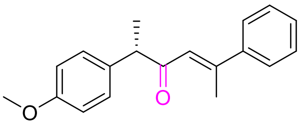

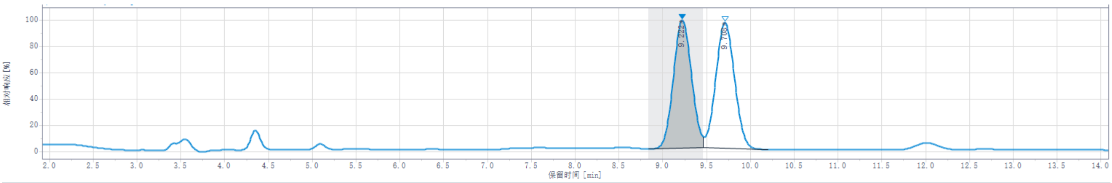

进样结果

峰 汇总

#	名称	RT (min)	峰面积 (mAU·s)	峰面积 %
1		9.222	1585.714	49.591
2		9.708	1611.891	50.409

Peak (#)	Ret Time (min)	Area (%)
1	9.222	49.591
2	9.708	50.409

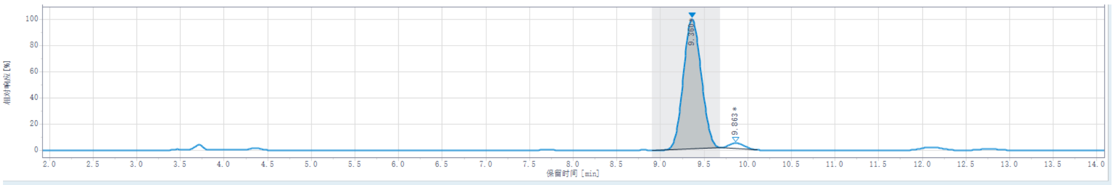

进样结果

峰 汇总

#	名称	RT (min)	峰面积 (mAU·s)	峰面积 %
1		9.360	3841.088	96.530
2		9.863	138.092	3.470

Peak (#)	Ret Time (min)	Area (%)
1	9.360	96.530
2	9.863	3.470

HPLC chromatogram (3ab)

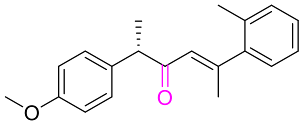

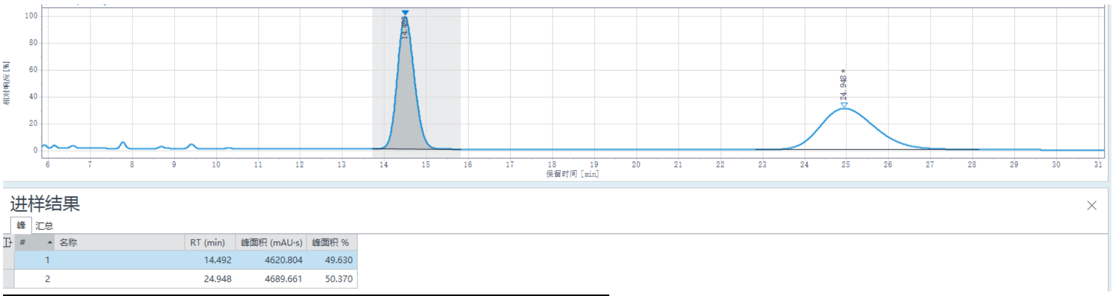

Peak (#)	Ret Time (min)	Area (%)
1	14.492	49.630
2	24.948	50.370

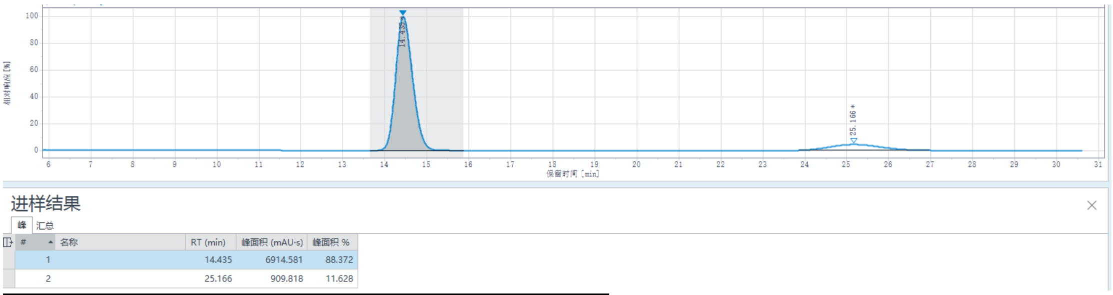

Peak (#)	Ret Time (min)	Area (%)
1	14.435	88.372
2	25.166	11.628

HPLC chromatogram (3ac)

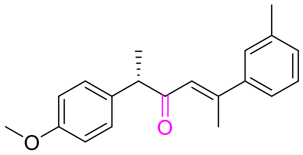

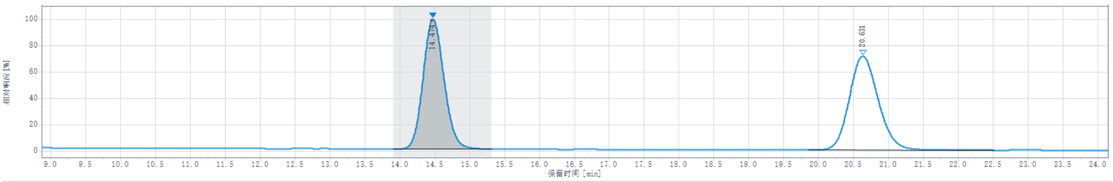

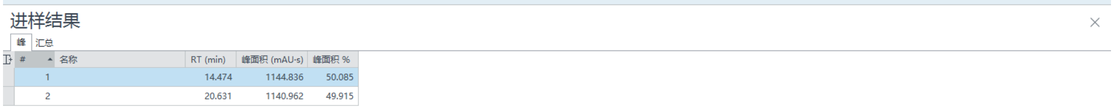

进样结果

峰 | 汇总

#	名称	RT (min)	峰面积 (mAU·s)	峰面积 %
1		14.474	1144.836	50.085
2		20.631	1140.962	49.915

Peak (#)	Ret Time (min)	Area (%)
1	14.474	50.085
2	20.631	49.915

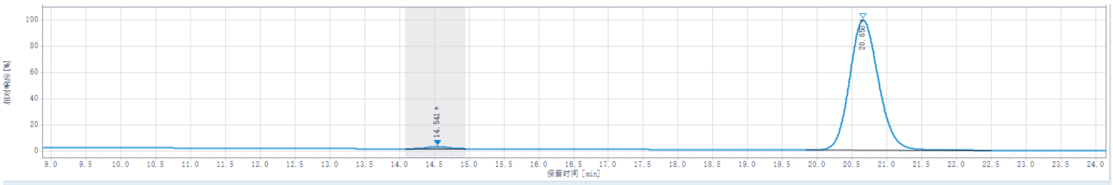

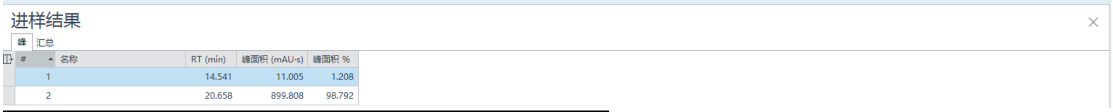

进样结果

峰 | 汇总

#	名称	RT (min)	峰面积 (mAU·s)	峰面积 %
1		14.541	11.005	1.208
2		20.658	899.808	98.792

Peak (#)	Ret Time (min)	Area (%)
1	14.541	1.208
2	20.658	98.792

HPLC chromatogram (3ad)

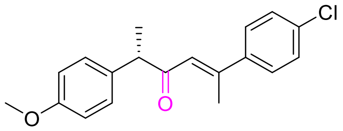

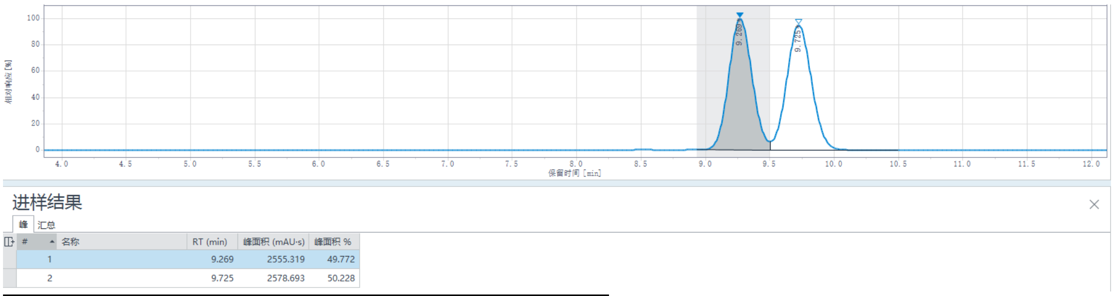

Peak (#)	Ret Time (min)	Area (%)
1	9.269	49.772
2	9.725	50.228

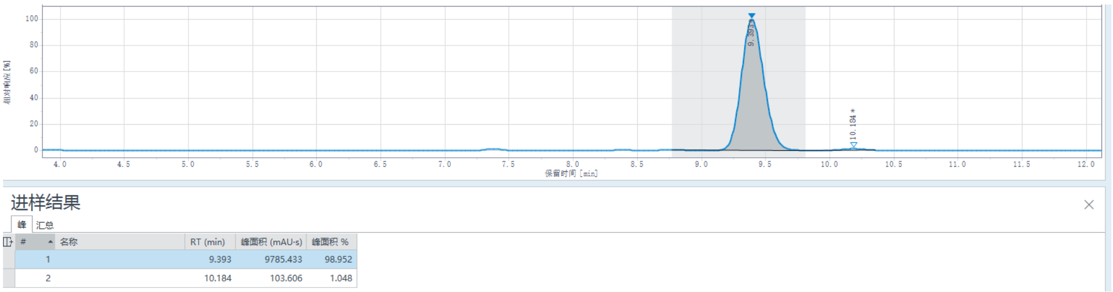

Peak (#)	Ret Time (min)	Area (%)
1	9.393	98.952
2	10.184	1.048

HPLC chromatogram (3ae)

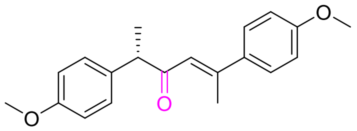

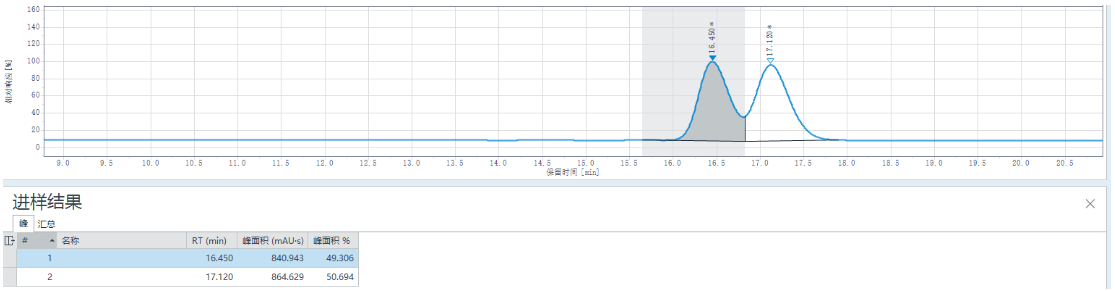

Peak (#)	Ret Time (min)	Area (%)
1	16.450	49.306
2	17.120	50.694

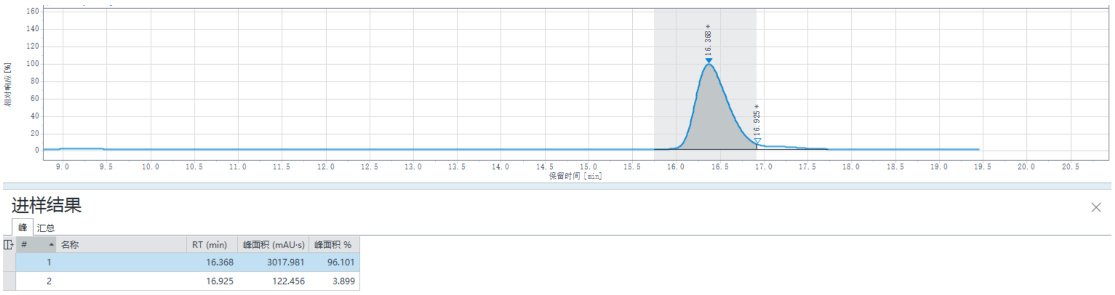

Peak (#)	Ret Time (min)	Area (%)
1	16.368	96.101
2	16.925	3.899

HPLC chromatogram (3af)

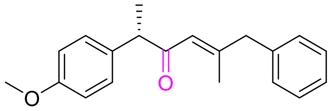

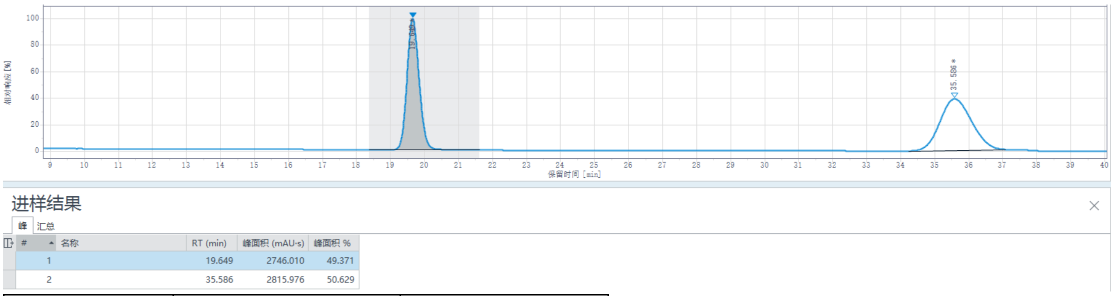

Peak (#)	Ret Time (min)	Area (%)
1	19.649	49.371
2	35.586	50.629

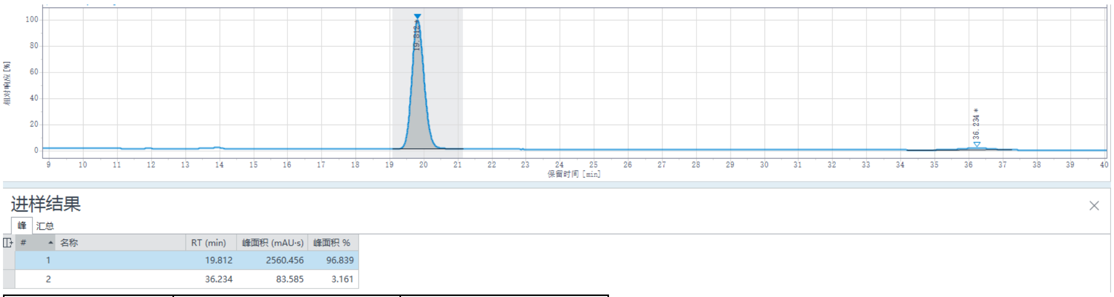

Peak (#)	Ret Time (min)	Area (%)
1	19.812	96.839
2	26.234	3.161

HPLC chromatogram ((*rac*)-5)

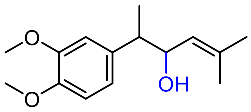

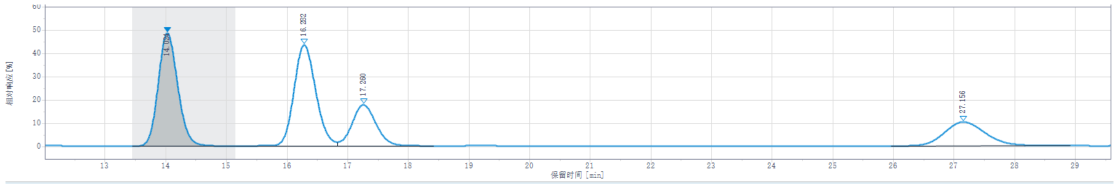

进样结果

峰 汇总

#	名称	RT (min)	峰面积 (mAU·s)	峰面积 %
1		14.024	1229.042	34.613
2		16.282	1225.259	34.507
3		17.260	550.211	15.496
4		27.156	546.259	15.384

Peak (#)	Ret Time (min)	Area (%)
1	14.024	34.613
2	16.282	34.507
3	17.260	15.496
4	27.156	15.384

HPLC chromatogram (5a)

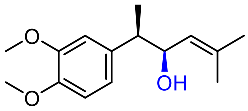

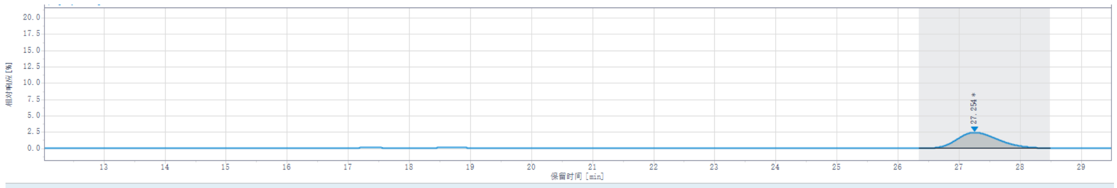

进样结果

峰 汇总

#	名称	RT (min)	峰面积 (mAU·s)	峰面积 %
1		27.254	2356.316	100.000

Peak (#)	Ret Time (min)	Area (%)
1	27.254	100.000

HPLC chromatogram (5b)

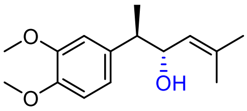

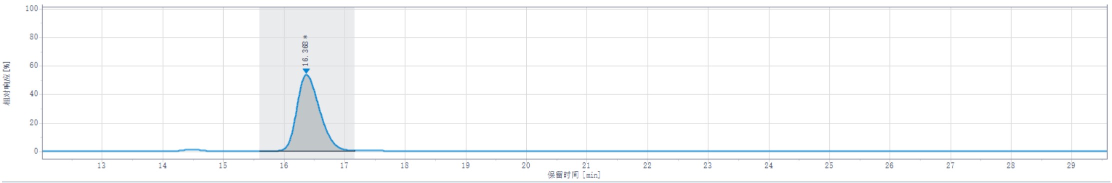

进样结果

峰 汇总

#	名称	RT (min)	峰面积 (mAU·s)	峰面积 %
1		16.368	4803.539	100.000

Peak (#)	Ret Time (min)	Area (%)
1	16.368	100.000

HPLC chromatogram (5c)

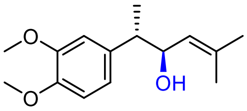

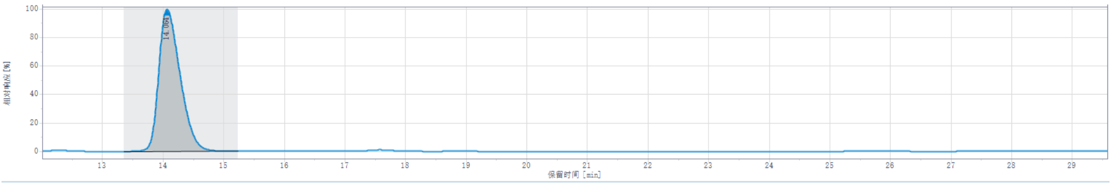

进样结果

峰 汇总

#	名称	RT (min)	峰面积 (mAU·s)	峰面积 %
1		14.064	11666.193	100.000

Peak (#)	Ret Time (min)	Area (%)
1	14.064	100.000

HPLC chromatogram (5d)

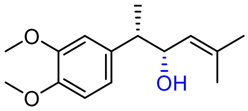

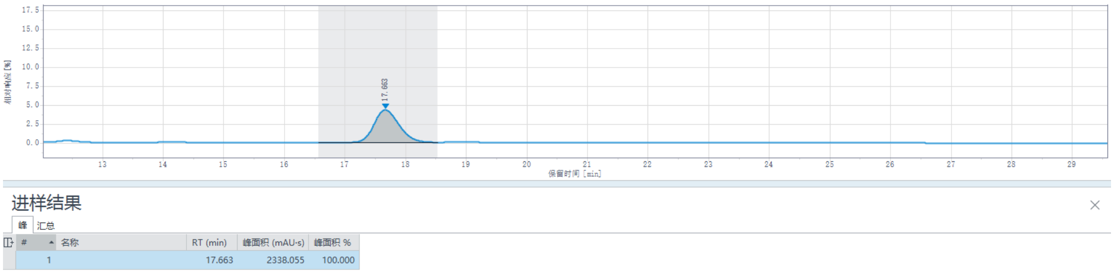

Peak (#)	Ret Time (min)	Area (%)
1	17.663	100.000

10. References

1. X. Zhang, Q. Zhou, Y. Zhou, Z. Wang, J. Wang, M. Wang, *RSC Adv.* **2023**, *13*, 30391-30400.
2. Y.-M. Wang, S. L. Buchwald, *J. Am. Chem. Soc.* **2016**, *138*, 5024-5027.
3. D. Valverde, R. Porcar, D. Izquierdo, M. I. Burguete, E. Garcia-Verdugo, S. V. Luis, *ChemSusChem.* **2019**, *12*, 3996-4004.
4. S. Ha, Y. Lee, Y. Kwak, A. Mishra, E. Yu, B. Ryou, C.-M. Park, *Nat. Commun.* **2020**, *11*, 11-18.
